# Bicyclic *N*,*S*-Acetals Containing Fused Cysteine-Amide System as New Heterocyclic Class Targeting Human Farnesyltransferase (FTase-h)

**DOI:** 10.3390/ijms26041717

**Published:** 2025-02-17

**Authors:** Fanny Danton, Mohamed Othman, Ata Martin Lawson, Amaury Farce, Emmanuelle Lipka, Alina Ghinet, Ján Moncol, Abdelhabib Semlali, Adam Daïch

**Affiliations:** 1 Normandie Univ., UNILEHAVRE, CNRS, URCOM, 76600 Le Havre, France; fanny1307@hotmail.fr (F.D.); mohamed.othman@univ-lehavre.fr (M.O.); lawsona@univ-lehavre.fr (A.M.L.); 2 Université Le Havre Normandie, UFR-ST, 25 rue Philipe Lebon, BP: 1123, 76063 Le Havre, France; 3 U995-LIRIC-Lille Inflammation Research International Center, CHU Lille, Inserm, Univ. Lille, 59000 Lille, France; amaury.farce@univ-lille.fr; 4 Faculté des Sciences Pharmaceutiques et Biologiques de Lille, 3 Rue du Pr Laguesse, B.P. 83, 59006 Lille, France; emmanuelle.lipka@univ-lille.fr; 5 UMR 1167—RID-AGE–Risk Factors and Molecular Determinants of Aging-Related Diseases, Institut Pasteur de Lille, CHU Lille, Inserm, Univ. Lille, 59000 Lille, France; alina.ghinet@junia.com; 6 Faculty of Chemistry, ‘Al. I. Cuza’ University of Iasi, B-dul Carol I, Nr. 11, Corp A, Ro-700506 Iasi, Romania; 7 Laboratoire de Chimie Durable et Santé, JUNIA, 16 rue Colson, 59000 Lille, France; 8 Department of Inorganic Chemistry, Faculty of Chemical and Food Technology, Slovak University of Technology, SK-81237 Bratislava, Slovakia; 9 Groupe de Recherche en Écologie Buccale (GREB), Faculté de Médecine Dentaire, Université Laval, Québec, QC G1V 0A6, Canada

**Keywords:** bicyclic *N*,*S*-acetal, amidation process, sulfoxidation and sulfonation reactions, farnesyltransferase inhibitor (FTI), molecular modelling

## Abstract

We report in this contribution the synthesis and in vitro biological evaluation of a novel class of chiral thiazoloisoindolinone scaffolds as potent inhibitors against human farnesyltransferase (FTase-h). The targeted products, sulfides (**4**), sulfoxides (**5**,**6**), and sulfones (**7**), containing up to three points of diversification, were obtained in a short-step sequence starting from the available and cost-effective *L*-cysteine hydrochloride (**1**), which is the source of N and S atoms and the chiral pool, and α-carbonyl benzoic acids (**2**), which are isoindolinone precursors. Concisely, the key ester intermediates (**1**) provide (a) sulfide-amides (**4**) by solvent-free amidation, (b) sulfoxides (**5**,**6**) by selective *S*-oxidation using NaIO_4_, and (c) sulfones (**7**) by oxidation using MMPP. Finally, the obtained *N*,*S*-acetal systems have shown promising inhibitory activities on FTase-h in the nanomolar range with excellent half maximal inhibitory concentration (IC_50_) values up to 4.0 nanomolar (for example, 25.1 nM for sulfide **4bI**, 67.3 nM for sulfone **7bG**, and more interesting of 4.03 nM for sulfoxide **5bG**).

## 1. Introduction

Farnesyltransferase (FTase), whose structure is currently well known [1,2], is a heterodimeric metalloenzyme capable of inducing protein prenylation by grafting a C_15_-isoprenoid fragment and activating the Ras proteins. This post-translational processing event, also called farnesylation, is associated with many pivotal functions in cells by involving two substrates which are the farnesyl pyrophosphate (FPP namely (2*E*,6*E*)-3,7,11-trimethyldodeca-2,6,10-trien-1-yl trihydrogen diphosphate. Farnesyl pyrophosphate is also known as farnesyl diphosphate (FDP)), donor of the farnesyl unit and the CaaX amino-acid (with C = cysteine, a = aliphatic and X = any amino-acid) pattern undergoing farnesylation [3]. Besides, FTase contains in its active site metallic cation Zn^2+^, which binds to CaaX fragment, a determinant of the substrate specificity of FTase requiring the presence of a bivalent magnesium species (Mg^2+^) [4] in the catalysis context.

Over the last decades, the clinical field has seen considerable progress in the treatment of many diseases. The fight against cancer remains a pharmaceutical priority today. Further research into conventional therapeutic targets such as DNA and the mitotic spindle by specialist pharmacological laboratories in both the academic and industrial sectors also remains essential. However, the discovery of new therapeutic targets is pivotal in the hope of increasing the cure rate of cancers. The discovery of FTase and its involvement in many intracellular processes has proven to be an alternative to existing methods. Due to its unique cellular properties, FTase was first studied with the aim of combating the many aspects of cancer. More recently, FTase inhibitors (FTIs) have also drawn growing interest in certain anti-parasitic therapies [5,6] and, more interestingly, in the treatment of Hutchinson–Gilford Progeria Syndrome, also called progeria [7,8].

**Figure 1:**
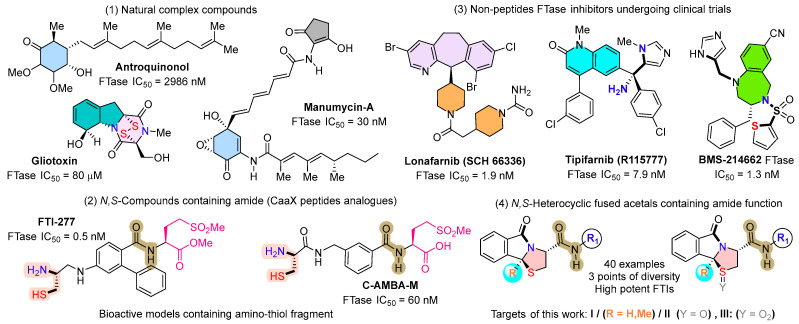
Representatives of natural and unnatural bioactive compounds containing piperazine and the families of compounds **I**, **II**, and **III** that are involved in this work.

Thus, following the discovery in the 1990s of the FTase protein, many selective inhibitors of this enzyme were quickly identified [9,10]. Two different strategies were used, focusing firstly on the analogues of the FTase substrates, the CaaX pattern, and the FPP fragment, which were rationally designed, while the second was carried out by high-throughput screening of synthetic and natural chemical libraries. From these considerations, four major classes of FTIs, in addition to a rare class of natural products, were identified according to their mechanisms of action: FPP competitive compounds, CaaX competitive compounds, inhibitors with both bis-substrates characters and non-peptide analogues. All these compounds can be classified, interestingly, into four categories (Figure 1). These include (1) natural products of complex structures such as Antroquinonol, Manumycin-A, and Gliotoxin; (2) aromatic compounds such as FTI-277 and C-AMBA-M with both 2-aminoethanethiol and amide functions as CaaX peptides analogues; (3) poly-heterocyclic products that can be considered as hybrid compounds and non-peptide inhibitors; compounds family which contains, in fact, the 1,4-*N*,*N* and/or 1,4-*N*,*S* donor ligands able to interact with metallic cation Zn^2+^ and finally, (4) the so-called bi-substrate compounds analogues not shown in Figure 1.

It is important to outline that only a few FTIs developed as anticancer agents have recently entered clinical trials on various types of tumours [7,11]. Among the small library concerned, we can observe natural Antroquinonol [12], Lonafarnib (SCH 66336) [13], Tipifarnib (R115777) [7] and BMS-214662 [14,15] (Figure 1). Beyond that, and especially since these products have not been approved by the Food and Drug Administration (FDA) principally due to their moderate to high-grade toxicity and relatively low anti-tumour activity, numerous studies demonstrated the efficacy of a high number of FTIs in the treatment of other human diseases, such as neurodegenerative disorders, cardiovascular diseases, and hepatitis as well as on progeria as outlined above [16,17,18].

## 2. Results and Discussion

In this context and with *the aim* of *developing* more efficient, scalable, and versatile approaches to produce complex aza-thia-compounds extremely few used for the inhibition of the protein FTase [19], we envisaged the incorporation of both 2-aminoethanethiol and amide molecular units into a single molecular framework containing an isoindole core [20] and synthesized a new series of hybrid derivatives of types **I**, **II** and **III** (Figure 1). Our inspiration was based on FTI-277 and C-AMBA-M ligands as interesting FTIs. The latter was pointed out to induce probably due to the presence in their structure of the reactive thiol-free function (SH) [21]. Both FTIs possess a secondary amide group (black circle in dotted line, Figure 1), especially the 2-aminoethanethiol fragment with amine NH_2_ and thiol SH free (blue circle in dotted line, Figure 1). Thus, we propose in this study to develop constrained cysteine-amide systems of types **I**, **II**, and **III** as novel classes of non-NH_2_ and SH free of FTase inhibitors without or less toxicity.

### 2.1. Chemistry

**Figure 2:**
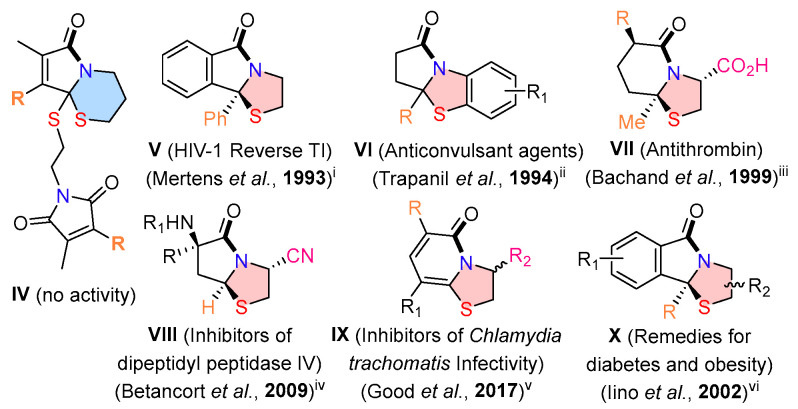
Representative examples of bioactive compounds **V**–**X** containing 1,3-thiazole nuclei. (i) [27]. (ii) [28]. (iii) [29]. (iv) [30]. (v) [31]. (vi) [32].

**Scheme 1:**
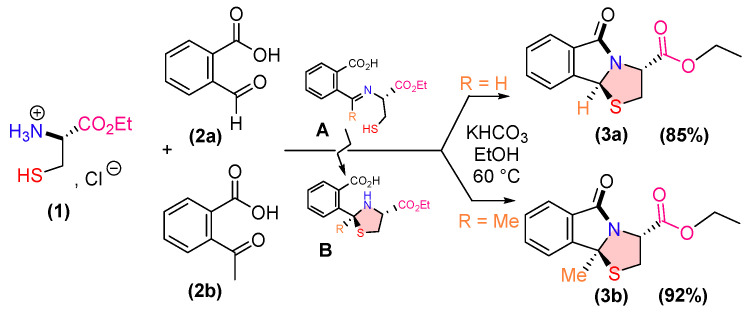
‘One-pot’ procedure access to enantiopure ethyl (9*bS*)-5-oxo-tetrahydrothiazolo[2,3-*a*]isoindole-3-carboxylate derivatives **3a** and **3b**.

In this perspective, we were interested in the synthesis of *N*,*S*-acetal containing amides **I**, **II**, and **III** (Figure 1) and the evaluation of their inhibition on the heterodimeric metalloenzyme FTase. Although five- and six-membered ring *N*,*S*-acetal fused to the aromatic or non-aromatic system from natural [20] and non-natural [22,23] sources is relatively abundant, judging by their place in the scientific documentation, curiously, none of them to our knowledge have been engaged in the inhibition of FTase except the natural bioactive Gliotoxin showing an IC_50_ of 80 nM. In addition, only one synthetic example (**IV**, Scheme 1) was used as an intermediate for the synthesis of known FTI Chaetomellic acid-A and derivatives, but no FTase activity concerning it was discussed by the authors in their report [24]. Nevertheless, numerous important contributions show that such structures have a plethora of interesting biological activities. The main parts are listed in Figure 2, including the most popular β-lactam antibiotics, penams, penems, and cephems, for which the interests are more than ever revived and topical [25,26].

As representative structures of these systems (Figure 2), enantiopure compound **V** is a selective HIV-1 reverse transcriptase inhibitor [27], and its regioisomers **VI** are effective as anticonvulsant agents [28]. In addition, the hexahydro-5*H*-thiazolo-[3,2-*a*]pyridine that bears chiral substituents **VII** were used as potent thrombin inhibitors [29]. The corresponding five-membered ring compounds **VIII** are potent dipeptidyl peptidase inhibitors [30]. Substituted dihydrothiazolo[3,2-*a*]pyridin-5-ones **IX** in both racemic and chiral forms are potent inhibitors of Chlamydia trachomatis, a global health burden due to its prevalence as a sexually transmitted infection (STI) [31]. Ultimately, dihydrothiazolo[2,3-*a*]isoindol-5(9*bH*)-one **X** bearing three points of diversification were patented for their use as remedies for diabetes and obesity [32].

Interestingly, many of the latter compounds belong to a peptidomimetics family, which are important as drug-designing tools due to the pivotal role of peptide and protein–protein interactions in molecular recognition, signalling, and especially in living systems [32]. In this context, in addition to our recent results with the unprecedented isoindolinones containing an amide function with strong FTase inhibition [33], we reasoned that natural *L*-cysteine (**1**) could serve both as nitrogen and sulphur atom sources as well as a chiral pool. In addition, 2-formyl- and 2-acetyl-benzoic acid **2a,b** (R = H, Me) can constitute valuable and cost-effective ingredients like isoindolone precursors able to promote new potent FTIs family (Scheme 1).

As a starting point of our study, the structure of *N*,*S*-acetals **3a** (R = H) and **3b** (R = Me), as key intermediates, constituted valuable platforms to access the target molecules. They were obtained by treatment of an equimolar amount of *L*-aminothiol ethyl ester as hydrochloride (**1**) and 2-formylbenzoic acid (**2a**) or 2-acetylbenzoic acid (**2b**) under azeotropic removal of water according to the published classical procedure. When the reaction was conducted with **1** and **2a** as a reaction model with a catalytic amount of p-toluene sulfonic acid [27] in toluene at reflux [34], the reaction was incomplete in all cases and furnished a difficult-to-separate mixture. The use of aminothiol **1** as hydrochloride salt seemed to be at the origin of this failure. Furthermore, the engagement of the same ingredients in absolute ethanol at room temperature with 1.1 equiv. of sodium acetate [35] has led to the formation of the expected reaction product with purification problems. In this case, only a small amount that was not totally pure was obtained. Finally, the reaction, after its completion, delivered the expected ethyl (3*R*,9*bS*)-5-oxo-2,3,5,9*b*-tetrahydrothiazolo[2,3-*a*]isoindole-3-carboxylate (**3a**) pure in 85% isolated yield when aqueous KHCO_3_ in ethanol at 60 °C were used [36]. Similarly, the same protocol with reactants **1** and **2b** provided ethyl (3*R*,9*bS*)-9b-methyl-5-oxo-2,3,5,9*b*-tetrahydrothiazolo [2,3-*a*]isoindole-3-carboxylate (**3b**) pure in 92% isolated yield. The stereochemical outcome of the cascade reaction is similar to what was demonstrated in the latter report, even if the X-ray crystallography was conducted with methyl ester instead of ethyl ester in our case.

This cascade process leading to optically pure **3a** and **3b** can be explained successively (Scheme 1): by (1) the generation of the free amine of **1** by an acid–base reaction, (2) the formation of imine **A** by amino-carbonyl dehydration, (3) the spontaneous cyclization of A via an intramolecular diastereospecific thiol addition on the imine providing thiazole intermediates **B** according to the Cram model (attack of RSH on the opposite side of the ester) and finally, (4) the bicyclic thiolactam formation via the classical intramolecular amino-acid peptide coupling of **B**. In the latter case, the reaction occurred with the help of a base in the media and left the configuration of both stereogenic centers unchanged [35,36].

**Scheme 2:**
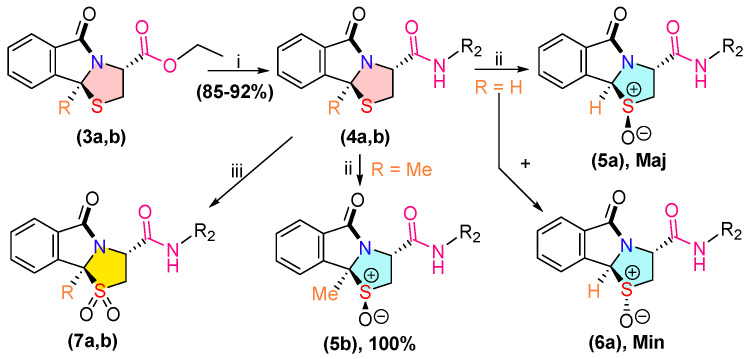
Procedures leading to chiral *N*,*S*-acetal skeleton diversity. Amidation of ester **3(a,b)** followed by sulfoxidation and sulfonation into corresponding **4(a,b)**, **5(a,b)**/**6(a)** and **7(a,b)**. Reaction conditions: (i) RNH_2_ (4 equiv.), r.t, time (see Figure 3); (ii) NaIO_4_ (1.4 equiv.), MeOH/H_2_O 9/1, r.t, 16 h (see Figure 4); (iii) MMPP (2.3 equiv.), MeOH, r.t, 16 h (see Figure 5).

**Figure 3:**
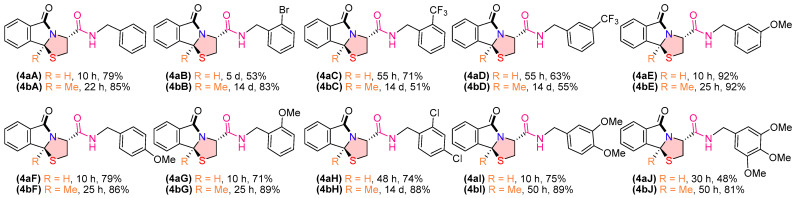
Structures of sulfides **4a(A–J)** and **4b(A–J)** obtained according to Scheme 2.

To expand our SAR study, target compounds bearing two points of diversity, such as amides **4(a,b)** and sulfones **7(a,b)** or three points of diversification **5(a,b)**/**6(a)**, were prepared as depicted in Scheme 2. Different procedures were recently developed for amidation of esters assisted by relatively weak Lewis acids such as Mg(OMe)_2_ or CaCl_2_ (60 mol%) in MeOH at 80 °C for 24 h [37] or by ZrCl_4_ (5 mol%) in acetonitrile at reflux for 5–96 h [38]. Thus, following the same latter protocol using benzylamine and ester **3a** as model substrates, the reaction delivers, in this case, the expected amide **4aA** (Figure 3) accompanied, however, by non-identified by-products in up to 10% of the reaction crude mixture difficult to isolate. In addition, when using other amines, the reaction profile remains unchanged. This is probably due to the partial formation of the stable *N*-acyliminium species by cleavage of *N*,*S*-acetal function under acid conditions [22].

In the screening of other reaction conditions, it was rapidly observed that this amidation process could be efficiently performed without the need for any catalyst assistance. In fact, the reaction of benzylamine (4 equiv.) and **3a** was complete after 10 h at room temperature solvent-free, and the expected product **4aA** was isolated pure in an interesting 79% yield (Figure 3).

The scope of *the reaction* (Figure 3) was *then extended* to other benzylamines bearing substituents at different positions of the phenyl ring with an electro-donating or electro-withdrawing effect. Gratifyingly, the process appeared to not be limited to only benzylamine. Of interest, the kinetics of the reaction of amines with *N*,*S*-acetal **3a(A–J)** seems to be faster than the one of amines with *N*,*S*-acetal **3b(A–J)**, presumably due to the steric hindrance induced by the angular methyl group at the same side of the ester group. On the contrary, the reaction yields are globally higher when R = Me (products **4b**) compared to product **4a** (R = H) except in the case of CF_3_ substituent on the phenyl ring both in ortho and meta positions, respectively, for **4a,b(C)** and **4a,b(D)**.

To establish another point of diversity necessary for SAR study, we decided to take advantage of the sulphur atom that can exist with different degrees of oxidation. Single oxygen atom donor NaIO_4_ (1.4 equiv.) in a 9:1 ratio of MeOH/H_2_O mixture at room temperature for 16 h proved to be the best conditions among numerous other attempts [39]. This interestingly avoids the formation of sulfones during *S*-oxidation of sulfides **4a(A–J)**. Under these optimized conditions, the reaction provides the expected sulfoxides (Scheme 2) as a mixture of two diastereomers **5a(A–J)** and **6a(A–J)** in a variable ratio depending on the substitution on the phenyl group of **4a(A–J)**. Even if the latter were separated with supercritical fluid chromatography (SFC) and assigned **5a(A–J)** as major diastereomers on the basis of ^1^H- and ^13^C-NMR bidimensional measurements including COSY and NOESY homo- and hetero-correlations, they were not included in our biological testing campaign (See Supplementary Materials for the structure determination of sulfoxide **5aF** as a representative example of this family). The observed diastereoselectivity during the sulfoxidation reaction seems to proceed via the *Felkin–Ahn type addition*, in which the oxidizing agent attacks the sulphur atom from the opposite side of both the amide function and the angular proton to furnish the sulfoxide **5aD** as a major diastereoisomer. This is in agreement with a rapid calculation (using Chem3D of ChemDraw Pro-17.0.0.206(121) software) of the stabilization energy of the sulfoxide **5aD,** giving a total energy of −61.9275 kcal/mol. Of interest, numerous attempts using the same calculation protocol starting from the sulfoxide **6aD** furnished in all cases a total energy of −61.2950 kcal/mol, which corresponds, in fact, to the one obtained for the above diastereoisomer **5aD** being obtained by isomerization of **6aD**. From these preliminary results, the more stable species seems to be the diastereoisomer **5aD** obtained as a major isomer. In addition, the bulky amide group, compared to the angular group H (or Me), seems to control the stereochemistry outcome of the sulfoxidation reaction. Of interest, the diastereospecificity of the sulfoxidation reaction of **5b(A–J)**, proven by the X-ray crystallography analysis of **5bA** (with R = Me), claimed favourably of this hypothesis, in which the amide function is at the origin of the stereochemistry outcome of this reaction. (See Supplementary Materials for sulfoxides **5Ad/6aD** as a representative example of this family and the X-ray analysis of **5bA**).

**Figure 4:**
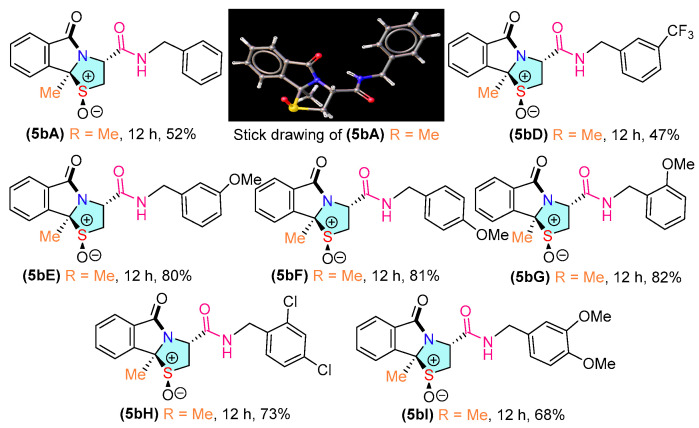
Structures of sulfoxides **5b(A,D–I)** according to Scheme 2 and the stick drawing from the X-ray analysis of compound **5bA**.

Interestingly, by using the same protocol with 2.3 equiv. of NaIO_4_ in only MeOH as solvent (the addition of water as in the case of sulfoxides does not bring improvement to the reaction), sulfoxidation of **4b(A–J)** with R = Me occurred diastereospecifically and delivered **5b(A–J)** as the sole reaction products with the structure secured by X-ray crystallography analysis of **5bA** (Figure 4) [40]. As shown in Figure 4, the reaction was complete after 12 h, and the reaction yields ranged from 47% to 82%, with the lowest yield again for the CF_3_ derivative **5bD**.

The oxidation of racemic or chiral sulfides to corresponding sulfoxides or sulfones in one or two steps has attracted a great deal of attention for centuries. After a large screening of experimental conditions (not discussed in the manuscript), the sulfonation was carried out directly in one step from sulfides **4a,b** by using 2.3 equivalents of the commercially available MMPP (magnesium monoperoxy-phthalate hexahydrate) in MeOH at room temperature for 12 h of the reaction [41]. For comparison reasons with sulfoxides, notably derivatives **5b(A,D–I)**, the reaction was conducted by only using sulfides **4a,b(A,D–I)** as starting materials. In addition, the reaction seemed to be generalizable to other phenyl-containing substituents, proceeded smoothly, and the expected sulfone products **7a,b(A,D–I)** were isolated pure after silica gel column purification in 39–91% yields as summarized in Figure 4.

Finally, the structure of all products and intermediates reported in this paper was secured and confirmed by their IR, ^1^H, and ^13^C NMR spectra, including DEPT programs, COSY and NOESY measurements in certain cases, and HRMS analyses. Ultimately, the structures of sulfoxide compounds **5b(A–J)** obtained by diastereospecific oxidation of sulfides **4b(A–J)** (R = Me) were secured by X-ray analyses.

### 2.2. Biological Evaluation of the Targeted Products and First SAR Aspects

With 40 new products in hand, the determination of their inhibitory activity on human farnesyl-transferase (FTase) in vitro was first undertaken. Dimethyl sulfoxide (DMSO) was used as the negative control, and Chaetomellic acid A was the positive reference in all assays. At the outset, and in order to select only significant results, products with an inhibitory power greater than 60% at an initial screening concentration of 100 µM were retained for the determination of their IC_50_ and, therefore, for further discussion. Moreover, given the number of products tested, they are grouped into three subfamilies on the basis of the degree of the sulphur atom oxidation (series of sulfides, series of sulfoxides, and series of sulfones), and the results obtained are grouped in Table 1, Table 2 and Table 3. Interestingly, the biological results of these frameworks allowed us to outline the first SAR in the chiral new thiazoloisoindolinone series.

Initial SAR studies focused on assessing the different effects of two points of diversity on the chiral thiazoloisoindolinone scaffolds bearing simply the thioether function **4a,b**. As highlighted in Table 1, the presence of electro-donating methoxy substituent on the *para* position of the phenyl group is important for the biological activity, and the two electro-donating methoxy substituents both in *meta* and *para* positions as in compound **4bI** were advantageous to FTase inhibition (FTase IC_50_ = 25.1 nM with FTI% = 67.7). These results clearly demonstrate the pivotal methoxy group itself and its position of the phenyl group and the superiority of the products of the series **4b** with R = Me (right part of the table) to the detriment of the series **4a** (R = H in the left part of the table). Moreover, the presence of Br, Cl, and CF_3_ substituents in the phenyl group generally diminished the inhibitory potential. The presence of the compounds of two methoxy groups seemed to be the best compromise for this series in comparison to products bearing one or three methoxy groups (compare trimethoxylated compound **4bJ** (IC_50_ = 4.346 μM) with dimethoxy-analogue **4bI** (IC_50_ = 25.1 nM, Table 1).

In order to improve the FTase activity, a series of **seven** sulfoxides **5b(A,D–I),** including both the angular methyl group and methoxy substituents, were designed. Two other electro-withdrawing substituents (Cl, CF_3_) in addition to non-substituted benzyl amide were considered (Figure 3). As shown in Table 2, the dimethoxy-benzyl amide derivative **5bI** resulted in a complete loss of inhibitory activity. On the contrary, in this sulfoxide series, derivative **5bG** bearing only one methoxy group in the *ortho* position displayed excellent activity against FTase (IC_50_ = 4.03 nM). Interestingly, the oxygen atom belonging to the sulfoxide function of the cyclic *N*,*S*-acetal derivative **5bG** seems to compensate for the loss of a second methoxy group on the phenyl group.

By changing the oxidation degrees of the sulphur atom as in sulfones **7a,b(A,D–I)**, we generally observed the loss of efficiency on FTase. However, in this series of sulfones, the methyl angular substituted derivatives **7b** were slightly more active than derivatives **7a** bearing hydrogen in the same position except in the case of **7bI** (R = Me) compared to **7aI** (R = H) (See the last line of Table 3). The *ortho* methoxy substituent in sulfone **7bG** (R = Me) seemed the best pharmacomodulation in the series, exhibiting an interesting IC_50_ of 67.3 nM. Again, the presence of electro-withdrawing substituents (Cl, CF_3_) on benzyl amide derivatives was detrimental to the FTase activity as in the previous series. The product **7bH** presented, however, an important inhibition percentage of 76%, but its intrinsic fluorescence in competition with the enzyme suggested a false positive and did not make the evaluation of its real inhibitory potential possible. To understand the importance of each group/substituent in the structure of newly identified FTase inhibitors obtained, molecular modelling studies were further realized. Their binding profile in the active site of the target protein and the results are summarized in Figure 6.

### 2.3. Docking

FTase inhibitors identified from the three different synthesized series (*series of sulfides*: **4aC**, **4aF**, **4aI**, **4bF**, **4bI,** and **4bJ**; *series of sulfoxides*: **5bE** and **5bG**; *series of sulfones*: **7bG**) were further docked in the active site of the target protein (Figure 6).

In the series of sulfides, molecule **4aC** is able to form a hydrogen bond with Tyr 112 through the carbonyl unit of the amide and the phenol of Tyr 112 and has eight conformations superimposed (Figure 6a). No interaction with the zinc of farnesyltransferase was detected. Molecule **4aF** interacts with a different tyrosine (Tyr 662) by a hydrogen bond between the carbonyl unit of the tricycle moiety and the phenol of Tyr 662 and has half of the conformations superimposed (Figure 6b). The same behaviour is adopted by molecule **4aI,** which forms a hydrogen bond with the same Tyr 662 via the carbonyl unit of the heterocyclic unit and has nine conformations well superimposed (Figure 6c). Now, methylated analogue **4bF** has half of the conformations that superpose badly, and the other half is scattered in the pocket. However, the hydrogen bonding is often conserved via the carbonyl amide with the Tyr 112. The best pose for compound **4bF** is represented in Figure 6d. Molecule **4bI** bearing two methoxy units has seven conformations that are well superimposed and have direct interaction with the zinc cation via the methoxy units (Figure 6e). The latter interaction may justify the excellent inhibitory activity of compound **4bI**. Bulkier trimethoxylated derivative **4bJ** seems to be less tolerated than the dimethoxy analogue **4bI,** even if a similar interaction exists (Figure 6f). In fact, small lots of poorly superimposed conformations can be distinguished, two of which stand out with seven representatives, as presented in Figure 6f.

Docking in the series of sulfoxides started with compound **5bE**. Three sets of conformations were retrieved for sulfoxide **5bE,** and the most important is represented in Figure 6g, in which no important interaction was noticed. Strong inhibitor **5bG** is kept in place in the active site of the protein by hydrogen bonding between its ortho-methoxy group and the Tyr 662 units (Figure 6h). Furthermore, seven conformations place the molecule in front of the zinc for favourable interaction. Finally, molecule **7bG**, representative of the sulfone series, has no interaction with the metal cation but forms two hydrogen bonds with both tyrosines Tyr 112 and Tyr 662 having ten conformations over the thirty very well superimposed in this configuration (Figure 6i). Key interactions in the current study seem to be established with Tyr 662 and Tyr 112 via chelation of the zinc cation of heterodimeric metalloenzyme FTase. The docking results largely agreed with the experimental IC_50_ values.

The entire series of sulfides regrouping twenty synthesized FTI candidates (**4aA–J**, **4bA–J**) were selected by the National Cancer Institute (NCI) for evaluation of their antiproliferative activity against 60 cancer cell lines (see Supplementary Materials for full evaluation reports).

Investigated molecules showed specific cytostatic activity against NCI-H522 non-small cell lung cancer cells (Figure 7). Indeed, seventeen molecules engendered 20–45% inhibition of the growth of these cells at a concentration of 10 µM. The most active compound on cancer cells was molecule **4bI,** which was also the best inhibitor against farnesyltransferase (IC_50_ = 25.1 nM, Table 1) among the twenty selected molecules. This methylated compound **4bI** also inhibited the growth of K-562 leukaemia (34% inhibition), UACC-257 melanoma (27% inhibition), MDA-MB-468 breast cancer (30% inhibition) and UO-31 renal cancer (20% inhibition) cells. In the non-methylated series, the analogue **4aI** proved to be the most cytostatic compound, inhibiting 36% of the NCI-H522 cells (Figure 6). Such a difference between IC_50_ on human FTase in the nanomolar range and IC_50_ on cancer cell growth in the micromolar range is characteristic of potent FTIs and was reported previously on different series of compounds (see, for example, ref. [42]). Finally, the activity against NCI-H522 measured for **4aI** and **4bI** is GI_50_ = 20.81 μM (R^2^ = 0.8923) and GI_50_ = 12.92 μM (R^2^ = 0.9546), respectively.

Moreover, none of the tested compounds at a high single dose (10 µM) in the full NCI 60 cell panel has shown a cytotoxic profile. These results raise good hope for further investigation of this series of functionalized *thiazolidine* compounds as agents fighting progeria.

## 3. Materials and Methods

### 3.1. General Information: Chemistry

Unless otherwise specified, reagents and starting materials were purchased from commercial sources and were used without further purification (suppliers: Carlo Erba Reagents S.A.S. (Val de Reuil, France), Thermo Fisher Scientific Inc. (Waltham, MA, USA), and Sigma-Aldrich Co. (Saint Louis, MO, USA)). Reactions were carried out in standard glassware. NMR spectra were recorded at room temperature on a Bruker Advance 300 spectrometer (^1^H: 300 MHz, ^13^C: 75 MHz) in deuterated chloroform (CDCl_3_), deuterated acetonitrile (CD_3_CN) or deuterated dimethyl sulfoxide (DMSO-d_6_) using TMS as internal standard (δ = 0 ppm). High-resolution ESI mass spectra were measured on a 6530 Q-TOF Agilent System spectrometer (Les Ulis, France). The separation procedure was carried out using an Interchim Puriflash 430 System equipped with a UV detector. Silicon dioxide (SiO_2_) (30 to 50 µm) from Macherey–Nagel was used as the solid phase, and a mixture of cyclohexane/ethyl acetate or dichloromethane/methanol served as eluent. Melting points were recorded on a Stuart Scientific analyzer SMP 10 apparatus and were uncorrected. Infrared spectra were performed neatly on the Perkin Elmer FT-IR spectrophotometer (Waltham, MA, USA), and only broad or strong signals were reported. Chiral supercritical fluid chromatography was performed on an SFC-PICLAB hybrid 10–20 apparatus (PIC Solution, Avignon, France). Stationary phases: The chiral analytical column used for this study was a Chiralpak AD-H, purchased from Chiral Technologies Europe (Illkirch, France). This column had dimensions 250 mm × 4.6 mm i.d. with 5 μm particle size and was coated on a silica-gel support. Specific rotations were measured on a Jasco P-2000 polarimeter for compounds never described before.

### 3.2. General Experimental Procedures

#### 3.2.1. General Procedure for the Formation of Fused *N*,*S*-Acetals **3a,b** as Starting Materials

To a stirred solution of the ketoacid **2a** or **2b** (1.0 equiv.) in ethanol was added a solution of chlorhydrate of *L*-ethyl ester of aminoethanethiol (**1**, 1.0 equiv.) and KHCO_3_ (1.0 equiv.) in water. The mixture was heated at 70 °C for 16 h. After cooling at room temperature, the solvent was evaporated in vacuo, and purification on flash chromatography was performed to provide the corresponding *N*,*S*-acetals **3a,b** (Scheme 1).

#### 3.2.2. General Procedure for the Synthesis of Amides **4a(A–J)** and **4b(A–J)** by Amidation of Fused *N*,*S*-Acetals **3a,b**

Fused *N*,*S*-acetal compound **3a** or **3b** (1.0 equiv.) and benzylamine (4.0 equiv.) were mixed at room temperature under stirring. After the starting material **3a** or **3b** had disappeared (monitored by TLC), the purification on flash chromatography was performed to furnish the corresponding tetrahydrothiazolo[2,3-*a*]isoindole-3-carboxamides **4a(A–J)** and **4b(A–J)** (Scheme 2, Figure 3).

#### 3.2.3. General Procedure for the Synthesis of *N*-Benzyl-9*b*-methyl-5-oxo-2,3,5,9*b*-tetrahydro-thiazolo[2,3-*a*]isoindole-3-carboxamides 1-Oxide **5bA** and **5b(D–I)**

Compound **4bA** or **4bD–bI** was dissolved in 9 mL of methanol and 1 mL of water, and sodium periodate (1.4 equiv.) was then added. The reaction mixture was stirred for 16 h at room temperature. After the completion of the reaction, the solvent was then evaporated under reduced pressure. The crude compound was then dissolved in 30 mL of dichloromethane and 30 mL of NaHCO_3_ saturated solution. The organic layer was collected, and the aqueous layer was extracted twice using 30 mL of dichloromethane. The combined organic layers were dried on MgSO_4_. The solvent was evaporated, and purification on flash chromatography provided carboxamides 1-oxide **5bA, 5bD–5Bi** (Scheme 2, Figure 4).

#### 3.2.4. General Procedure for the Synthesis of *N*-Benzyl-5-oxo-2,3,5,9*b*-tetrahydrothiazolo[2,3-*a*]isoindole-3-carboxamide 1,1-Dioxides **7aA**, **7a(D–G)** or **7aI**

**Figure 5:**
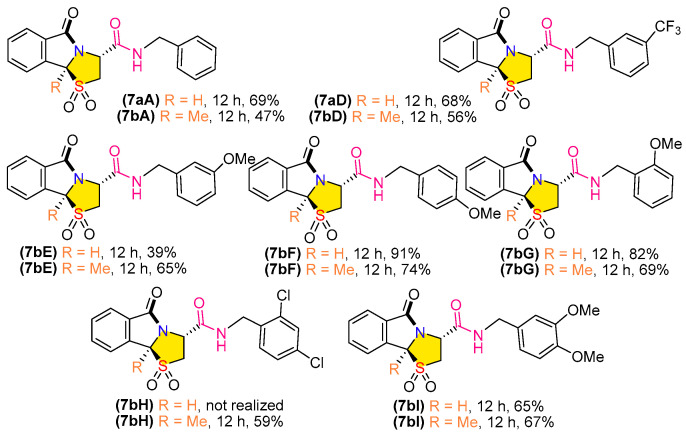
Structures of sulfones **7a,b(A,D–I)** according to Scheme 2.

**Figure 6:**
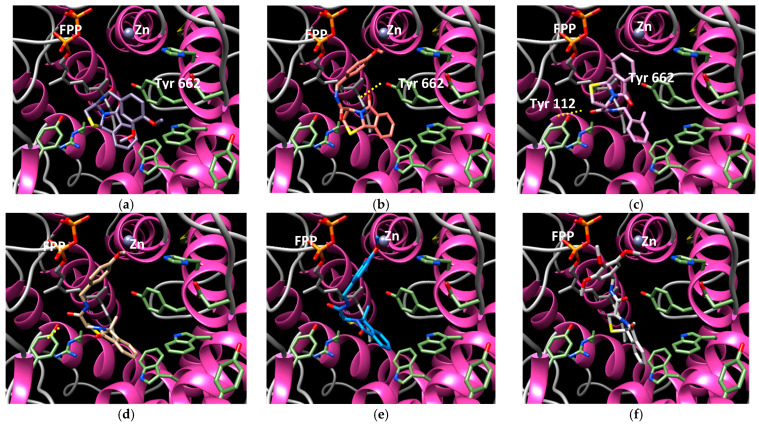

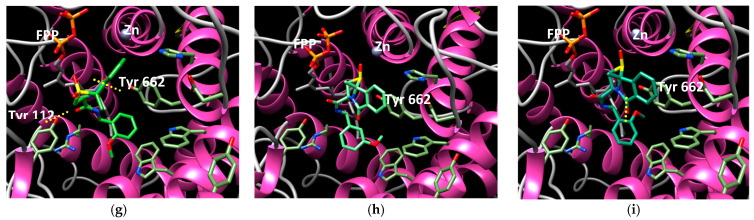
Docking of newly identified inhibitors in the active site of human FTase: Sulfides series: compounds **4aC** (**a**), **4aF** (**b**), **4aI** (**c**), **4bF** (**d**), **4bI** (**e**) and compound **4bJ** (**f**); Sulfoxides series: compound **5bE** (**g**) and compound **5bG** (**h**); and Sulfones series: compound **7bG** (**i**).

**Figure 7:**
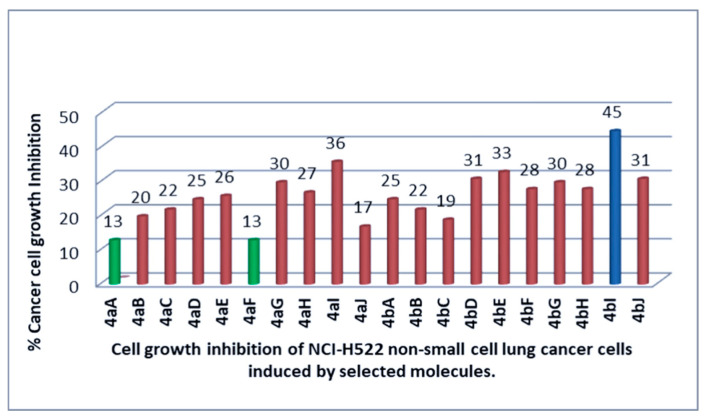
Comparison of NCI-H522 cell growth inhibition of the selected molecules **4a(A–J)** and **4b(A–J)** at 10µM concentration.

To the substrate **4aA**, **4a(D-G)** or **4aI** was dissolved in 20 mL of methanol, and MMPP (2.3 equiv.) was added. The reaction mixture was stirred for 16 h at room temperature, and the solvent was then evaporated under reduced pressure. The crude compound was then dissolved in 30 mL of dichloromethane and 30 mL of a NaHCO_3_-saturated solution. The organic layer was separated and collected, and the resulting aqueous layer was extracted twice with 30 mL of dichloromethane. The combined organic layers were dried over MgSO_4_. The solvent was evaporated in vacuo, and purification on flash chromatography provided compound **7aA**, **7a(D–G)**, or **7Ai** (Scheme 2, Figure 5).

### 3.3. FTase Inhibitory Assay [42]

Assays were realized in 96-well plates, prepared with a Biomek NKMC and a Biomek 3000 from Beckman Coulter, and read on a Wallac Victor fluorimeter from PerkinElmer. Per well, 20 µL of farnesyl pyrophosphate (10 μM) was added to 180 µL of a solution containing 2 µL of varied concentrations of potential inhibitors (dissolved in DMSO) and 178 μL of a solution composed of 10 μL of partially purified recombinant human FTase (15 µg mL^−1^) and 1.0 µL of Dansyl-GCVLS peptide (in the following buffer: 5.6 mM DTT, 5.6 mM MgCl_2_, 12 μM ZnCl_2_ and 0.2% (*w*/*v*) octyl-β-D-glucopyranoside, 52 mM Tris/HCl, pH 7.5). Fluorescence was recorded for 15 min (0.7 s per well, 20 repeats) at 30 °C with an excitation filter at 340 nm and an emission filter of 486 nm. Each measurement was reproduced twice, in duplicate. The kinetic experiments were realized under the same conditions, either with FPP as varied substrate with a constant concentration of Dns-GCVLS of 2.5 μM or with Dns-GCVLS as varied substrate with a constant concentration of FPP of 10 μM. Non-linear regressions were performed using the Excel software.

### 3.4. Cell Proliferation Assay [43]

Selected compounds were tested on a panel of 60 human cancer cell lines at the National Cancer Institute (NCI), Germantown, MD, USA. The cytotoxicity studies were conducted using a 48 h exposure protocol using the sulfo-rhodamine B assay [44].

### 3.5. X-ray Crystallography

Single-crystal X-ray diffraction data were collected on a Rigaku XtaLAB Synergy-S Dualflex diffractometer equipped with a HyPix-6000HE Hybrid Photon Counting (HPC) detector. The unit cell determination and data integration were carried out using the CrysAlisPro package from Oxford Diffraction [45]. Multi-scan correction for absorption was applied. The structures were solved with the SHELXT 2018/2 structure solution program using the Intrinsic Phasing method and refined by the full-matrix least-squares method on F2 with SHELXL 2018/3 [46,47]. Olex2 was used as an interface to the SHELX programs [48]. Non-hydrogen atoms were refined anisotropically. Hydrogen atoms were added in idealized positions and refined using a riding model. Crystal data, data collection, and structure refinement details are provided in Supplementary Materials and the corresponding CIF files. The supplementary crystallographic data can also be obtained free of charge via the following link: https://www.ccdc.cam.ac.uk/structures/ (accessed on 13 February 2025).

## 4. Conclusions

In summary, the synthesis and in vitro biological evaluation of a new class of chiral thiazoloisoindolinone scaffolds as potent inhibitors against human farnesyltransferase (FTase) is reported in this paper. The targeted products, like chiral sulfides, sulfoxides, and sulfones containing up to three points of diversification, were obtained in short two- or three-step sequences starting from available and cost-effective starting materials with L-cysteine hydrochloride as a chiral pool. The ester key intermediates of our synthetic strategy were obtained in a four-step pathway, including an acid–base reaction, imine formation by amino-carbonyl dehydration, spontaneous cyclization of the forming imine via an intramolecular diastereospecific thiol addition on the imine that provides thiazoles intermediates in accordance with the Cram model, and finally, the bicyclic thiolactam formation via the classical intramolecular amino-acid peptide coupling.

Sulfides (**I**), as the first subfamily of these chiral *N*,*S*-acetal compounds, were obtained by direct amidation in a solvent-free medium at room temperature. The latter led to a one-step procedure, using NaIO_4_ in a mixture of MeOH/H_2_O to produce sulfoxides (**II**) by diastereoselective oxidation, whose structure was secured by X-ray analysis. Ultimately, sulfones (**III**) were also obtained from sulfides (**I**) by direct and double oxidation using MMPP in MeOH without the need to isolate the sulfoxides intermediates.

In the current study, chiral fused *N*,*S*-acetal containing cysteine-amides have been highlighted as powerful novel inhibitors of human farnesyltransferase. Three derivatives in the current work displayed IC_50_ values in the small nanomolar range of 25.1 nM for sulfide **4bI**, 67.3 nM for sulfone **7bG**, and, more interestingly, 4.03 nM for sulfoxide **5bG**. From the first SAR study in the chiral thiazoloisoindolinone series proposed herein (sulfides, sulfoxides, and corresponding sulfones), we observed that the presence of a methyl group tethered to the angular position and a methoxy group (or two) on the phenyl group according to the subfamilies of the series are clearly advantageous for the FTase activity. They are definitively the best groups associated with high enzyme inhibitory activity and must be retained for the next QSAR studies. This, in fact, will constitute an important layout foundation for the further design of molecules with improved inhibitory activity that our group is planning to investigate in the future, particularly in the context of Hutchinson–Gilford progeria syndrome cells.

## Figures and Tables

**Table 1:** Inhibitory activities of sulfides **4a(A–J)** and corresponding methyl substituent **4b(A–J)** on human farnesyltransferase in vitro.

Prod	Structure	% FTI ^1,2^	IC_50_ (nM) ^1^	(R^2^) ^3^	(Log *P*) ^4^	Prod.	Structure	% FTI ^1,2^	IC_50_ (nM) ^1^	(R^2^) ^3^	(Log *P*) ^4^
**4aA**	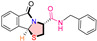	- ^5^	- ^5^	- ^5^	1.63 ± 0.71	**4bA**	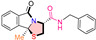	49	- ^5^	- ^5^	2.11 ± 0.72
**4aB**	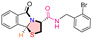	- ^5^	- ^5^	- ^5^	2.40 ± 0.76	**4bB**	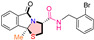	39	- ^5^	- ^5^	2.88 ± 0.77
**4aC**	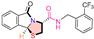	62	593.88	0.9779	2.20 ± 0.74	**4bC**	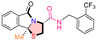	58	- ^5^	- ^5^	2.68 ± 0.75
**4aD**	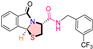	37	- ^5^	- ^5^	2.20 ± 0.74	**4bD**	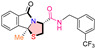	51	- ^5^	- ^5^	2.68 ± 0.75
**4aE**	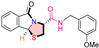	35	- ^5^	- ^5^	1.55 ± 0.72	**4bE**	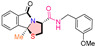	56	- ^5^	- ^5^	2.03 ± 0.73
**4aF**	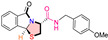	80	2725.7	0.8499	1.55 ± 0.72	**4bF**	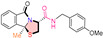	67	9213.6	0.8659	2.03 ± 0.73
**4aG**	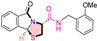	21	- ^5^	- ^5^	1.55 ± 0.72	**4bG**	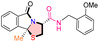	30	- ^5^	- ^5^	2.03 ± 0.73
**4aH**	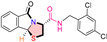	52	- ^5^	- ^5^	2.87 ± 0.73	**4bH**	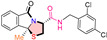	22	- ^5^	- ^5^	3.31 ± 0.74
**4aI**	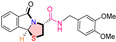	62	*Fluorescent*	- ^5^	1.37 ± 0.73	**4bI**	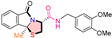	78	**25.1**	**0.9045**	**1.85 ± 0.74**
**4aJ**	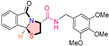	23	- ^5^	- ^5^	1.07 ± 0.74	**4bJ**	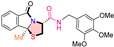	74	4346.6	0.8703	1.55 ± 0.75

^1^ Inhibition of human farnesyltransferase (FTase) at a 100 μM concentration of the tested compound. Reference used for the assay: Chaetomellic acid A (see Table 2). ^2^ Values in percentages represent the mean of two experiments. ^3^ Regression factor. ^4^ Predicted values with ACD software/ACD/Labs 2018.2.5. ^5^ Not determined.

**Table 2:** Inhibitory activities of sulfoxides **5b(A,D–I)** on human farnesyltransferase in vitro.

Prod	Structure	% FTI ^1,2^	IC_50_ (nM) ^1^	(R^2^) ^3^	(Log *P*) ^4^	Prod.	Structure	% FTI ^1,2^	IC_50_ (nM) ^1^	(R^2^) ^3^	(Log *P*) ^4^
**5bA**	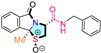	30	- ^5^	- ^5^	0.35 ± 0.72	**5bG**	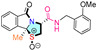	**87**	**4.03**	**0.8031**	**0.27 ± 0.73**
**5bD**	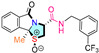	43	- ^5^	- ^5^	0.92 ± 0.75	**5bH**	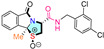	31	- ^5^	- ^5^	1.55 ± 0.73
**5bE**	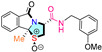	62	- ^5^	- ^5^	0.27 ± 0.73	**5bI**	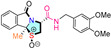	0	- ^5^	- ^5^	0.09 ± 0.74
**5bF**	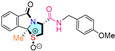	68	54,773	0.8211	0.27 ± 0.73	**Ref.**	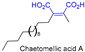	100	183.0	0.9898	- ^5^

^1^ Inhibition of human farnesyltransferase (FTase) at a 100 μM concentration of the tested compound. Reference used for the assay: Chaetomellic acid A. ^2^ Values in percentages represent the mean of two experiments. ^3^ Regression factor. ^4^ Predicted values with ACD software/ACD/Labs 2018.2.5. ^5^ Not determined.

**Table 3:** Inhibitory activities of sulfones **7a(A,D–I)** and corresponding methyl substituent **7b(A,D–I)** on human farnesyltransferase in vitro.

Prod	Structure	% FTI ^1,2^	IC_50_ (nM) ^1^	(R^2^) ^3^	(Log *P*) ^4^	Prod.	Structure	% FTI ^1,2^	IC_50_ (nM) ^1^	(R^2^) ^3^	(Log *P*) ^4^
**7aA**	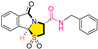	20	- ^5^	- ^5^	0.31 ± 0.71	**7bA**	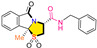	41	- ^5^	- ^5^	0.79 ± 0.72
**7aD**	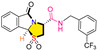	28	- ^5^	- ^5^	0.88 ± 0.74	**7bD**	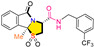	32	- ^5^	- ^5^	1.36 ± 0.75
**7aE**	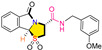	42	- ^5^	- ^5^	0.23 ± 0.72	**7bE**	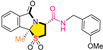	53	- ^5^	- ^5^	0.71 ± 0.73
**7aF**	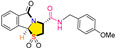	34	- ^5^	- ^5^	0.23 ± 0.72	**7bF**	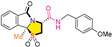	44	- ^5^	- ^5^	0.71 ± 0.73
**7aG**	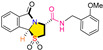	47	- ^5^	- ^5^	0.23 ± 0.72	**7bG**	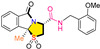	74	**67.3**	**0.8197**	**0.71 ± 0.73**
**7aH**	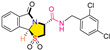	-^5^	- ^5^	- ^5^	1.55 ± 0.72	**7bH**	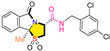	76	Fluorescent	- ^5^	1.99 ± 0.73
**7aI**	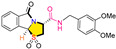	53	- ^5^	- ^5^	0.05 ± 0.73	**7bI**	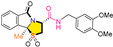	13	- ^5^	- ^5^	0.71 ± 0.74

^1^ Inhibition of human farnesyltransferase (FTase) at a 100 μM concentration of the tested compound. Reference used for the assay: Chaetomellic acid A (see Table 2). ^2^ Values in percentages represent the mean of two experiments. ^3^ Regression factor. ^4^ Predicted values with ACD software/ACD/Labs 2018.2.5. ^5^ Not determined.

## Data Availability

Data are contained within the article or the Supplementary Materials.

## Supplementary Materials

The following supporting information can be downloaded at https://www.mdpi.com/article/10.3390/ijms26041717/s1.
