# Supplementary material for: Bicyclic N,S-Acetals Containing Fused Cysteine-Amide System as New Heterocyclic Class Targeting Human Farnesyltransferase (FTase-h)
Source: Int J Mol Sci. 2025 Feb 17;26(4):1717. doi: 10.3390/ijms26041717 (PMC11855478; doi:10.3390/ijms26041717)

Supporting information

Bicyclic *N,S*-acetals containing fused cysteine-amide system as new heterocyclic class targeting human farnesyltransferase (FTase-h)

Fanny Danton [1], Mohamed Othman [1], Ata Martin Lawson [1], Amaury Farce [2,3], Emmanuelle Lipka [3,4], Alina Ghinet [4,5,6,†], Ján Moncol [7], Abdelhabib Semlali [8] and Adam Daïch [1,†,*]

1. Normandie Univ, UNILEHAVRE, CNRS, URCOM, 76600 Le Havre, France. UFR-ST, 25 rue Philipe Lebon, BP: 1123, F-76063 Le Havre Cedex, France
2. Univ. Lille, Inserm, CHU Lille, U995-LIRIC-Lille Inflammation Research International Center, F-59000 Lille, France
3. Faculté des Sciences Pharmaceutiques et Biologiques de Lille, 3 Rue du Pr Laguesse, B.P. 83, F-59006 Lille, France
4. Univ. Lille, Inserm, CHU Lille, Institut Pasteur de Lille, UMR 1167 – RID-AGE –Risk Factors and Molecular Determinants of Aging-Related Diseases, F-59000 Lille, France
5. Faculty of Chemistry, ‘Al. I. Cuza’ University of Iasi, B-dul Carol I, Nr. 11, Corp A, Ro-700506, Iasi, Romania
6. Laboratoire de Chimie Durable et Santé, JUNIA, 16 rue Colson, F-59000 Lille Cedex, France
7. Department of Inorganic Chemistry, Faculty of Chemical and Food Technology, Slovak University of Technology, SK-81237 Bratislava, Slovakia
8. Groupe de Recherche en Écologie Buccale (GREB), Faculté de Médecine Dentaire, Université Laval, Québec (QC) G1V 0A6, Canada

[†] *For corresponding author*
E-mail: adam.daich@univ-lehavre.fr
ORCID Adam Daïch: 0000-0002-6942-0519

Table of Contents

I. Full Experimental Details 3

II. Synthesis of the tetrahydrothiazolo[2,3-*a*]isoindole-3-carboxylate 3

III. General procedure for the synthesis of *N*-benzyl-5-oxo-2,3,5,9b-tetrahydrothiazolo[2,3-*a*]-isoindole-3-carboxamides 4

IV. General procedure for the synthesis of *N*-Benzyl-5-oxo-2,3,5,9*b*-tetrahydrothiazolo[2,3-*a*]-isoindole-3-carboxamide 1-oxides (**5a,6a**) *N*-benzyl-9*b*-methyl-5-oxo-2,3,5,9*b*-tetrahydrothiazolo- [2,3-*a*]isoindole-3-carboxamides 1-oxides (**5b**) 10

Chromatogram of sulfoxides mixture of **5aD** and **6AD**........ 10

V. General procedure for the synthesis of *N*-benzyl-5-oxo-2,3,5,9b-tetrahydrothiazolo[2,3-*a*]-isoindole-3-carboxamide 1,1-dioxides 17

VI. General procedure for the synthesis of *N*-benzyl-9*b*-methyl-5-oxo-2,3,5,9*b*-tetrahydrothiazolo [2,3-*a*]isoindole-3-carboxamides 1,1-dioxide 19

VII. Copies of NMR spectra 23

VIII. 2D-NMR Analysis of the sulfoxide product **5aF** 66

IX. X-Ray data including Stick drawing of compound **5bA** 69

X. NCI One Dose Mean Graph of sulfides **4a(A-J)** and **4b(A-J)** 74

I. Full Experimental Details

General remarks

Unless otherwise specified, reagents and starting materials were purchased from commercial sources and were used without further purification. Reactions were carried out in standard glassware. NMR spectra were recorded at room temperature on a Bruker Advance 300 spectrometer (^{1}H: 300 MHz, ^{13}C: 75 MHz) in deuterated chloroform ($CDCl_3$), deuterated acetonitrile (CD_3CN) or deuterated dimethyl sulfoxide (C_2D_6OS) using TMS as internal standard (δ = 0). High resolution ESI mass spectra were measured on 6530 Q-TOF Agilent System spectrometer. Separation procedure was carried out using Interchim Puriflash 430 System equipped with UV detector. Silicon dioxide (SiO_2) (30 to 50 µm) from Macherey-Nagel was used as the solid phase and a mixture of cyclohexane / ethyl acetate or dichlormethane / methanol has served as eluent. Melting points were recorded on a Stuart Scientific analyzer SMP 10 apparatus and are uncorrected. Infrared spectra were performed as neat on Perkin Elmer FT-IR spectrophotometer and only broad or strong signals are reported. Specific rotations were measured on a Jasco P-2000 polarimeter for compounds never described before.

II. Synthesis of the tetrahydrothiazolo[2,3-*a*]isoindole-3-carboxylate

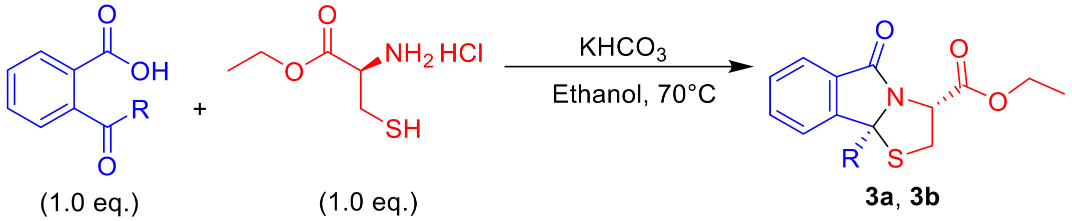

To a stirred solution of the ketoacid (1.0 eq.) in ethanol, was added a solution of chlorhydrate of *L*-ethyl ester aminoethanthiol (1.0 eq.) and $KHCO_3$ (1.0 eq.) in water. The mixture was heated at 60 °C for 16 h. After cooling at room temperature, the solvent was evaporated *in vacuo* and a purification on flash chromatography was performed to provide the corresponding *N,S*-acetals **3a,b**.

Entry	R group	Product 3
1	H	**3a**
2	M	**3b**

Ethyl (3*R*,9*bS*)-5-oxo-2,3,5,9*b*-tetrahydrothiazolo[2,3-*a*]isoindole-3-carboxylate (3a)
Colorless oil, R_f = 0.38, eluent (ethyl acetate / cyclohexane = 2/8), 2.00 g scale reaction (in 60 mL of Ethanol and 30 mL of water), 3.80 g was isolated, 92% yield.

IR (ν_{max} / cm^{-1}): 1740, 1700, 1354, 1316, 719.
^{1}H NMR (300 MHz, $CDCl_3$): δ_H 7.82 (d, *J* = 7.6 Hz, 1H), 7.60 (td, *J* = 7.4, 1.3 Hz, 1H), 7.56 – 7.44 (m, 2H), 6.09 (s, 1H), 5.24 (dd, *J* = 7.3, 4.6 Hz, 1H), 4.28 (q, *J* = 7.2 Hz, 1H), 3.69 – 3.55 (m, 2H), 1.32 (t, *J* = 7.1 Hz, 3H).
^{13}C NMR (75 MHz, $CDCl_3$): δ_C 170.4, 170.1, 145.1, 133.2, 130.7, 129.5, 124.8, 123.5, 66.4, 62.2, 57.8, 39.8, 14.3.
$[\alpha]_D^{25}$= -448.7 (c 0.290 g/100 mL, CH_2Cl_2).

Ethyl (3*R*,9*bS*)-9b-methyl-5-oxo-2,3,5,9b-tetrahydrothiazolo[2,3-*a*]isoindole-3-carboxylate (3b)
This product was already described by us in the following reference: Danton, F.; Othman, M.; Lawson, A.M.; Moncol, J.; Ghinet, A.; Rigo, B.; Daïch, A. *Chem. Eur. J.* **2019**, *25*, 6113–6118.

III. General procedure for the synthesis of *N*-benzyl-5-oxo-2,3,5,9b-tetrahydrothiazolo[2,3-*a*]-isoindole-3-carboxamides

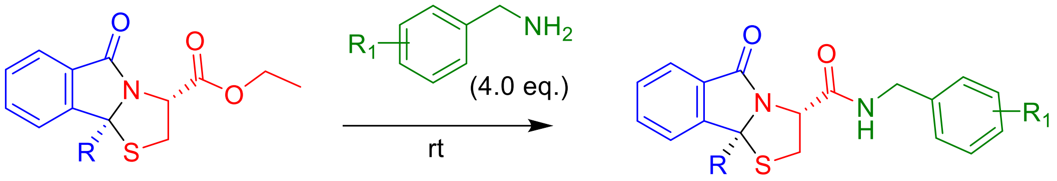

Fused *N*,*S*-acetal **3a** or **3b** (1.0 eq.) and benzylamine (4.0 eq.) were mixed at room temperature under stirring. After the starting material **3a** or **3b** was disappeared (monitored by TLC), the purification on flash chromatography was performed to furnish the corresponding tetrahydrothiazolo[2,3-*a*]isoindole-3-carboxamide **4**.

Entry	R group	R1 group	Product 4a	Entry	R group	R1 group	Product 4b
1	H	H	**4aA**	11	Me	H	**4bA**
2	H	*o*-Br	**4aB**	12	Me	*o*-Br	**4bB**
3	H	*o*-CF_3	**4aC**	13	Me	*o*-CF_3	**4bC**
4	H	*m*-CF_3	**4aD**	14	Me	*m*-CF_3	**4bD**
5	H	*m*-OMe	**4aE**	15	Me	*m*-OMe	**4bE**
6	H	*p*-OMe	**4aF**	16	Me	*p*-OMe	**4bF**
7	H	*o*-OMe	**4aG**	17	Me	*o*-OMe	**4bG**
8	H	*o,p*-2Cl	**4aH**	18	Me	*o,p*-2Cl	**4bH**
9	H	*o,p*-2OMe	**4aI**	19	Me	*o,p*-2OMe	**4bI**
10	H	*m,m',p-3OMe*	**4aJ**	20	Me	*m,m',p-3OMe*	**4bJ**

(3*R*,9b*S*)-*N*-Benzyl-5-oxo-2,3,5,9b-tetrahydrothiazolo[2,3-*a*]isoindole-3-carboxamide (4aA)

Following the application of the general procedure, this product **4aA** was isolated as colorless oil, R_f = 0.30, eluent (ethyl acetate / cyclohexane = 3/7), 0.500 g scale reaction (10 h), 0. 485 g was isolated, 79% yield.

IR (ν_{max} / cm^{-1}): 3287, 1704, 1651, 1513, 1352, 720.

^{1}H NMR (300 MHz, $CDCl_3$): δ_H 7.77 (d, *J* = 7.6 Hz, 1H, H-3), 7.61 (td, *J* = 7.5, 1.3 Hz, 1H, H-5), 7.55 – 7.44 (m, 2H, H-4, H-6), 7.37 – 7.26 (m, 5H, Har), 5.92 (s, 1H, H-8), 4.87 (t, *J* = 7.2 Hz, 1H, H-10), 4.49 (d, *J* = 5.9 Hz, 2H, H-12), 4.03 (dd, *J* = 12.0, 6.9 Hz, 1H, H-9), 3.64 (dd, *J* = 12.0, 7.5 Hz, 1H, H-9).

^{13}C NMR (75 MHz, $CDCl_3$): δ_C 172.4, 169.0, 144.3, 137.8, 133.5, 130.7, 129.9, 128.9 (x2), 127.8 (x2), 127.7, 124.9, 123.6, 67.2, 59.8, 44.0, 38.8.

HRMS (ESI$^+$): Calcd for $C_{18}H_{17}N_2O_2S$ $[M+H]^+$ 325.1005, found 325.1017.

$[\alpha]_D^{25}$= -26.9 (c 0.140 g/100 mL, CH_2Cl_2).

(3*R*,9*bS*)-*N*-(2-Bromobenzyl)-9*b*-methyl-5-oxo-2,3,5,9*b*-tetrahydrothiazolo[2,3-*a*]isoindole-3-carboxamide (4aB)

Following the application of the general procedure, this product **4aB** was isolated as yellow solid, R_f = 0.36, eluent (ethyl acetate / cyclohexane = 3/7), mp = 53-55 °C, 0.200 g scale reaction (14 days), 0.162 g was isolated, 53% yield.

IR (ν_{max} / cm^{-1}): 3306, 1676, 1659, 1552, 1324, 701.

^{1}H NMR (300 MHz, $CDCl_3$): δ_H 7.79 (dt, *J* = 8.5, 1.0 Hz, 1H), 7.63 (td, *J* = 7.5, 1.2 Hz, 1H), 7.50 (ddt, *J* = 13.5, 7.6, 1.0 Hz, 3H), 7.38 (dd, *J* = 7.6, 1.8 Hz, 1H), 7.31 – 7.23 (m, 2H), 7.13 (td, *J* = 7.7, 1.8 Hz, 1H),

4.88 (t, J = 8.2 Hz, 1H), 4.61 (dd, J = 15.1, 6.2 Hz, 1H), 4.53 (dd, J = 15.1, 6.0 Hz, 1H), 4.25 (dd, J = 12.4, 7.9 Hz, 1H), 3.76 (dd, J = 12.4, 8.6 Hz, 1H), 1.88 (s, 3H).
^{13}C NMR (75 MHz, $CDCl_3$): δ_C 172.0, 169.5, 149.2, 136.9, 133.7, 133.0, 130.1, 129.7, 129.4, 129.0, 127.9, 124.8, 123.8, 122.1, 77.4, 59.5, 44.3, 38.9, 28.0.
HRMS (ESI$^+$): Calcd for $C_{19}H_{18}BrN_2O_2S$ $[M+H]^+$ 417.0267, found 417.0287.
$[\alpha]_D^{25}$= -122.5 (c 0.255 g/100 mL, CH_2Cl_2).

(3*R*,9*bS*)-5-Oxo-*N*-(2-(trifluoromethyl)benzyl)-2,3,5,9*b*-tetrahydrothiazolo[2,3-*a*]isoindole-3-carboxamide (4aC)

Following the application of the general procedure, this product **4aC** was isolated as white solid, R_f = 0.20, eluent (ethyl acetate / cyclohexane = 2/8), mp = 118-120 °C, 0.500 g scale reaction (66 h), 0.530 g was isolated, 71% yield.

IR (ν_{max} / cm^{-1}): 3367, 1691, 1665, 1531, 1350, 719.
^{1}H NMR (300 MHz, $CDCl_3$): δ_H 7.72 – 7.69 (m, 1H), 7.61 – 7.54 (m, 2H), 7.52 – 7.43 (m, 5H), 7.35 – 7.30 (m, 1H), 5.91 (s, 1H), 4.81 (t, J = 7.2 Hz, 1H), 4.72 – 4.50 (m, 2H), 3.92 (dd, J = 12.0, 7.0 Hz, 1H), 3.57 (dd, J = 12.1, 7.5 Hz, 1H).
^{13}C NMR (75 MHz, $CDCl_3$): δ_C 172.5, 169.1, 144.3, 136.2 (d, J = 1.3 Hz), 133.5, 132.5, 130.7, 130.3, 129.9, 128.3 (q (apparent doublet), J = 30.4 Hz), 127.8, 126.2 (q, J = 5.6 Hz), 125.0, 124.5, 123.7 (q (apparent doublet), J = 273.8 Hz), 123.6, 67.2, 59.8, 40.6 (q, J = 2.7 Hz), 38.8.
^{19}F NMR (282 MHz, $CDCl_3$): δ_F -59.7.
HRMS (ESI$^+$): Calcd for $C_{19}H_{16}F_3N_2O_2S$ $[M+H]^+$ 393.0879, found 393.0883.
$[\alpha]_D^{25}$= -245.8 (c 0.340 g/100 mL, CH_2Cl_2).

(3*R*,9*bS*)-5-oxo-*N*-(3-(Trifluoromethyl)benzyl)-2,3,5,9*b*-tetrahydrothiazolo[2,3-*a*]isoindole-3-carboxamide (4aD)

Following the application of the general procedure, this product **4aD** was isolated as orange solid, R_f = 0.33, eluent (ethyl acetate / cyclohexane = 3/7), mp = 110-112 °C, 0.200 g scale reaction (70h), 0.190 g was isolated, 63% yield.

IR (ν_{max} / cm^{-1}): 3279, 1716, 1656, 1530, 1327, 719.
^{1}H NMR (300 MHz, $CDCl_3$): δ_H 7.78 (dd, J = 7.5, 1.3 Hz, 1H), 7.62 (td, J = 7.5, 1.3 Hz, 1H), 7.58 – 7.35 (m, 7H), 5.93 (s, 1H), 4.85 (t, J = 7.4 Hz, 1H), 4.54 (d, J = 6.1 Hz, 2H), 4.04 (dd, J = 12.2, 7.3 Hz, 1H), 3.65 (dd, J = 12.2, 7.5 Hz, 1H).
^{13}C NMR (75 MHz, $CDCl_3$): δ_C 172.5, 169.2, 144.2, 139.1, 133.6, 131.1 (q, J = 32.3 Hz), 131.0 (d, J = 1.5 Hz), 130.6, 129.9, 129.4, 124.8, 124.5 (q, J = 3.7 Hz, 2 x CH), 124.1 (q, J = 272.0 Hz, Cq), 123.7, 67.2, 59.7, 43.4, 38.7.
^{19}F NMR (282 MHz, $CDCl_3$) δ_F -62.6.
HRMS (ESI$^+$): Calcd for $C_{19}H_{16}F_3N_2O_2S$ $[M+H]^+$ 393.0879, found 393.0887.
$[\alpha]_D^{25}$= -234.6 (c 0.320 g/100 mL, CH_2Cl_2).

(3*R*,9*bS*)-*N*-(3-Methoxybenzyl)-5-oxo-2,3,5,9*b*-tetrahydrothiazolo[2,3-*a*]isoindole-3-carboxamide (4aE)

Following the application of the general procedure, this product **4aE** was isolated as colorless oil, R_f = 0.18, eluent (ethyl acetate / cyclohexane = 3/7), 0.500 g scale reaction (10 h), 0.617 g was isolated, 92% yield.

IR (ν_{max} / cm^{-1}): 3315, 1705, 1664, 1528, 1354, 724.

^{1}H NMR (300 MHz, CDCl$_3$): δ_H 7.80 (d, *J* = 7.4 Hz, 1H), 7.65 – 7.59 (m, 1H), 7.55 – 7.48 (m, 2H), 7.26 – 7.21 (m, 2H), 6.88 – 6.79 (m, 3H), 5.93 (s, 1H), 4.88 (t, *J* = 7.3 Hz, 1H), 4.53 – 4.40 (m, 2H), 4.04 (dd, *J* = 12.1, 7.0 Hz, 1H), 3.78 (s, 3H), 3.66 (dd, *J* = 12.1, 7.5 Hz).
^{13}C NMR (75 MHz, CDCl$_3$): δ_C 172.3, 168.9, 160.1, 144.4, 139.4, 133.5, 130.7, 129.9, 129.9, 124.9, 123.6, 119.9, 113.3 (x2), 67.2, 59.8, 55.4, 43.9, 38.9.
HRMS (ESI$^+$): Calcd for $C_{19}H_{19}N_2O_3S$ $[M+H]^+$ 355.1111, found 355.1118.
$[\alpha]_D^{25}$= -236.0 (c 0.305g/100 mL, CH_2Cl_2).

(3*R*,9*bS*)-*N*-(4-Methoxybenzyl)-5-oxo-2,3,5,9*b*-tetrahydrothiazolo[2,3-*a*]isoindole-3-carboxamide (4aF)
Following the application of the general procedure, this product **4aF** was isolated as white solid, R_f = 0.27, eluent (ethyl acetate / cyclohexane = 4/6), mp = 128-130 °C, 0.420 g scale reaction (10 h), 0. 420 g was isolated, 74% yield.
IR (ν_{max} / cm^{-1}): 3289, 1713, 1650, 1512, 1351, 719.
^{1}H NMR (300 MHz, CDCl$_3$): δ_H 7.68 – 7.65 (m, 1H), 7.56 – 7.53 (m, 1H), 7.47– 7.42 (m, 2H), 7.33 – 7.26 (bs, 1H), 7.18 (d, *J* = 8.4 Hz, 2H), 6.81 (d, *J* = 8.6 Hz, 2H), 5.88 (s, 1H), 4.80 (t, *J* = 7.1 Hz, 1H), 4.38 (d, *J* = 5.6 Hz, 2H), 3.93 (ddt, *J* = 12.1, 6.6, 1.8 Hz, 1H), 3.74 (s, 3H), 3.62 – 3.51 (m, 1H).
^{13}C NMR (75 MHz, CDCl$_3$): δ_C 172.3, 168.8, 159.2, 144.4, 133.5, 130.7, 129.9, 129.8, 129.2, 124.9, 123.6, 114.3, 67.1, 59.8, 55.4, 43.4, 38.9.
HRMS (ESI$^+$): Calcd for $C_{19}H_{18}N_2NaO_3S$ $[M+Na]^+$ 377.0930 found 377.0944.
$[\alpha]_D^{25}$= -120.2 (c 0.255 g/100 mL, CH_2Cl_2).

(3*R*,9*bS*)-*N*-(2-Methoxybenzyl)-5-oxo-2,3,5,9*b*-tetrahydrothiazolo[2,3-*a*]isoindole-3-carboxamide (4aG)
Following the application of the general procedure, this product **4aG** was isolated as colorless oil, R_f = 0.30, eluent (ethyl acetate / cyclohexane = 3/7), 0.200 g scale reaction (10 h), 0.190 g was isolated, 71% yield.
IR (ν_{max} / cm^{-1}): 3321, 1697, 1664, 1528, 1356, 724.
^{1}H NMR (300 MHz, CDCl$_3$): δ_H 7.84 (d, *J* = 7.5 Hz, 1H), 7.62 (td, *J* = 7.4, 1.3 Hz), 7.55 – 7.50 (m, 2H), 7.34 – 7.30 (bs, 1H), 7.28 – 7.23 (m, 2H), 6.92 – 6.85 (m, 2H), 5.91 (s, 1H), 4.87 (t, *J* = 7.2 Hz, 1H), 4.49 (d, *J* = 5.9 Hz, 2H), 4.03 (dd, *J* = 12.0, 6.7 Hz, 1H), 3.83 (s, 3H), 3.62 (dd, *J* = 12.0, 7.6 Hz, 1H).
^{13}C NMR (75 MHz, CDCl$_3$): δ_C 172.0, 168.7, 157.6, 144.4, 133.4, 130.7, 129.7, 129.6, 129.0, 125.8, 124.8, 123.6, 120.7, 110.5, 67.0, 59.6, 55.4, 39.8, 38.8.
HRMS (ESI$^+$): Calcd for $C_{19}H_{19}N_2O_3S$ $[M+H]^+$ 355.1111, found 355.1118.
$[\alpha]_D^{25}$= -225.3 (c 0.380 g/100 mL, CH_2Cl_2).

(3*R*,9*bS*)-*N*-(2,4-Dichlorobenzyl)-5-oxo-2,3,5,9*b*-tetrahydrothiazolo[2,3-*a*]isoindole-3-carboxamide (4aH)
Following the application of the general procedure, this product **4aH** was isolated as yellow solid, R_f = 0.37, eluent (ethyl acetate / cyclohexane = 3/7), mp = 109-111 °C, 0.500 g scale reaction (70 h), 0.530 g was isolated, 71% yield.
IR (ν_{max} / cm^{-1}): 3259, 1702, 1650, 1548, 1353, 718.
^{1}H NMR (300 MHz, CDCl$_3$): δ_H 7.84 (d, *J* = 7.4 Hz, 1H), 7.64 (td, *J* = 7.5, 1.3 Hz, 1H), 7.60 – 7.49 (m, 2H), 7.42 – 7.40 (bs, 1H), 7.38 (d, *J* = 2.1 Hz, 1H), 7.33 (d, *J* = 8.3 Hz, 1H), 7.23 (dd, *J* = 8.3, 2.1 Hz, 1H), 5.93 (s, 1H), 4.84 (t, *J* = 7.5 Hz, 1H), 4.54 (d, *J* = 6.1 Hz, 2H), 4.03 (dd, *J* = 12.2, 7.5 Hz, 1H), 3.65 (dd, *J* = 12.2, 7.5 Hz, 1H).

^{13}C NMR (75 MHz, CDCl$_3$): δ_C 172.5, 169.1, 144.2, 134.4, 134.3, 134.0, 133.6, 130.9, 130.7, 130.0, 129.6, 127.6, 125.0, 123.7, 67.3, 59.7, 41.4, 38.7.
HRMS (ESI$^+$): Calcd for $C_{18}H_{15}Cl_2N_2O_2S$ $[M+H]^+$ 393.0226, found 393.0240.
$[\alpha]_D^{25}$= -230.6 (c 0.410 g/100 mL, CH_2Cl_2).

(3*R*,9*bS*)-*N*-(3,4-Dimethoxybenzyl)-5-oxo-2,3,5,9*b*-tetrahydrothiazolo[2,3-*a*]isoindole-3-carboxamide (4aI)

Following the application of the general procedure, this product **4aI** was isolated as white solid, R_f = 0.40, eluent (ethyl acetate / cyclohexane = 6/4), mp = 146-149 °C, 0.200 g scale reaction (10 h), 0.220 g was isolated, 75% yield.

IR (ν_{max} / cm^{-1}): 3297, 1713, 1648, 1519, 1353, 700.
^{1}H NMR (300 MHz, CDCl$_3$): δ_H 7.75 (d, *J* = 7.6 Hz, 1H), 7.62 – 7.57 (m, 1H), 7.52 – 7.46 (m, 2H), 7.24 – 7.20 (bs, 1H), 6.82 – 6.80 (m, 3H), 5.90 (s, 1H), 4.86 (t, *J* = 7.2 Hz, 1H), 4.41 (d, *J* = 5.8 Hz, 2H), 4.01 (dd, *J* = 12.0, 6.9 Hz, 1H), 3.85 (s, 3H), 3.83 (s, 3H), 3.63 (dd, *J* = 12.0, 7.6 Hz, 1H).
^{13}C NMR (75 MHz, CDCl$_3$): δ_C 172.2, 168.8, 149.2, 148.5, 144.3, 133.5, 130.6, 130.4, 129.8, 124.8, 123.6, 120.0, 111.3, 111.0, 67.0, 59.7, 56.0, 56.0, 43.6, 38.8.
HRMS (ESI$^+$): Calcd for $C_{20}H_{20}N_2NaO_4S$ $[M+Na]^+$ 407.1036, found 407.1050.
$[\alpha]_D^{25}$= -230.9 (c 0.280 g/100 mL, CH_2Cl_2).

(3*R*,9*bS*)-5-Oxo-*N*-(3,4,5-trimethoxybenzyl)-2,3,5,9*b*-tetrahydrothiazolo[2,3-*a*]isoindole-3-carboxamide (4aJ)

Following the application of the general procedure, this product **4aJ** was isolated as yellow solid, R_f = 0.34, eluent (ethyl acetate / cyclohexane = 6/4), mp = 162-164 °C, 0.200 g scale reaction (30 h), 0.151 g was isolated, 48% yield.

IR (ν_{max} / cm^{-1}): 3279, 1715, 1649, 1511, 1348, 720.
^{1}H NMR (300 MHz, CDCl$_3$): δ_H 7.80 (d, *J* = 7.4 Hz, 1H), 7.68 – 7.60 (m, 1H), 7.57 – 7.48 (m, 2H), 7.23 (t, *J* = 5.7 Hz, 1H), 6.51 (s, 2H), 5.93 (s, 1H), 4.88 (t, *J* = 7.3 Hz, 1H), 4.43 (d, *J* = 6.0 Hz, 2H), 4.05 (dd, *J* = 12.1, 7.2 Hz, 1H), 3.83 (s, 6H), 3.81 (s, 3H), 3.67 (dd, *J* = 12.1, 7.5 Hz, 1H).
^{13}C NMR (75 MHz, CDCl$_3$): δ_C 172.4, 168.9, 153.6 (x2), 144.3, 137.5, 133.6, 130.7, 129.9, 124.8, 123.7, 104.7 (x2), 67.2, 61.0, 59.8, 56.3 (x2), 44.1, 38.8.
HRMS (ESI$^+$): Calcd for $C_{21}H_{22}N_2NaO_5S$ $[M+H]^+$ 437,1142 found 437.1149.
$[\alpha]D_{25}$= - 256.0 (c 0.255 g/100 mL, CH_2Cl_2).

(3*R*,9*bS*)-*N*-Benzyl-9*b*-methyl-5-oxo-2,3,5,9b-tetrahydrothiazolo[2,3-*a*]isoindole-3-carboxamide (4bA)

This product was already described by us in the following reference: Danton, F.; Othman, M.; Lawson, A.M.; Moncol, J.; Ghinet, A.; Rigo, B.; Daïch, A. *Chem. Eur. J.* **2019**, *25*, 6113–6118.

(3*R*,9b*S*)-*N*-(2-Bromobenzyl)-9b-methyl-5-oxo-2,3,5,9b-tetrahydrothiazolo[2,3-*a*]isoindole-3-carboxamide (4bB)

Following the application of the general procedure, this product **4bB** was isolated as yellow solid, R_f = 0.36, eluent (ethyl acetate / cyclohexane = 3/7), mp = 53-55 °C, 0.200 g scale reaction (14 days), 0.250 g was isolated, 83% yield.
IR (ν_{max} / cm^{-1}): 3306, 1676, 1659, 1552, 1324, 701.
^{1}H NMR (300 MHz, CDCl$_3$): δ_H 7.79 (dt, *J* = 8.5, 1.0 Hz, 1H), 7.63 (td, *J* = 7.5, 1.2 Hz, 1H), 7.50 (ddt, *J* = 13.5, 7.6, 1.0 Hz, 3H), 7.38 (dd, *J* = 7.6, 1.8 Hz, 1H), 7.31 – 7.23 (m, 2H), 7.13 (td, *J* = 7.7, 1.8 Hz, 1H), 4.88 (t, *J* = 8.2 Hz, 1H), 4.61 (dd, *J* = 15.1, 6.2 Hz, 1H), 4.53 (dd, *J* = 15.1, 6.0 Hz, 1H), 4.25 (dd, *J* = 12.4, 7.9 Hz, 1H), 3.76 (dd, *J* = 12.4, 8.6 Hz, 1H), 1.88 (s, 3H).

^{13}C NMR (75 MHz, CDCl$_3$): δ_C 172.0, 169.5, 149.2, 136.9, 133.7, 133.0, 130.1, 129.7, 129.4, 129.0, 127.9, 124.8, 123.8, 122.1, 77.4, 59.5, 44.3, 38.9, 28.0.
HRMS (ESI$^+$): Calcd for $C_{19}H_{18}BrN_2O_2S$ $[M+H]^+$ 417.0267, found 417.0287.
$[\alpha]_D^{25}$= -162.0 (c 0.345 g/100 mL, CH_2Cl_2).

(3*R*,9*bS*)-9*b*-Methyl-5-oxo-*N*-(2-(trifluoromethyl)benzyl)-2,3,5,9*b*-tetrahydrothiazolo[2,3-*a*]-isoindole-3-carboxamide (4bC)
Following the application of the general procedure, this product **4bC** was isolated as orange oil, R_f = 0.50, eluent (ethyl acetate / cyclohexane = 4/6), 0.200 g scale reaction (14 days), 0.157 g was isolated, 51% yield.
IR (ν_{max} / cm^{-1}): 3327, 1699, 1674, 1526, 1311, 702.
^{1}H NMR (300 MHz, CDCl$_3$): δ_H 7.79 (d, *J* = 7.5 Hz, 1H), 7.66 – 7.61 (m, 2H), 7.56 – 7.49 (m, 4H), 7.40 – 7.35 (m, 1H), 7.24 – 7.11 (bs, 1H), 4.87 (t, *J* = 8.3 Hz, 1H), 4.69 (d, *J* = 6.2 Hz, 2H), 4.27 (dd, *J* = 12.4, 8.0 Hz, 1H), 3.77 (dd, *J* = 12.4, 8.6 Hz, 1H), 1.87 (s, 3H).
^{13}C NMR (75 MHz, CDCl$_3$): δ_C 172.1, 169.6, 149.2, 136.2 (d, *J* = 1.7 Hz), 133.8, 132.5 (d, *J* = 0.8 Hz), 130.3, 129.7, 129.0, 128.2 (q, *J* = 30.4 Hz), 127. 9 (q, *J* = 274.0 Hz), 127.8, 126.2 (q, *J* = 5.8 Hz), 124.9, 122.2, 77.4 , 59.6, 40.6 (q, *J* = 2.5 Hz), 38.8, 27.9.
^{19}F NMR (282 MHz, CDCl$_3$): δ_F -59.7.
HRMS (ESI$^+$): Calcd for $C_{20}H_{17}F_3N_2NaO_2S$ $[M+Na]^+$ 429.0855, found 429.0863.
$[\alpha]_D^{25}$= -134.9 (c 0.285 g/100 mL, CH_2Cl_2).

(3*R*,9*bS*)-9*b*-Methyl-5-oxo-*N*-(3-(trifluoromethyl)benzyl)-2,3,5,9*b*-tetrahydrothiazolo[2,3-*a*]-isoindole-3-carboxamide (4bD)
Following the application of the general procedure, this product **4bD** was isolated as yellow solid, R_f = 0.27, eluent (ethyl acetate / cyclohexane = 3/7), mp = 134-136 °C, 0.200 g scale reaction (14 days), 0.170 g was isolated, 55% yield.
IR (ν_{max} / cm^{-1}): 3305, 1677, 1667, 1551, 1324, 701.
^{1}H NMR (300 MHz, CDCl$_3$): δ_H 7.78 – 7.70 (m, 1H), 7.63 (td, *J* = 7.4, 1.2 Hz, 1H), 7.55 – 7.41 (m, 7H), 7.37 (t, *J* = 6.0 Hz, 1H), 4.85 (t, *J* = 8.3 Hz, 1H), 4.66 (dd, *J* = 15.3, 6.6 Hz, 1H), 4.47 (dd, *J* = 15.3, 5.7 Hz, 1H), 4.31 (dd, *J* = 12.5, 8.1 Hz, 1H), 3.77 (dd, *J* = 12.5, 8.6 Hz, 1H), 1.90 (s, 3H).
^{13}C NMR (75 MHz, CDCl$_3$): δ_C 172.1, 169.7, 149.0, 139.2, 133.8, 131.12, 130.9, 129.7, 129.3, 128.9, 124.8, 124.4 (q, *J* = 3.8 Hz), 124.2 (q, *J* = 3.8 Hz), 124.1 (q, *J* = 272.3 Hz), 122.2, 77.4, 59.4, 43.3, 38.7, 27.8.
^{19}F NMR (282 MHz, CDCl$_3$): δ_F -62.6.
HRMS (ESI$^+$): Calcd for $C_{20}H_{17}F_3N_2NaO_2S$ $[M+Na]^+$ 429.0855, found 429.0869.
$[\alpha]_D^{25}$= -156.0 (c 0.220 g/100 mL, CH_2Cl_2).

(3*R*,9*bS*)-*N*-(3-Methoxybenzyl)-9*b*-methyl-5-oxo-2,3,5,9*b*-tetrahydrothiazolo[2,3-*a*]-isoindole-3-carboxamide (4bE)
Following the application of the general procedure, this product **4bE** was isolated as orange oil, R_f = 0.25, eluent (ethyl acetate / cyclohexane = 3/7), 0.200 g scale reaction (30 h), 0.245 g was isolated, 92% yield.
IR (ν_{max} / cm^{-1}): 3336, 1698, 1663, 1524, 1315, 699.
^{1}H NMR (300 MHz, CDCl$_3$): δ_H 7.78 (d, *J* = 7.5 Hz, 1H), 7.64 (td, *J* = 7.5, 1.2 Hz, 1H), 7.54 – 7.42 (m, 2H), 7.23 (t, *J* = 7.7 Hz, 1H), 7.10 (bs, 1H), 6.88 – 6.77 (m, 3H), 4.88 (t, *J* = 8.3 Hz, 1H), 4.54 (dd, *J* = 14.9, 6.2 Hz, 1H), 4.44 (dd, *J* = 14.9, 5.7 Hz, 1H), 4.31 (dd, *J* = 12.4, 7.9 Hz, 1H), 3.85 – 3.72 (m, 4H), 1.91 (s, 3H).
^{13}C NMR (75 MHz, CDCl$_3$): δ_C 172.0, 169.5, 160.0, 149.1, 139.5, 133.7, 129.9, 129.7, 129.0, 124.8, 122.2, 119.9, 113.2, 113.2, 77.4, 59.5, 55.4, 43.9, 38.9, 28.0.

HRMS (ESI$^+$): Calcd for $C_{20}H_{21}N_2O_3S$ $[M+H]^+$ 369.1267, found 369.1267.
$[\alpha]_D^{25}$= -166.9 (c 0.440 g/100 mL, CH_2Cl_2).

(3*R*,9*bS*)-*N*-(4-Methoxybenzyl)-9*b*-methyl-5-oxo-2,3,5,9*b*-tetrahydrothiazolo[2,3-*a*]isoindole-3-carboxamide (4bF)

Following the application of the general procedure, this product **4bF** was isolated as yellow solid, R_f = 0.34, eluent (ethyl acetate / cyclohexane = 4/6), mp = 136-138 °C, 0.300 g scale reaction (25h), 0.343 g was isolated, 86% yield.

IR (ν_{max} / cm^{-1}): 3340, 1698, 1664, 1511, 1315, 700.
^{1}H NMR (300 MHz, $CDCl_3$): δ_H 7.64 – 7.60 (m, 1H), 7.57 (dd, *J* = 7.5, 1.3 Hz, 1H), 7.46 – 7.38 (m, 2H), 7.21 – 7.16 (m, 3H), 6.83 – 6.78 (m, 2H), 4.80 (t, *J* = 8.1 Hz, 1H), 4.49 – 4.33 (m, 2H), 4.22 (dd, *J* = 12.2, 7.6 Hz, 1H), 3.74 (s, 3H), 3.73 – 3.65 (m, 1H), 1.88 (s, 3H).
^{13}C NMR (75 MHz, $CDCl_3$): δ_C 171.7, 169.3, 159.0, 149.0, 133.5, 130.0, 129.5, 129.0, 128.8, 124.5, 122.0, 114.1, 77.2, 59.3, 55.3, 43.3, 39.0, 27.8.
HRMS (ESI$^+$): Calcd for $C_{20}H_{21}N_2O_3S$ $[M+H]^+$ 369.1267, found 369.1277.
$[\alpha]_D^{25}$= -239.4 (c 0.255 g/100 mL, CH_2Cl_2).

(3*R*,9b*S*)-*N*-(2-Methoxybenzyl)-9*b*-methyl-5-oxo-2,3,5,9*b*-tetrahydrothiazolo[2,3-*a*]isoindole-3-carboxamide (4bG)

Following the application of the general procedure, this product **4bG** was isolated as white solid, R_f = 0.30, eluent (ethyl acetate / cyclohexane = 3/7), mp = 47-49 °C, 0.200 g scale reaction (25h), 0.236 g was isolated, 89% yield.

IR (ν_{max} / cm^{-1}): 3330, 1699, 1665, 1523, 1314, 700.
^{1}H NMR (300 MHz, $CDCl_3$): δ_H 7.79 (d, *J* = 7.3 Hz, 1H), 7.63 (td, *J* = 7.5, 1.3 Hz, 1H), 7.54 – 7.46 (m, 2H), 7.28 – 7.24 (m, 2H), 7.22 – 7.19 (bs, 1H), 6.90 (t, *J* = 6.0 Hz, 1H), 6.84 (d, *J* = 8.1 Hz, 1H), 4.88 (t, *J* = 7.8 Hz, 1H), 4.53 (dd, *J* = 14.5, 6.1 Hz, 1H), 4.45 (dd, *J* = 14.5, 5.8 Hz, 1H), 4.25 (dd, *J* = 12.2, 7.5 Hz, 1H), 3.81 (s, 3H), 3.73 (dd, *J* = 12.2, 8.6 Hz, 1H), 1.84 (s, 3H).
^{13}C NMR (75 MHz, $CDCl_3$): δ_C 171.7, 169.2, 157.6, 149.3, 133.6, 129.6, 129.6, 129.0, 129.0, 125.9, 124.7, 122.1, 120.7, 110.4, 77.3, 59.5, 55.4, 39.9, 39.0, 27.9.
HRMS (ESI$^+$): Calcd for $C_{20}H_{21}N_2O_3S$ $[M+H]^+$ 369.1267, found 369.1275.
$[\alpha]_D^{25}$= -204.5 (c 0.410 g/100 mL, CH_2Cl_2).

(3*R*,9*bS*)-*N*-(2,4-Dichlorobenzyl)-9*b*-methyl-5-oxo-2,3,5,9*b*-tetrahydrothiazolo[2,3-*a*]isoindole-3-carboxamide (4bH)

Following the application of the general procedure, this product **4bH** was isolated as yellow solid, R_f = 0.44, eluent (ethyl acetate / cyclohexane = 3/7), mp = 56-58 °C, 0.200 g scale reaction (14 days), 0.258 g was isolated, 88% yield.

IR (ν_{max} / cm^{-1}): 3324, 1693, 1678, 1521, 1314, 701.
^{1}H NMR (300 MHz, $CDCl_3$): δ_H 7.77 (d, *J* = 7.5 Hz, 1H), 7.63 (td, *J* = 7.5, 1.2 Hz, 1H), 7.54 – 7.47 (m, 2H), 7.36 – 7.29 (m, 3H), 7.20 (dd, *J* = 8.3, 2.1 Hz, 1H), 4.84 (t, *J* = 8.3 Hz, 1H), 4.58 (dd, *J* = 15.2, 6.3 Hz, 1H), 4.49 (dd, *J* = 15.2, 6.0 Hz, 1H), 4.23 (dd, *J* = 12.4, 8.0 Hz, 1H), 3.75 (dd, *J* = 12.4, 8.6 Hz, 1H), 1.87 (s, 3H).
^{13}C NMR (75 MHz, $CDCl_3$): δ_C 172.0, 169.6, 149.1, 134.3, 134.2, 134.0, 133.8, 130.8, 129.7, 129.5, 128.9, 127.5, 124.8, 122.1, 77.4, 59.5, 41.4, 38.7, 27.9.
HRMS (ESI$^+$): Calcd for $C_{19}H_{17}Cl_2N_2O_2S$ $[M+H]^+$ 407.0382, found 407.0393.
$[\alpha]_D^{25}$= -208.0 (c 0.305 g/100 mL, CH_2Cl_2).

(3*R*,9b*S*)-*N*-(3,4-Dimethoxybenzyl)-9*b*-methyl-5-oxo-2,3,5,9*b*-tetrahydrothiazolo[2,3-*a*]isoindole-3-carboxamide (4bI)

Following the application of the general procedure, this product **4bI** was isolated as yellow solid, R_f = 0.29, eluent (ethyl acetate / cyclohexane = 5/5), mp = 51-53 °C, 0.200 g scale reaction (50 h), 0.255 g was isolated, 89% yield.

IR (ν_{max} / cm^{-1}): 3336, 1696, 1667.

^{1}H NMR (300 MHz, CDCl$_3$): δ_H 7.68 (d, J = 8.2 Hz, 1H), 7.60 (td, J = 7.5, 1.2 Hz, 1H), 7.48 – 7.43 (m, 2H), 7.14 (t, J = 5.9 Hz, 1H), 6.83 – 6.75 (m, 3H), 4.83 (t, J = 8.2 Hz, 1H), 4.47 (dd, J = 14.6, 6.1 Hz, 1H), 4.37 (dd, J = 14.7, 5.6 Hz, 1H), 4.25 (dd, J = 12.3, 7.8 Hz, 1H), 3.82 (s, 6H), 3.75 (dd, J = 12.3, 8.6 Hz, 1H), 1.88 (s, 3H).

^{13}C NMR (75 MHz, CDCl$_3$): δ_C 171.8, 169.3, 149.1, 149.0, 148.4, 133.6, 130.4, 129.6, 128.8, 124.6, 122.0, 119.9, 111.2, 110.9, 77.3, 59.4, 55.9, 55.9, 43.6, 38.9, 27.8.

HRMS (ESI$^+$): Calcd for $C_{21}H_{22}N_2NaO_4S$ [M+Na]$^+$ 421.1192, found 421.1204.

$[\alpha]_D^{25}$= -154.8 (c 0.295 g/100 mL, CH_2Cl_2)

9*b*-Methyl-5-oxo-*N*-(3,4,5-trimethoxybenzyl)-2,3,5,9*b*-tetrahydrothiazolo[2,3-*a*]isoindole-3-carboxamide (4bJ)

Following the application of the general procedure, this product **4bJ** was isolated as white solid, R_f = 0.28, eluent (ethyl acetate / cyclohexane = 5/5), mp = 66-68 °C, 2.00 g scale reaction (50 h), 0.251 g was isolated, 81% yield.

IR (ν_{max} / cm^{-1}): 3331, 1697, 1669, 1328, 701.

^{1}H NMR (300 MHz, CDCl$_3$): δ_H 7.76 (d, J = 7.5 Hz, 1H), 7.63 (td, J = 7.5, 1.2 Hz, 1H), 7.57 – 7.43 (m, 2H), 7.14 (t, J = 6.2 Hz, 1H), 6.50 (s, 2H), 4.86 (t, J = 8.3 Hz, 1H), 4.48 (dd, J = 14.9, 6.1 Hz, 1H), 4.40 (dd, J = 14.9, 5.7 Hz, 1H), 4.29 (dd, J = 12.4, 8.1 Hz, 1H), 3.97 – 3.68 (m, 10H).

^{13}C NMR (75 MHz, CDCl$_3$): δ_C 172.0, 169.5, 153.6, 149.1, 137.5, 133.8, 133.6, 129.7, 129.0, 124.7, 122.2, 104.7, 77.4, 60.9, 59.6, 56.2, 44.1, 38.9, 28.0.

HRMS (ESI$^+$): Calcd for $C_{22}H_{24}N_2NaO_5S$ [M+Na]$^+$ 451.1298, found 451.1298.

$[\alpha]_D^{25}$= -171.6 (c 0.325 g/100 mL, CH_2Cl_2).

IV. General procedure for the synthesis of *N*-Benzyl-5-oxo-2,3,5,9*b*-tetrahydrothiazolo[2,3-*a*]-isoindole-3-carboxamide 1-oxides (5a,6a) *N*-benzyl-9*b*-methyl-5-oxo-2,3,5,9*b*-tetrahydrothiazolo-[2,3-*a*]isoindole-3-carboxamides 1-oxides (5b)

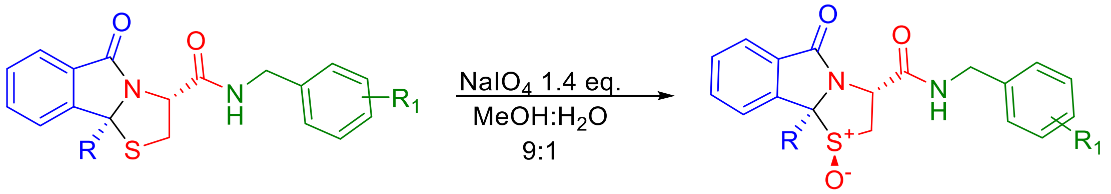

4aA,F,G,J (R=H)
4bA or **4bD-I (R=Me)**

5aA,F,G,J and **6aA,F,G,J (R=H)**
5bA or **5bD-I (R=Me)**

Compound **4aA,F,J** or **4bA** or **4bD-bI** was dissolved in 9 mL of methanol and 1 mL of water, sodium periodate (1.4 eq.) was then added. The reaction mixture was stirred for 16 h at room temperature. After completion of the reaction, the solvent was then evaporated under reduced pressure. The crude compound was then dissolved in 30 mL of dichloromethane and 30 mL of $NaHCO_3$ saturated solution. The organic layer was collected and the aqueous layer was extracted twice with 30 mL of dichloromethane. The combined organic layers were dried on $MgSO_4$. The solvent was evaporated and purification on flash chromatography provided **5aA,F,J/6aA,F,J** or **5bA, 5bD-5bI**.

Important: Sulfoxides compounds **5a/6a** seemed to be instable and decompose in numerous cases.

Entry	R group	R$_1$ group	Product 5a/6a[3]	Entry	R group	R$_1$ group	Product 5b[5]
1	H	H	**5aA/6aA**	11	Me	H	**5bA**
2	H	-[1]	**5aB/6aB**	12	Me	-[4]	**5bB**
3	H	-[1]	**5aC/6aC**	13	Me	-[4]	**5bC**
4	H	*m*-CF_3[2]	**5aD/6aD***	14	Me	*m*-CF_3	**5bD**
5	H	-[1]	**5aE/6aE**	15	Me	*m*-OMe	**5bE**
6	H	*p*-OMe	**5aF/6aF**	16	Me	*p*-OMe	**5bF**
7	H	*o*-OMe	**5aG/6aG**	17	Me	*o*-OMe	**5bG**
8	H	-[1]	**5aH/6aH**	18	Me	*o,p*-2Cl	**5bH**
9	H	-[1]	**5aI/6aI**	19	Me	*o,p*-2OMe	**5bI**
10	H	*m,m',p*-3OMe	**5aJ/6aJ**	20	Me	-[4]	**5bJ**

[1] Decomposition in the solution. [2] Decomposition out of the solution. [3] 2 Diastereoisomers were obtained. [4] Not realised. [5] Only one diastereoisomer was obtained.

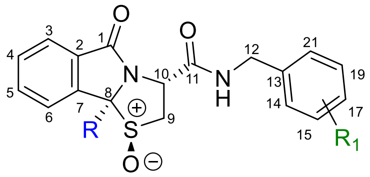

R=H (5a, Maj)
R=Me (5b, 100%)

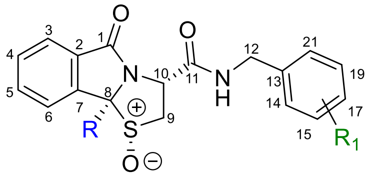

R=H (6a, Min)
R=Me (6b, 0%)

(9*bS*)-*N*-Benzyl-5-oxo-2,3,5,9*b*-tetrahydrothiazolo[2,3-*a*]isoindole-3-carboxamide 1-oxide

Major diastereoisomer (5aA)

White solid, R_f = 0.53, eluent (dichloromethane / methanol = 9/1), mp = 215-217 °C, 200 mg scale reaction, 58 mg was isolated, 28% yield.

IR (ν_{max} / cm^{-1}): 3331, 1707, 1659, 1555, 1354, 1046, 703.

^{1}H NMR (300 MHz, $CDCl_3$): δ_H 7.76 (d, *J* = 7.6 Hz, 1H, H-3), 7.71 – 7.58 (m, 2H), 7.62 – 7.49 (m, 2H), 7.34 – 7.21 (m, 5H), 5.74 (s, 1H, H-8), 4.49 (dd, *J* = 14.9, 6.0 Hz, 1H, H-12), 4.40 (dd, *J* = 14.9, 5.7 Hz, 1H, H-12), 3.78 (dd, *J* = 14.5, 7.2 Hz, 1H, H-9), 3.69 (dd, *J* = 14.5, 9.6 Hz, 1H, H-9).

^{13}C NMR (75 MHz, $CDCl_3$): δ_C 172.5 ($Cq_{C=O}$, C-1), 168.3 ($Cq_{C=O}$, C-11), 137.6 (Cq, C-7), 135.6 (Cq, C-2), 133.6 (CH, C-5), 133.3 (Cq, C-2), 130.8 (CH, C-4), 128.9 (2x CH, C-15, C-17), 127.7 (2x CH, C-14, C-18), 127.7 (CH, C-16), 124.6 (CH, C-3), 124.2 (CH, C-6), 83.9 (CH, C-8), 56.9 (CH_2, C-9), 56.0 (CH, C-10), 44.0 (CH_2, C-12).

HRMS (ESI^+): Calcd for $C_{18}H_{17}N_2O_3S$ $[M+H]^+$ 341.0954, found 341. 0970.

Minor diastereoisomer (6aA)

Brown solid, R_f = 0.55, eluent (dichloromethane / methanol = 9/1), mp = 82-84 °C, 200 mg scale reaction, 12 mg was isolated, 6% yield.

IR (ν_{max} / cm^{-1}): 3299, 1705, 1664, 1533, 1355, 1037, 721.

^{1}H NMR (300 MHz, $CDCl_3$): δ_H 7.82 (d, *J* = 8.5 Hz, 2H, H-3, H-6), 7.72 (td, *J* = 7.4, 1.3 Hz, 1H, H-5), 7.66 – 7.58 (m, 1H, H-4), 7.38 – 7.27 (m, 5H, Har), 7.22 – 7.15 (bs, 1H, NH), 5.59 (s, 1H, H-8), 4.96 (t, *J* = 7.8 Hz, 1H, H-10), 4.50 (d, *J* = 5.8 Hz, 2H, H-12), 4.05 (dd, *J* = 13.2, 7.8 Hz, 1H, H-9), 3.95 (dd, *J* = 13.2, 7.9 Hz, 1H, H-9).

^{13}C NMR (75 MHz, $CDCl_3$): δ_C 170.7 ($Cq_{C=O}$, C-1), 167.4 ($Cq_{C=O}$, C-11), 138.9 (Cq, C-7), 137.5 (Cq, C-13), 134.2 (CH, C-5), 130.9 (CH, C-4), 130.2 (Cq, C-2), 129.0 (2x CH, C-17, 15), 127.9 (2x CH, C-15, C-17), 125.0 (CH, C-3), 124.2 (CH, C-6), 84.1 (CH, C-10), 57.4 (CH_2, C-9), 54.7 (CH, C-10), 44.2 (CH, C-12).

HRMS (ESI$^+$): Calcd for $C_{18}H_{17}N_2O_3S$ $[M+H]^+$ 341.0954, found 341.0967.

(9b*S*)-*N*-(4-Methoxybenzyl)-5-oxo-2,3,5,9*b*-tetrahydrothiazolo[2,3-*a*]isoindole-3-carboxamide 1-oxide

Major diastereoisomer (5aF)
White solid, R_f = 0.58, eluent (dichloromethane / methanol = 9/1), mp = 218-220 °C, 200 mg scale reaction, 63 mg was isolated, 30% yield.

IR (ν_{max} / cm^{-1}): 3293, 1705, 1651, 1512, 1350, 1039, 710.
^{1}H NMR (300 MHz, $CDCl_3$): δ_H 7.78 (d, *J* = 7.5 Hz, 1H, H-3), 7.67 – 7.48 (m, 3H, H-4, H-5, H-6), 7.32 – 7.24 (bt, 1H, NH), 7.15 (d, *J* = 8.5 Hz, 2H, H-14, H-19), 6.79 (d, *J* = 8.6 Hz, 2H, H-15, H-18), 5.65 (s, 1H, H-7), 5.07 (dd, *J* = 9.5, 7.5 Hz, 1H, H-10), 4.39 (dd, *J* = 14.6, 5.9 Hz, 1H, H-12), 4.32 (dd, *J* = 14.6, 5.7 Hz, 1H, H-12), 3.87 – 3.61 (m, 5H, H-17, H-9).
^{13}C NMR (75 MHz, $CDCl_3$): δ_C 172.7 ($Cq_{C=O}$, C-1), 168.0 ($Cq_{C=O}$, C-11), 159.2 (Cq, C-17), 135.7 (CH, C-5), 133.6 (Cq, C-7), 133.3 (Cq, C-2), 130.9 (CH, C-4), 129.6 (Cq, C-13), 129.2 (2x CH, C-14, C-19), 124.8 (CH, C-3), 124.3 (CH, C-6), 114.3 (2x CH, C-15, C-18), 83.8 (CH, C-8), 56.7 (CH_2, C-9), 56.1 (CH, C-10), 55.4 (CH3, C-17), 43.7 (CH_2, C-12).

Minor diastereoisomer (6aF)

Brown solid, R_f = 0.50, eluent (dichloromethane / methanol = 9/1), mp = 83-85 °C, 200 mg scale reaction, 10 mg was isolated, 8% yield.

IR (ν_{max} / cm^{-1}): 3300, 1706, 1666, 1512, 1354, 1030.
^{1}H NMR (300 MHz, $CDCl_3$): δ_H 7.82 (d, *J* = 7.9 Hz, 2H, H-3, H-6), 7.72 (td, *J* = 7.5, 1.1 Hz, 1H, H-, H-5), 7.67 – 7.54 (m, 1H, H-4), 7.22 (d, *J* = 8.6 Hz, 2H, H-14, H-19), 7.09 – 7.00 (bt, 1H, NH), 6.86 (d, *J* = 8.6 Hz, 2H, H-15, H-18), 5.58 (s, 1H, H-8), 4.94 (t, *J* = 7.9 Hz, 1H, H-10), 4.44 (d, *J* = 5.7 Hz, 2H, H-12), 4.06 (dd, *J* = 13.2, 7.9 Hz, 1H, H-9), 3.95 (dd, *J* = 13.2, 7.9 Hz, 1H, H-9), 3.78 (s, 3H, H-17).
^{13}C NMR (75 MHz, $CDCl_3$): δ_C 170.7 ($Cq_{C=O}$, C-1), 167.3 ($Cq_{C=O}$, C-11), 159.3 (Cq, C-16), 138.9 (Cq, C-7), 134.2 (CH, C-5), 130.9 (CH, C-4), 130.2 (Cq, C-2), 129.6 (Cq, C-13), 129.3 (2x CH, C-14, C-19), 125.0 (CH, C-3), 124.2 (CH, C-6), 114.4 (CH, C-15, C-18), 84.1 (CH, C-8), 57.5 (CH2, C-9), 55.4 (CH_3, C-17), 54.8 (CH, C-10), 43.7 (CH_2, C-12).
HRMS (ESI$^+$): Calcd for $C_{19}H_{19}N_2O_4S$ $[M+H]^+$ 371.1060, found 371.1073.

(9*bS*)-*N*-(2-Methoxybenzyl)-5-oxo-2,3,5,9*b*-tetrahydrothiazolo[2,3-*a*]isoindole-3-carboxamide 1-oxide

Major diastereoisomer (5aG)
White solid, R_f = 0.67, eluent (dichloromethane / methanol = 9/1), mp = 219-221 °C, 200 mg scale reaction, 64 mg was isolated, 30% yield.

IR (ν_{max} / cm^{-1}): 3330, 1705, 1664, 1533, 1350, 1046, 699.
^{1}H NMR (300 MHz, $CDCl_3$): δ_H 7.86 (d, *J* = 7.6 Hz, 1H, H-3), 7.72 – 7.50 (m, 4H), 7.30 – 7.22 (m, 2H), 6.95 – 6.81 (m, 2H, H-14, H-16), 5.71 (s, 1H), 5.12 (t, *J* = 8.4 Hz, 1H, H-10), 4.58 – 4.39 (m, 2H, H-12), 3.86 (s, 3H, H-19), 3.77 (d, *J* = 8.4 Hz, 2H, H-9).
^{13}C NMR (75 MHz, $CDCl_3$): δ_C 172.3 ($Cq_{C=O}$, C-1), 167.8 ($Cq_{C=O}$, C-1), 157.7 (Cq, C-18), 135.6 (CH, C-5), 133.5 (Cq, C-7), 133.4 (Cq, C-2), 130.8 (CH, C-4), 129.5 (CH, C-14)), 129.2 (CH, C-16), 125.6 (Cq, C-13), 124.6 (CH, C-3), 124.2 (CH, C-6), 120.7 (CH, C-15), 110.5 (CH, C-17), 83.8 (CH, C-8), 56.8 (CH_2, C-9), 56.0 (CH_3, C-19), 55.4 (CH, C-10), 40.2 (CH_2, C-12).
HRMS (ESI$^+$): Calcd for $C_{19}H_{19}N_2O_4S$ $[M+H]^+$ 371.1060, found 371.1064

Minor diastereoisomer (6aG)

Brown solid, R_f = 0.73, eluent (dichloromethane / methanol = 9/1), mp = 74-76 °C, 200 mg scale reaction, 10 mg was isolated, 5% yield.

IR (ν_{max} / cm^{-1}): 3303, 1703, 1670, 1534, 1357, 1045, 722.

^{1}H NMR (300 MHz, $CDCl_3$): δ_H 7.83 (dd, *J* = 7.5, 5.9 Hz, 2H), 7.78 – 7.66 (m, 1H), 7.69 – 7.56 (m, 1H), 7.33 – 7.20 (m, 3H, NH, H-15, H-17), 6.97 – 6.82 (m, 2H, H-14, H-16), 5.57 (s, 1H, H-8), 4.88 (t, *J* = 8.0 Hz, 1H, H-10), 4.60 – 4.40 (m, 2H, H-12), 4.12 – 3.91 (m, 1H, H-9), 3.90 – 3.71 (m, 4H, H-9, H-19).

^{13}C NMR (75 MHz, $CDCl_3$): δ_C 170.3 ($Cq_{C=O}$, C-1), 167.1 ($Cq_{C=O}$, C-11), 157.7 (Cq, C-18), 138.9 (Cq, C-7), 134.0 (CH, C-5), 130.9 (CH, C-4), 130.3 (Cq, C-2), 129.8 (CH, C-14), 129.3 (CH, C-16), 125.5 (Cq, C-13), 124.9 (CH, C-3), 124.2 (CH, C-6), 120.8 (CH, C-15), 110.5 (CH, C-17), 83.4 (CH, C-8), 57.5 (CH2, C-9), 55.4 (CH_3, C-19), 54.3 (CH, C-10), 40.3 (CH_2, C-12).

HRMS (ESI$^+$): Calcd for $C_{19}H_{19}N_2O_4S$ $[M+H]^+$ 371.1060, found 371.1075.

(9*bS*)-5-Oxo-*N*-(3,4,5-trimethoxybenzyl)-2,3,5,9*b*-tetrahydrothiazolo[2,3-*a*]isoindole-3-carboxamide 1-oxide

Major diastereoisomer (5aJ)

White solid, R_f = 0.60, eluent (dichloromethane / methanol = 9/1), mp = 221-223 °C, 200 mg scale reaction, 3 mg was isolated, 1% yield.

IR (ν_{max} / cm^{-1}): 3339, 1739, 1697, 1596, 1354, 1019, 699.

^{1}H NMR (300 MHz, $CDCl_3$): δ_H 7.79 (d, *J* = 7.5 Hz, 1H), 7.75 – 7.49 (m, 4H), 6.50 (s, 2H, H-14, H-21), 5.75 (s, 1H, H-8), 5.16 (dd, *J* = 9.7, 7.2 Hz, 1H, H-12), 4.52 – 4.32 (m, 2H, H-12), 3.95 – 3.66 (m, 11H, H-16, H-18, H-20, H-9).

^{13}C NMR (75 MHz, $CDCl_3$): δ_C 172.6 ($Cq_{C=O}$, C-1), 168.1 ($Cq_{C=O}$, C-11), 153.5 (2x Cq, C-15, C-19), 137.4 (Cq, C-13), 135.6 (Cq, C-7), 133.7 (CH, C-5), 133.2 (Cq, C-17), 133.2 (Cq, C-2), 130.9 (CH, C-4), 124.6 (CH, C-3), 124.3 (CH, C-6), 104.6 (2x CH, C-14, C-21), 83.9 (CH, C-8), 61.0 (CH_3, C-18), 56.8 (CH_2, C-9), 56.2 (CH, C-10), 56.0 (CH_3, C-16, C-20), 44.2 (CH_2, C-12).

HRMS (ESI$^+$): Calcd for $C_{21}H_{23}N_2O_6S$ $[M+H]^+$ 431.1271, found 431.1289.

Minor diastereoisomer (6aJ)

Brown solid, R_f = 0.48, eluent (dichloromethane / methanol = 9/1), mp = 69-71 °C, 200 mg scale reaction, 48 mg was isolated, 23% yield.

IR (ν_{max} / cm^{-1}): 3323, 1712, 1705, 1593, 1350, 1048, 716.

^{1}H NMR (300 MHz, $CDCl_3$): δ_H 7.83 (d, *J* = 7.8 Hz, 2H, H-3, H-6), 7.73 (t, *J* = 7.5 Hz, 1H, H-5), 7.69 – 7.59 (m, 1H, H-4), 7.18 – 7.04 (bs, 1H, NH), 6.51 (s, 2H, H-14, H-21), 5.59 (s, 1H, H-10), 4.99 (t, *J* = 7.7 Hz, 1H, H-10), 4.58 – 4.34 (m, 2H, H-12), 4.07 (dd, *J* = 13.2, 7.7 Hz, 1H, H-9), 3.93 (dd, *J* = 13.2, 7.9 Hz, 1H, H-6), 3.84 (s, 6H, H-16, H-20), 3.81 (s, 3H, H-18).

^{13}C NMR (75 MHz, $CDCl_3$): δ_C 170.7 ($Cq_{C=O}$, C-1), 167.4 ($Cq_{C=O}$, C-11), 153.6 (2xCq, C-15, C-19), 138.9 (Cq, C-7), 137.5 (Cq, C-13) 134.3 (CH, C-5), 133.2 (Cq, C-17), 131.0 (CH, C-5), 130.1 (Cq, C-2), 125.0 (CH, C-6), 124.3 (CH, C-3), 104.8 (2x CH, C-14, C-21), 84.4 (CH, C-8), 61.0 (CH_3, C-18), 57.4 (CH_2, C-9), 56.3 (CH, C-10), 55.0 (CH3, C-16, C-20), 44.4 (CH_2, C-12).

Chromatogram of sulfoxides mixture of 5aD and 6aD

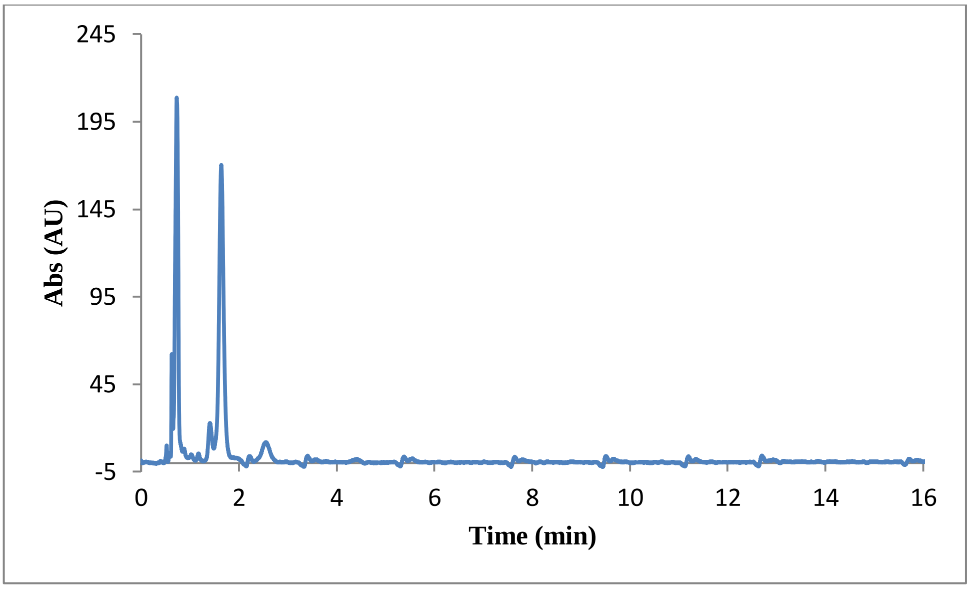

The purification was performed by using *Supercritical fluid chromatography (SFC)* (Prof. E. Lipka).
Experimental Conditions: Chiralpak AD-H 30% MeOH, 150 bar outlet 40C 5 mL/min at λ = 210 nm.
The 2 diastereoisomers at t_1 = 0.72 min (**5aD-Maj**) and t_2 = 1.62 min (**6aD-Min**)

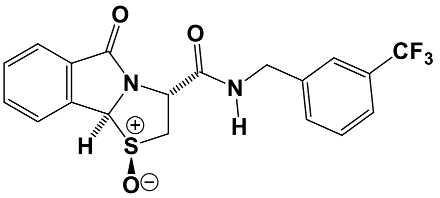

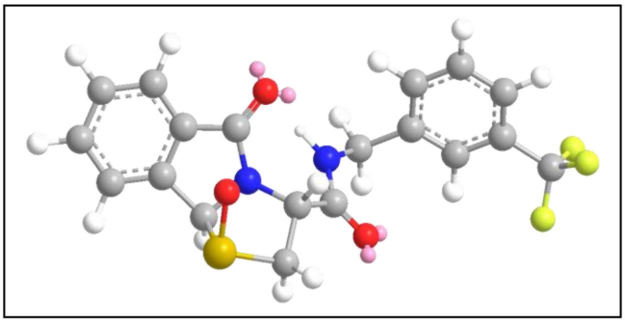

Product **5aD-Maj**
MM2 Calculations from ChemDraw3D software
Total Energy = -61.9275 kcal/mol

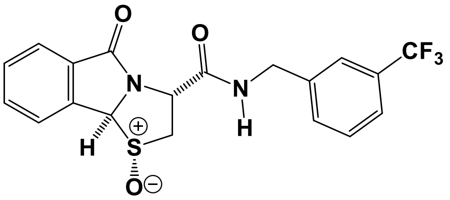

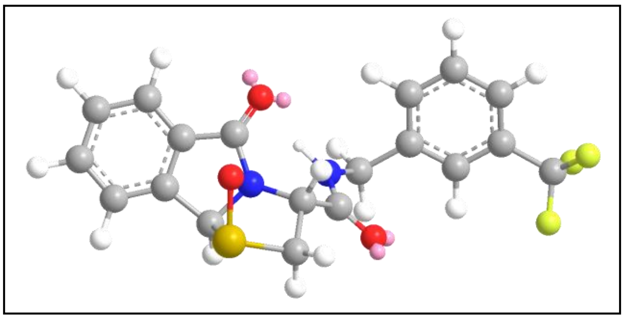

Product **6aD-Min**
MM2 Calculations from ChemDraw3D software
Total Energy = -61.2950 kcal/mol

Important comments:
The observed diastereoselectivity during the sulfoxidation reaction seems to proceed *via* the Felkin-Ahn type addition in with the oxidizing agent attack the sulphur atom from the opposite side of both the amide function and the angular proton to furnish the sulfoxide **5aD** as Major diastereoisomer. This is in an agreement with a rapid calculation (using Chem3D of ChemDraw Pro-17.0.0.206(121) software) of the stabilization energy of the sulfoxide **5aD** giving a total energy of -61.9275 kcal/mol. Of interest, numerous attempts using same calculation protocol starting from the sulfoxide **6aD** furnished in all cases a total energy of -61.2950 kcal/mol, which correspond in fact to the one obtained for the above diastereoisomer **5aD** being obtained by isomerization of **6aD**.
Ultimately, from these preliminary results, the more stable species seems to be the diastereoisomer **5aD** obtained as a major isomer. In addition, the bulky amide group, compared to the angular group H (or Me) seems to control the stereochemistry outcome of the sulfoxidation reaction.

(3*R*,9*bS*)-*N*-Benzyl-9*b*-methyl-5-oxo-2,3,5,9*b*-tetrahydrothiazolo[2,3-*a*]isoindole-3-carboxamide 1-oxide (5bA)

Following the application of the general procedure, this product **5bA** was isolated as yellow solid, R_f = 0.63, eluent (ethyl acetate / cyclohexane = 6/4), mp = 207-209 °C, 175 mg scale reaction (in 9 mL of methanol + 1 mL of water), 95 mg was isolated, 52% yield.

IR (ν_{max} / cm^{-1}): 3305, 1706, 1670, 1537, 1335, 1045, 692.
^{1}H NMR (300 MHz, $CDCl_3$): δ_H 7.73 (d, *J* = 7.3 Hz, 1H), 7.69 – 7.64 (m, 1H), 7.56 – 7.50 (m, 2H), 7.33 – 7.23 (m, 5H), 5.19 (t, *J* = 8.4 Hz, 1H), 4.47 (d, *J* = 5.9 Hz, 2H), 3.93 (dd, *J* = 14.9, 8.8 Hz, 1H), 3.67 (dd, *J* = 14.9, 8.2 Hz, 1H), 1.78 (s, 3H).
^{13}C NMR (75 MHz, $CDCl_3$): δ_C 172.3, 168.6, 140.9, 137.7, 133.7, 132.1, 130.7, 128.8, 127.7, 127.6, 124.3, 122.9, 91.2, 56.3, 55.8, 44.1, 19.1.
HRMS (ESI^+): Calcd for $C_{19}H_{19}N_2O_3S$ $[M+H]^+$ 355.1111, found 355.1108.
$[\alpha]_D^{25}$= -518.2 (c 0.240 g/100 mL, CH_2Cl_2).

(3*R*,9*bS*)-9*b*-Methyl-5-oxo-*N*-(3-(trifluoromethyl)benzyl)-2,3,5,9*b*-tetrahydrothiazolo[2,3-*a*]-isoindole-3-carboxamide 1-oxide (5bD)

Following the application of the general procedure, this product **5bD** was isolated as yellow solid, R_f = 0.28, eluent (ethyl acetate / cyclohexane = 4/1), mp = 194-196 °C, 185 mg scale reaction (in 9 mL of methanol + 1 mL of water), 90 mg was isolated, 47% yield.

IR (ν_{max} / cm^{-1}): 3334, 1718, 1683, 1547, 1326, 1058, 700.
^{1}H NMR (300 MHz, $CDCl_3$): δ_H 7.71 – 7.64 (m, 2H), 7.57 – 7.48 (m, 5H), 7.46 – 7.38 (m, 2H), 5.20 (t, *J* = 8.5 Hz, 1H), 4.57 (dd, *J* = 15.3, 6.4 Hz, 1H), 4.44 (dd, *J* = 15.3, 5.9 Hz, 1H), 3.92 (dd, *J* = 14.9, 8.9 Hz, 1H), 3.68 (dd, *J* = 14.9, 8.2 Hz, 1H), 1.78 (s, 3H).
^{13}C NMR (75 MHz, $CDCl_3$): δ_C 172.5, 168.9, 140.9, 139.0, 133.8, 132.0, 131.0 (q, *J* = 32.2 Hz), 130.9 (d, *J* = 1.5 Hz), 130.7, 129.3, 124.4 (q, *J* = 3.8 Hz), 124.6 (q, *J* = 273.1 Hz), 124.2, 124.1 (q, *J* = 5.0 Hz), 122.9, 91.2, 56.3, 55.7, 43.4, 19.1.
HRMS (ESI^+): Calcd for $C_{20}H_{18}F_3N_2O_3S$ $[M+H]^+$ 423.0985, found 423.0994.
$[\alpha]_D^{25}$= -291.6 (c 0.295 g/100 mL, CH_2Cl_2).

(3*R*,9*bS*)-*N*-(3-Methoxybenzyl)-9*b*-methyl-5-oxo-2,3,5,9*b*-tetrahydrothiazolo[2,3-*a*]isoindole-3-carboxamide 1-oxide (5bE)

Following the application of the general procedure, this product **5bE** was isolated as white solid, R_f = 0.20, eluent (ethyl acetate / cyclohexane = 8/2), mp = 68-70 °C, 200 mg scale reaction (in 9 mL of methanol + 1 mL of water), 167 mg was isolated, 80% yield.

IR (ν_{max} / cm^{-1}): 3337, 1719, 1668, 1557, 1338, 1061, 726.
^{1}H NMR (300 MHz, $CDCl_3$): δ_H 7.69 – 7.62 (m, 2H), 7.55 – 7.47 (m, 2H), 7.40 – 7.39 (bs, 1H), 7.23 – 7.18 (m, 1H), 6.84 – 6.77 (m, 3H), 5.17 (t, *J* = 8.5 Hz, 1H), 4.42 (d, *J* = 5.9 Hz, 2H), 3.95 – 3.81 (m, 1H), 3.74 (s, 3H), 3.65 (dd, *J* = 14.9, 8.2 Hz, 1H), 1.78 (s, 3H).
^{13}C NMR (75 MHz, $CDCl_3$): δ_C 172.2 5, 168.7, 159.8, 140.8, 139.3, 133.6, 132.1, 130.6, 129.8, 124.1, 122.8, 119.7, 113.1, 113.0, 91.2, 56.2, 55.8, 55.2, 43.8, 19.0.
HRMS (ESI^+): Calcd for $C_{20}H_{21}N_2O_4S$ $[M+H]^+$ 385.1217, found 385.1218.
$[\alpha]_D^{25}$= -260.1 (c 0.415 g/100 mL, CH_2Cl_2).

(3*R*,9*bS*)-*N*-(4-Methoxybenzyl)-9*b*-methyl-5-oxo-2,3,5,9*b*-tetrahydrothiazolo[2,3-*a*]isoindole-3-carboxamide 1-oxide (5bF)

Following the application of the general procedure, this product **5bF** was isolated as yellow solid, R_f = 0.21, eluent (ethyl acetate / cyclohexane = 9/1), mp = 72-74 °C, 170 mg scale reaction (in 9 mL of methanol + 1 mL of water), 90 mg was isolated, 51% yield.

IR (ν_{max} / cm^{-1}): 3290, 1713, 1652, 1560, 1325, 1029, 695.
^{1}H NMR (300 MHz, CDCl$_3$): δ_H 7.69 – 7.62 (m, 2H), 7.55 – 7.43 (m, 2H), 7.32 (t, *J* = 5.7 Hz, 1H), 7.16 (d, *J* = 8.5 Hz, 2H), 6.82 (d, *J* = 8.5 Hz, 2H), 5.15 (t, *J* = 8.4 Hz, 1H), 4.37 (d, *J* = 6.0 Hz, 2H), 3.88 (dd, *J* = 14.8, 8.7 Hz, 1H), 3.76 (s, 3H), 3.66 (dd, *J* = 14.9, 8.2 Hz, 1H), 1.77 (s, 3H).
^{13}C NMR (75 MHz, CDCl$_3$): δ_C 172.2, 168.5, 159.0, 140.8, 133.6, 132.1, 130.6, 129.8, 129.0, 124.2, 122.8, 114.1, 91.2, 56.2, 55.9, 55.3, 43.5, 19.0.
HRMS (ESI$^+$): Calcd for $C_{20}H_{21}N_2O_4S$ [M+Na]$^+$ 385.1217, found 385.1239.
$[\alpha]_D^{25}$= -296.2 (c 0.270 g/100 mL, CH_2Cl_2).

(3*R*,9*bS*)-*N*-(2-Methoxybenzyl)-9*b*-methyl-5-oxo-2,3,5,9*b*-tetrahydrothiazolo[2,3-*a*]isoindole-3-carboxamide 1-oxide (5bG)

Following the application of the general procedure, this product **5bG** was isolated as white solid, R_f = 0.25, eluent (ethyl acetate / cyclohexane = 9/1), mp = 199-201 °C, 163 mg scale reaction (in 9 mL of methanol + 1 mL of water), 140 mg was isolated, 82% yield.

IR (ν_{max} / cm^{-1}): 3304, 1706, 1670, 1537, 1336, 1046, 727.
^{1}H NMR (300 MHz, CDCl$_3$): δ_H 7.78 (dt, *J* = 7.7, 1.6 Hz, 1H), 7.65 (tt, *J* = 7.1, 1.3 Hz, 1H), 7.57 – 7.52 (m, 2H), 7.34 (t, *J* = 5.8 Hz, 1H), 7.26 – 7.24 (m, 2H), 6.91 – 6.83 (m, 2H), 5.16 (t, *J* = 8.2 Hz, 1H), 4.46 (d, *J* = 5.9 Hz, 2H), 3.92 (ddd, *J* = 14.9, 8.7, 2.2 Hz, 1H), 3.82 (s, 3H), 3.62 (ddd, *J* = 15.0, 8.3, 1.3 Hz, 1H), 1.72 (s, 3H).
^{13}C NMR (75 MHz, CDCl$_3$): δ_C 171.9, 168.3, 157.5, 140.8, 133.5, 132.1, 130.5, 129.3, 129.0, 125.6, 124.1, 122.8, 120.6, 110.3, 91.0, 56.1, 55.8, 55.3, 40.0, 18.9.
HRMS (ESI$^+$): Calcd for $C_{20}H_{21}N_2O_4S$ [M+H]$^+$ 386.1217, found 385.1233.
$[\alpha]_D^{25}$= -307.9 (c 0.290 g/100 mL, CH_2Cl_2).

(3*R*,9*bS*)-*N*-(2,4-Dichlorobenzyl)-9*b*-methyl-5-oxo-2,3,5,9*b*-tetrahydrothiazolo[2,3-*a*]isoindole-3-carboxamide 1-oxide (5bH)

Following the application of the general procedure, this product **5bH** was isolated as colorless oil, R_f = 0.32, eluent (ethyl acetate / cyclohexane = 9/1), 200 mg scale reaction (in 9 mL of methanol + 1 mL of water), 152 mg was isolated, 73% yield.

IR (ν_{max} / cm^{-1}): 3333, 1717, 1668, 1529, 1337, 1045, 726.
^{1}H NMR (300 MHz, CDCl$_3$): δ_H 7.78 (d, *J* = 8.2 Hz, 1H), 7.69 (t, *J* = 7.5 Hz, 1H), 7.59 – 7.54 (m, 2H), 7.41 – 7.37 (m, 1H), 7.27 (d, *J* = 7.3 Hz, 1H), 7.23 (t, *J* = 6.21 Hz, 1H), 7.19 (dd, *J* = 8.2, 2.1 Hz, 1H), 5.20 (t, *J* = 8.5 Hz, 1H), 4.73 – 4.41 (m, 2H), 3.90 (dd, *J* = 14.9, 8.9 Hz, 1H), 3.69 (dd, *J* = 14.9, 8.1 Hz, 1H), 1.77 (s, 3H).
^{13}C NMR (75 MHz, CDCl$_3$): δ_C 172.5, 168.8, 141.0, 134.3, 134.2, 133.8, 133.8, 131.2, 130.7, 130.6, 129.5, 127.5, 124.4, 122.9, 91.2, 56.3, 55.6, 41.6, 19.3.
HRMS (ESI$^+$): Calcd for $C_{19}H_{17}Cl_2N_2O_3S$ [M+H]$^+$ 423.0331, found 423.0343.
$[\alpha]_D^{25}$= -222.2 (c 0.230 g/100 mL, CH_2Cl_2).

(3*R*,9*bS*)-*N*-(3,4-Dimethoxybenzyl)-9*b*-methyl-5-oxo-2,3,5,9*b*-tetrahydrothiazolo[2,3-*a*]isoindole-3-carboxamide 1-oxide (5bI)

Following the application of the general procedure, this product **5bI** was isolated as white solid, R_f = 0.13, eluent (ethyl acetate / cyclohexane = 9/1), mp = 159-161 °C, 195 mg scale reaction (in 10 mL of methanol), 141 mg was isolated, 68% yield.

IR (ν_{max} / cm^{-1}): 3330, 1692, 1671, 1515, 1336, 1023, 693.
^{1}H NMR (300 MHz, CDCl$_3$): δ_H 7.70 – 7.62 (m, 2H), 7.55 – 7.49 (m, 2H), 7.29 (t, *J* = 6.0 Hz, 1H), 6.82 – 6.76 (m, 3H), 5.17 (t, *J* = 8.5 Hz, 1H), 4.40 (d, *J* = 5.9 Hz, 2H), 3.91 (dd, *J* = 14.9, 8.9 Hz, 1H), 3.83 (s, 3H), 3.80 (s, 3H), 3.68 (dd, *J* = 14.9, 8.1 Hz, 1H), 1.78 (s, 3H).

^{13}C NMR (75 MHz, $CDCl_3$): δ_C 172.3, 168.5, 149.1, 148.5, 140.8, 133.7, 132.1, 130.6, 130.2, 124.2, 122.8, 119.9, 111.3, 110.9, 91.2, 56.3, 56.0, 55.9, 55.8, 43.8, 19.1.
$[\alpha]_D^{25}$= -245.5 (c 0.280 g/100 mL, CH_2Cl_2).

V. General procedure for the synthesis of *N*-benzyl-5-oxo-2,3,5,9b-tetrahydrothiazolo[2,3-a]-isoindole-3-carboxamide 1,1-dioxides

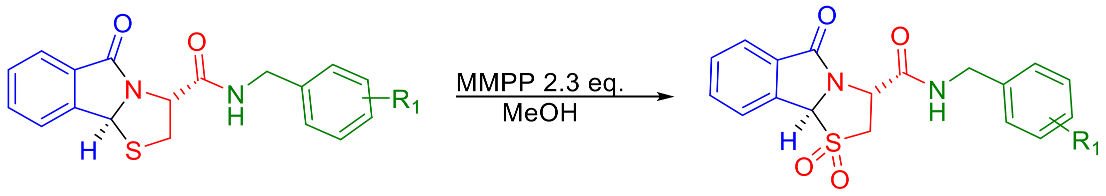

4aA, **4aD**-a**G**, **4aI** **7aA**, **7aD**-a**G**, **7aI**

To the substrate **4aA, 4aD-4aG** or **4aI** was dissolved in 20 mL of methanol, MMPP (2.3 eq.) was added. The reaction mixture was stirred for 16 h at room temperature and the solvent was then evaporated under reduced pressure. The crude compound was then dissolved in 30 mL of dichloromethane and 30 mL of a $NaHCO_3$ saturated solution. The organic layer was separated, collected and the resulting aqueous layer was extracted twice with 30 mL of dichloromethane. The combined organic layers were dried over $MgSO_4$. The solvent was evaporated *in vacuo* and purification on flash chromatography provided compound **7aA, 7aD-7aG** or **7aI.**

Entry	R group	R_1 group	Product 7a
1	H	H	**7aA**
2	H	-[1]	**7aB**
3	H	-[1]	**7aC**
4	H	*m*-CF_3	**7aD**
5	H	*m*-OMe	**7aE**
6	H	*p*-OMe	**7aF**
7	H	*o*-OMe	**7aG**
8	H	-[1]	**7aH**
9	H	*m,p*-2OMe	**7aI**
10	H	-[1]	**7aJ**

[1] Not realised.

(3*R*,9b*S*)-*N*-Benzyl-5-oxo-2,3,5,9b-tetrahydrothiazolo[2,3-*a*]isoindole-3-carboxamide 1,1-dioxide (7aA)
Following the application of the general procedure, this product **7aA** was isolated as white solid, R_f = 0.26, eluent (ethyl acetate / cyclohexane = 4/6), mp = 144-146 °C, 155 mg scale reaction (in 10 mL of methanol), 118 mg was isolated, 69% yield.

IR (ν_{max} / cm^{-1}): 3338, 1710, 1674, 1533, 1327, 1140, 698.
^{1}H NMR (300 MHz, $CDCl_3$): δ_H 7.79 (d, *J* = 7.4 Hz, 1H), 7.71 – 7.58 (m, 3H), 7.37 – 7.22 (m, 6H), 5.66 (s, 1H), 5.01 (t, *J* = 8.6 Hz, 1H), 4.50 (dd, *J* = 14.9, 6.0 Hz, 1H), 4.41 (dd, *J* = 14.9, 5.8 Hz, 1H), 4.16 (dd, *J* = 14.0, 8.6 Hz, 1H), 3.67 (dd, *J* = 14.0, 8.8 Hz, 1H).
^{13}C NMR (75 MHz, $CDCl_3$): δ_C 172.1, 167.1, 137.3, 134.2, 134.1, 131.5, 131.4, 128.9, 127.8, 127.7, 125.2, 124.4, 74.1, 53.3, 52.3, 44.1.
HRMS (ESI$^+$): Calcd for $C_{18}H_{17}N_2O_4S$ $[M+H]^+$ 357.0904, found 357.0920.
$[\alpha]_D^{25}$= -118.3 (c 0.240 g/100 mL, CH_2Cl_2).

(3*R*,9*bS*)-5-Oxo-*N*-(3-(trifluoromethyl)benzyl)-2,3,5,9*b*-tetrahydrothiazolo[2,3-*a*]isoindole-3-carboxamide 1,1-dioxide (7aD)

Following the application of the general procedure, this product **7aD** was isolated as white solid, R_f = 0.66, eluent (ethyl acetate / cyclohexane = 6/4), mp = 188-190 °C, 150 mg scale reaction (in 10 mL of methanol), 110 mg was isolated, 68% yield.

IR (ν_{max} / cm^{-1}): 3382, 1721, 1675, 1538, 1330, 1139, 700.

^{1}H NMR (300 MHz, $CDCl_3$): δ_H 7.87 (d, *J* = 7.4 Hz, 1H), 7.76 – 7.64 (m, 3H), 7.55 – 7.39 (m, 5H), 5.66 (s, 1H), 5.04 (t, *J* = 8.8 Hz, 1H), 4.55 (t, *J* = 5.6 Hz, 1H), 4.25 (dd, *J* = 14.2, 9.1 Hz, 1H), 3.74 (dd, *J* = 14.2, 8.5 Hz, 1H).

^{13}C NMR (75 MHz, $CDCl_3$): δ_C 172.5, 167.2, 138.4, 134.3, 134.3, 131.6, 131.5, 131.2 (q, *J* = 32.4 Hz), 131.1 (d, *J* = 1.1 Hz), 129.5, 127.6 (q, *J* = 272.3 Hz), 125.4, 124.8 (q, *J* = 3.7 Hz), 124.5 (q, *J* = 3.9Hz), 124.5, 74.1, 53.5, 52.0, 43.7.

^{19}F NMR (282 MHz, $CDCl_3$): δ_F -62.7.

HRMS (ESI$^+$): Calcd for $C_{19}H_{16}F_3N_2O_4S$ $[M+H]^+$ 425.0777, found 425.0792.

$[\alpha]_D^{25}$= -108.9 (c 0.215 g/100 mL, CH_2Cl_2).

(3*R*,9*bS*)-*N*-(3-Methoxybenzyl)-5-oxo-2,3,5,9*b*-tetrahydrothiazolo[2,3-*a*]isoindole-3-carboxamide 1,1-dioxide (7aE)

Following the application of the general procedure, this product **7aE** was isolated as white solid, R_f = 0.55, eluent (ethyl acetate / cyclohexane = 6/4), mp = 194-196 °C, 185 mg scale reaction (in 10 mL of toluene), 80 mg was isolated, 39% yield.

IR (ν_{max} / cm^{-1}): 3345, 1714, 1670, 1512, 1321, 1146, 701.

^{1}H NMR (300 MHz, $CDCl_3$): δ_H 7.89 (d, *J* = 7.1 Hz, 1H), 7.77 – 7.64 (m, 3H), 7.28 – 7.22 (m, 1H), 7.23 – 7.18 (bs, 1H), 6.90 – 6.77 (m, 3H), 5.64 (s, 1H), 5.03 (t, *J* = 8.7 Hz, 1H), 4.56 – 4.40 (m, 2H), 4.26 (dd, *J* = 14.2, 8.9 Hz, 1H), 3.78 (s, 3H), 3.72 (m, 1H).

^{13}C NMR (75 MHz, $CDCl_3$): δ_C 172.3, 167.0, 160.1, 138.8, 134.3, 131.6, 130.1, 125.4, 124.5, 120.0, 113.4, 113.3, 74.1, 55.4, 53.5, 52.2, 44.2.

HRMS (ESI$^+$): Calcd for $C_{19}H_{19}N_2O_5S$ $[M+H]^+$ 387.1009, found 421.1035.

$[\alpha]_D^{25}$= -129.0 (c 0.335 g/100 mL, CH_2Cl_2).

(3*R*,9*bS*)-*N*-(4-Methoxybenzyl)-5-oxo-2,3,5,9*b*-tetrahydrothiazolo[2,3-*a*]isoindole-3-carboxamide 1,1-dioxide (7aF)

Following the application of the general procedure, this product **7aF** was isolated as light brown solid, R_f = 0.56, eluent (ethyl acetate / cyclohexane = 6/4), mp = 134-136 °C, 150 mg scale reaction (10 mL of methanol), 150 mg was isolated, 91% yield.

IR (ν_{max} / cm^{-1}): 3348, 1715, 1669, 1528, 1321, 1146, 701.

^{1}H NMR (300 MHz, $CDCl_3$): δ_H 7.79 (d, *J* = 7.3 Hz, 1H), 7.72 – 7.59 (m, 3H), 7.30 – 7.25 (bs, 1H), 7.18 (d, *J* = 8.2 Hz, 2H), 6.83 (d, *J* = 8.6 Hz, 2H), 5.65 (s, 1H), 4.99 (t, *J* = 8.6 Hz, 1H), 4.44 (dd, *J* = 14.6, 5.9 Hz, 1H), 4.34 (dd, *J* = 14.6, 5.6 Hz, 1H), 4.17 (dd, *J* = 14.1, 8.6 Hz, 1H), 3.76 (s, 3H), 3.69 (dd, *J* = 14.1, 8.6 Hz, 1H).

^{13}C NMR (75 MHz, $CDCl_3$): δ_C 172.0, 167.0, 159.2, 134.2, 134.1, 131.5, 131.4, 129.4, 129.2, 125.2, 124.4, 114.3, 74.1, 55.4, 53.3, 52.4, 43.6.

HRMS (ESI$^+$): Calcd for $C_{19}H_{19}N_2O_5S$ $[M+H]^+$ 387.1009, found 387.1020.

$[\alpha]_D^{25}$= -209.7 (c 0.220 g/100 mL, CH_2Cl_2).

(3*R*,9*bS*)-*N*-(2-Methoxybenzyl)-5-oxo-2,3,5,9*b*-tetrahydrothiazolo[2,3-*a*]isoindole-3-carboxamide 1,1-dioxide (7aG)

Following the application of the general procedure, this product **7aG** was isolated as white solid, R_f = 0.61, eluent (ethyl acetate / cyclohexane = 6/4), mp = 83-85 °C, 180 mg scale reaction (in 10 mL of methanol), 160 mg was isolated, 82% yield.

IR (ν_{max} / cm^{-1}): 3363, 1712, 1676, 1516, 1318, 1139, 722.
^{1}H NMR (300 MHz, CDCl$_3$): δ_H 7.89 (d, J = 7.1 Hz, 1H), 7.72 – 7.63 (m, 3H), 7.40 (t, J = 6.2 Hz, 1H), 7.33 – 7.26 (m, 1H), 7.30 – 7.23 (m, 1H), 6.89 (dd, J = 15.0, 7.6 Hz, 2H), 5.62 (s, 1H), 4.99 (t, J = 8.7 Hz, 1H), 4.60 – 4.37 (m, 2H), 4.23 (dd, J = 14.2, 8.7 Hz, 1H), 3.85 (s, 3H), 3.69 (dd, J = 14.2, 8.7 Hz, 1H).
^{13}C NMR (75 MHz, CDCl$_3$): δ_C 171.9, 166.7, 157.7, 134.2, 134.1, 131.7, 131.5, 129.7, 129.3, 125.3, 125.3, 124.4, 120.8, 110.5, 74.1, 55.4, 53.3, 52.2, 40.4.
HRMS (ESI$^+$): Calcd for $C_{19}H_{19}N_2O_5S$ [M+H]$^+$ 387.1009, found 387.1013.
$[\alpha]_D^{25}$= 100.4 (c 0.240 g/100 mL, CH_2Cl_2).

***N*-(3,4-Dimethoxybenzyl)-5-oxo-2,3,5,9*b*-tetrahydrothiazolo[2,3-*a*]isoindole-3-carboxamide 1,1-dioxide (7aI)**

Following the application of the general procedure, this product **7aI** was isolated as light brown solid, R_f = 0.24, eluent (ethyl acetate / cyclohexane = 5/5), mp = 98-100 °C, 200 mg scale reaction (in 10 mL of methanol), 100 mg was isolated, 46% yield.

IR (ν_{max} / cm^{-1}): 3339, 1711, 1675, 1514, 1328, 1137, 721.
^{1}H NMR (300 MHz, CDCl$_3$): δ_H 7.77 (d, J = 7.4 Hz, 1H), 7.70 – 7.57 (m, 3H), 7.32 – 7.28 (bs, 1H), 6.81 – 6.75 (m, 3H), 5.65 (s, 1H), 5.00 (t, J = 8.6 Hz, 1H), 4.43 (dd, J = 14.7, 5.9 Hz, 1H), 4.35 (dd, J = 14.6, 5.7 Hz, 1H), 4.15 (dd, J = 14.1, 8.5 Hz, 1H), 3.80 (d, J = 2.1 Hz, 6H, H-17), 3.69 (dd, J = 14.2, 8.6 Hz, 1H).
^{13}C NMR (75 MHz, CDCl$_3$): δ_C 172.0, 166.9, 149.1, 148.6, 134.2, 134.1, 131.4, 131.4, 129.8, 125.1, 124.4, 120.0, 111.3, 111.0, 74.1, 55.9, 55.9, 53.3, 52.3, 43.9.
HRMS (ESI$^+$): Calcd for $C_{20}H_{20}N_2NaO_6S$ [M+Na]$^+$ 439.0934, found 439.0952.
$[\alpha]_D^{25}$= -77.5 (c 0.360 g/100 mL, CH_2Cl_2).

VI. General procedure for the synthesis of *N*-benzyl-9*b*-methyl-5-oxo-2,3,5,9*b*-tetrahydrothiazolo-[2,3-*a*]isoindole-3-carboxamides 1,1-dioxide

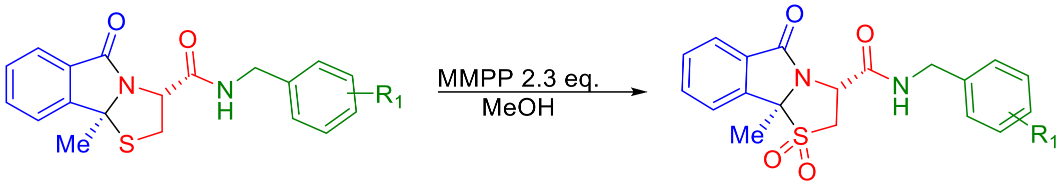

4bA, 4bD-bG, 4bI **7bA, 7bD-bG, 7bI**

These compounds were synthesized according to the same protocol as in part V.

Entry	R group	R_1 group	Product 7b
1	Me	H	**7bA**
2	Me	-[1]	**7bB**
3	Me	-[1]	**7bC**
4	Me	*m*-CF_3	**7bD**
5	Me	*m*-OMe	**7bE**
6	Me	*p*-OMe	**7bF**
7	Me	*o*-OMe	**7bG**
8	Me	*m,p*-2Cl	**7bH**
9	Me	*m,p*-2OMe	**7bI**
10	H	-[1]	**7aJ**

[1] Not realised.

(3*R*,9*bS*)-*N*-Benzyl-9*b*-methyl-5-oxo-2,3,5,9*b*-tetrahydrothiazolo[2,3-*a*]isoindole-3-carboxamide 1,1-dioxide (7bA)

Following the application of the general procedure, this product **7bA** was isolated as white solid, R_f = 0.58, eluent (ethyl acetate / cyclohexane = 6/4), mp = 91-93 °C, 185 mg scale reaction (in 10 mL of methanol), 95 mg was isolated, 47% yield.

IR (ν_{max} / cm^{-1}): 3357, 1715, 1668, 1529, 1322, 1146, 700.
^{1}H NMR (300 MHz, $CDCl_3$): δ_H 7.80 – 7.74 (m, 1H), 7.73 – 7.67 (m, 1H), 7.62 – 7.56 (m, 2H), 7.35 – 7.23 (m, 5H), 7.16 – 7.07 (bs, 1H), 5.00 (t, *J* = 9.0 Hz, 1H), 4.55 – 4.42 (m, 2H), 4.35 – 4.26 (m, 1H), 3.73 – 3.65 (m, 1H), 1.97 (s, 3H).
^{13}C NMR (75 MHz, $CDCl_3$): δ_C 172.0, 167.5, 139.8, 137.5, 134.3, 131.2, 130.7, 128.9, 127.8, 127.7, 125.1, 123.1, 79.7, 52.0, 49.7, 44.2, 20.5.
HRMS (ESI$^+$): Calcd for $C_{19}H_{19}N_2O_4S$ $[M+H]^+$ 371.1060, found 371.1060.
$[\alpha]_D^{25}$= -249.2 (c 0.250 g/100 mL, CH_2Cl_2).

(3*R*,9*bS*)-9*b*-Methyl-5-oxo-*N*-(3-(trifluoromethyl)benzyl)-2,3,5,9*b*-tetrahydrothiazolo[2,3-*a*]-isoindole-3-carboxamide 1,1-dioxide (7bD)

Following the application of the general procedure, this product **7bD** was isolated as white solid, R_f = 0.51, eluent (ethyl acetate / cyclohexane = 5/5), mp = 176-179 °C, 180 mg scale reaction (in 10 mL of methanol), 109 mg was isolated, 56% yield.

IR (ν_{max} / cm^{-1}): 3373, 1709, 1688, 1541, 1324, 1128, 702.
^{1}H NMR (300 MHz, $CDCl_3$): δ_H 7.73 – 7.66 (m, 2H), 7.59 – 7.56 (m, 2H), 7.54 – 7.41 (m, 4H), 7.38 – 7.34 (bs, 1H), 5.02 (t, *J* = 8.9 Hz, 1H), 4.61 (dd, *J* = 15.3, 6.5 Hz, 1H), 4.44 (dd, *J* = 15.3, 5.8 Hz, 1H), 4.29 (dd, *J* = 14.3, 8.5 Hz, 1H), 3.71 (dd, *J* = 14.3, 9.4 Hz, 1H), 1.96 (s, 3H).
^{13}C NMR (75 MHz, $CDCl_3$): δ_C 172.2, 167.8, 139.7, 138.8, 134.4, 131.2, 131.0 (q, *J* = 32.3 Hz), 130.9 (d, *J* = 1.5 Hz), 130.6, 129.3, 124.9, 124.5 (q, *J* = 3.8 Hz), 124.1 (q, *J* = 3.8 Hz), 124.0 (q, *J* = 272.3 Hz), 123.1, 79.7, 52.0, 49.6, 43.4, 20.4.
^{19}F NMR (282 MHz, $CDCl_3$): δ -62.7.
HRMS (ESI$^+$): Calcd for $C_{20}H_{17}F_3N_2NaO_4S$ $[M+Na]^+$ 461.0753, found 461.0767.
$[\alpha]_D^{25}$= -216.32 (c 0.250 g/100 mL, CH_2Cl_2).

(3*R*,9*bS*)-*N*-(3-Methoxybenzyl)-9*b*-methyl-5-oxo-2,3,5,9*b*-tetrahydrothiazolo[2,3-*a*]isoindole-3-carboxamide 1,1-dioxide (7bE)

Following the application of the general procedure, this product **7bE** was isolated as white solid, R_f = 0.65, eluent (ethyl acetate / cyclohexane = 6/4), mp = 156-158 °C, 250 mg scale reaction (in 10 mL of methanol), 183 mg was isolated, 67% yield.

IR (ν_{max} / cm^{-1}): 3358, 1709, 1689, 1556, 1321, 1148, 693.
^{1}H NMR (300 MHz, $CDCl_3$): δ 7.78 (d, *J* = 8.1 Hz, 1H), 7.71 (td, *J* = 7.4, 1.2 Hz, 1H), 7.63 – 7.58 (m, 2H), 7.27 – 7.21 (m, 1H), 7.14 – 7.10 (bst, 1H), 6.85 – 6.79 (m, 3H), 5.01 (t, *J* = 8.9 Hz, 1H), 4.54 – 4.40 (m, 2H), 4.31 (dd, *J* = 14.2, 8.4 Hz, 1H), 3.76 (s, 3H), 3.70 (dd, *J* = 14.2, 9.5 Hz, 1H), 1.99 (s, 3H).
^{13}C NMR (75 MHz, $CDCl_3$): δ_C 172.0, 167.5, 160.0, 139.8, 139.0, 134.3, 131.2, 130.7, 130.0, 125.0, 123.1, 119.8, 113.4, 113.1, 79.7, 55.3, 52.0, 49.7, 44.1, 20.5.
HRMS (ESI$^+$): Calcd for $C_{20}H_{21}N_2O_5S$ $[M+H]^+$ 401.1166, found 401.1182.
$[\alpha]_D^{25}$= -124.2 (c 0.210 g/100 mL, CH_2Cl_2).

(3*R*,9*bS*)-*N*-(4-Methoxybenzyl)-9*b*-methyl-5-oxo-2,3,5,9*b*-tetrahydrothiazolo[2,3-*a*]isoindole-3-carboxamide 1,1-dioxide (7bF)

Following the application of the general procedure, this product **7bF** was isolated as beige solid, R_f = 0.48, eluent (ethyl acetate / cyclohexane = 6/4), mp = 91-93 °C, 150 mg scale reaction (in 10 mL of methanol), 120 mg was isolated, 74% yield.

IR (ν_{max} / cm^{-1}): 3345, 1714, 1668, 1512, 1321, 1146, 701.
^{1}H NMR (300 MHz, CDCl$_3$): δ_H 7.79 (d, *J* = 7.4 Hz, 1H), 7.70 (td, *J* = 7.5, 1.3 Hz, 1H), 7.63 – 7.57 (m, 2H), 7.18 (d, *J* = 8.6 Hz, 2H), 7.02 – 6.88 (bs, 1H), 6.83 (d, *J* = 8.6 Hz, 2H), 4.99 (t, *J* = 8.9 Hz, 1H), 4.42 (dd, *J* = 5.9, 2.6 Hz, 2H), 4.32 (dd, *J* = 14.3, 8.5 Hz, 1H), 3.77 (s, 3H), 3.70 (dd, *J* = 14.3, 9.5 Hz, 1H), 1.97 (s, 3H).
^{13}C NMR (75 MHz, CDCl$_3$): δ_C 172.0, 167.4, 159.3, 139.8, 134.3, 131.2, 130.8, 129.5, 129.1, 125.1, 123.1, 114.3, 79.7, 55.4, 52.0, 49.8, 43.7, 20.6.
HRMS (ESI$^+$): Calcd for $C_{20}H_{20}N_2NaO_5S$ [M+Na]$^+$ 423.0985, found 423.0999.
$[\alpha]_D^{25}$= 53.1 (c 0.300 g/100 mL, CH_2Cl_2).

(3*R*,9*bS*)-*N*-(2-Methoxybenzyl)-9*b*-methyl-5-oxo-2,3,5,9*b*-tetrahydrothiazolo[2,3-*a*]isoindole-3-carboxamide 1,1-dioxide (7bG)

Following the application of the general procedure, this product **7bG** was isolated as white solid, R_f = 0.60, eluent (ethyl acetate / cyclohexane = 6/4), mp = 92-94 °C, 200 mg scale reaction (in 10 mL of methanol), 150 mg was isolated, 69% yield.

IR (ν_{max} / cm^{-1}): 3358, 1712, 1681, 1529, 1321, 1147, 701.
^{1}H NMR (300 MHz, CDCl$_3$): δ_H 7.83 – 7.79 (m, 1H), 7.70 – 7.64 (m, 1H), 7.62 – 7.53 (m, 2H), 7.24 – 7.20 (m, 3H), 6.85 (dd, *J* = 16.9, 8.0 Hz, 2H), 4.99 – 4.92 (m, 1H), 4.46 (d, *J* = 5.9 Hz, 2H), 4.31 – 4.21 (m, 1H), 3.85 – 3.76 (m, 3H), 3.71 – 3.55 (m, 1H), 1.89 (s, 3H).
^{13}C NMR (75 MHz, CDCl$_3$): δ_C 171.6, 167.1, 157.5, 139.7, 134.1, 131.1, 130.8, 129.5, 129.2, 125.3, 124.9, 123.0, 120.7, 110.4, 79.6, 55.3, 51.9, 49.7, 40.3, 20.3.
HRMS (ESI$^+$): Calcd for $C_{20}H_{21}N_2O_5S$ [M+Na]$^+$ 401.1166, found 401.1183.
$[\alpha]_D^{25}$= -129.3 (c 0.290 g/100 mL, CH_2Cl_2).

(3*R*,9*bS*)-*N*-(2,4-Dichlorobenzyl)-9*b*-methyl-5-oxo-2,3,5,9*b*-tetrahydrothiazolo[2,3-*a*]isoindole-3-carboxamide 1,1-dioxide (7bH)

Following the application of the general procedure, this product **7bH** was isolated as white solid, R_f = 0.29, eluent (ethyl acetate / cyclohexane = 6/4), mp = 185-187 °C, 200 mg scale reaction (in 10 mL of methanol), 128 mg was isolated, 59% yield.

IR (ν_{max} / cm^{-1}): 3362, 1715, 1685, 1319, 1139, 722.
^{1}H NMR (300 MHz, CDCl$_3$): δ_H 7.80 (d, *J* = 7.5 Hz, 1H), 7.70 (td, *J* = 7.5, 1.3 Hz, 1H), 7.65 – 7.56 (m, 1H), 7.36 (d, *J* = 2.1 Hz, 1H), 7.28 (d, *J* = 5.9 Hz, 1H), 7.26 – 7.22 (m, 1H), 7.19 (dd, *J* = 8.3, 2.1 Hz, 1H), 5.01 (t, *J* = 9.0 Hz, 1H), 4.61 – 4.43 (m, 2H), 4.25 (dd, *J* = 14.3, 8.6 Hz, 1H), 3.70 (dd, *J* = 14.3, 9.4 Hz, 1H), 1.95 (s, 3H).
^{13}C NMR (75 MHz, CDCl$_3$): δ_C 172.1, 167.6, 139.8, 134.4, 134.3, 134.3, 133.5, 131.2, 130.8, 130.6, 129.5, 127.4, 125.0, 123.1, 79.7, 52.0, 49.5, 41.6, 20.6.
HRMS (ESI$^+$): Calcd for $C_{19}H_{17}Cl_2N_2O_4S$ [M+Na]$^+$ 439.0281, found 439.0296.
$[\alpha]_D^{25}$= -274.7 (c 0.280 g/100 mL, CH_2Cl_2).

(3*R*,9*bS*)-*N*-(3,4-Dimethoxybenzyl)-9*b*-methyl-5-oxo-2,3,5,9*b*-tetrahydrothiazolo[2,3-*a*]isoindole-3-carboxamide 1,1-dioxide (7bI)

Following the application of the general procedure, this product **7bI** was isolated as beige solid, R_f = 0.38, eluent (ethyl acetate / cyclohexane = 6/4), mp = 127-129 °C, 195 mg scale reaction (in 9 mL of methanol + 1 mL of water), 141 mg was isolated, 67% yield.

IR (ν_{max} / cm^{-1}): 3363, 1710, 1674, 1516, 1321, 1138, 705.

^{1}H NMR (300 MHz, $CDCl_3$): δ_H 7.79 (d, *J* = 7.4 Hz, 1H), 7.71 (td, *J* = 7.5, 1.3 Hz, 1H), 7.60 (td, *J* = 8.0, 7.5, 2.0 Hz, 2H), 7.02 (t, *J* = 6.0 Hz), 6.82 – 6.78 (m, 3H), 4.99 (t, *J* = 9.0 Hz, 1H), 4.43 (dd, *J* = 5.9, 3.1 Hz, 2H), 4.32 (dd, *J* = 14.3, 8.6 Hz, 1H), 3.84 (s, 3H), 3.83 (s, 3H), 3.71 (dd, *J* = 14.2, 9.4 Hz, 1H), 1.97 (s, 3H).
^{13}C NMR (75 MHz, $CDCl_3$): δ_C 172.0, 167.3, 149.2, 148.7, 139.8, 134.3, 131.3, 130.7, 130.0, 125.1, 123.1, 120.1, 111.3, 111.0, 79.7, 56.0, 56.0, 52.1, 49.7, 44.0, 20.6.
HRMS (ESI^+): Calcd for $C_{21}H_{23}N_2O_6S$ $[M+H]^+$ 431.1271, found 431.1292.
$[\alpha]_D^{25}$= -47.6 (c 0.315 g/100 mL, CH_2Cl_2).

VII. Copies of NMR spectra

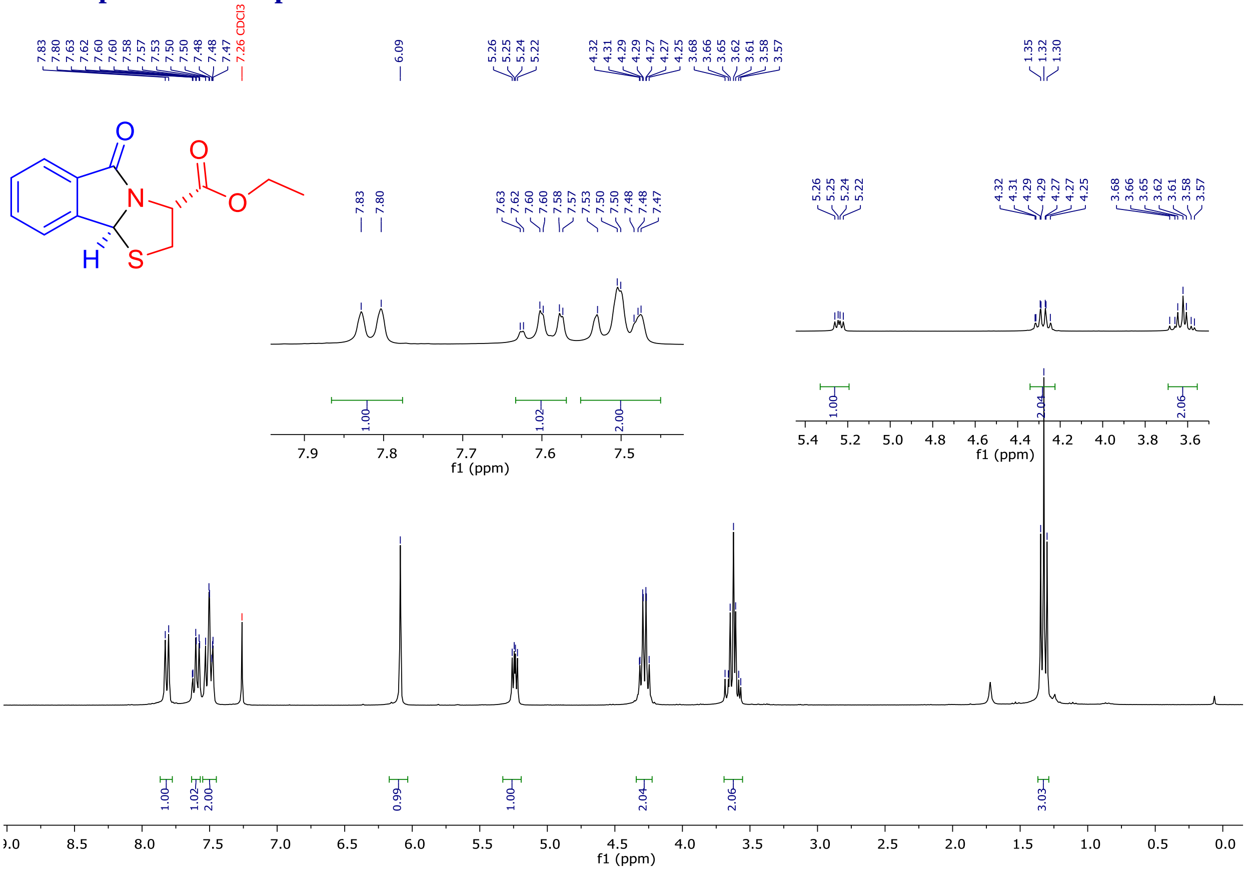

^{1}H NMR spectrum of compound **3a**

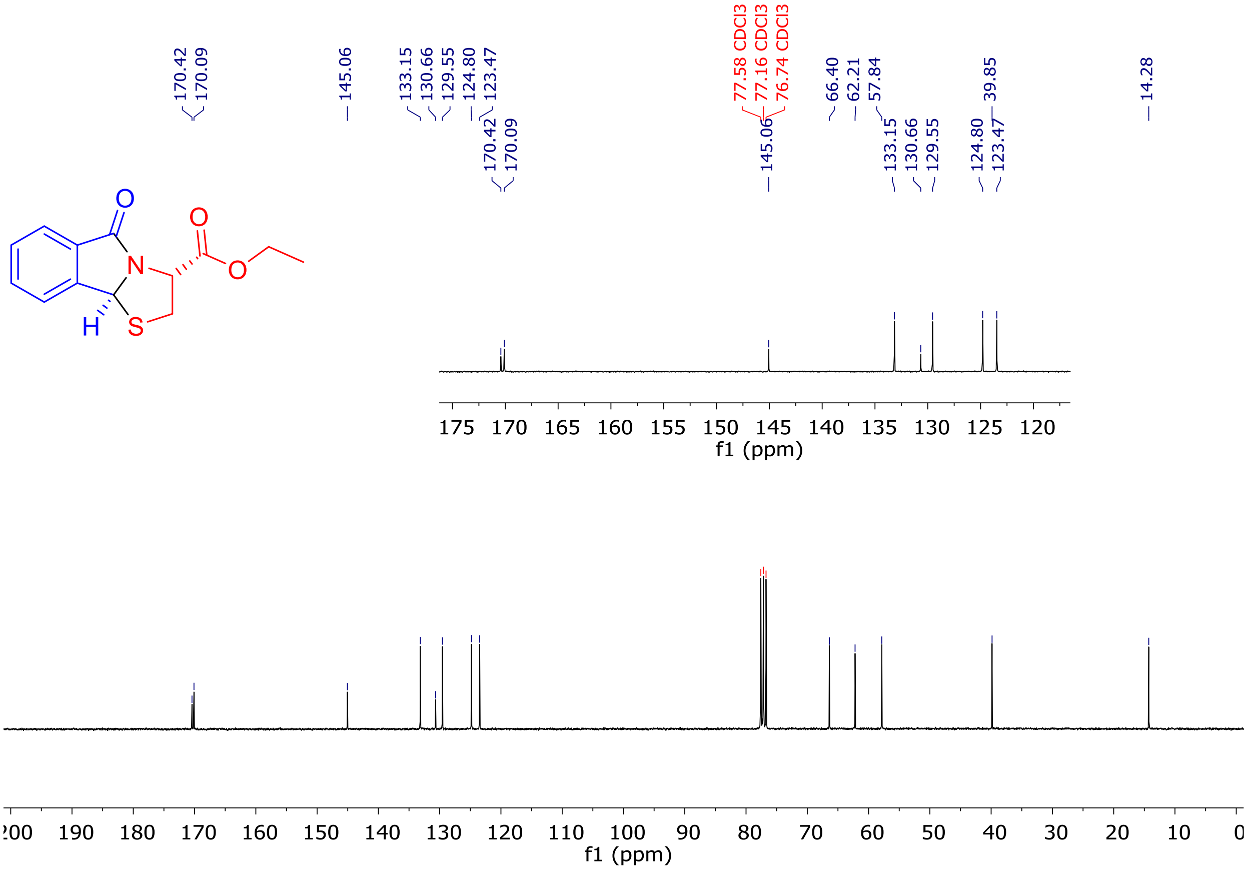

^{13}C NMR spectrum of compound **3a**

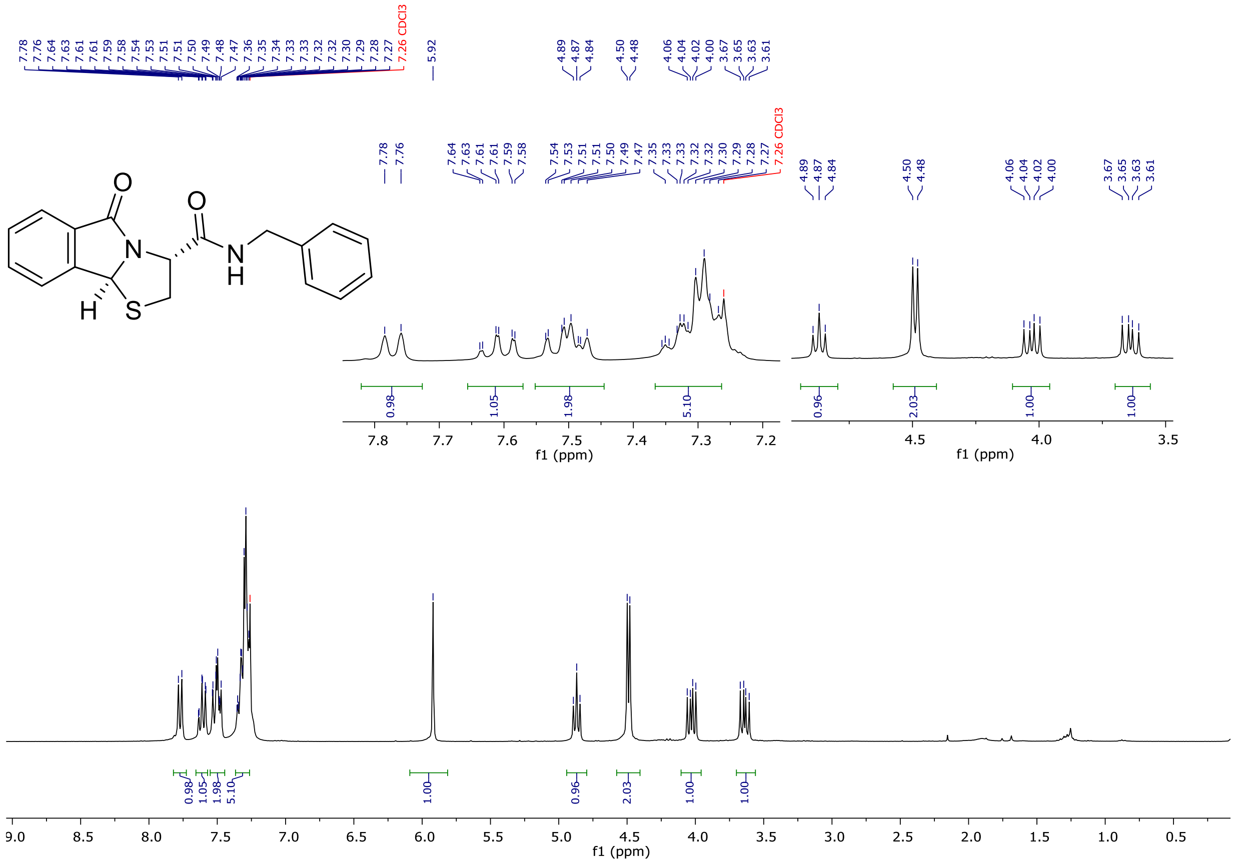

^{1}H NMR spectrum of compound **4aA**

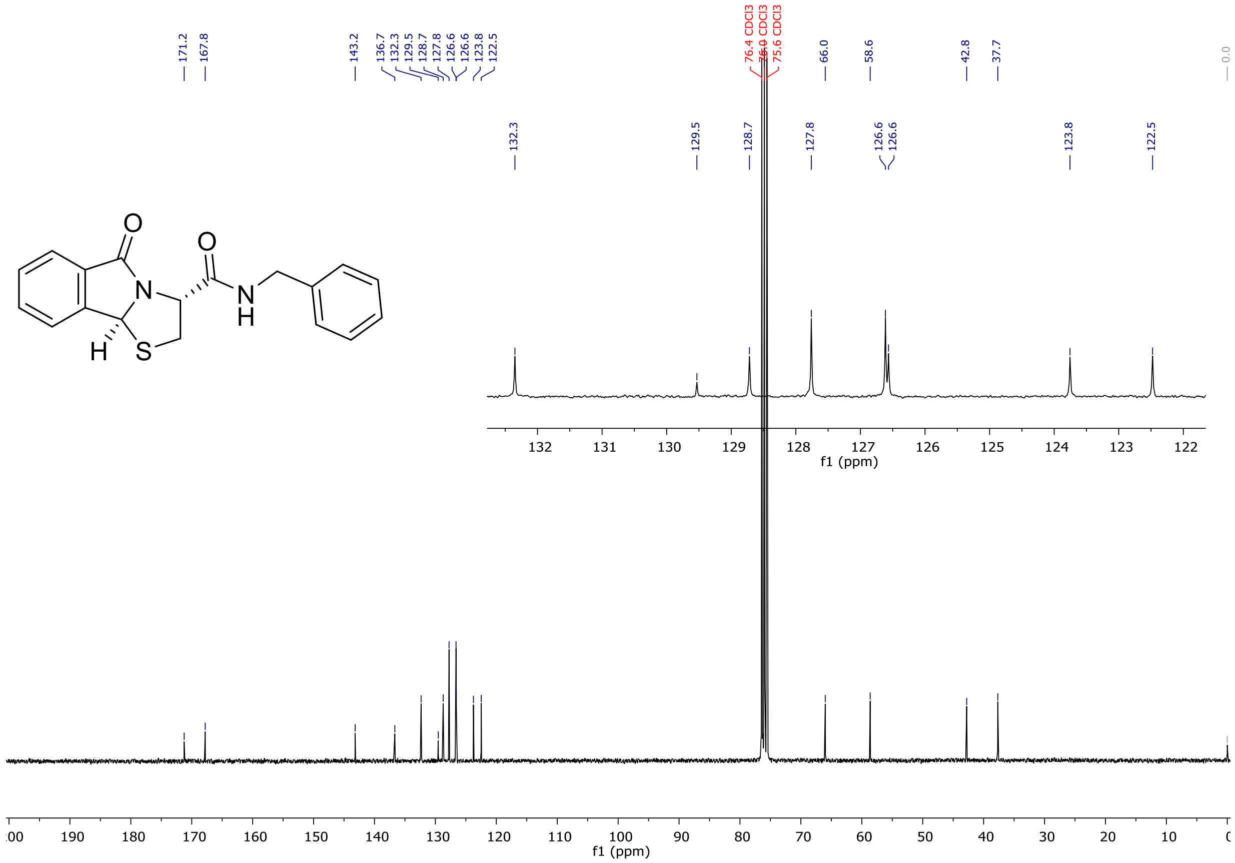

^{13}C NMR spectrum of compound **4aA**

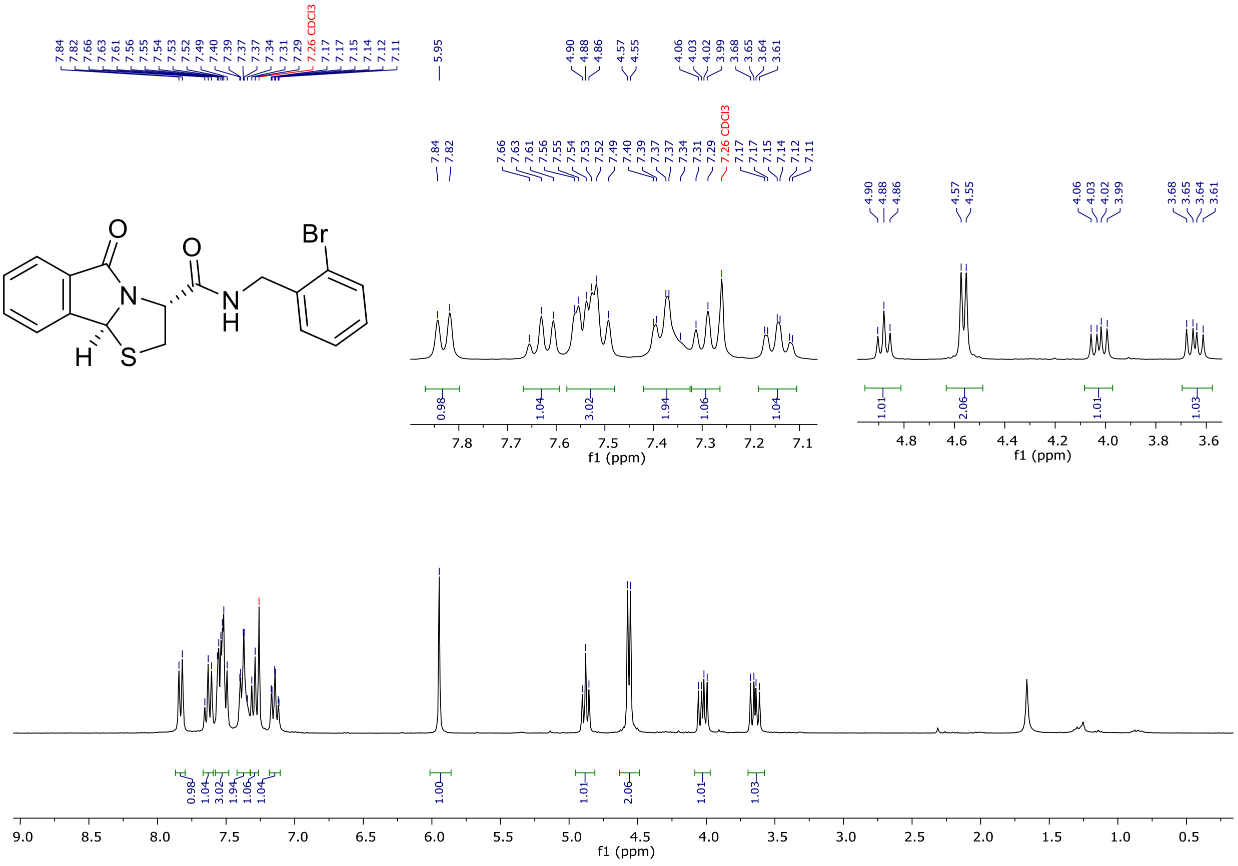

^{1}H NMR spectrum of compound **4aB**

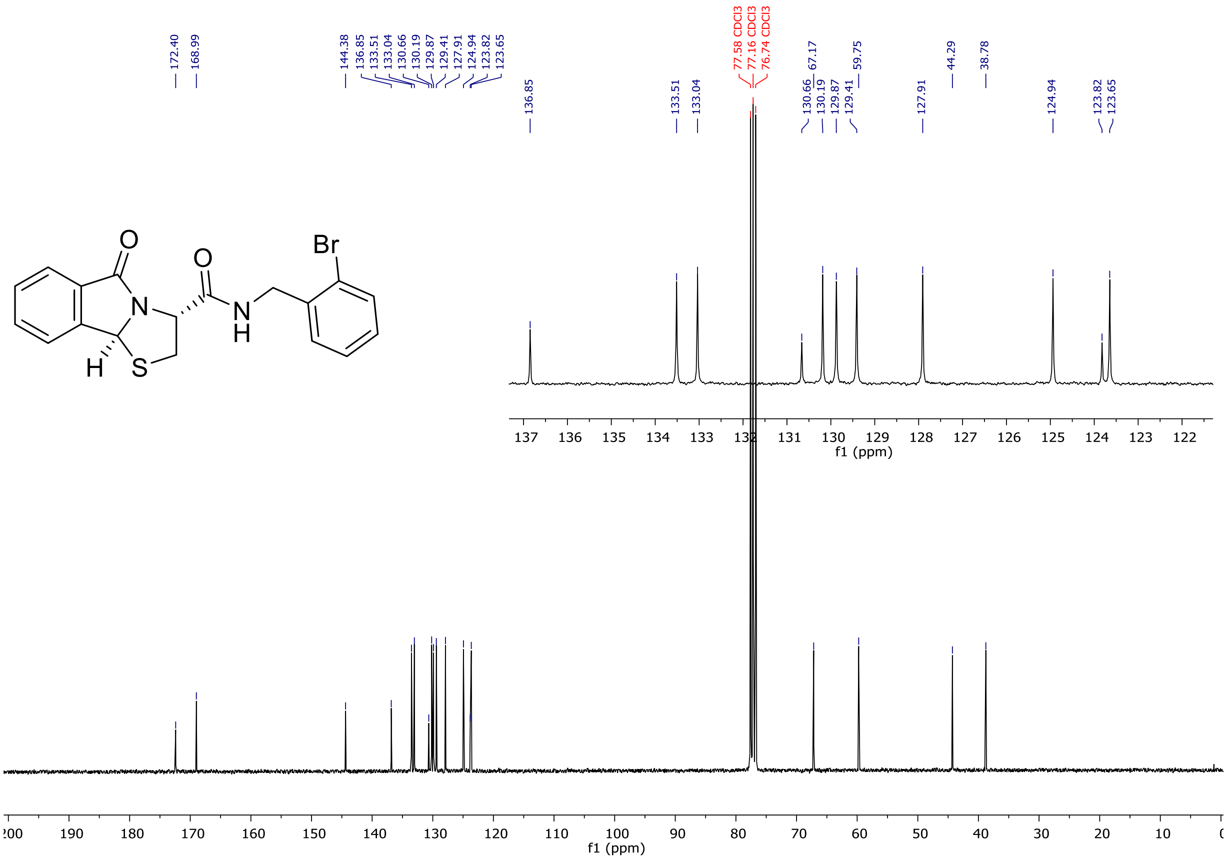

^{13}C NMR spectrum of compound **4aB**

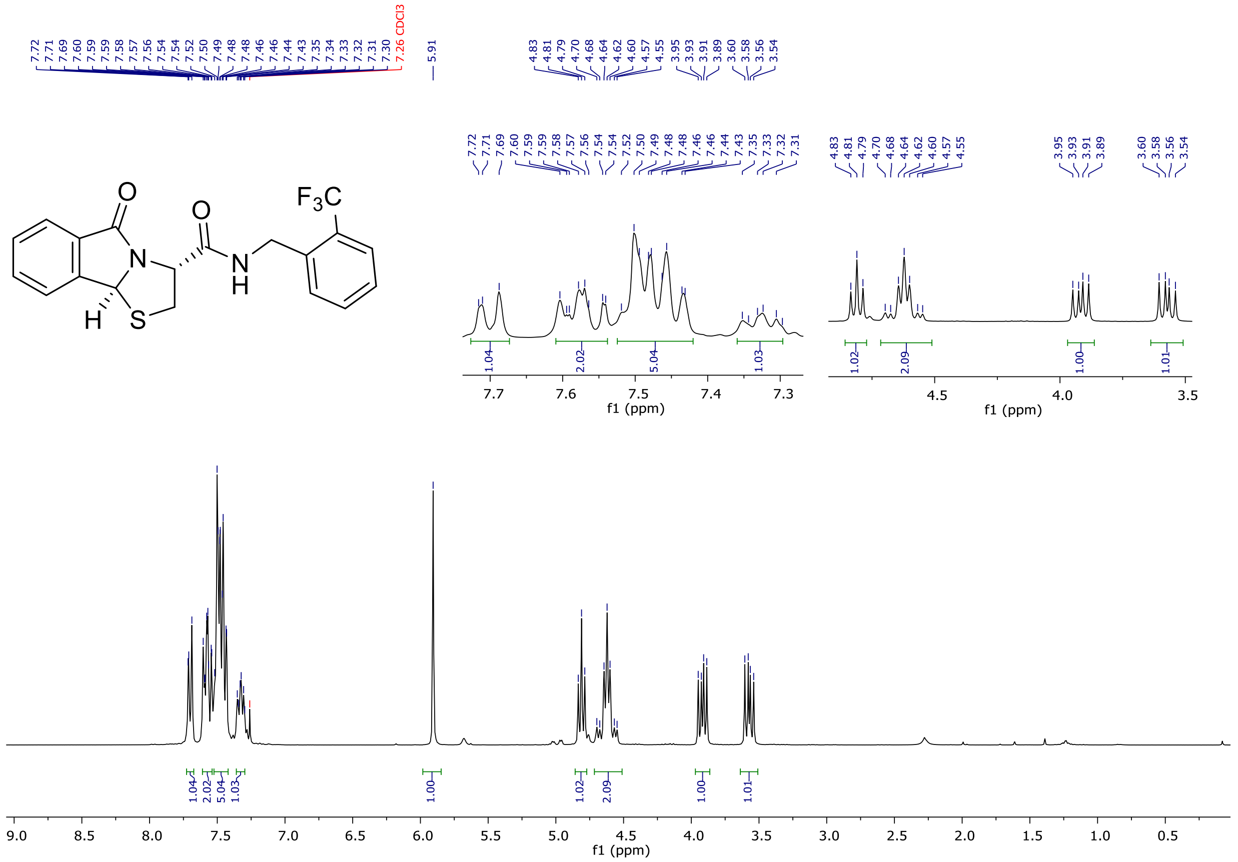

^{1}H NMR spectrum of compound **4aC**

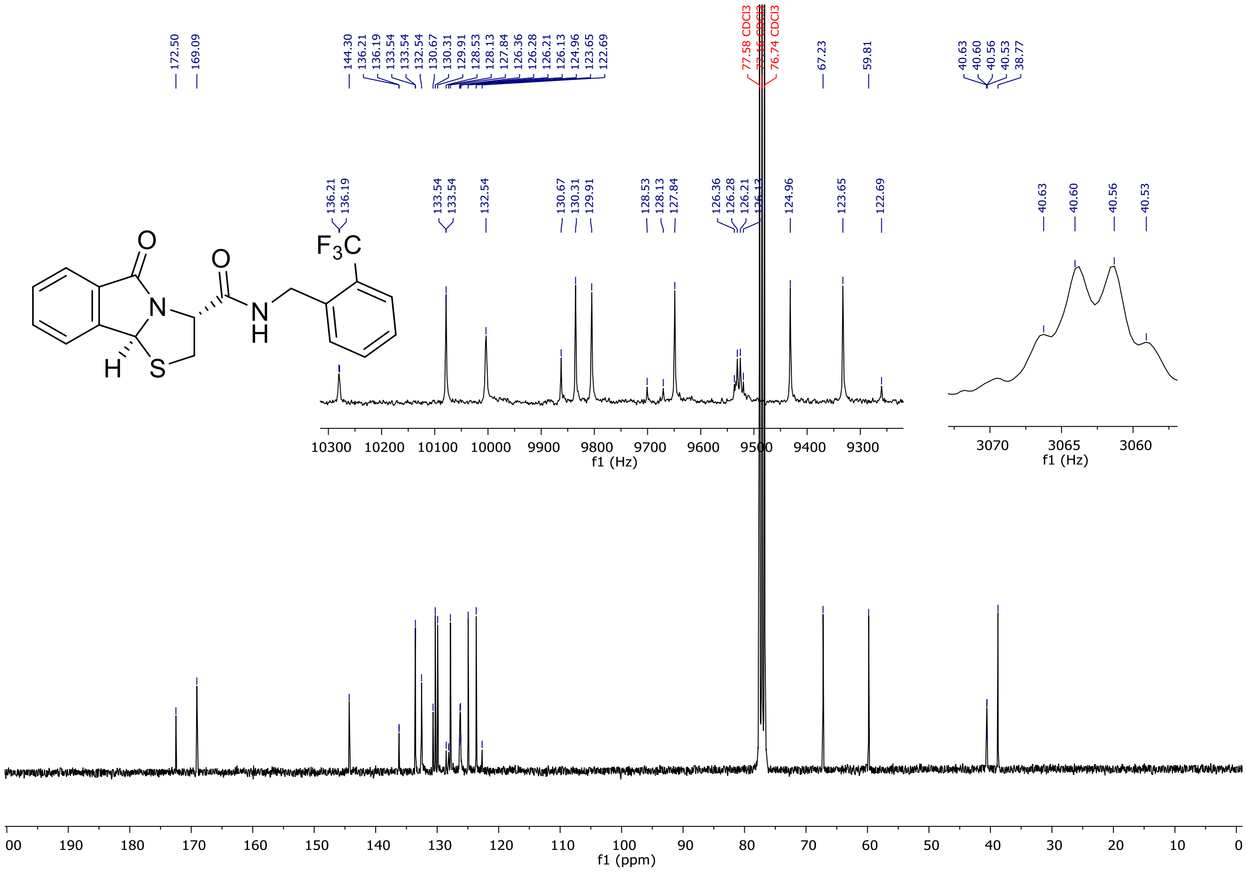

^{13}C NMR spectrum of compound **4aC**

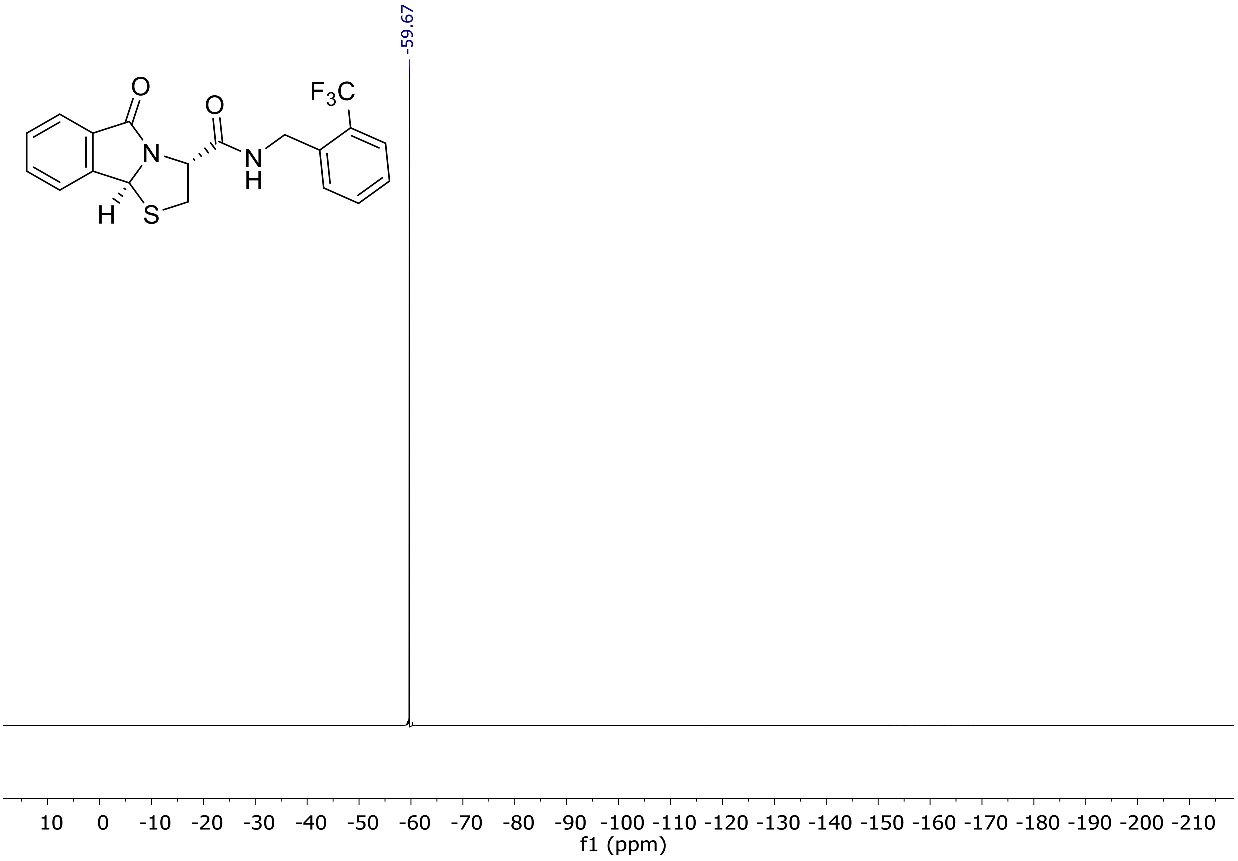

^{19}F NMR spectrum of compound **4aC**

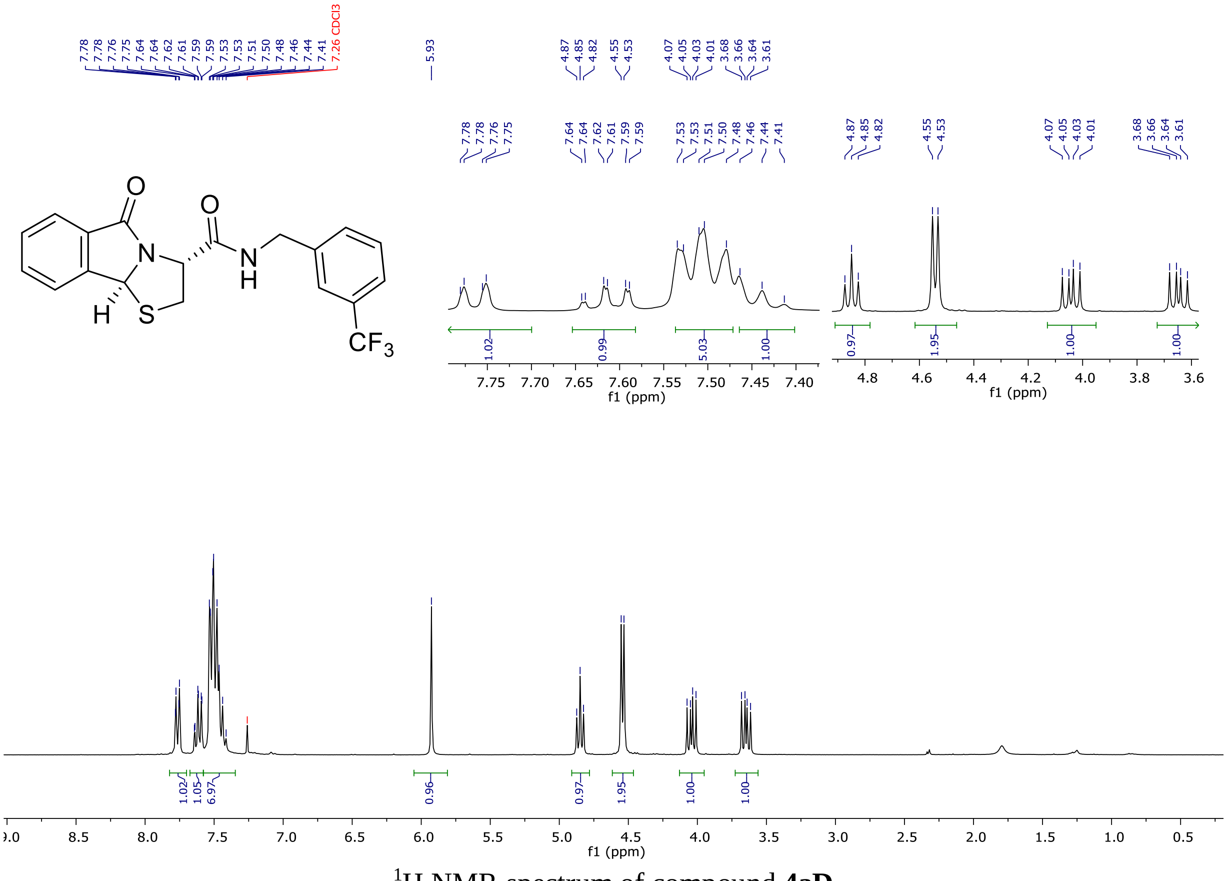

^{1}H NMR spectrum of compound **4aD**

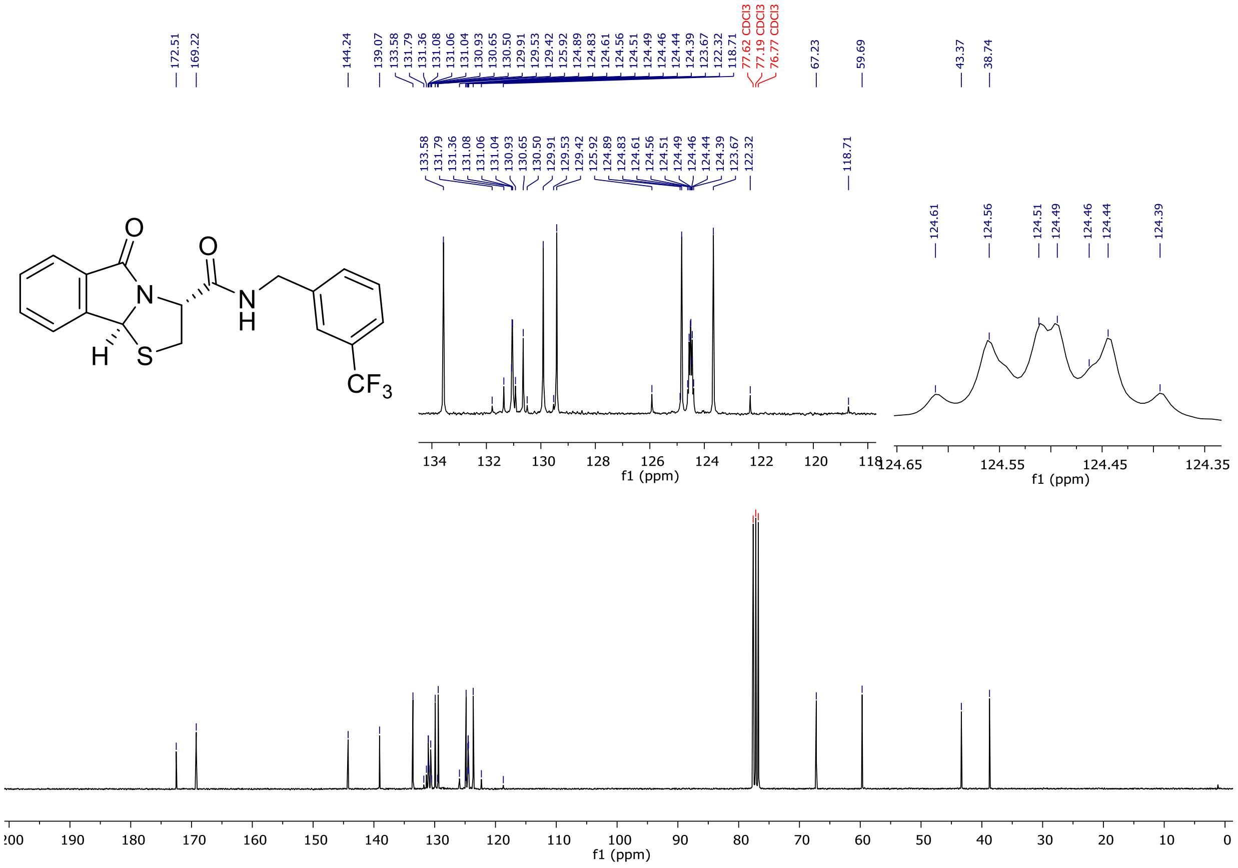

^{13}C NMR spectrum of compound **4aD**

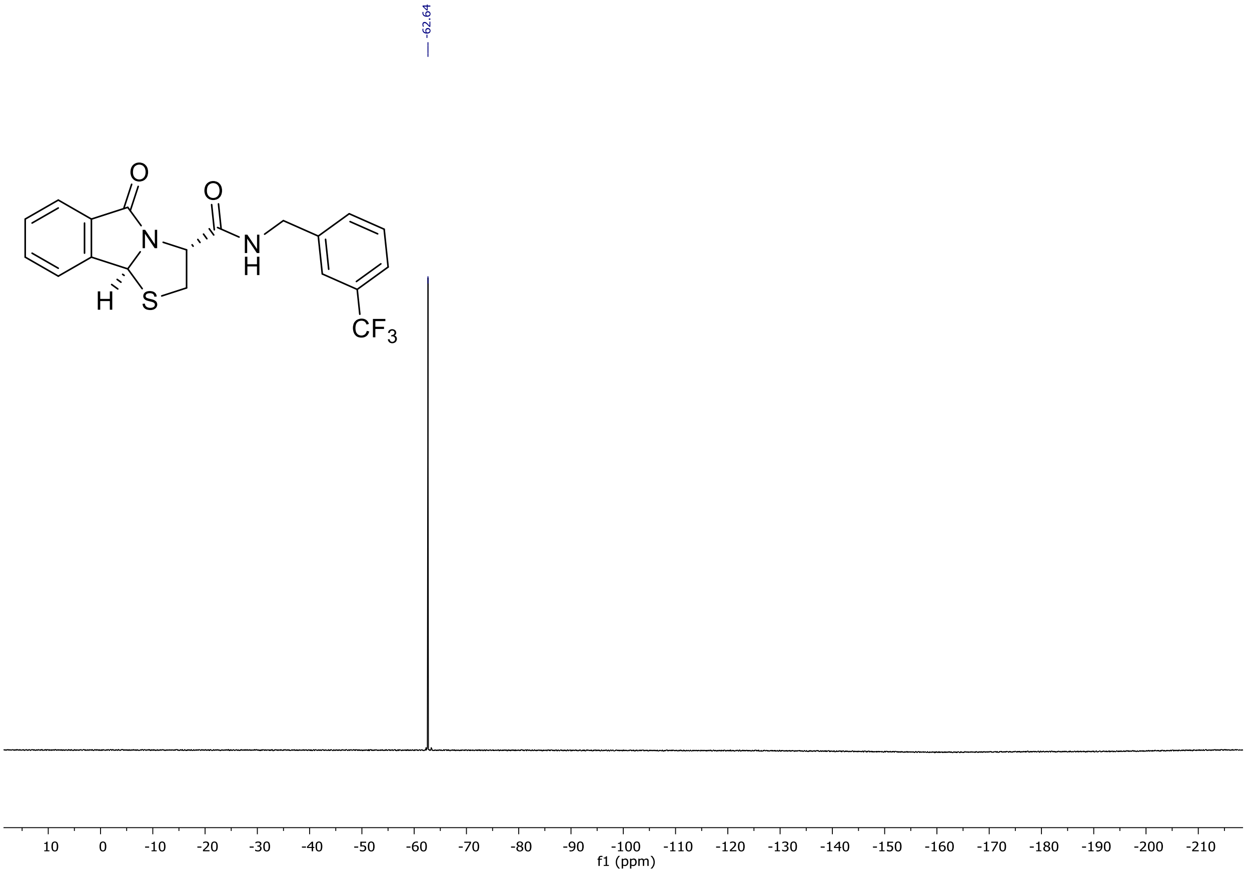

^{19}F NMR spectrum of compound **4aD**

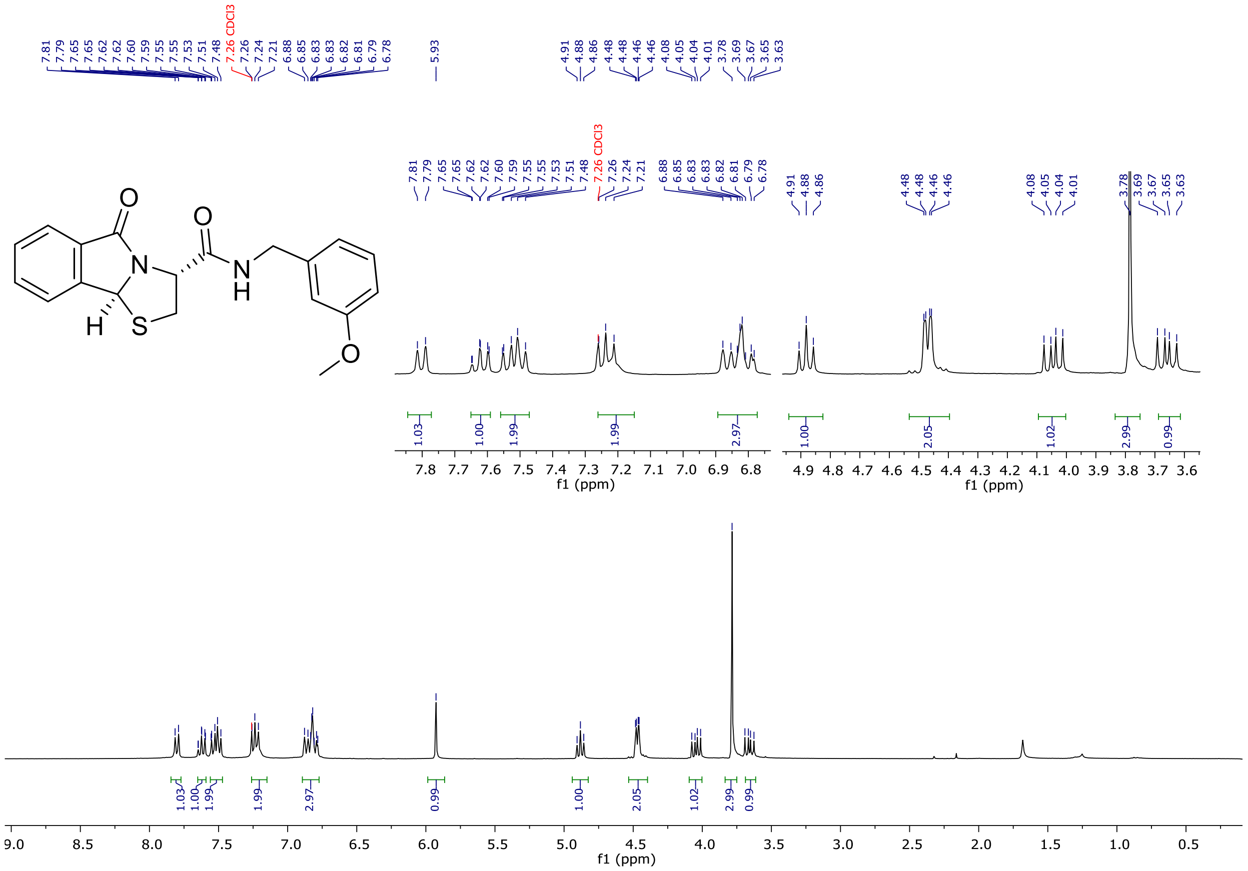

^{1}H NMR spectrum of compound **4aE**

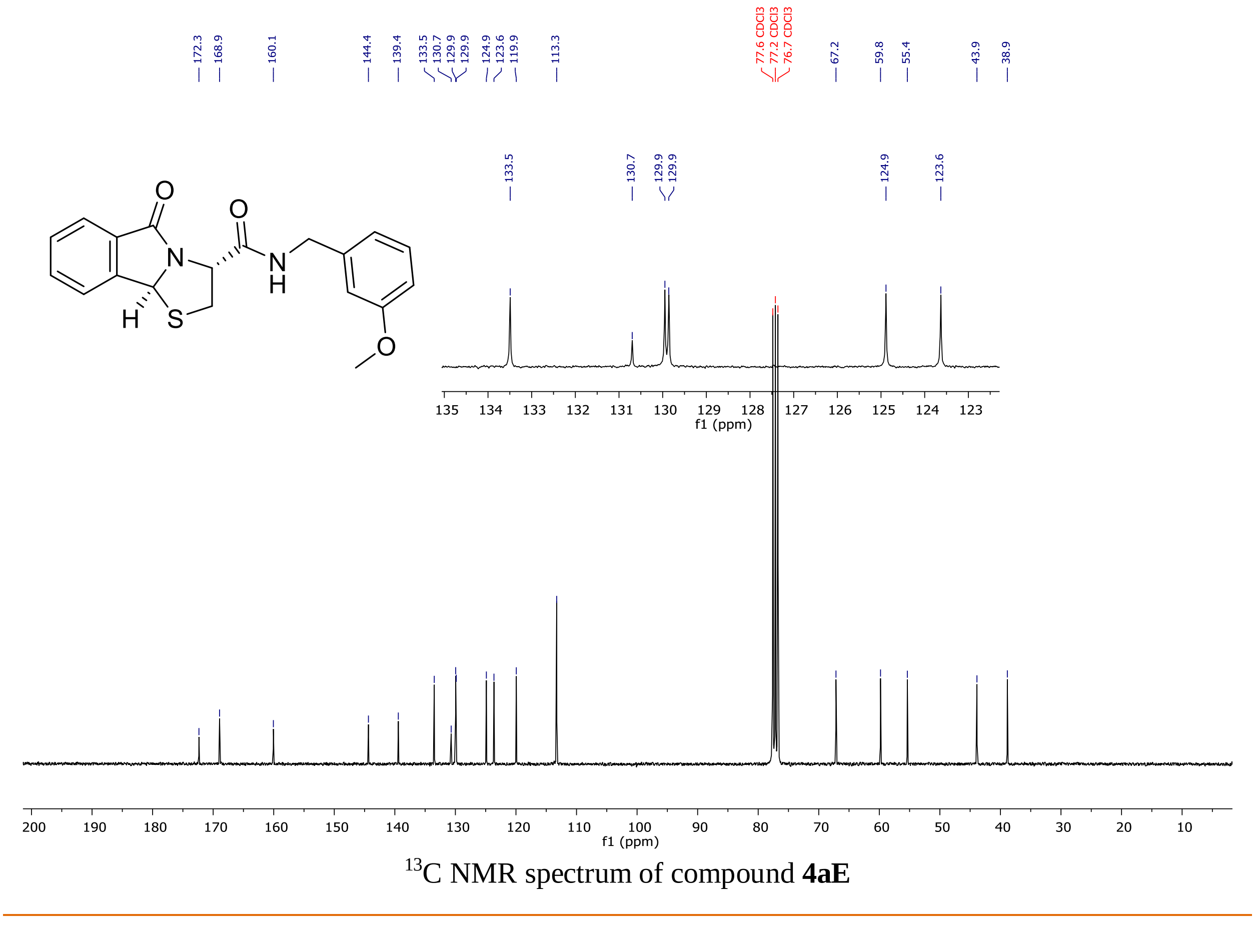

^{13}C NMR spectrum of compound **4aE**

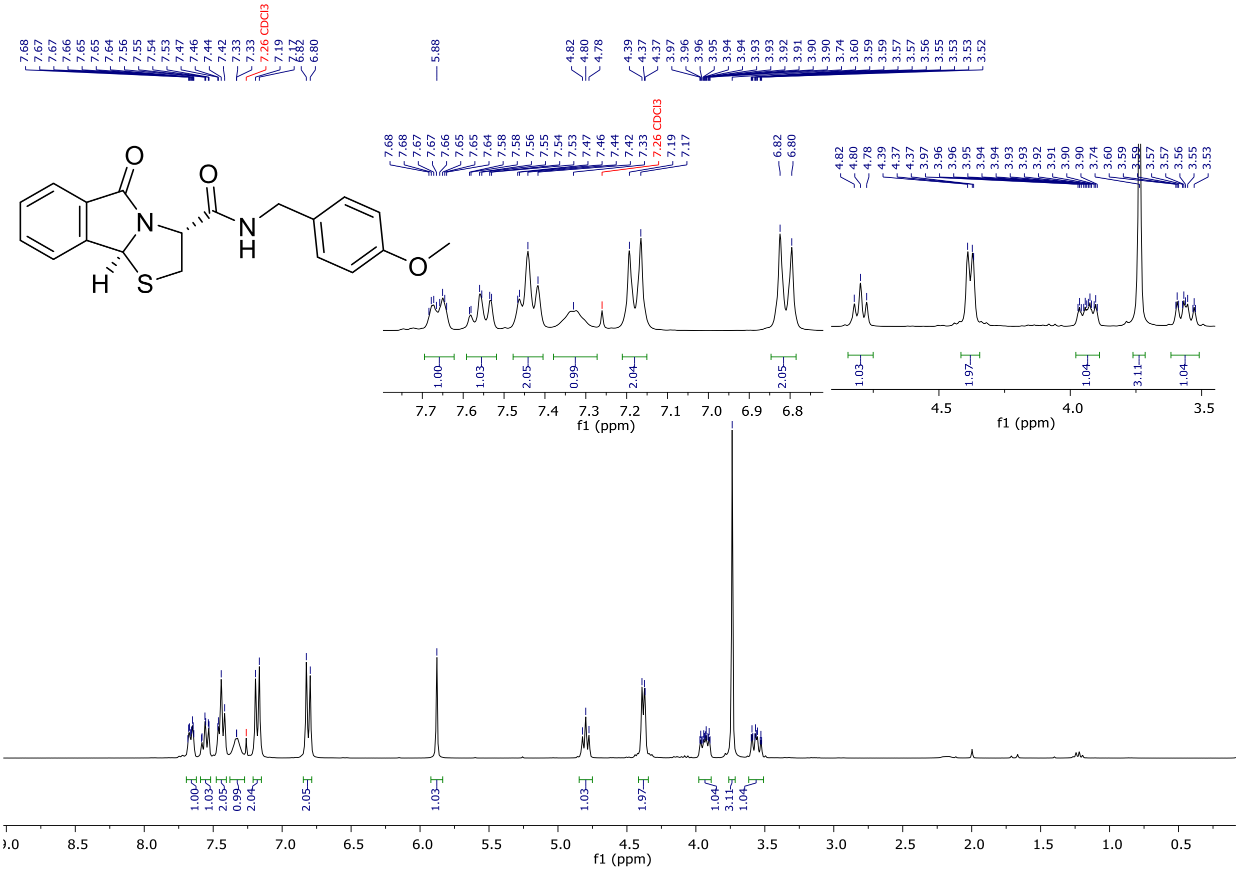

^{1}H NMR spectrum of compound **4aF**

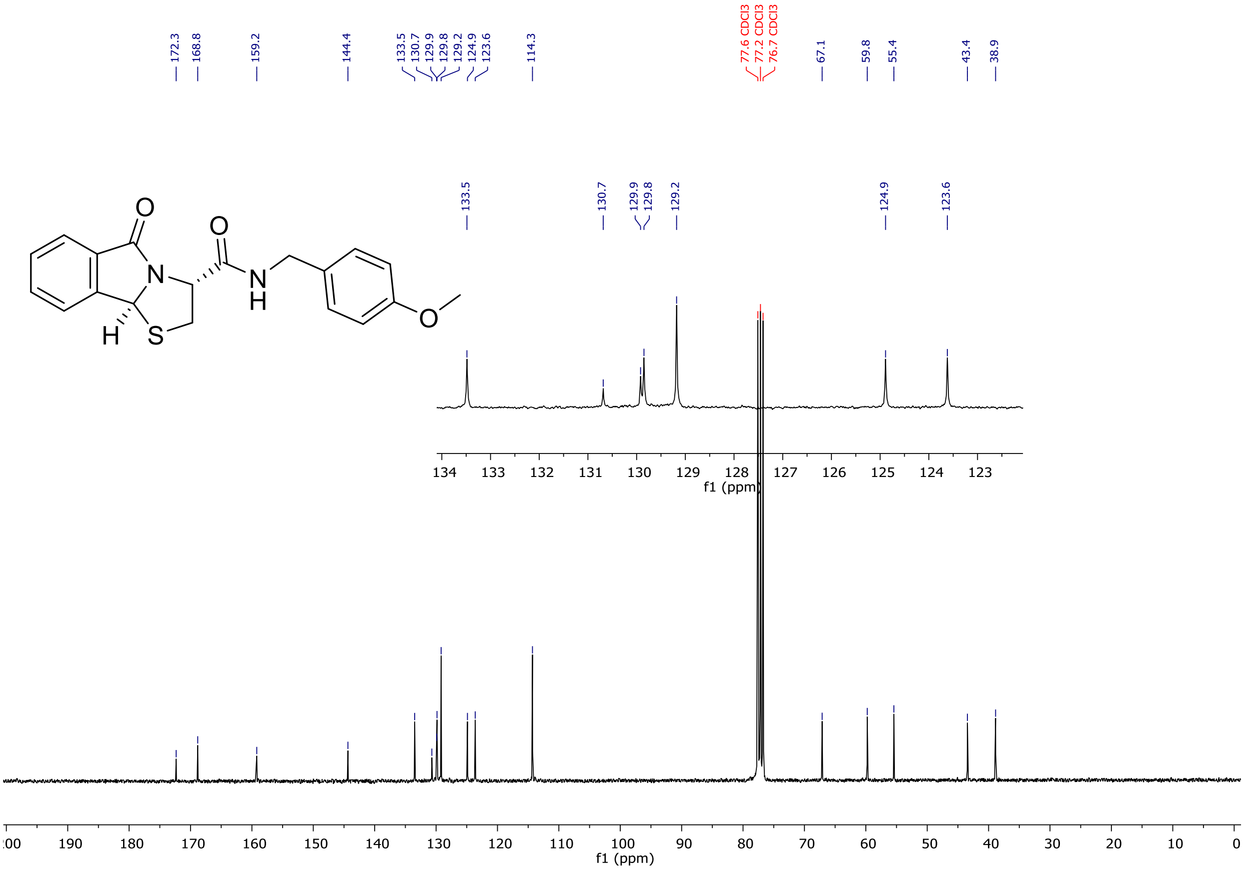

^{13}C NMR spectrum of compound **4aF**

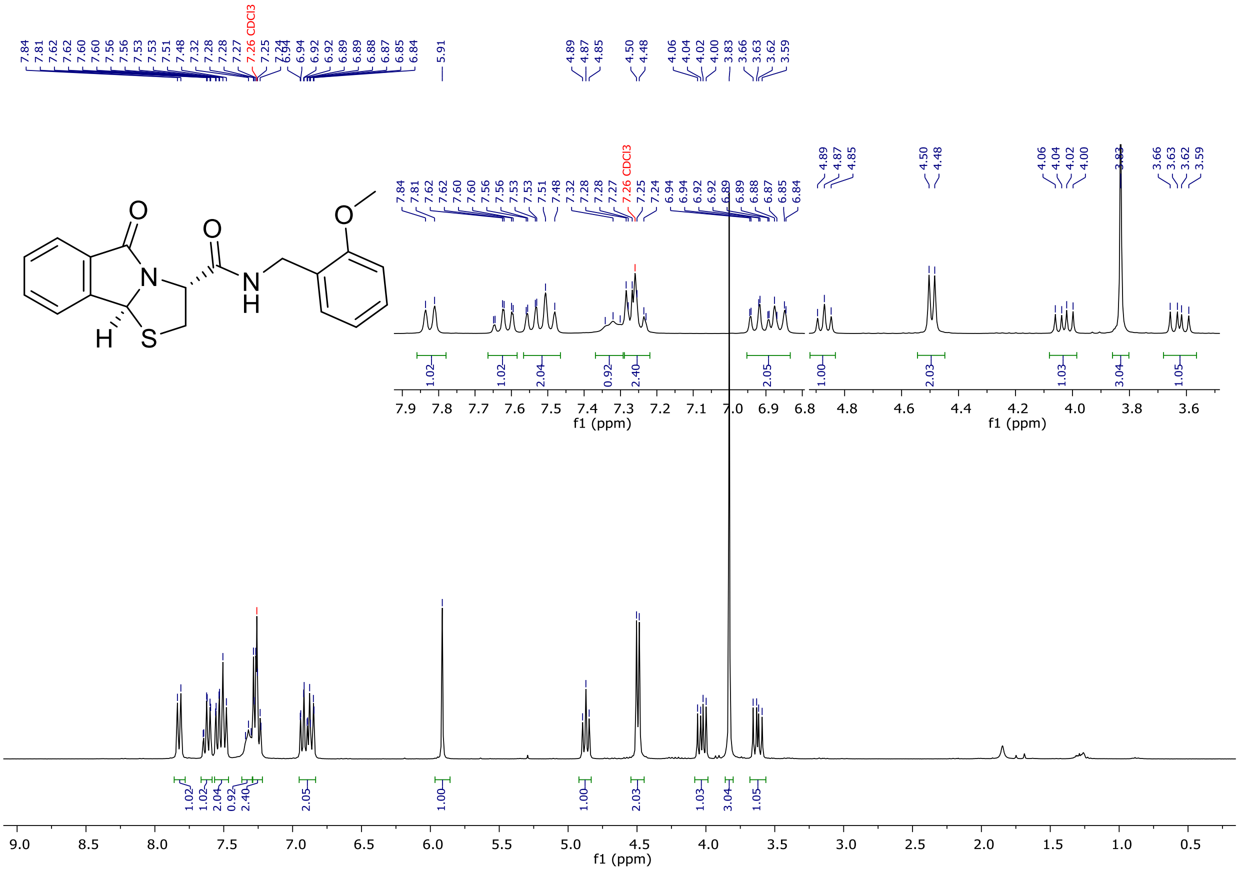

^{1}H NMR spectrum of compound **4aG**

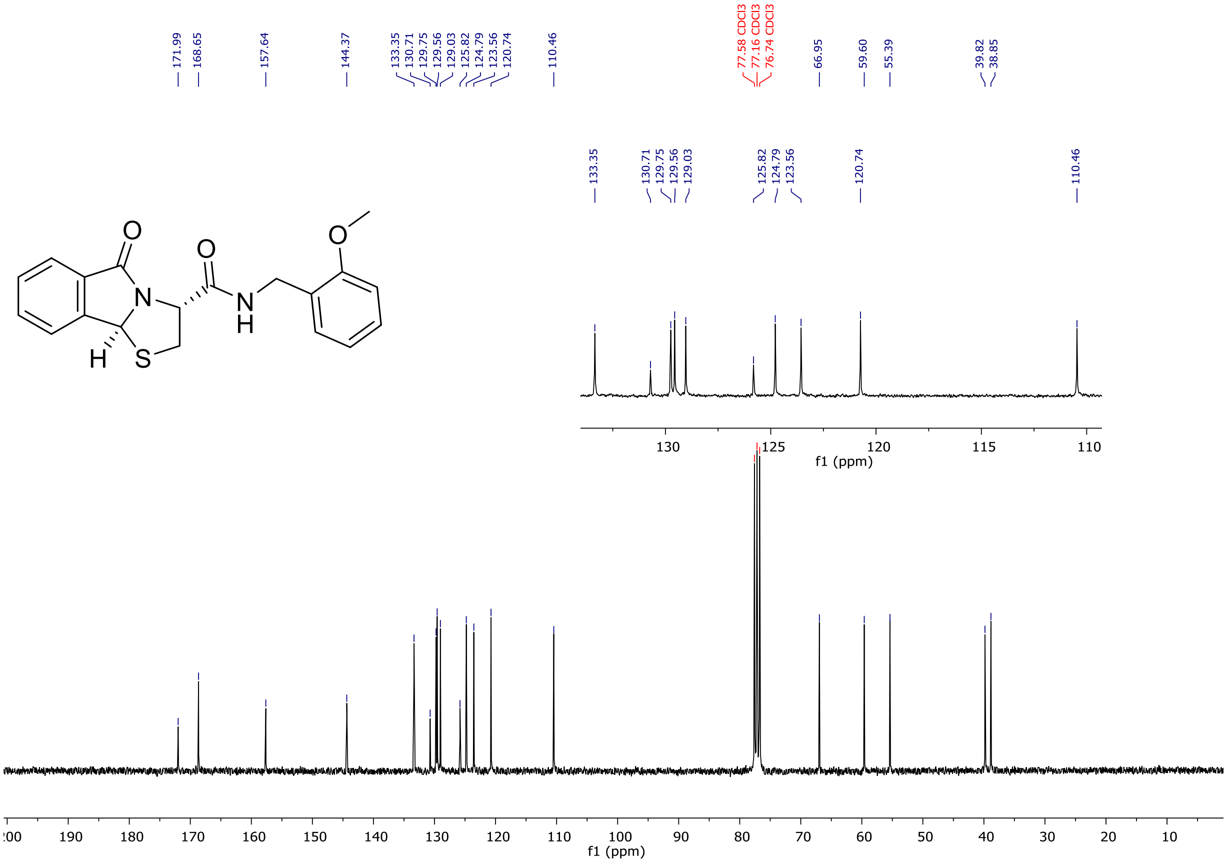

^{13}C NMR spectrum of compound **4aG**

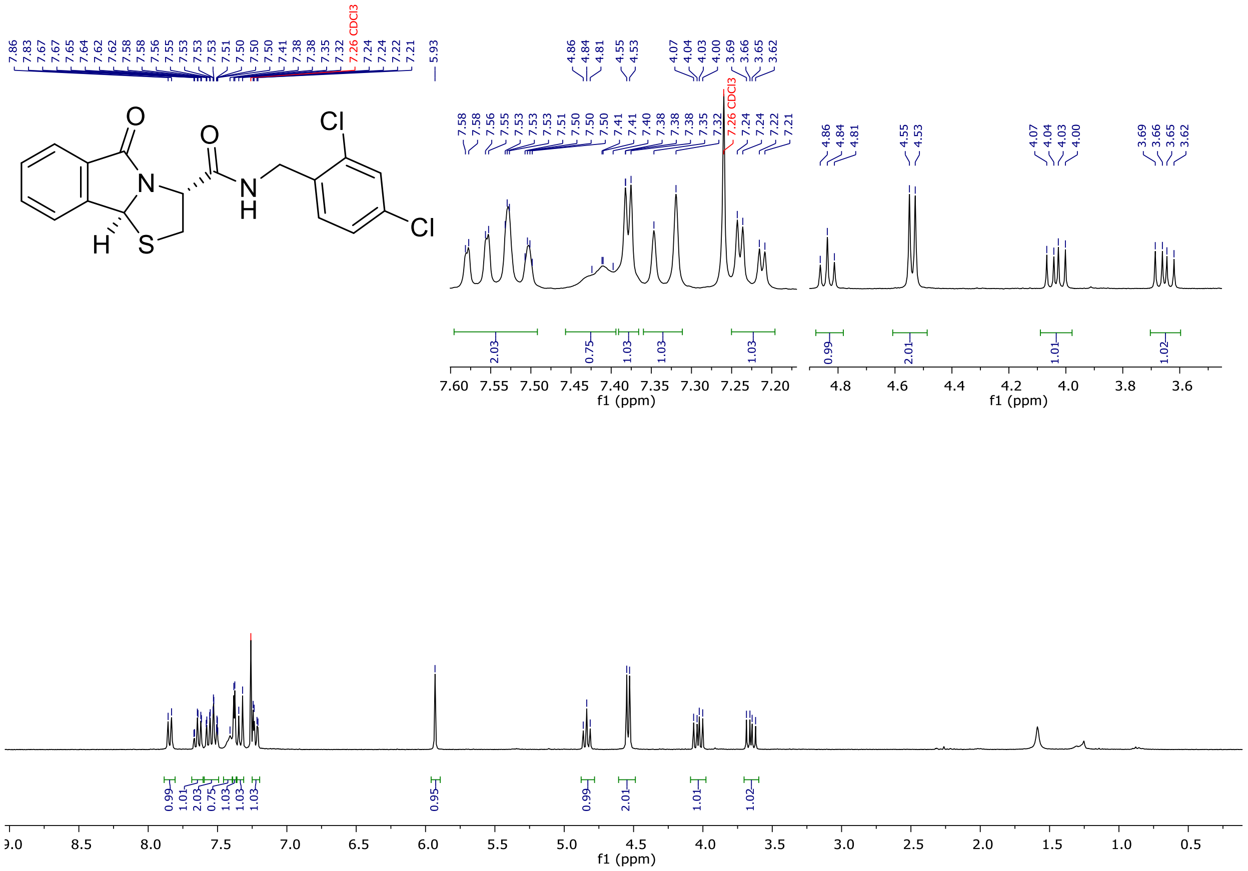

^{1}H NMR spectrum of compound **4aH**

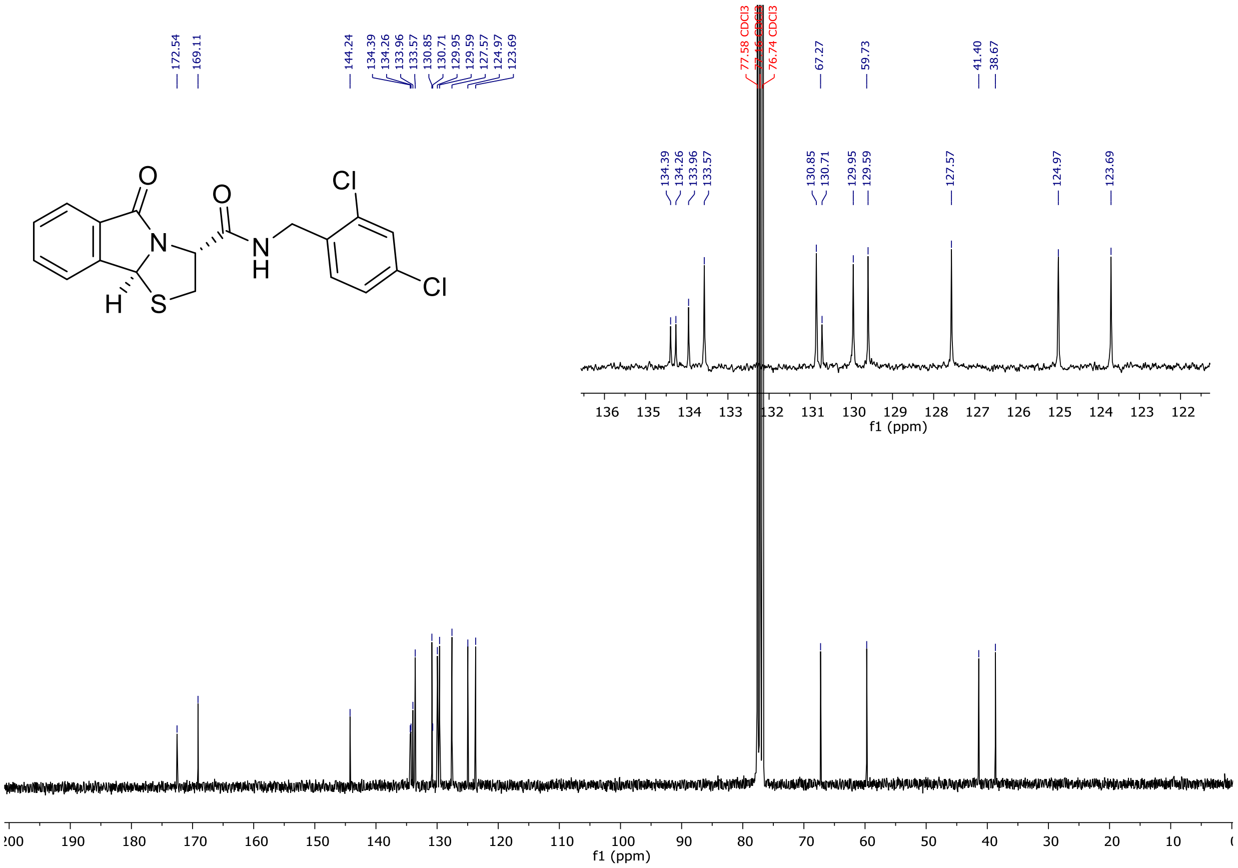

^{13}C NMR spectrum of compound **4aH**

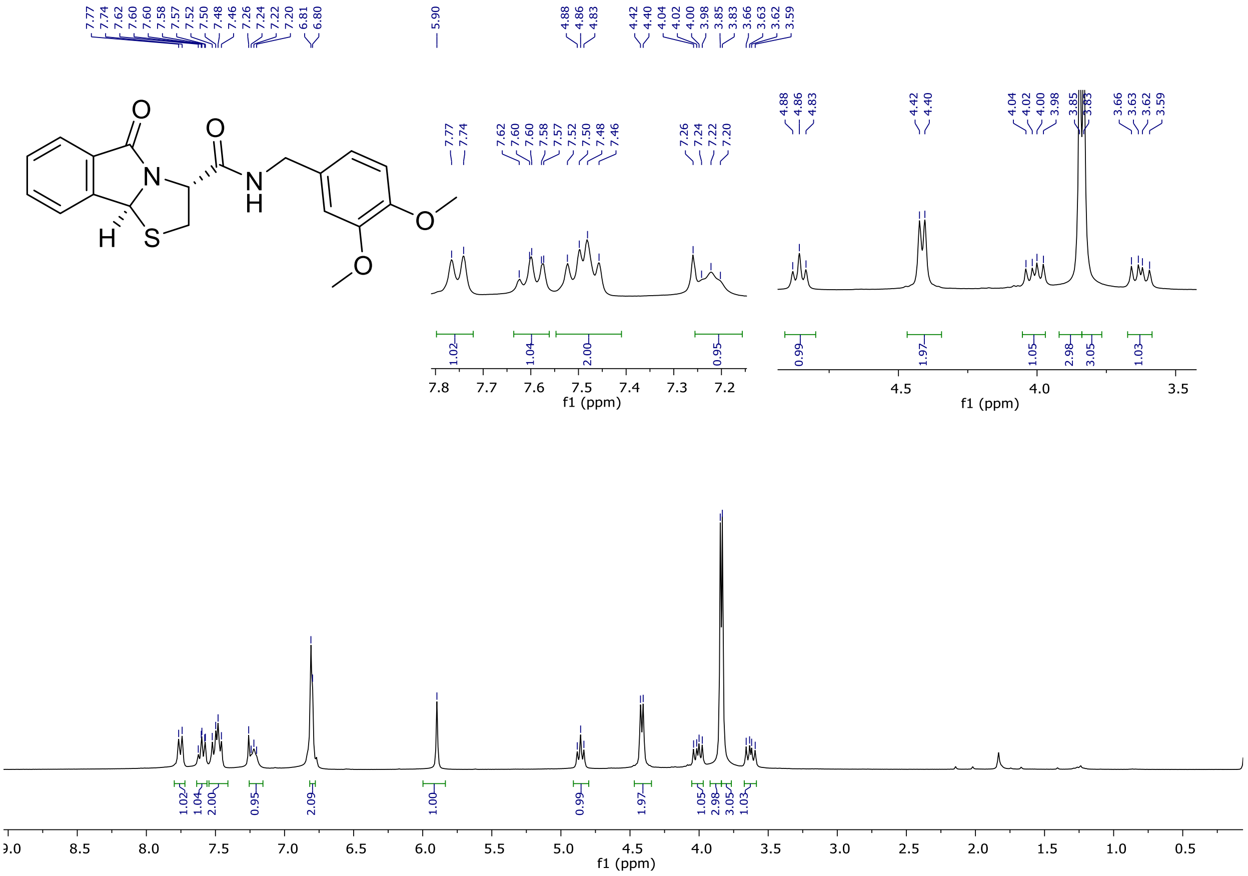

^{1}H NMR spectrum of compound **4al**

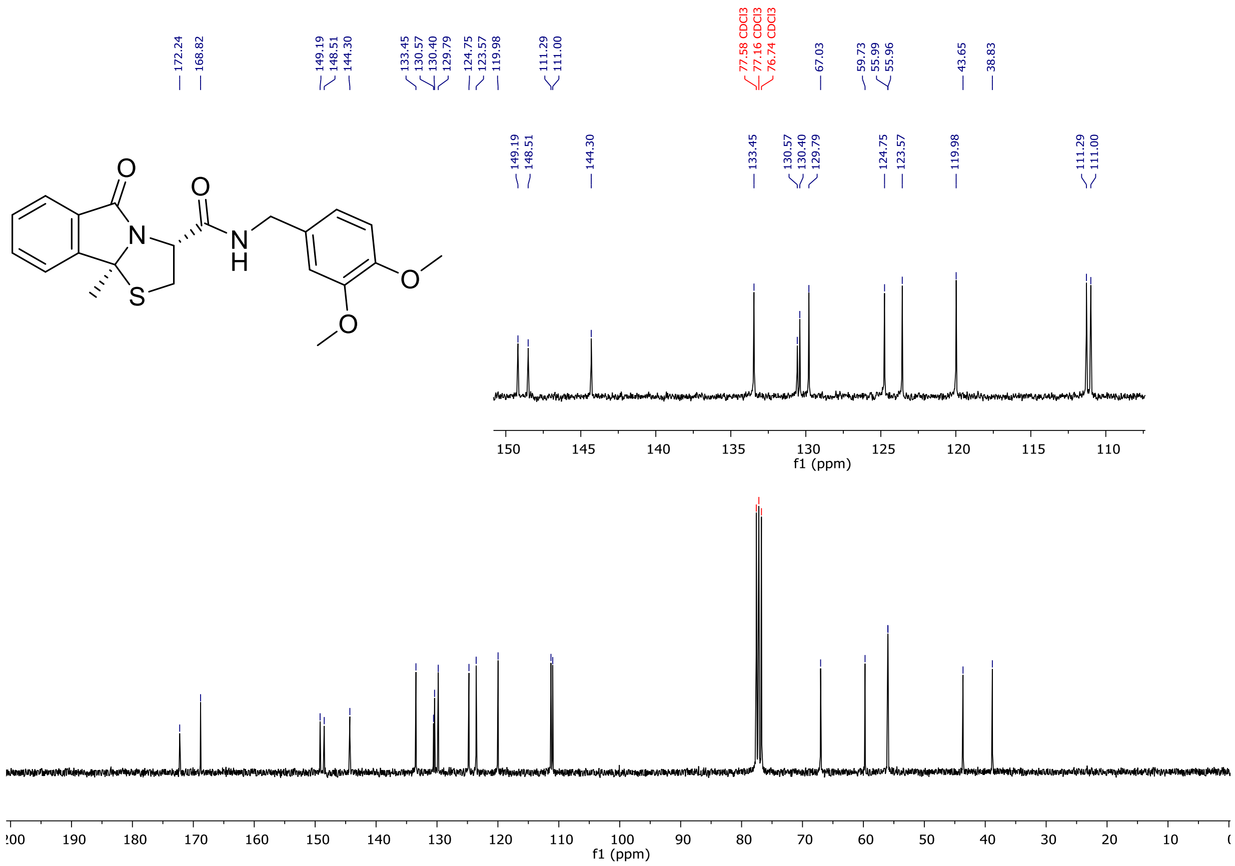

^{13}C NMR spectrum of compound **4al**

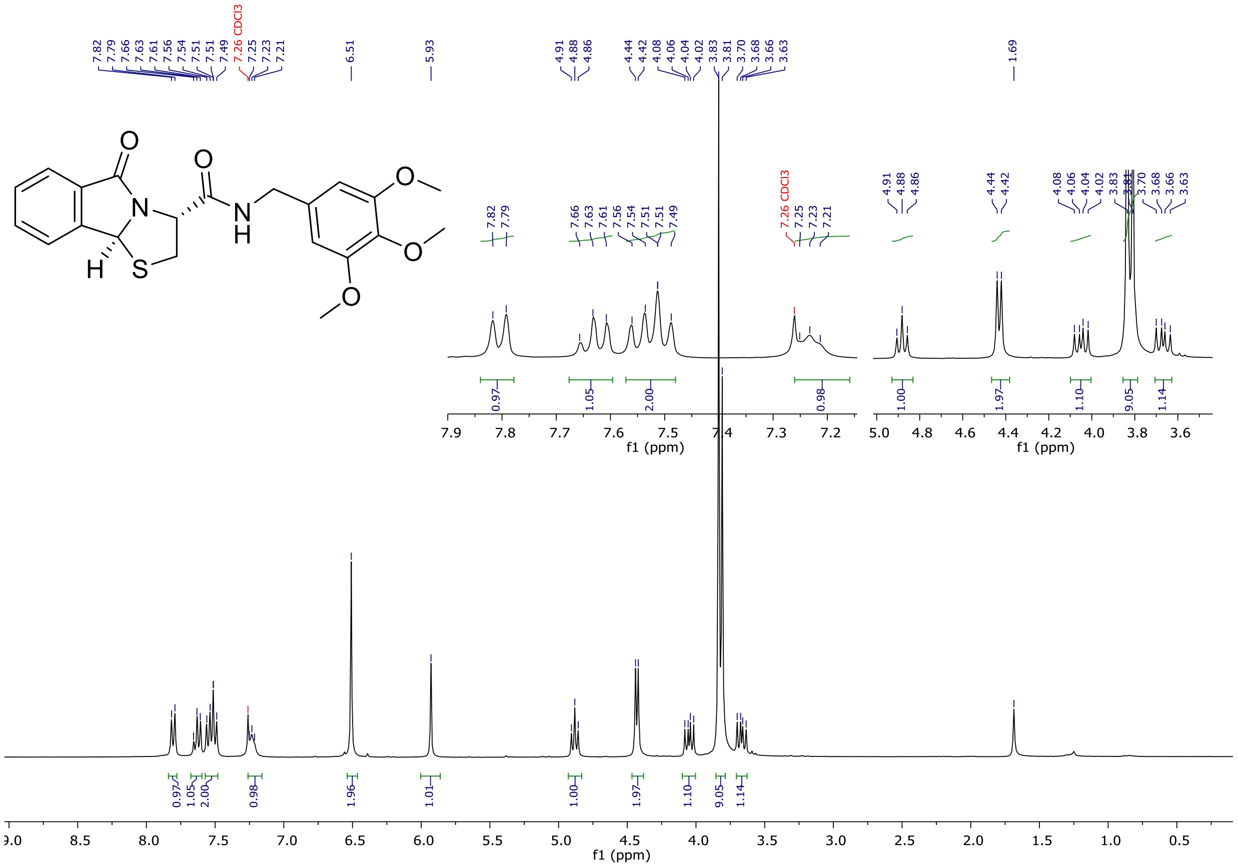

^{1}H NMR spectrum of compound **4aJ**

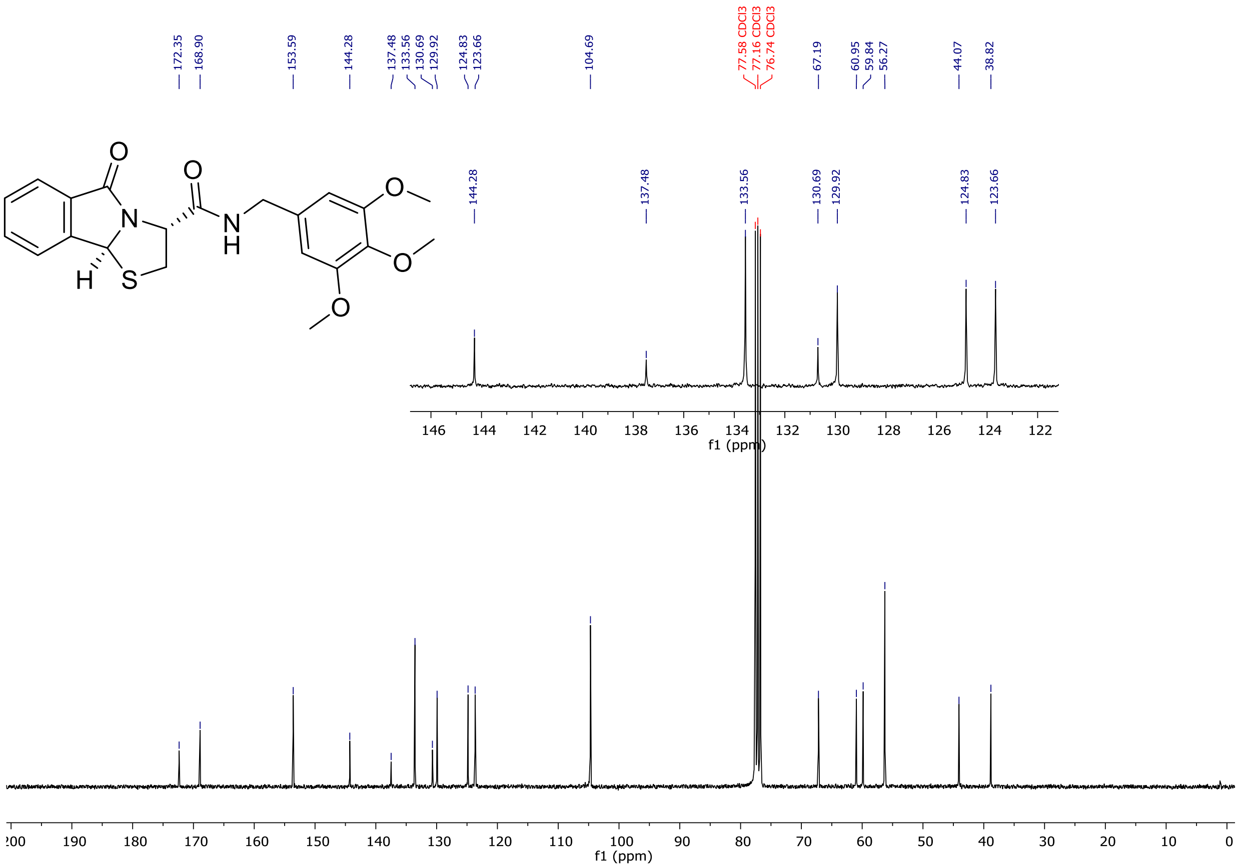

^{13}C NMR spectrum of compound **4aJ**

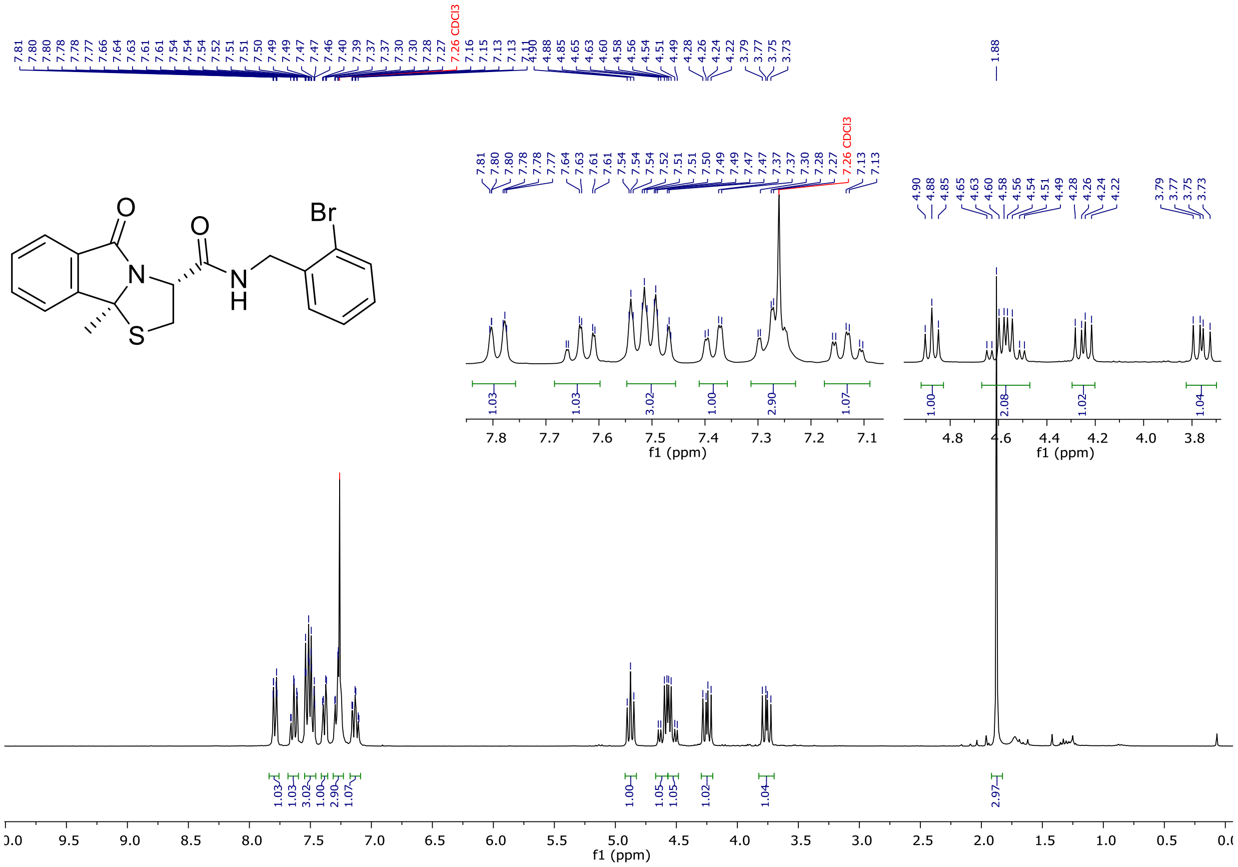

^{1}H NMR spectrum of compound **4bB**

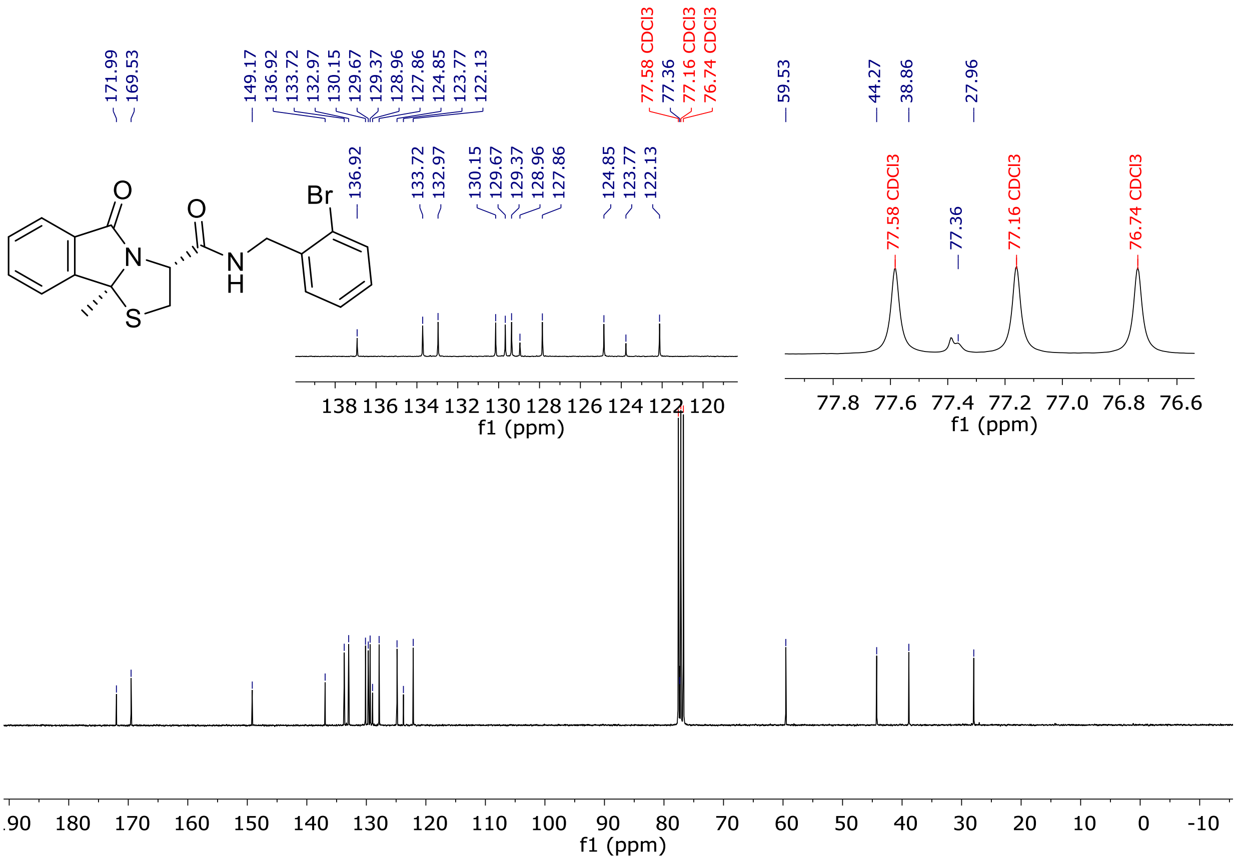

^{13}C NMR spectrum of compound **4bB**

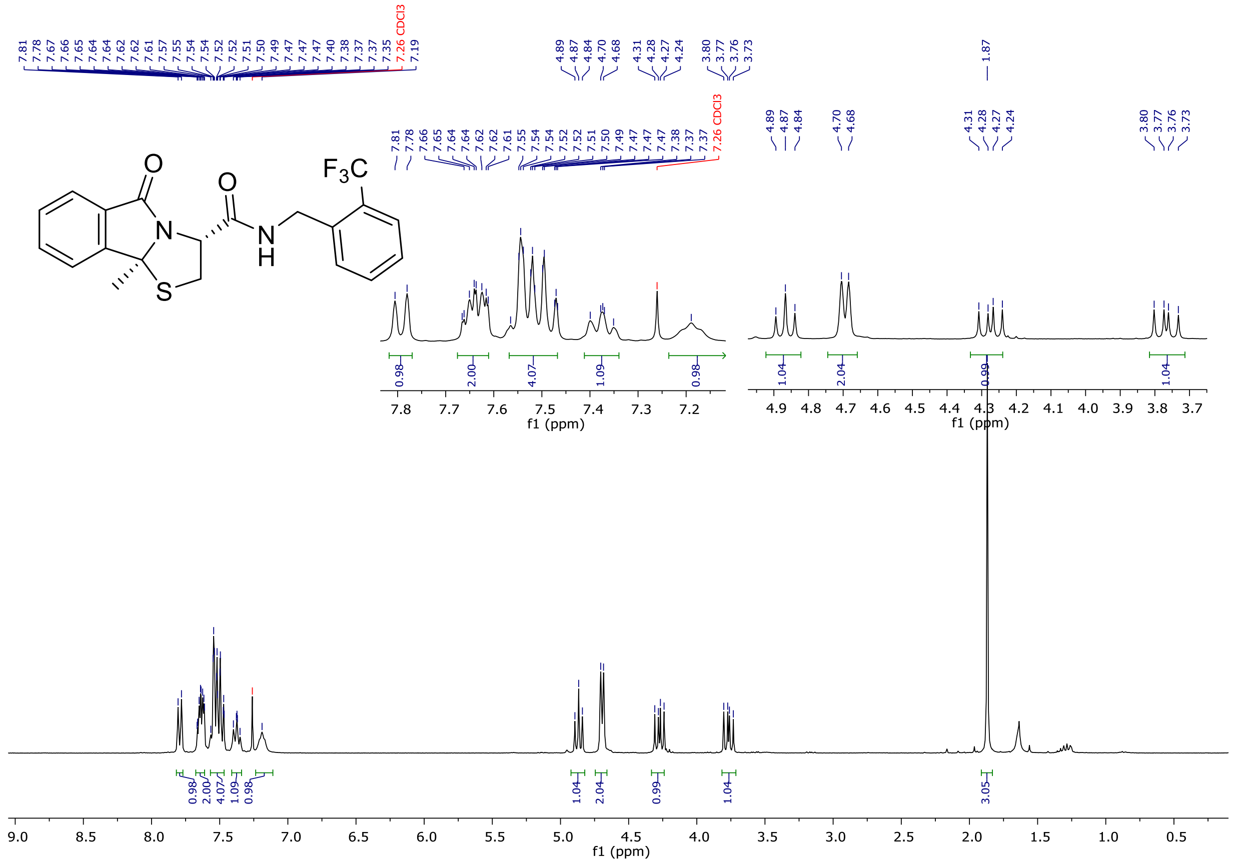

^{1}H NMR spectrum of compound **4bC**

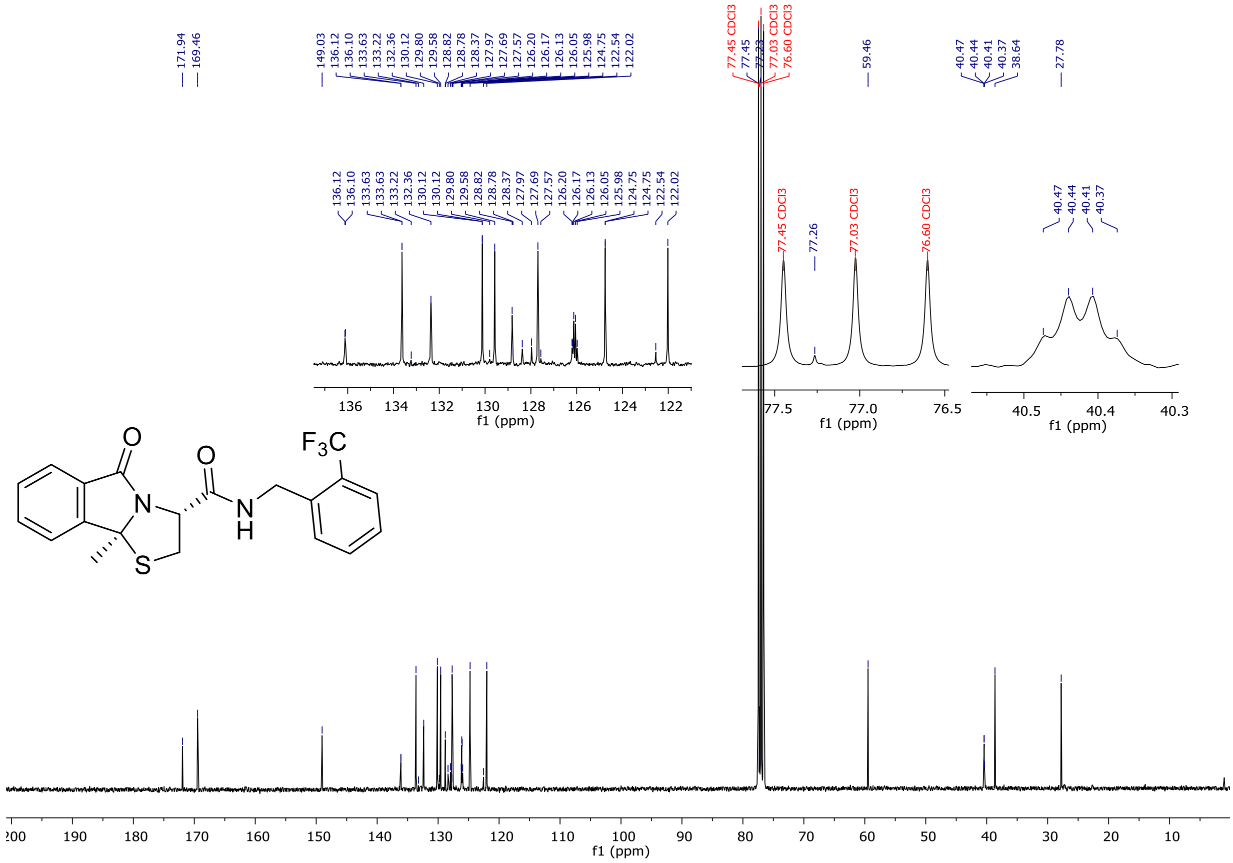

^{13}C NMR spectrum of compound **4bC**

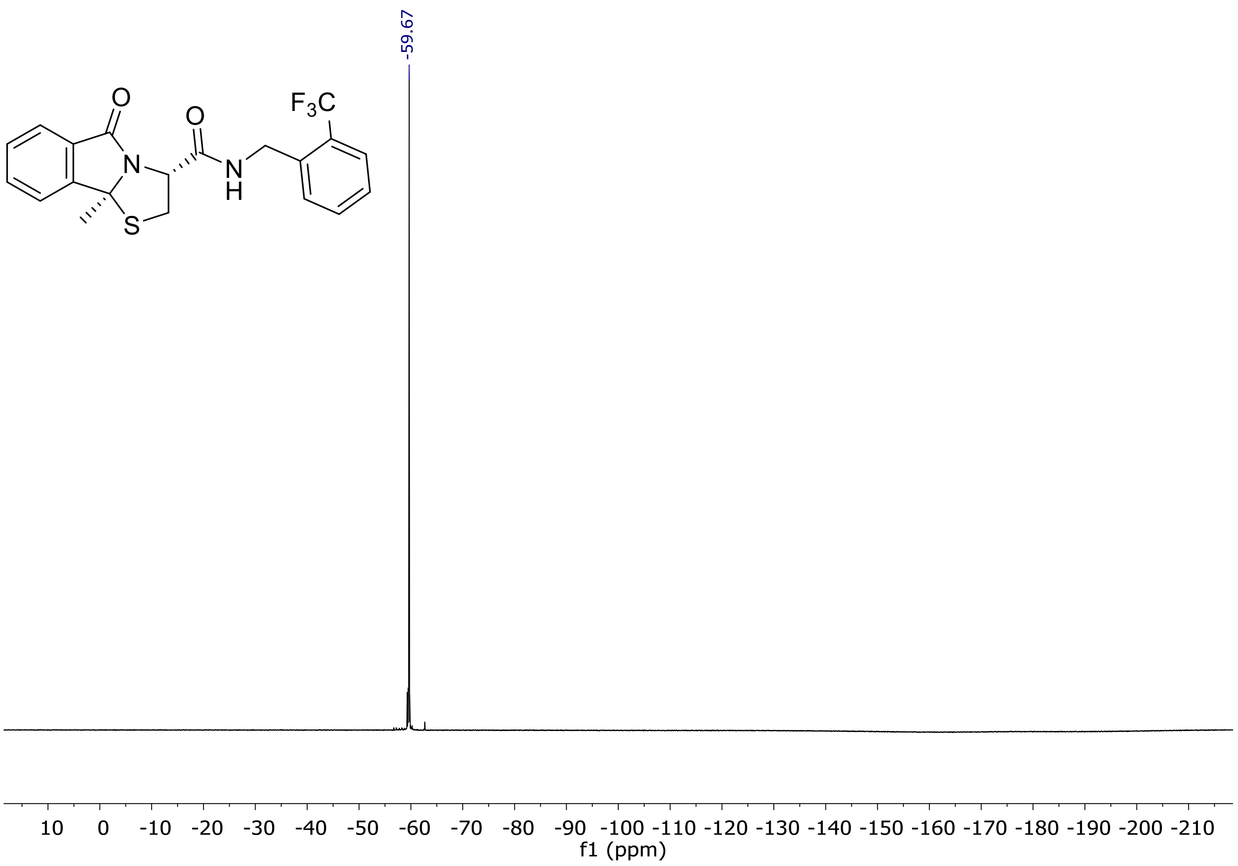

^{19}F NMR spectrum of compound **4bC**

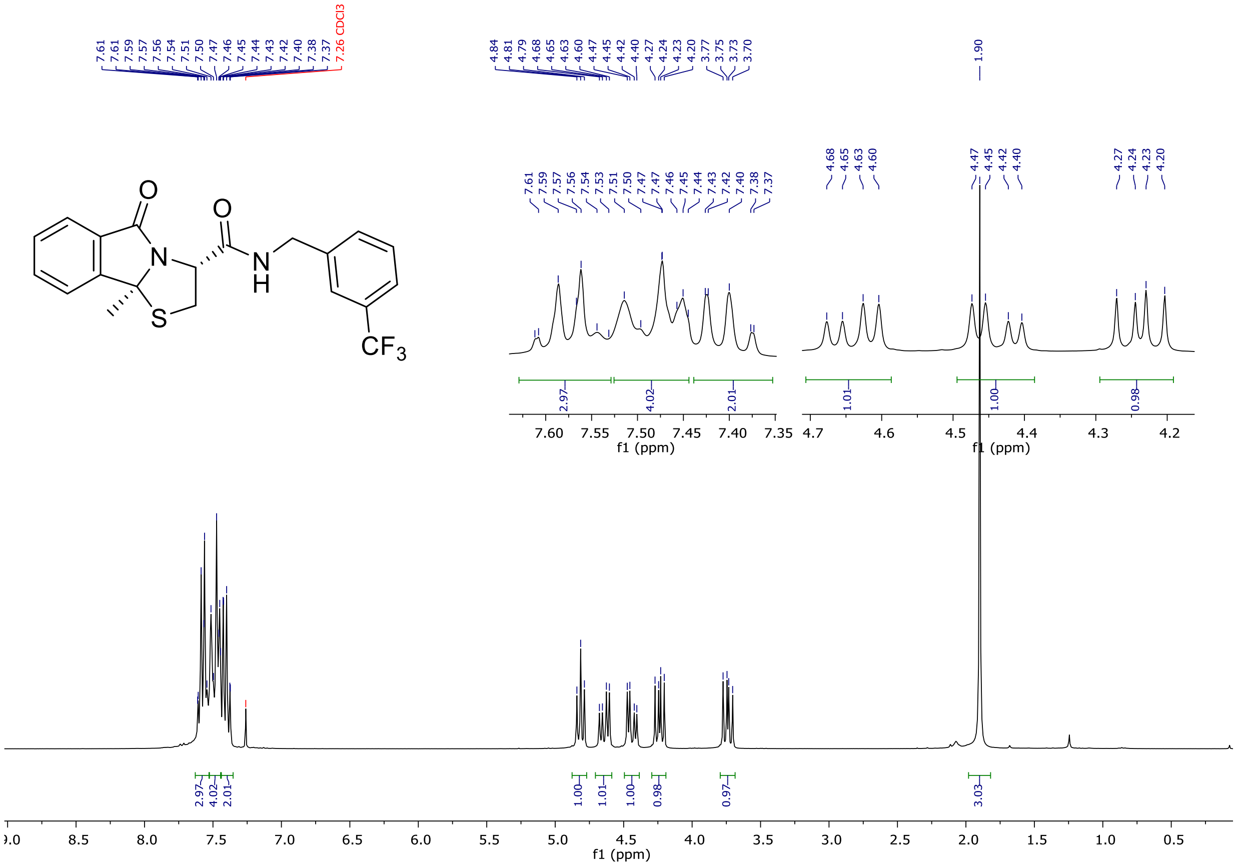

^{1}H NMR spectrum of compound **4bD**

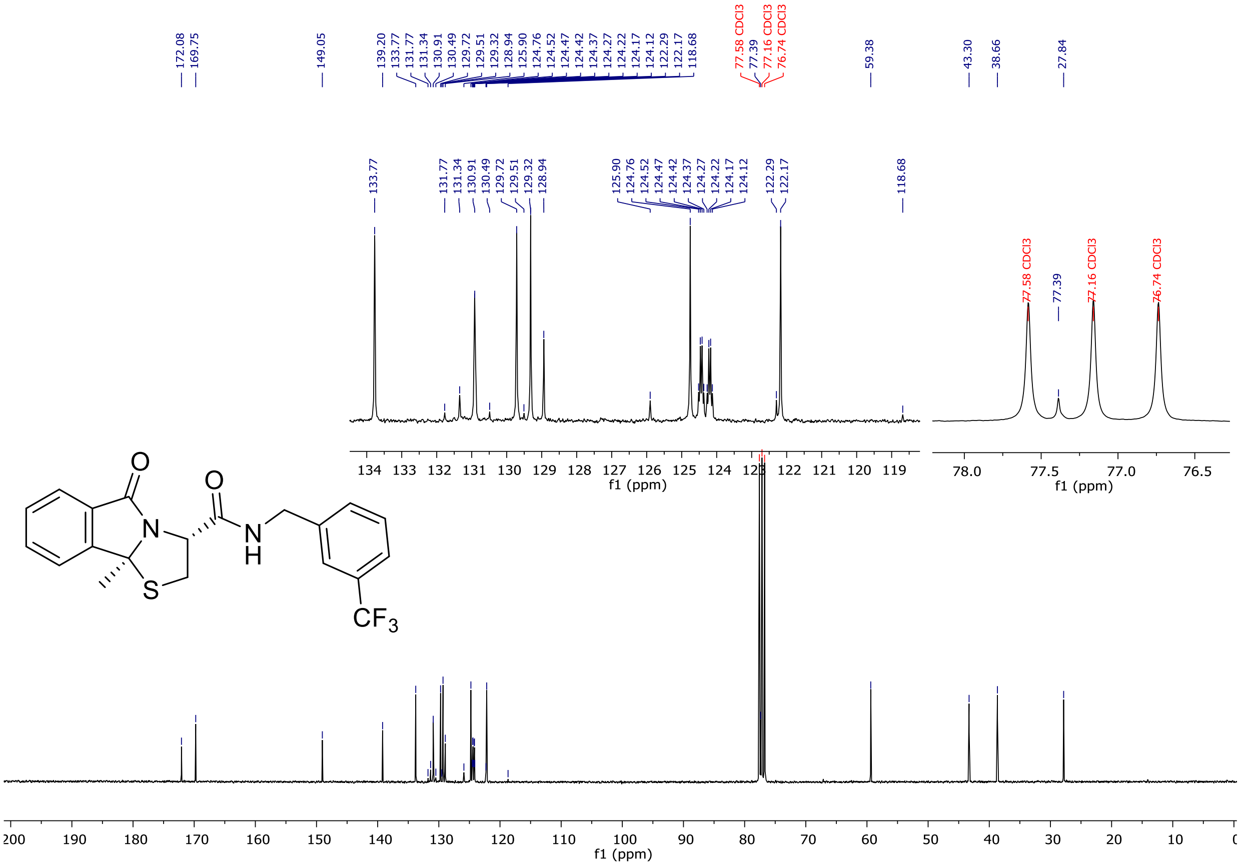

^{13}C NMR spectrum of compound **4bD**

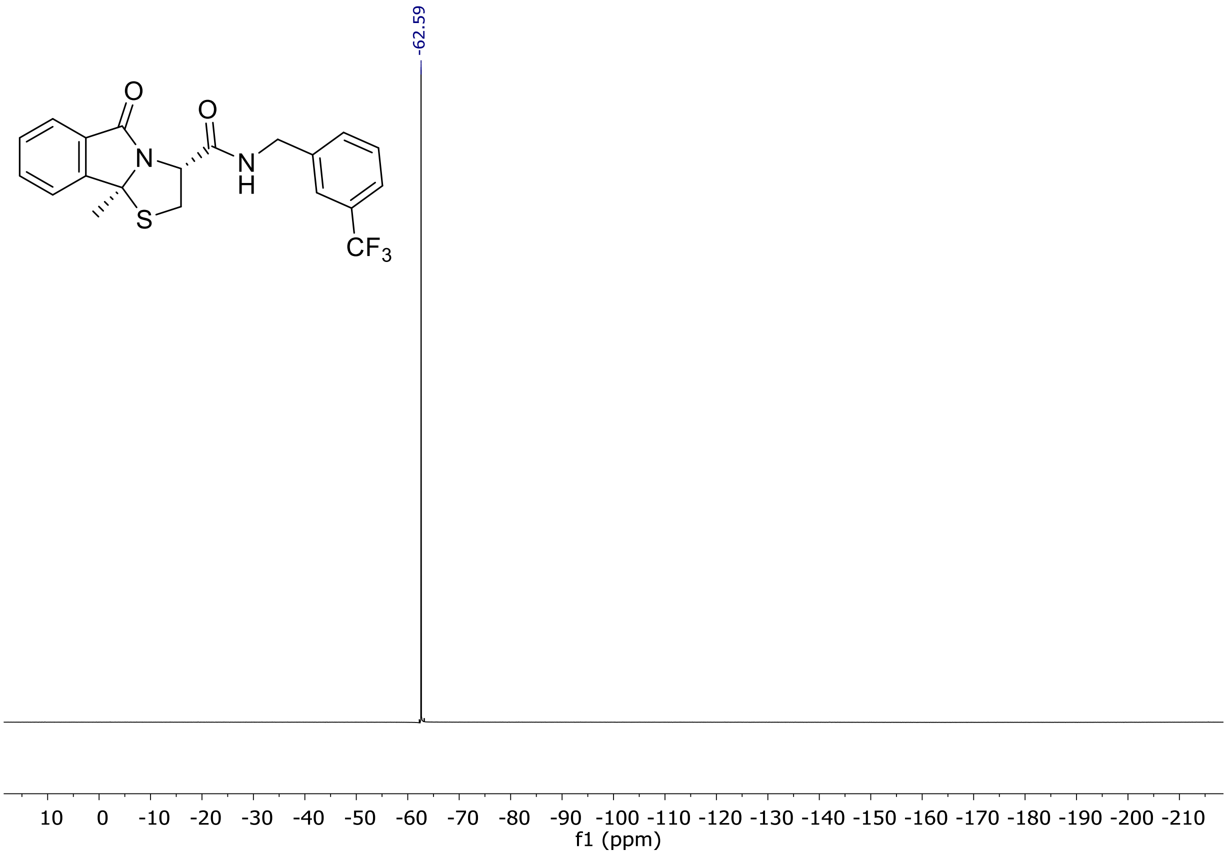

^{19}F NMR spectrum of compound **4bD**

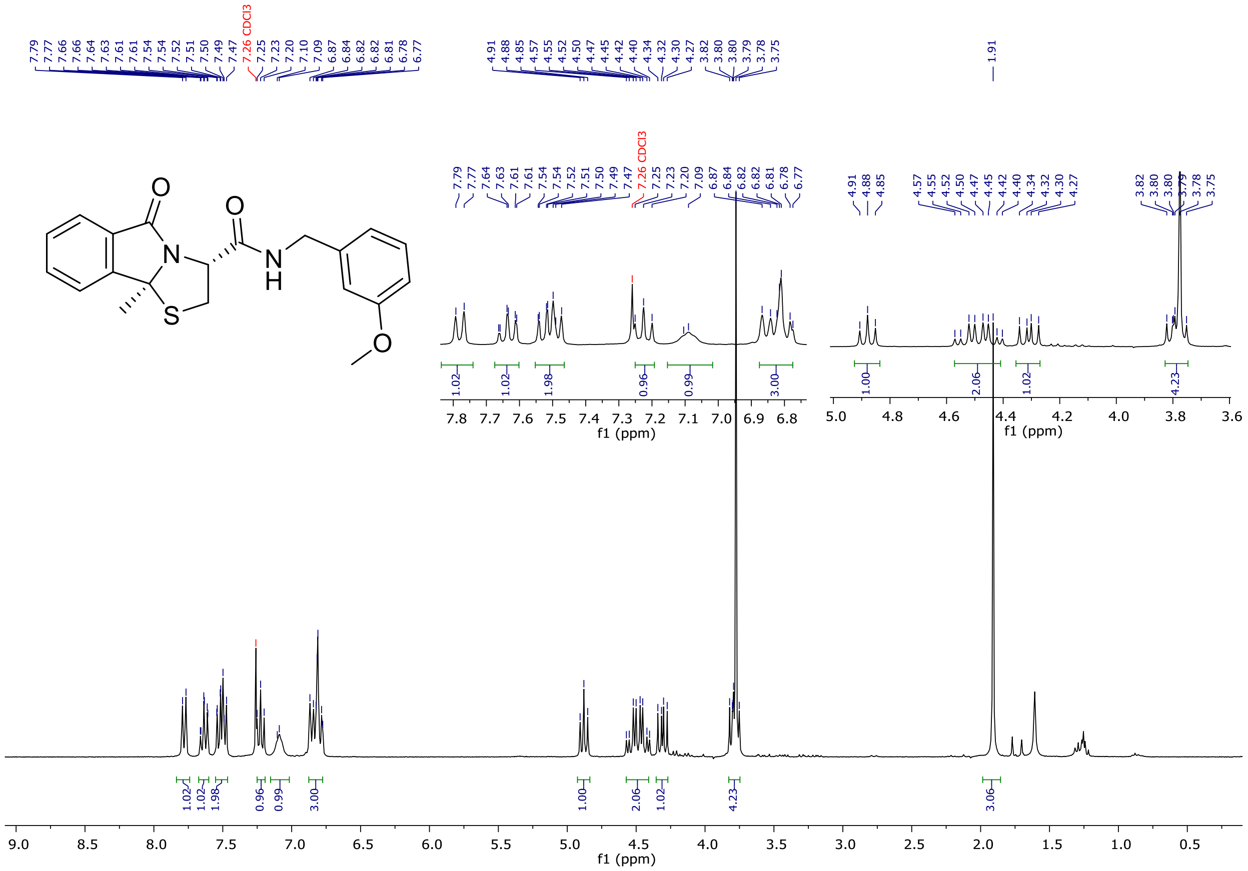

^{1}H NMR spectrum of compound **4bE**

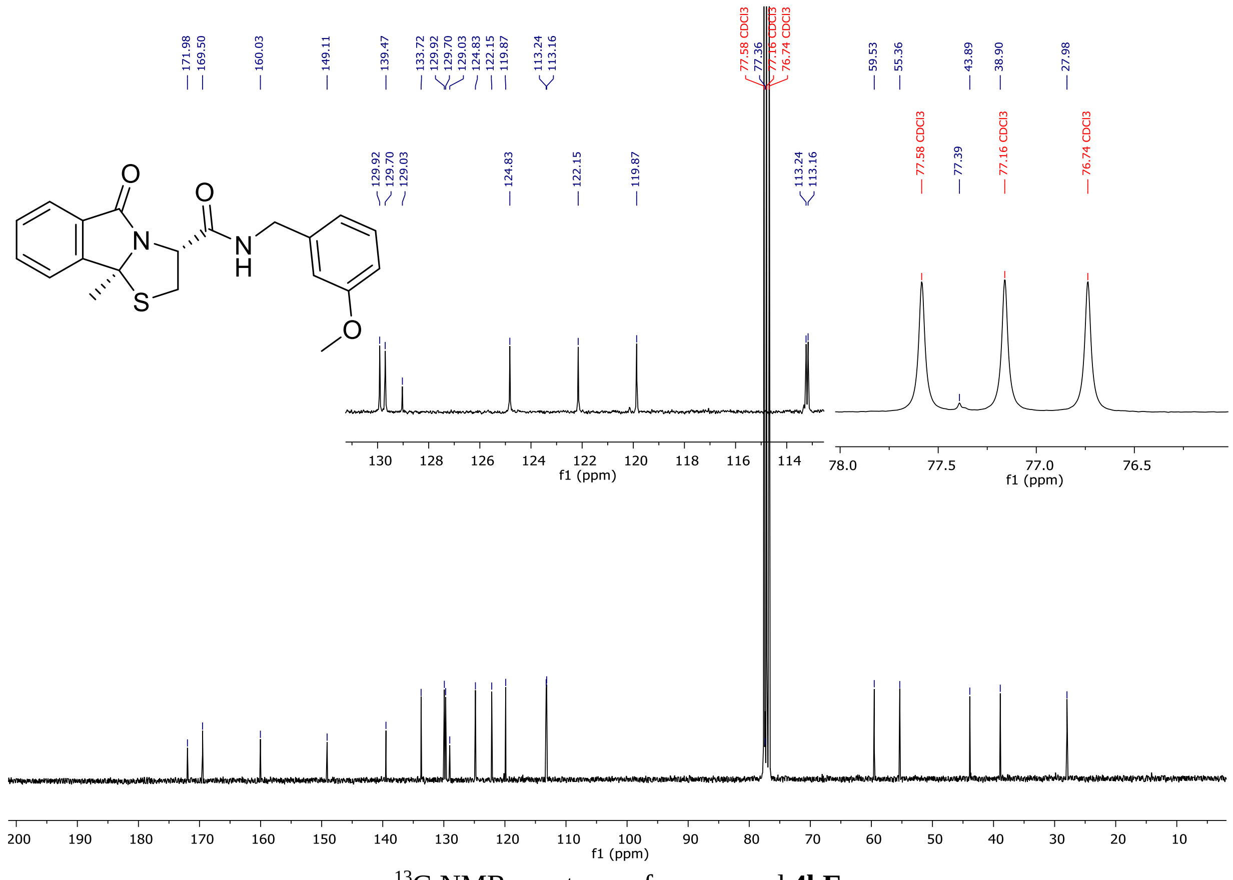

^{13}C NMR spectrum of compound **4bE**

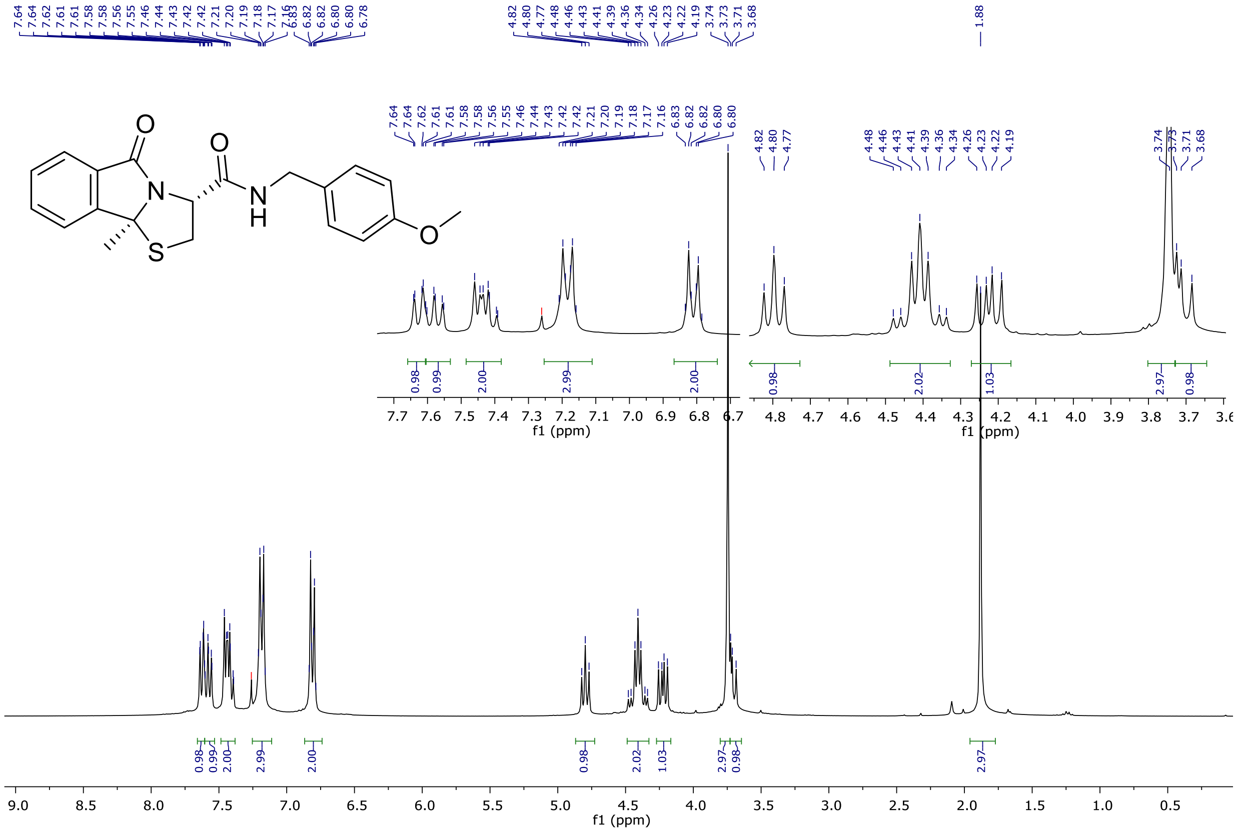

^{1}H NMR spectrum of compound **4bF**

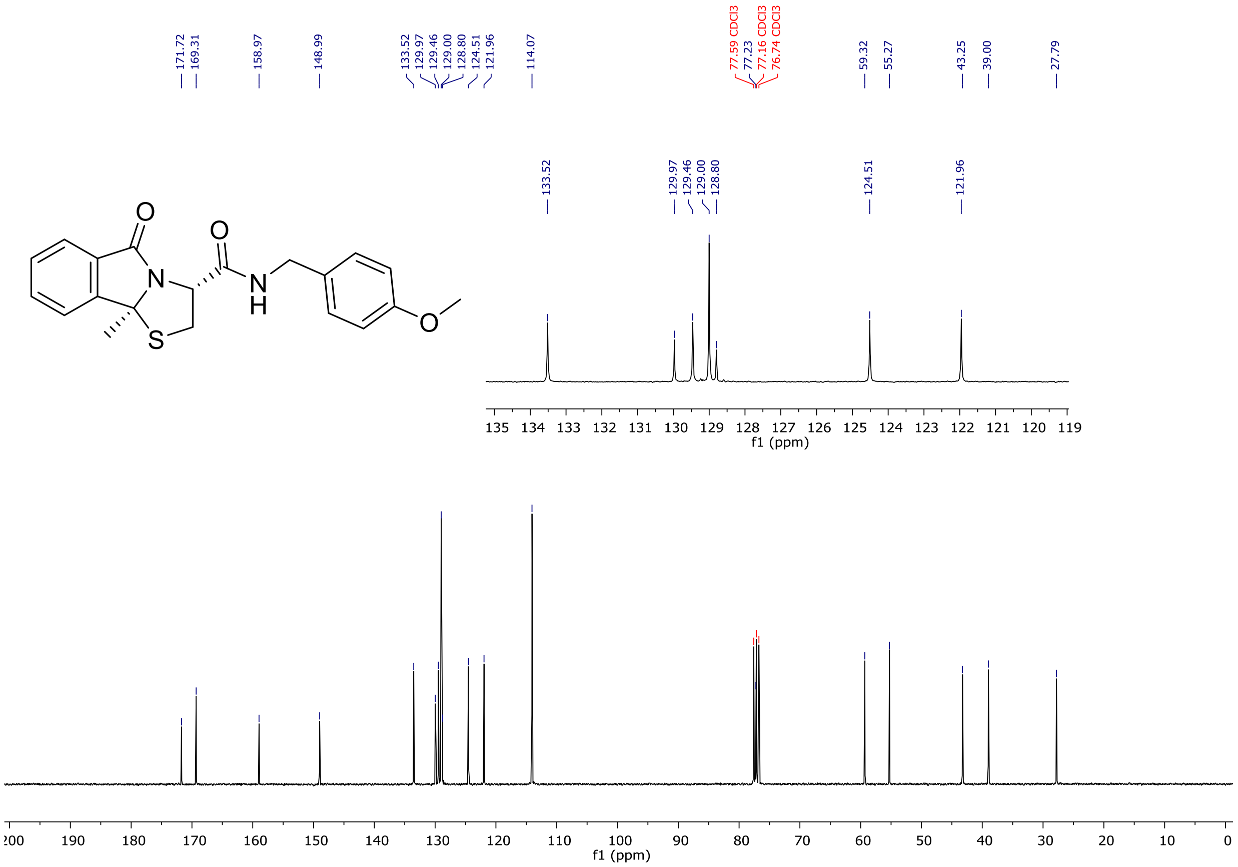

^{13}C NMR spectrum of compound **4bF**

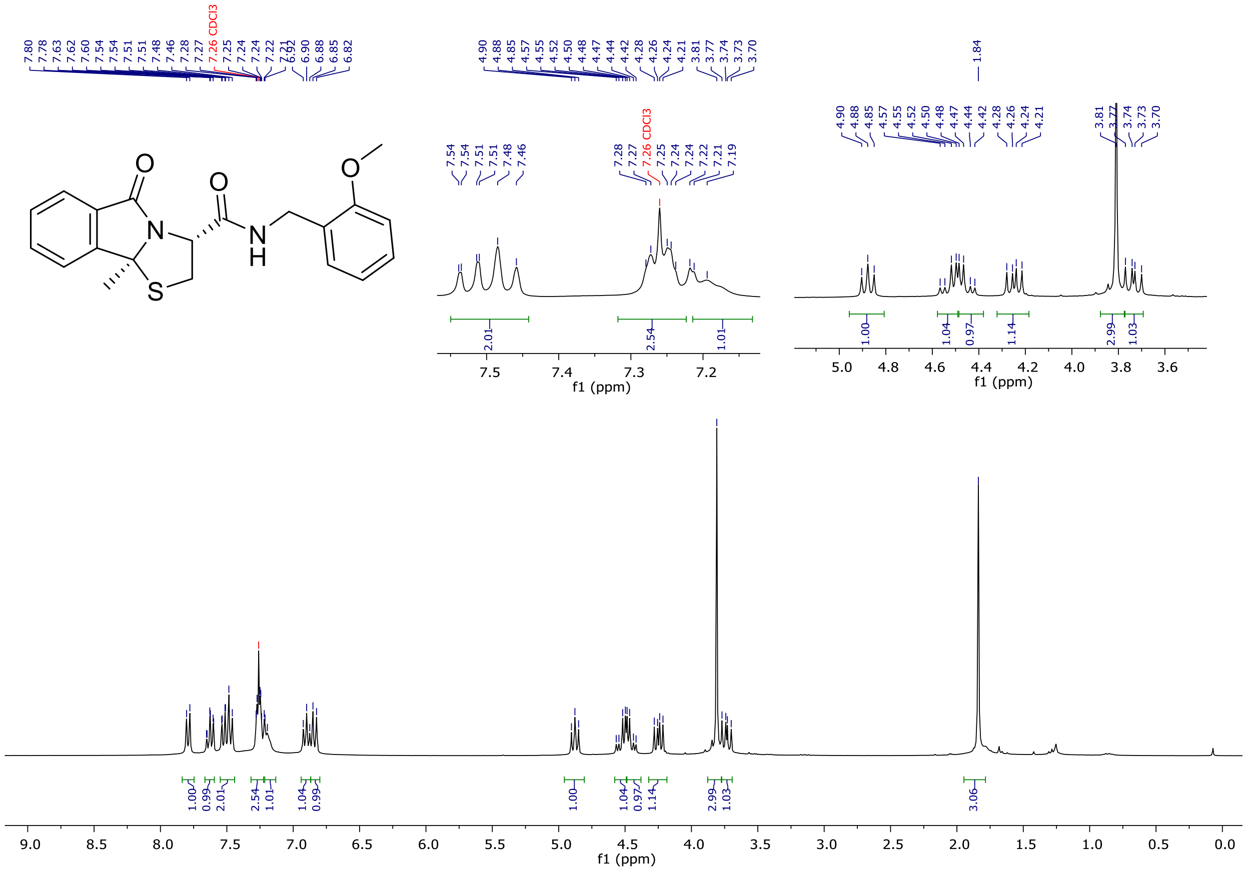

^{1}H NMR spectrum of compound **4bG**

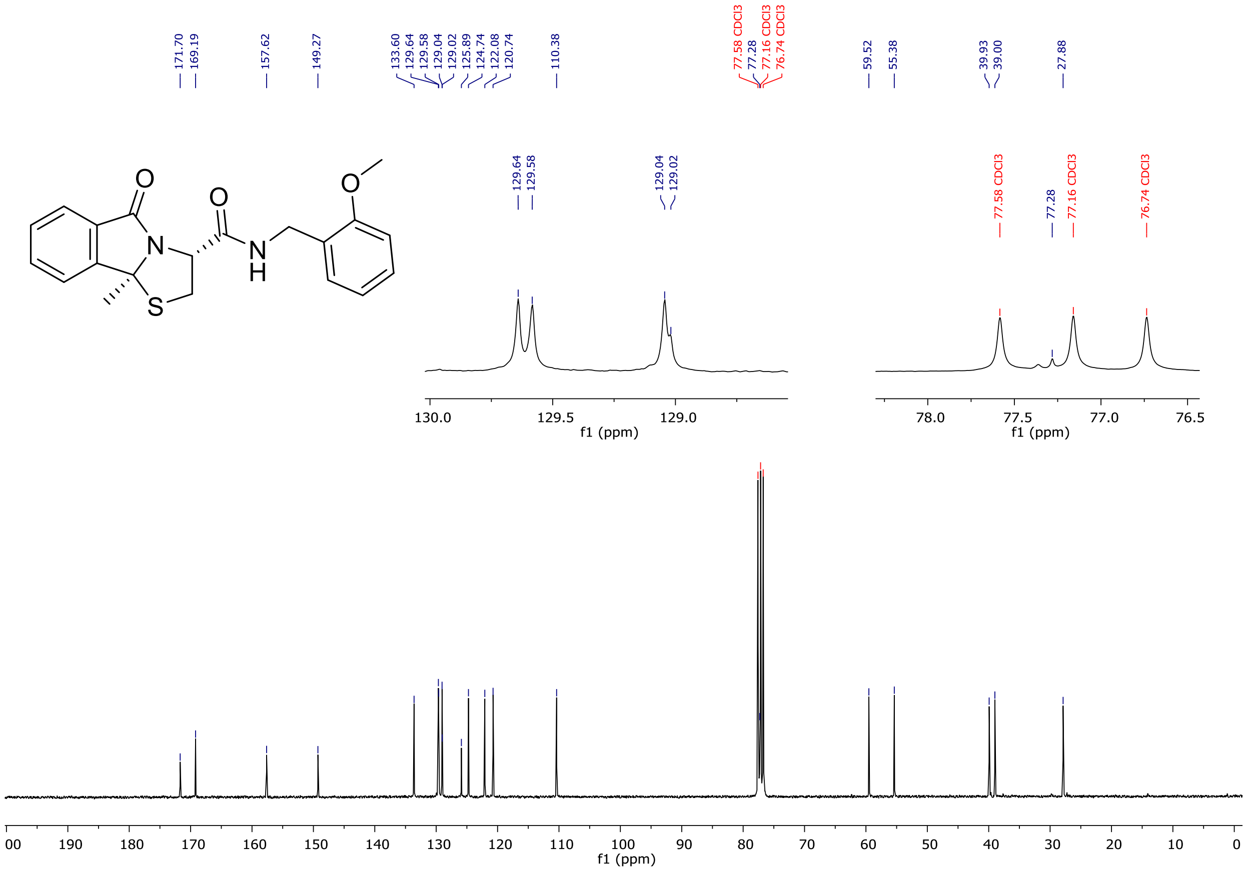

^{13}C NMR spectrum of compound **4bG**

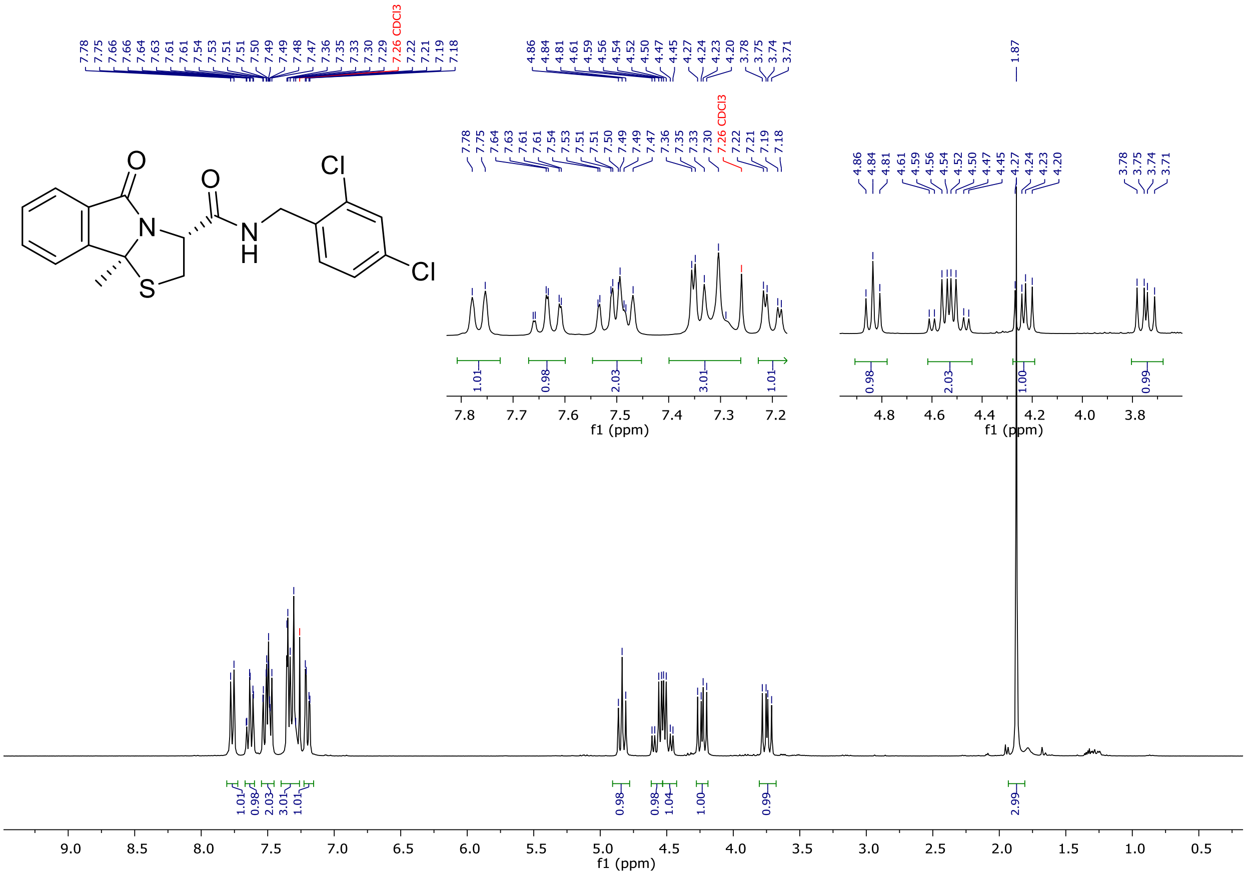

^{1}H NMR spectrum of compound **4bH**

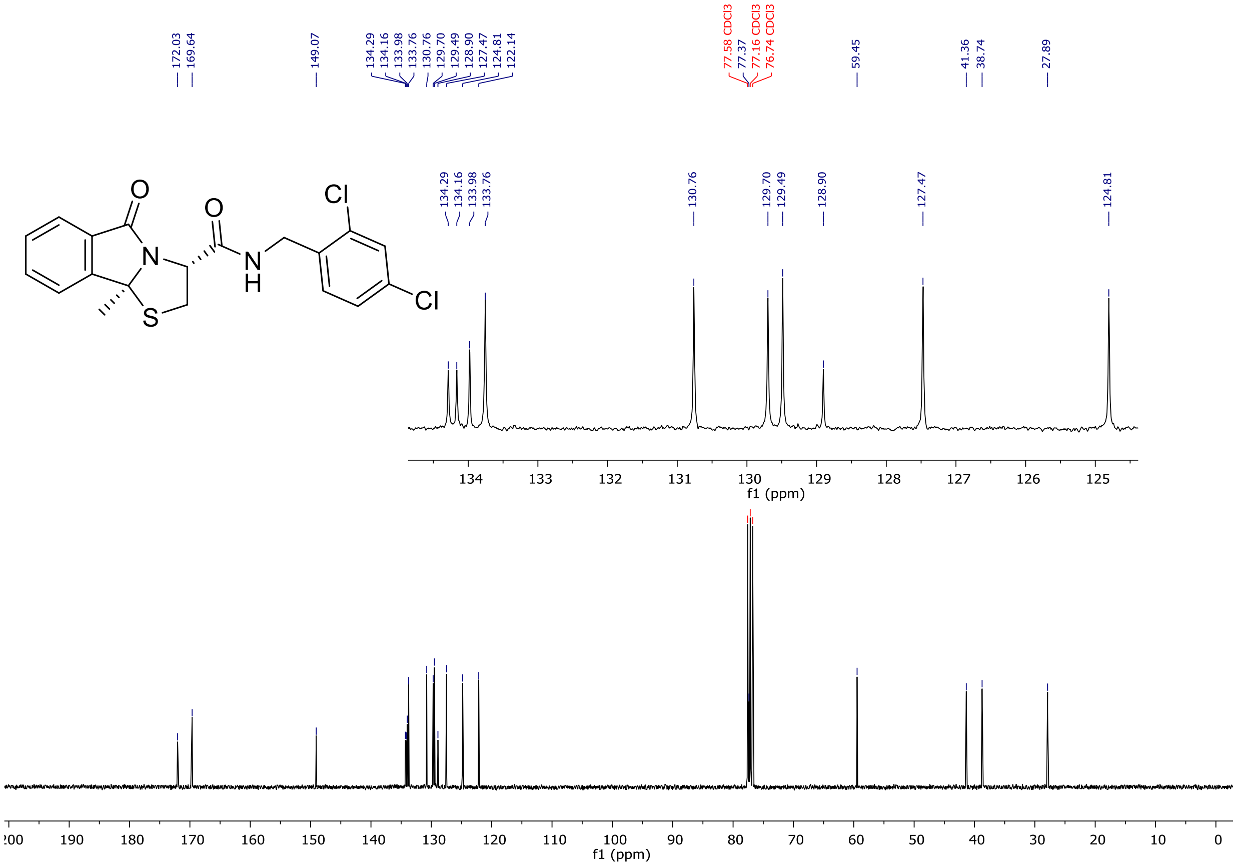

^{13}C NMR spectrum of compound **4bH**

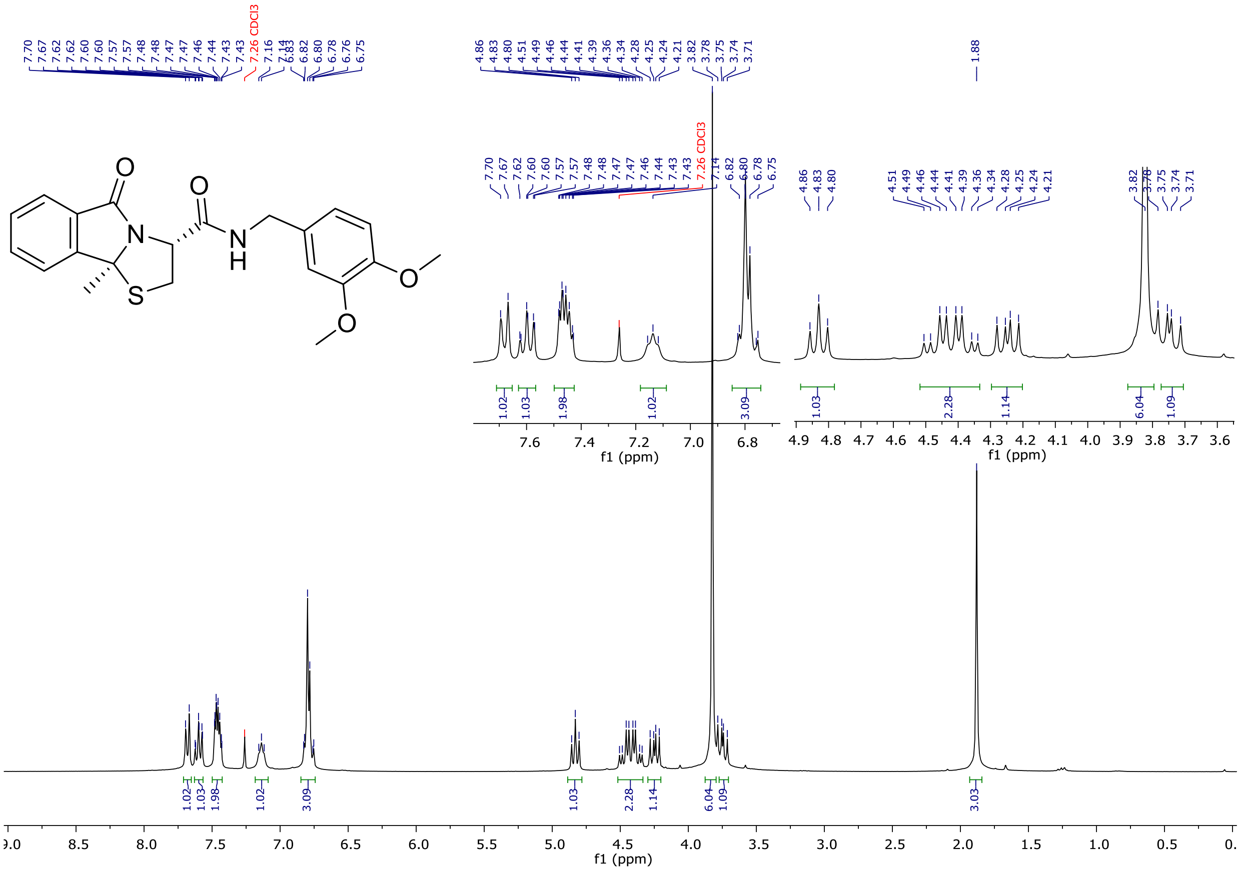

^{1}H NMR spectrum of compound **4bI**

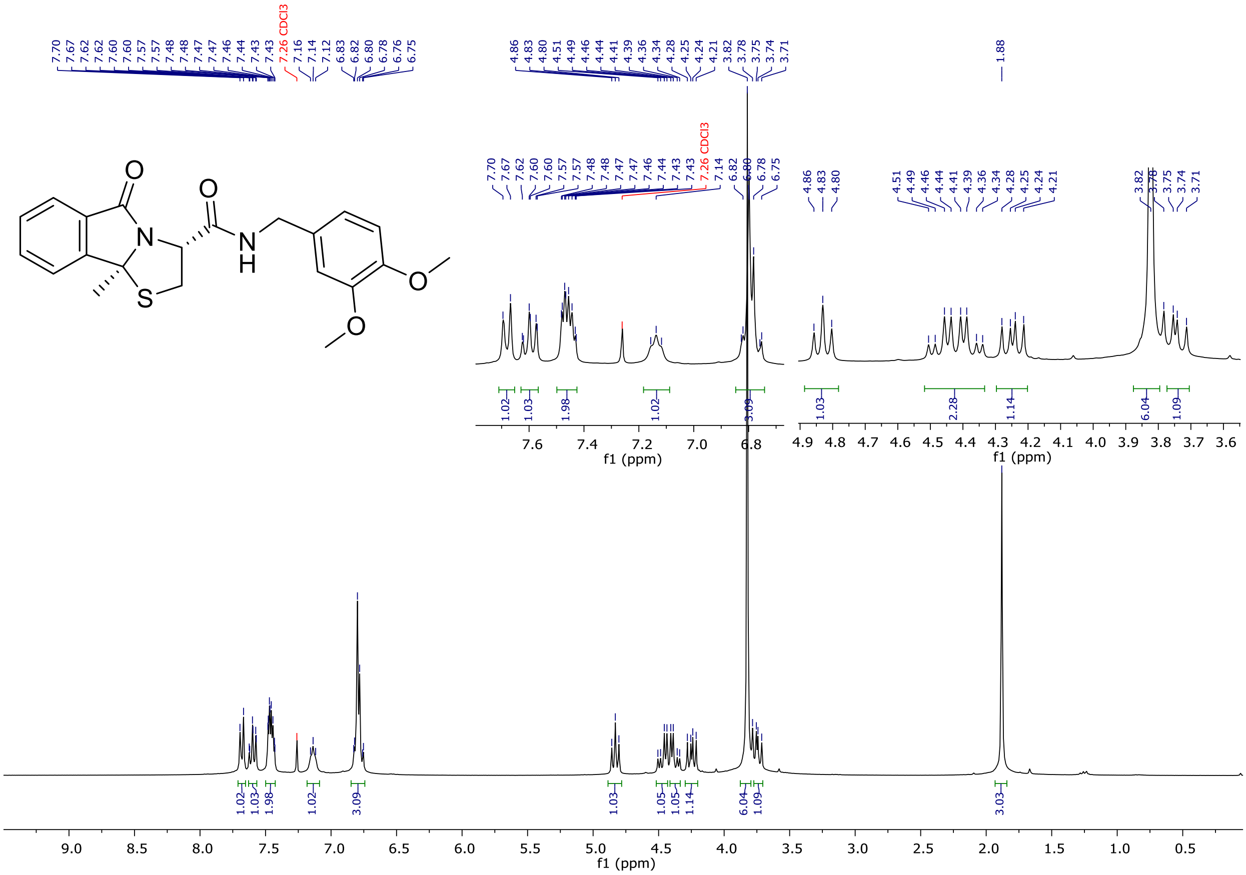

^{13}C NMR spectrum of compound **4bI**

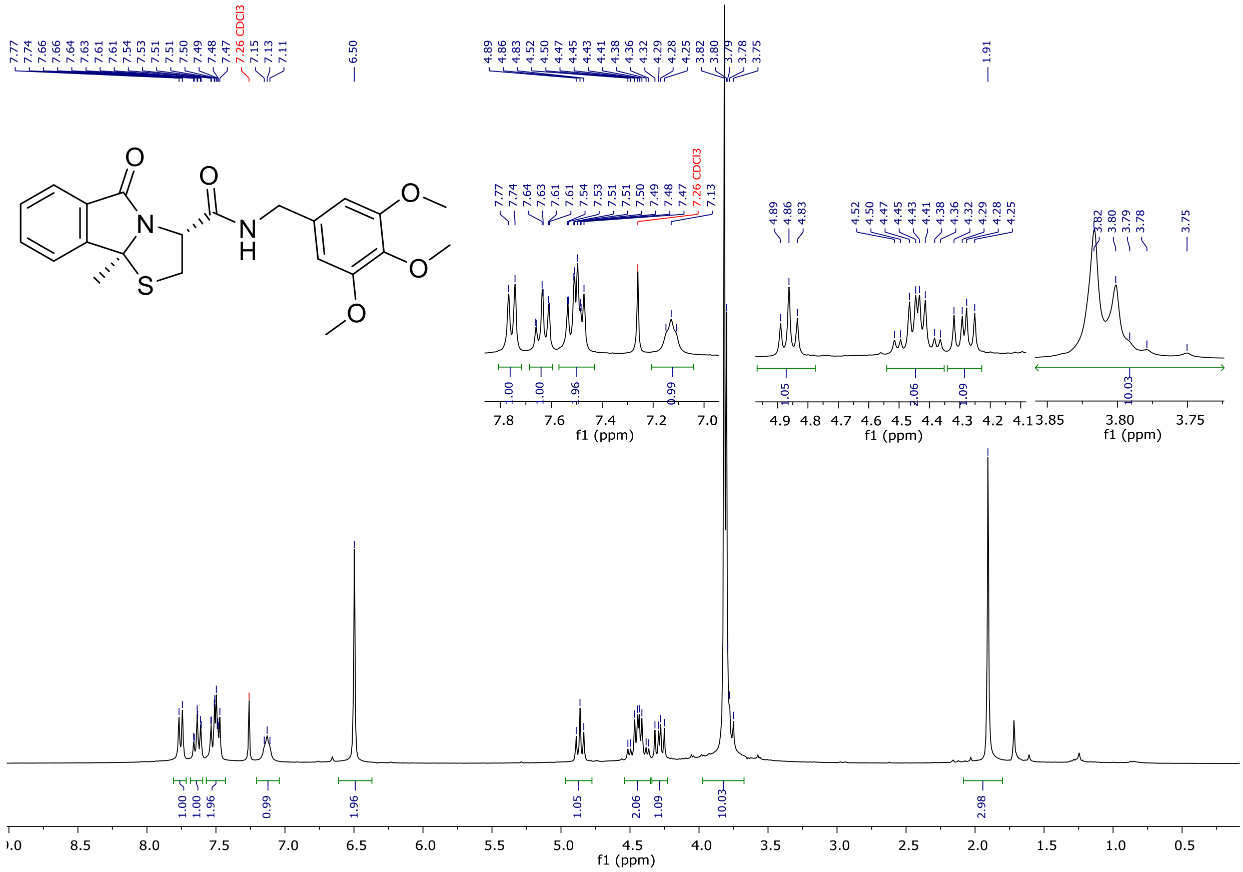

^{1}H NMR spectrum of compound **4bJ**

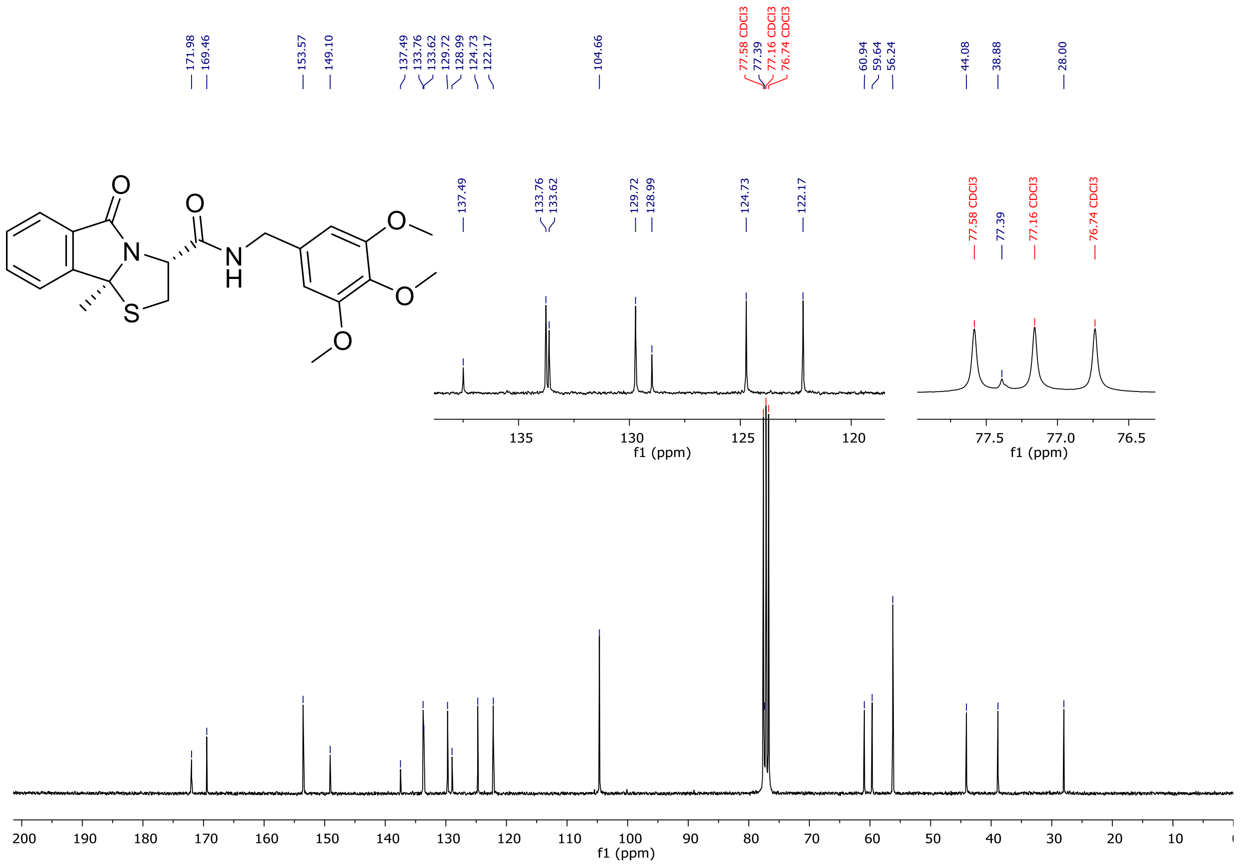

^{13}C NMR spectrum of compound **4bJ**

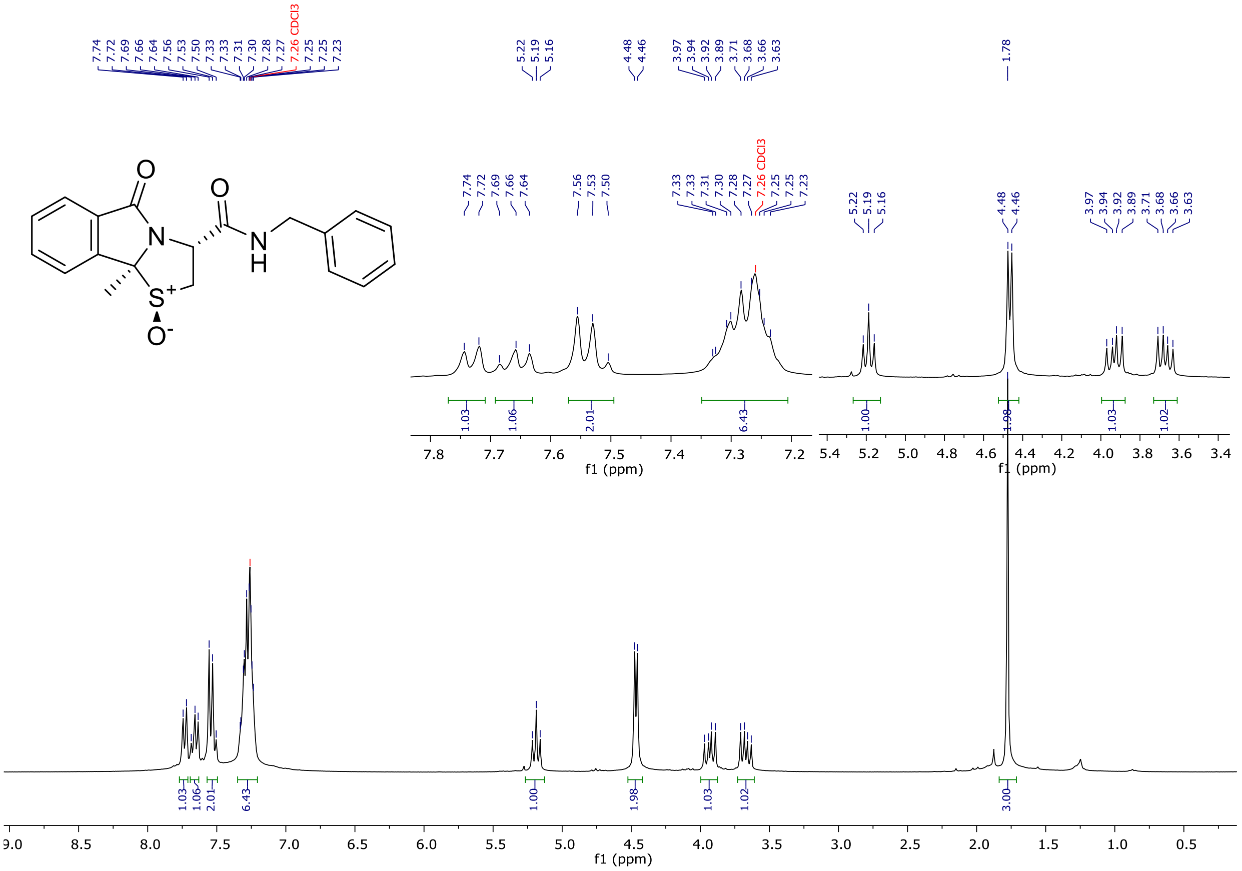

^{1}H NMR spectrum of compound **5bA**

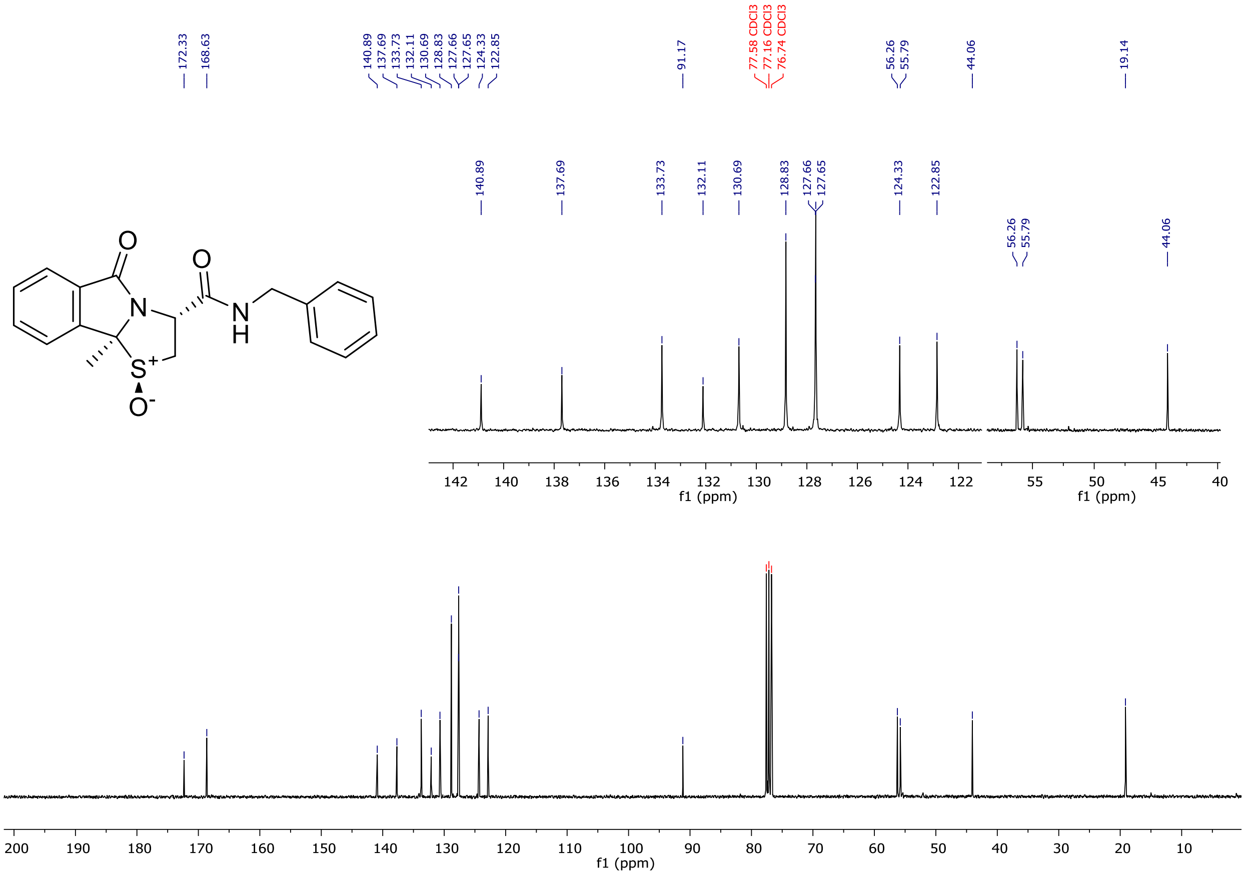

^{13}C NMR spectrum of compound **5bA**

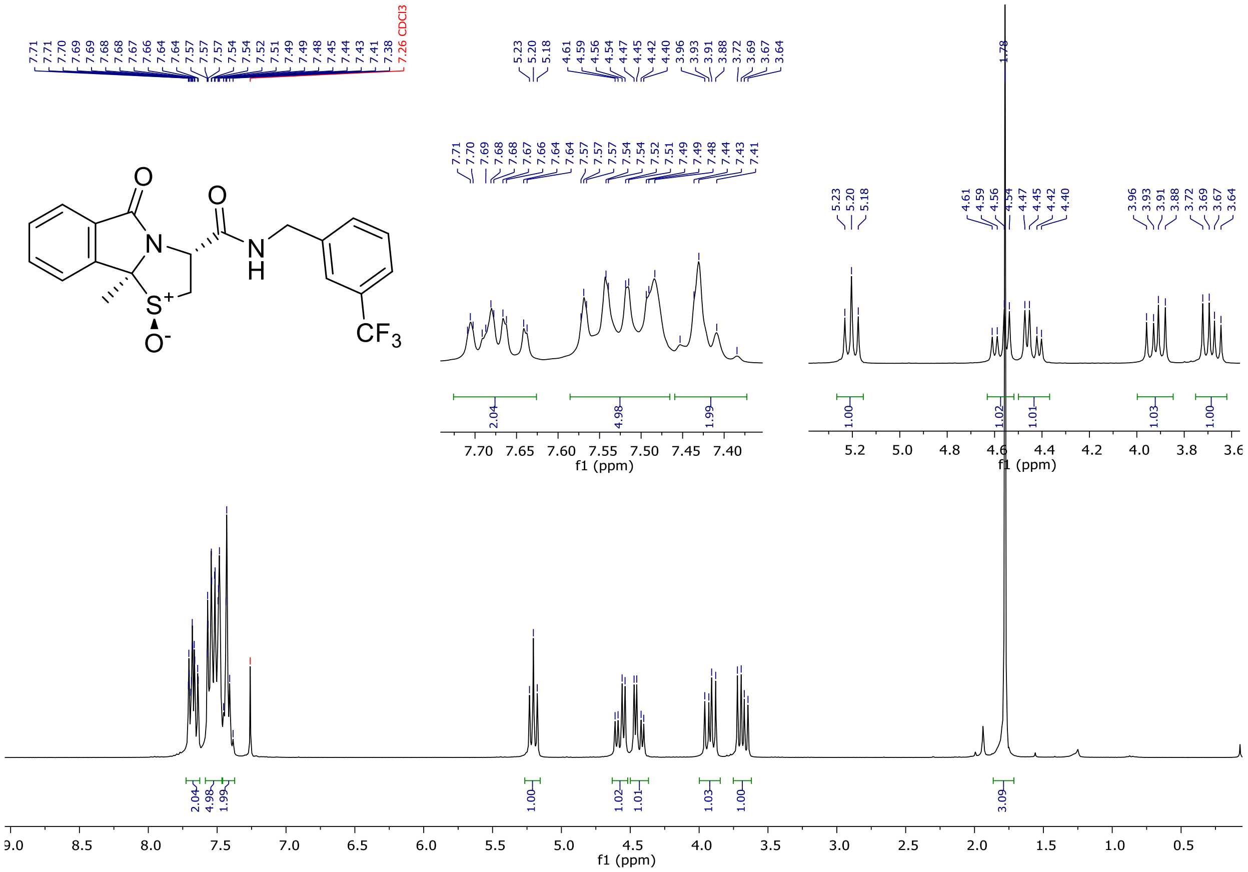

^{1}H NMR spectrum of compound **5Bd**

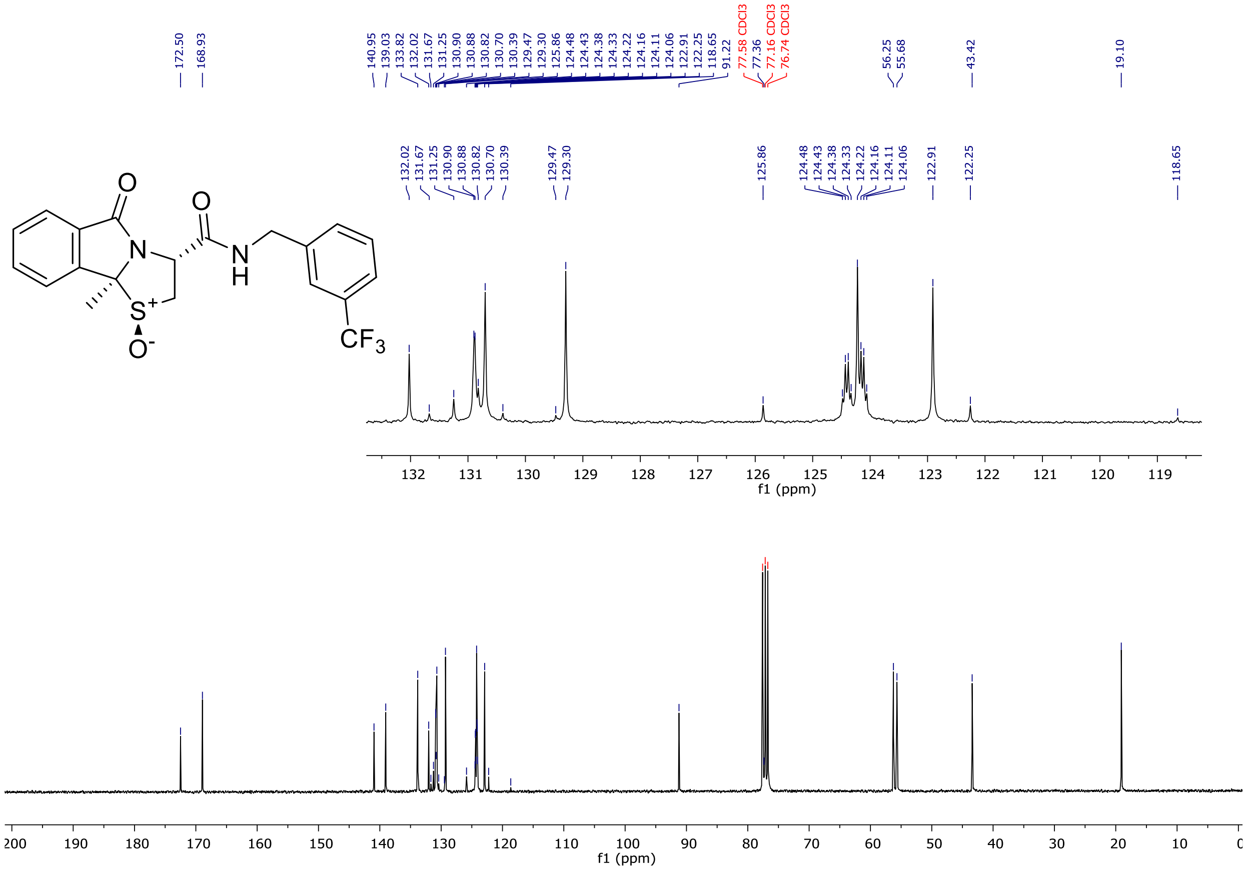

^{13}C NMR spectrum of compound **5bD**

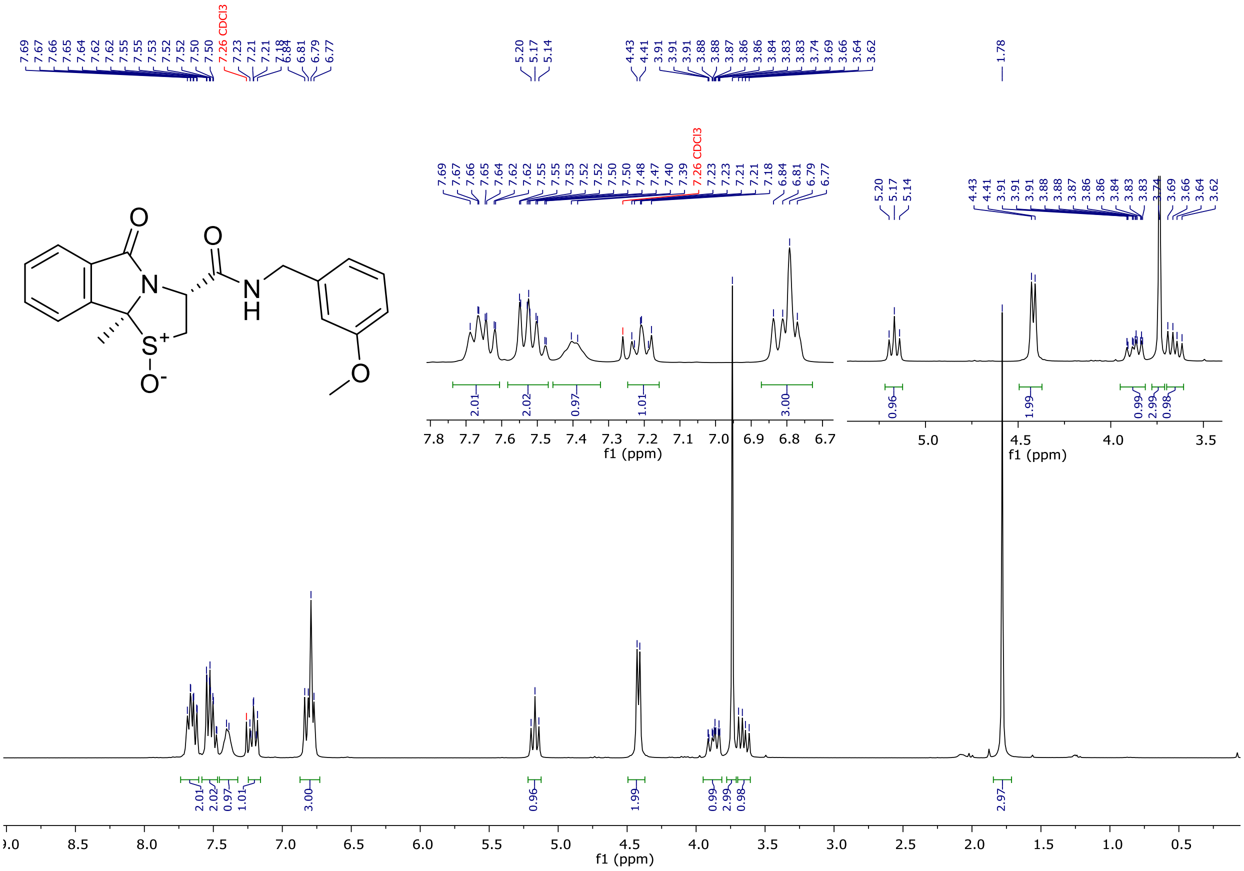

^{1}H NMR spectrum of compound **5bE**

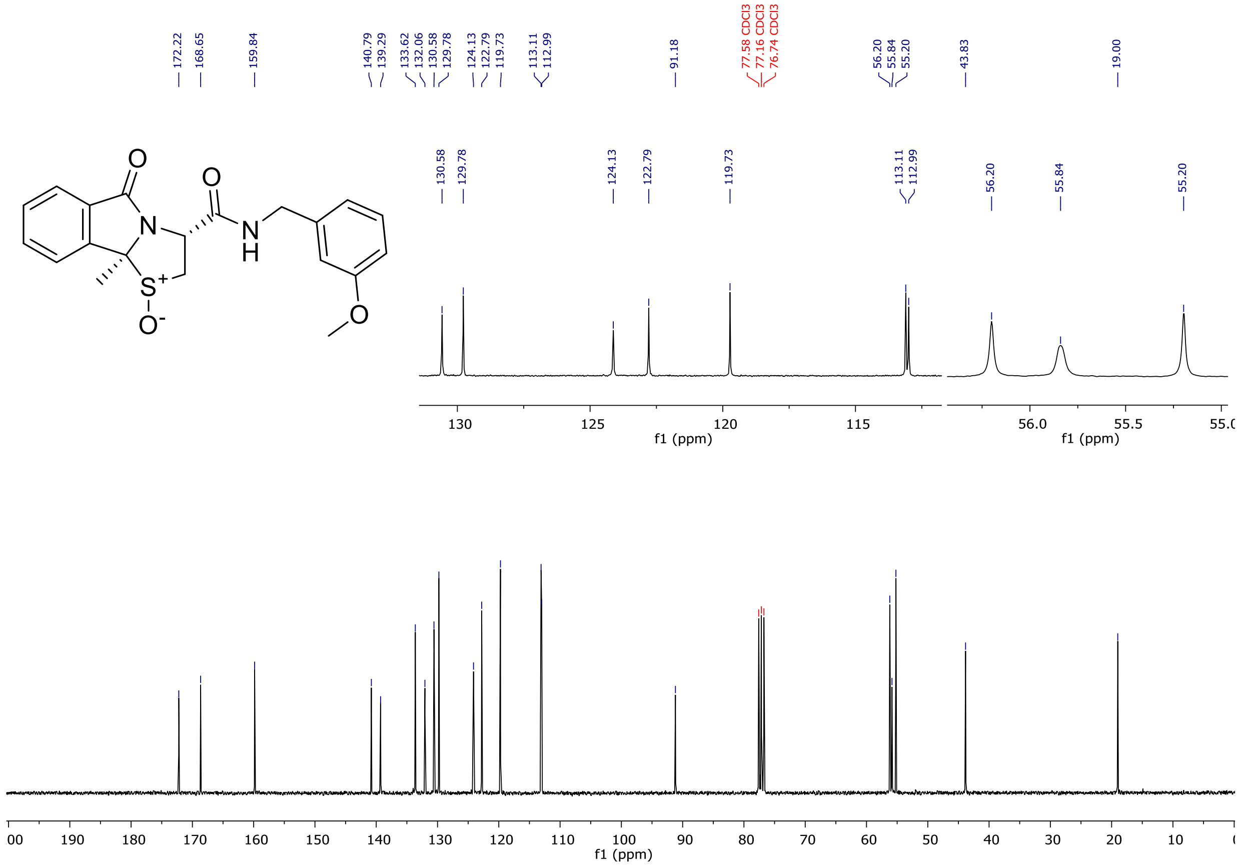

^{13}C NMR spectrum of compound **5bE**

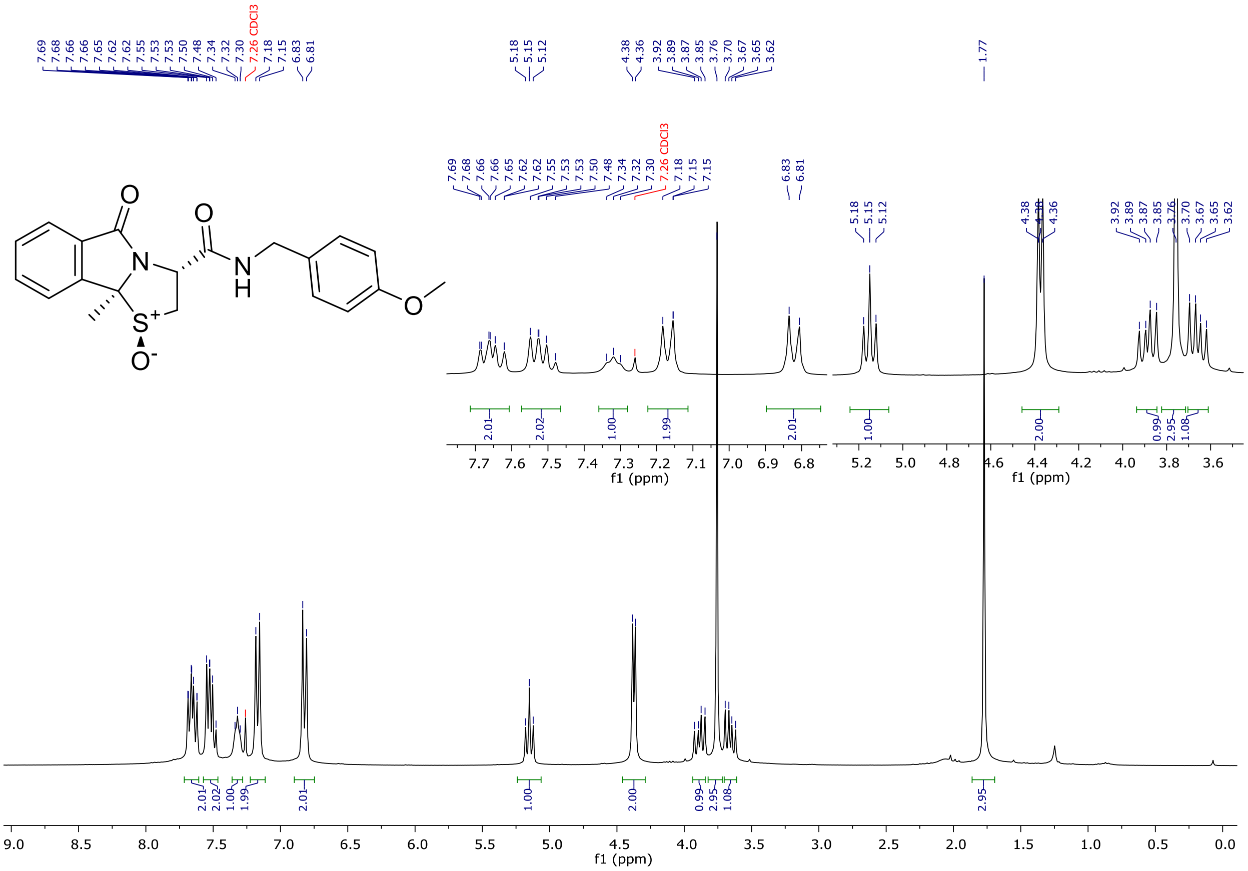

^{1}H NMR spectrum of compound **5bF**

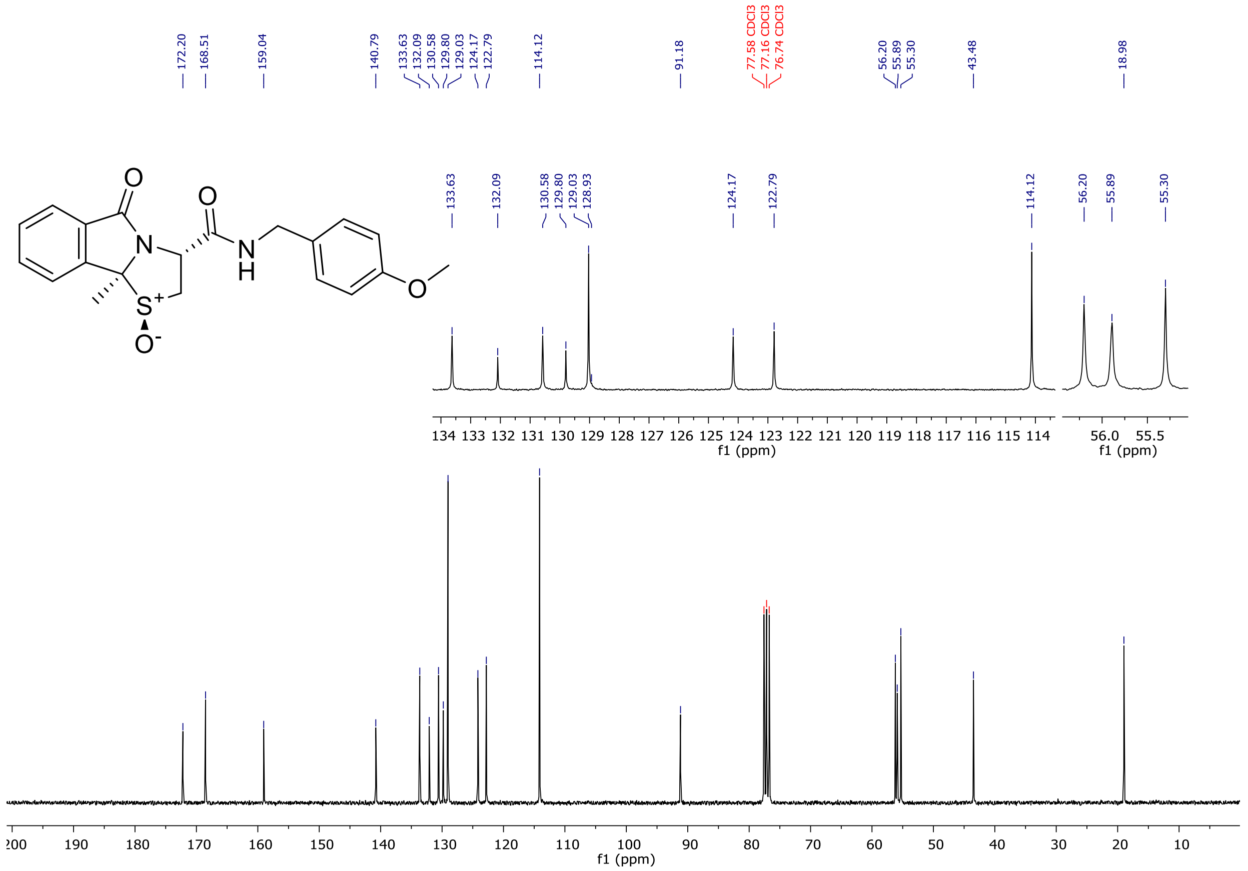

^{13}C NMR spectrum of compound **5bF**

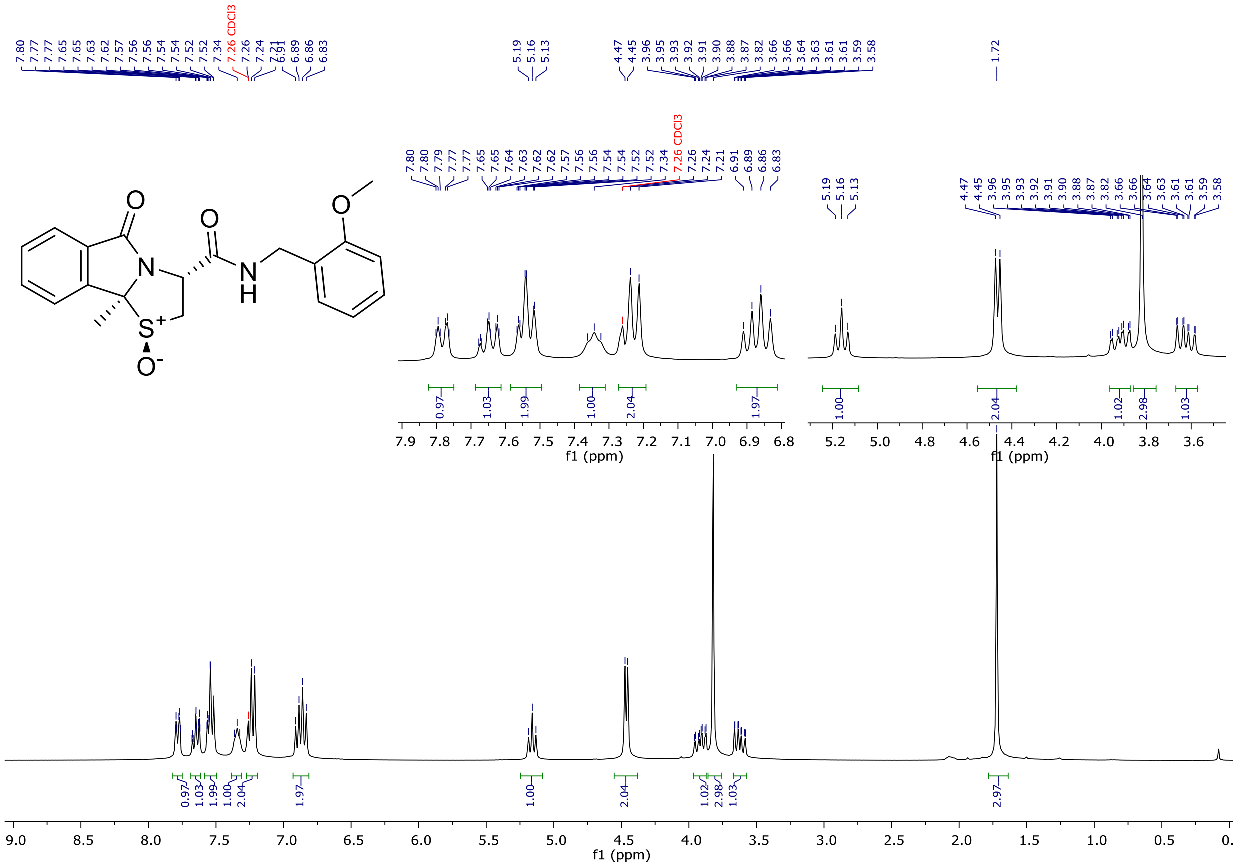

^{1}H NMR spectrum of compound **5bG**

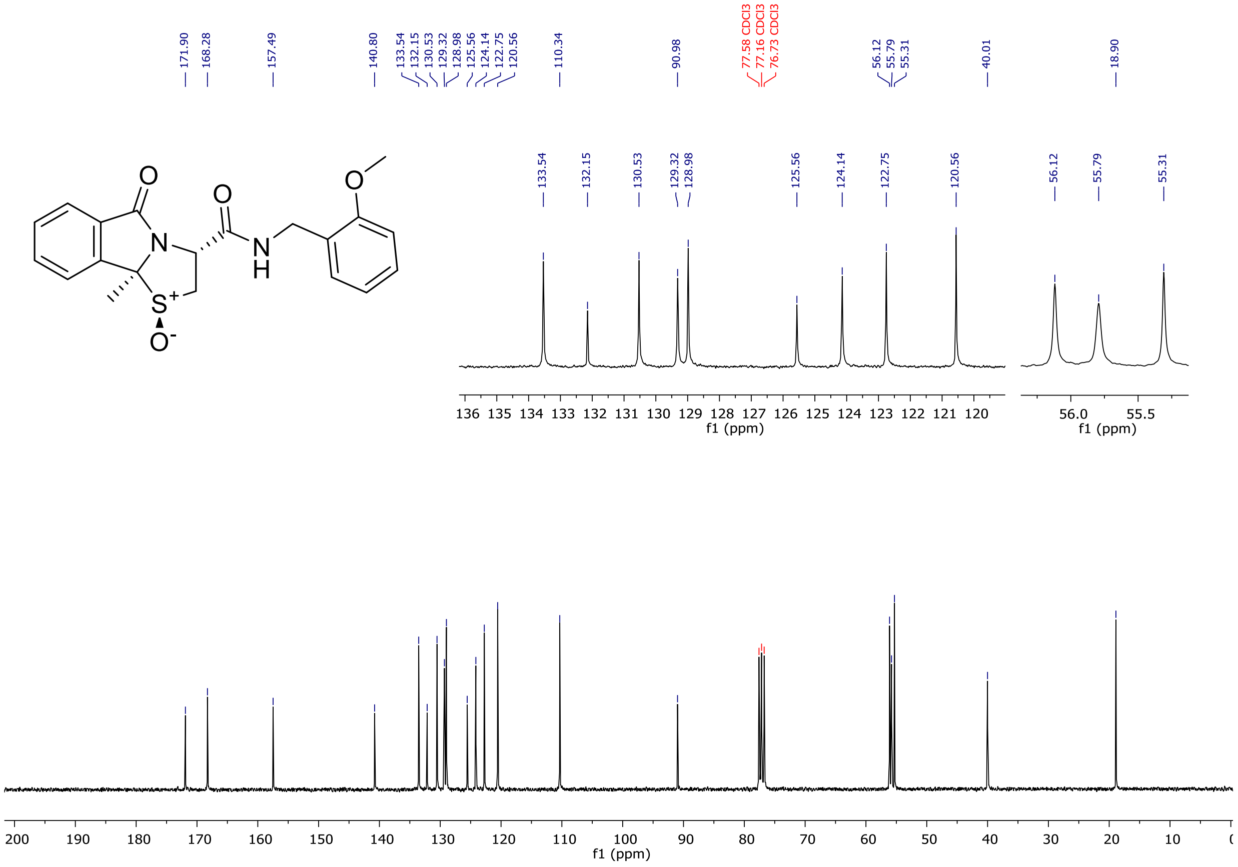

^{13}C NMR spectrum of compound **5bG**

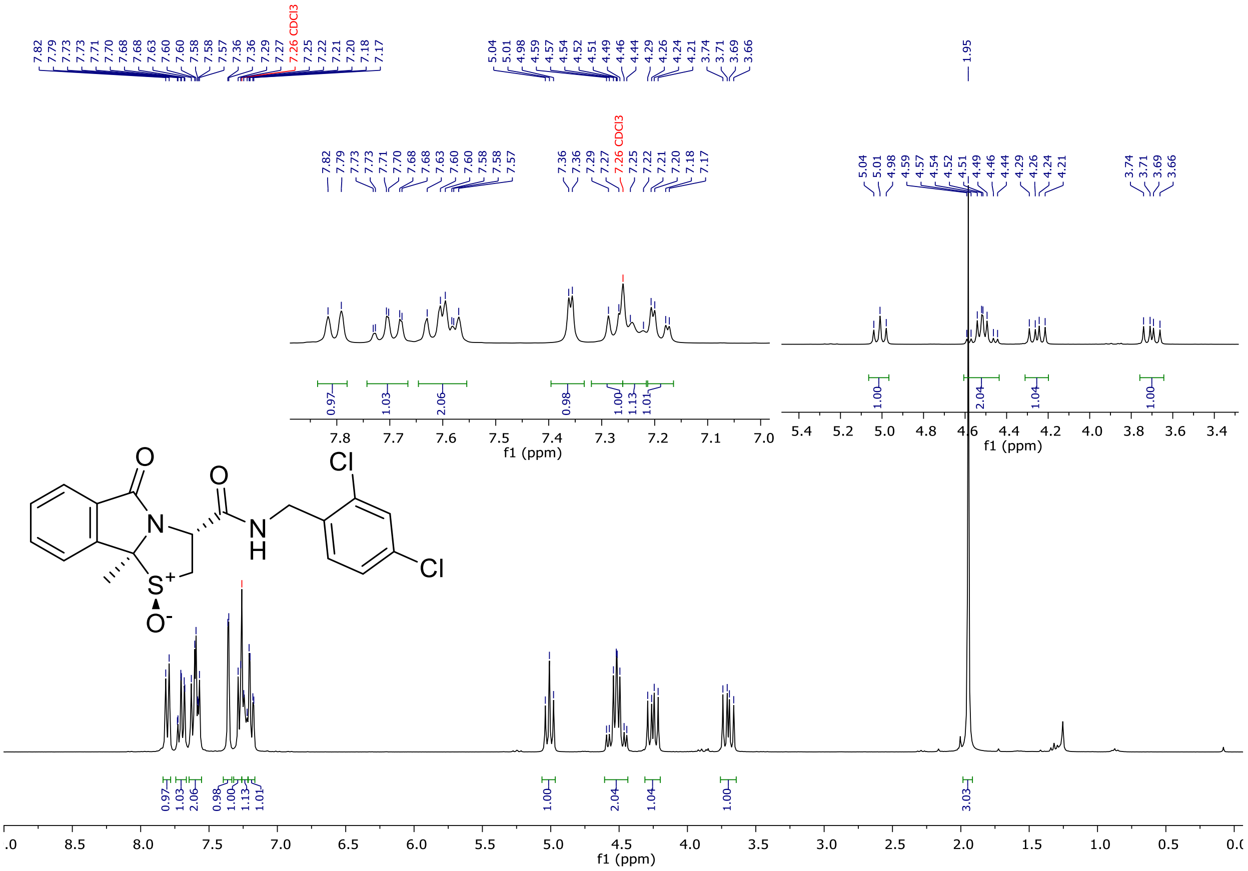

^{1}H NMR spectrum of compound **5bH**

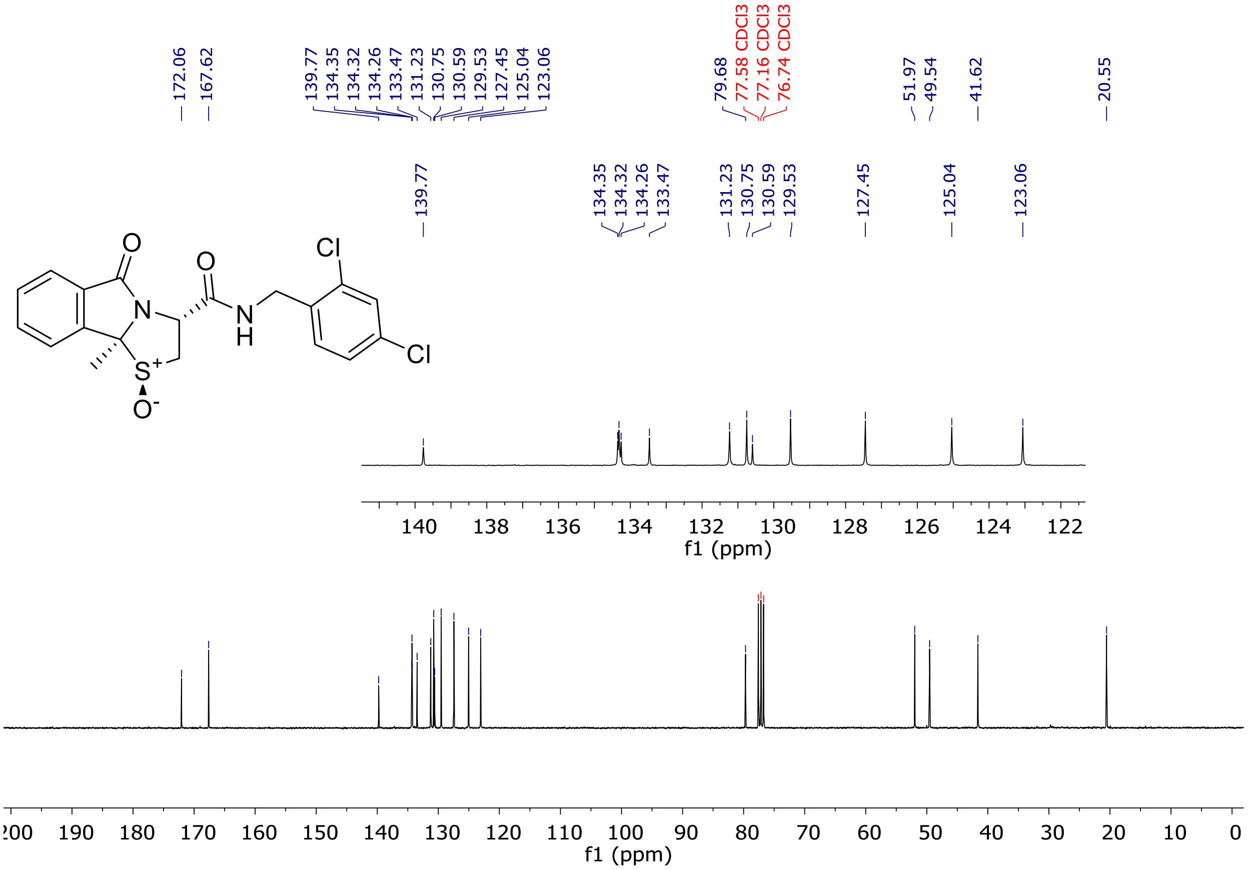

^{13}C NMR spectrum of compound **5bH**

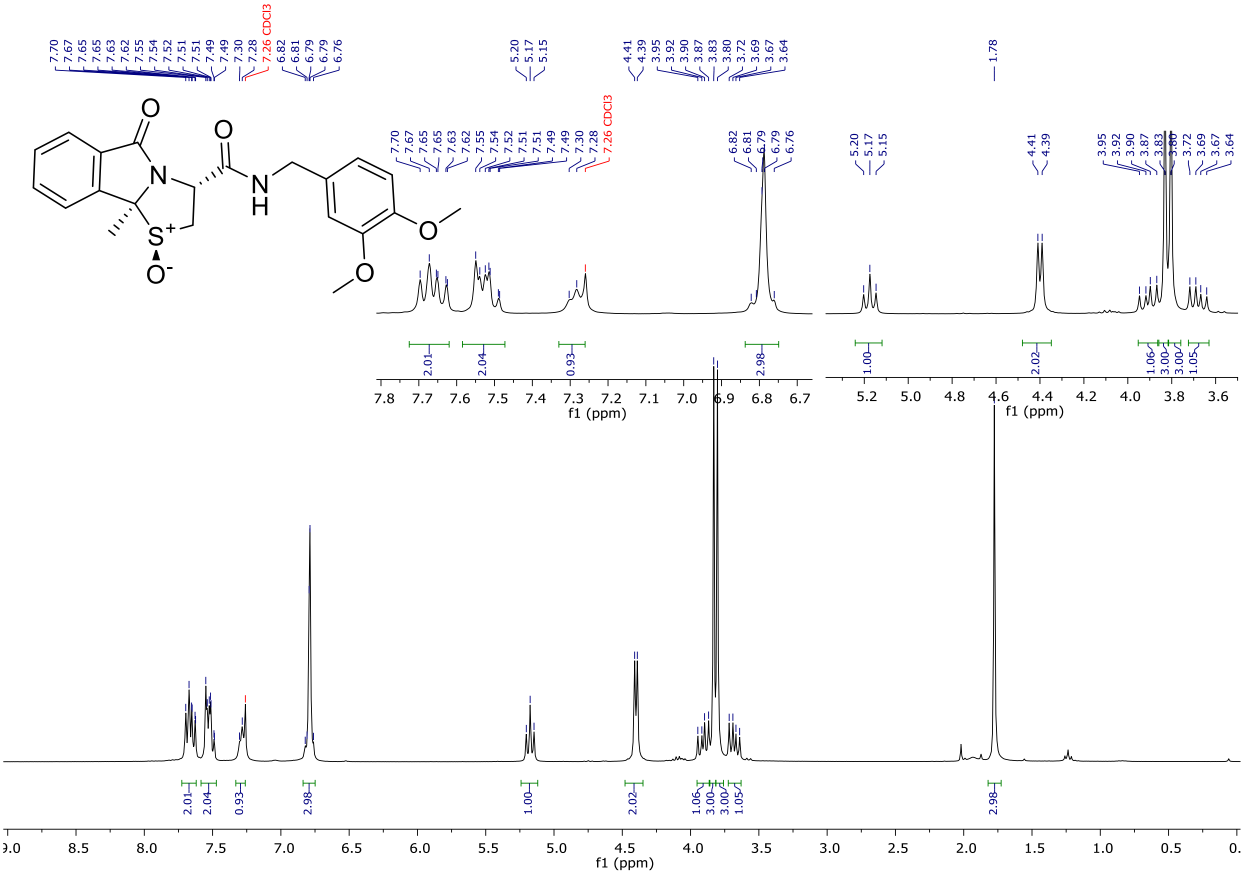

^{1}H NMR spectrum of compound **5bI**

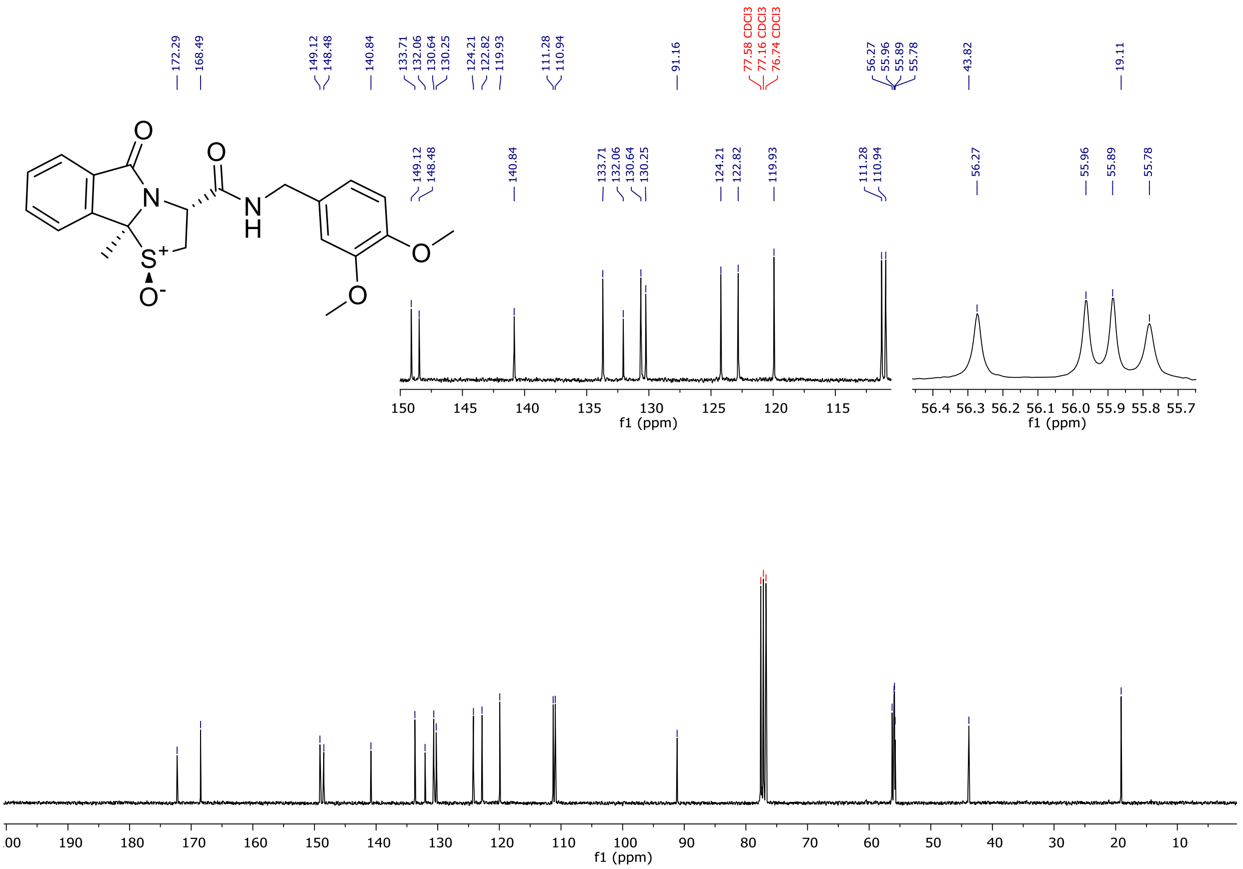

^{13}C NMR spectrum of compound **5bI**

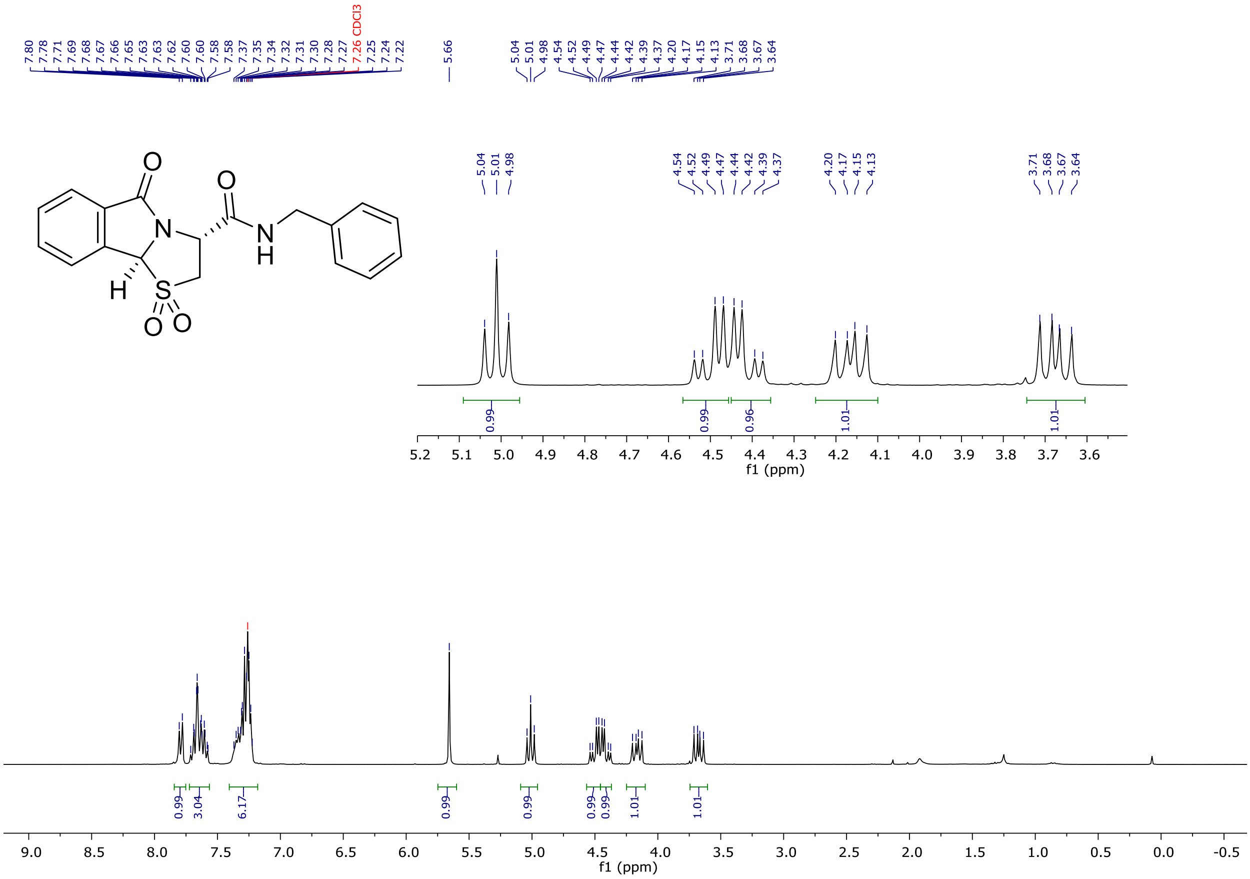

^{1}H NMR spectrum of compound **7aA**

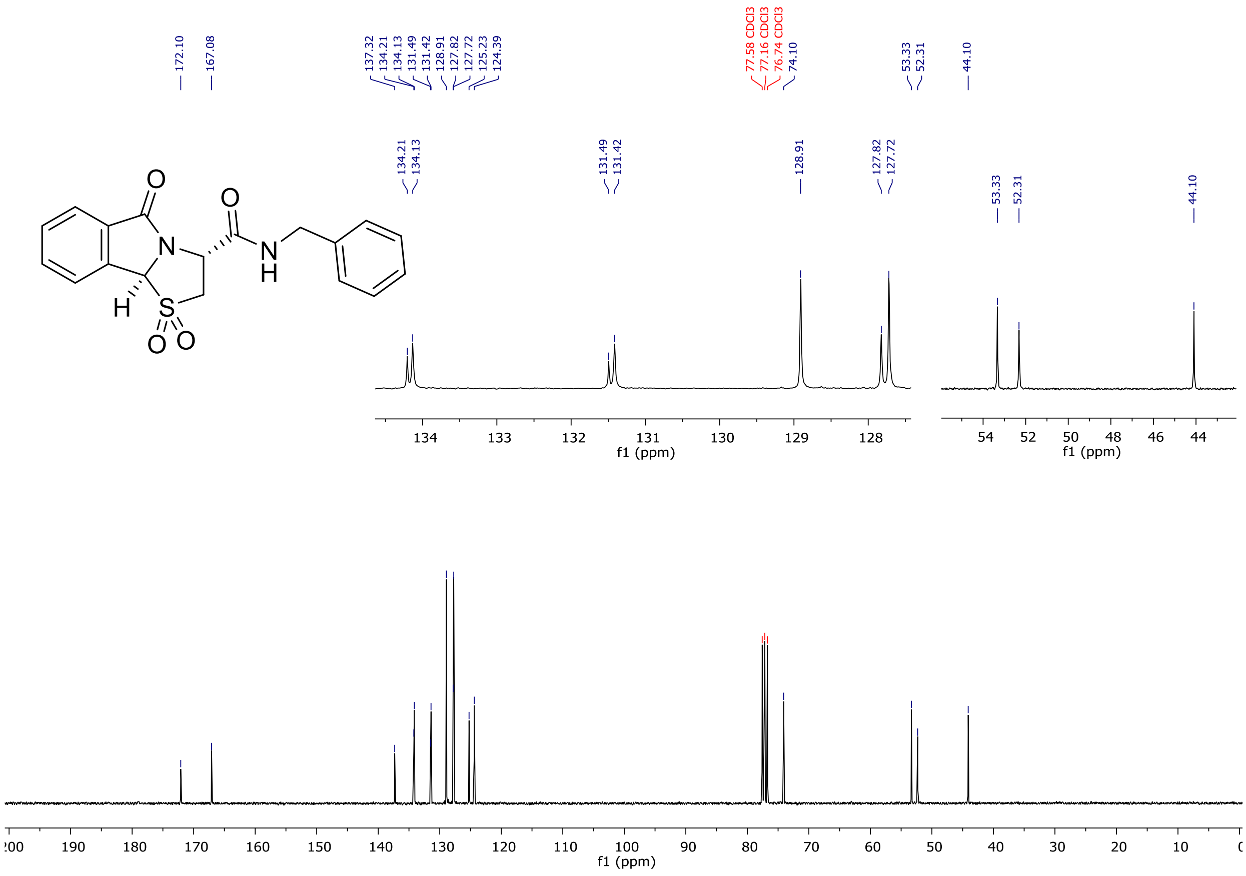

^{13}C NMR spectrum of compound **7aA**

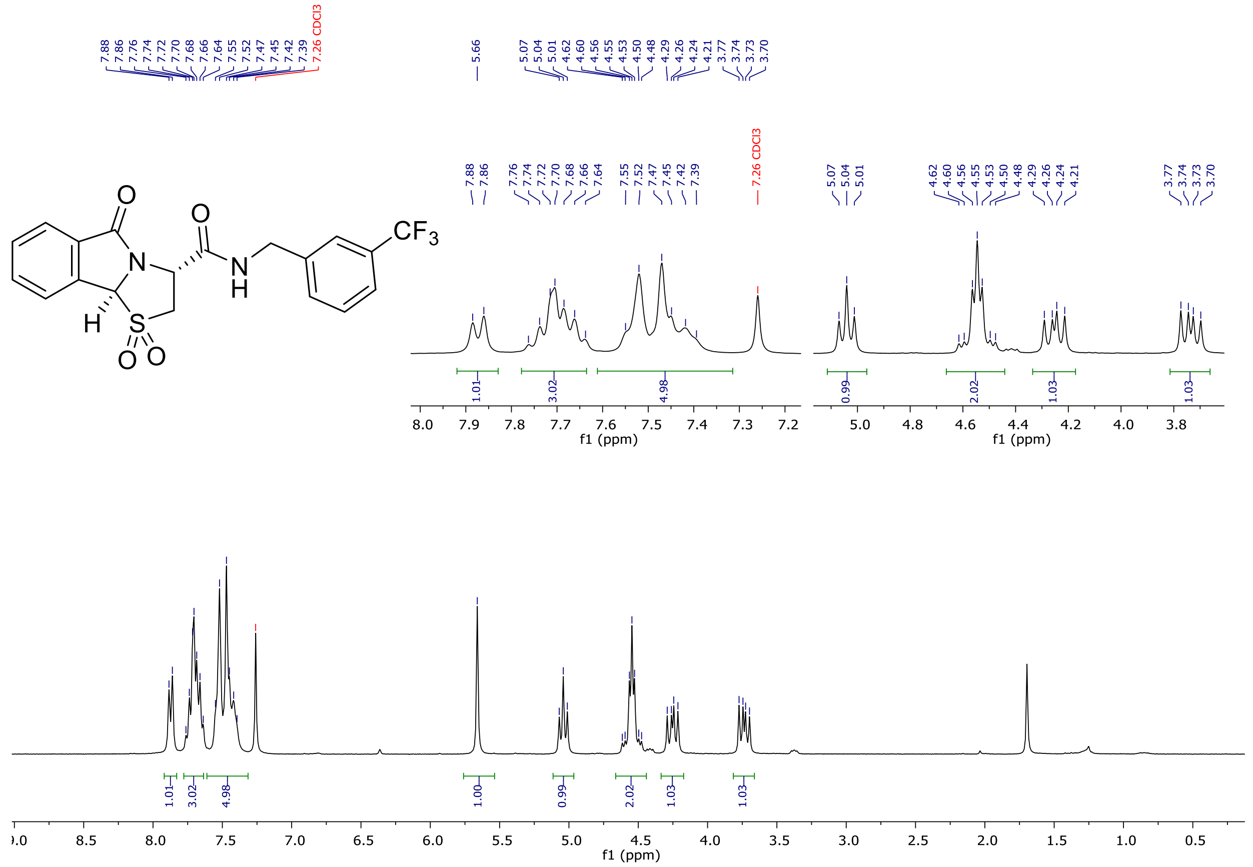

^{1}H NMR spectrum of compound **7aD**

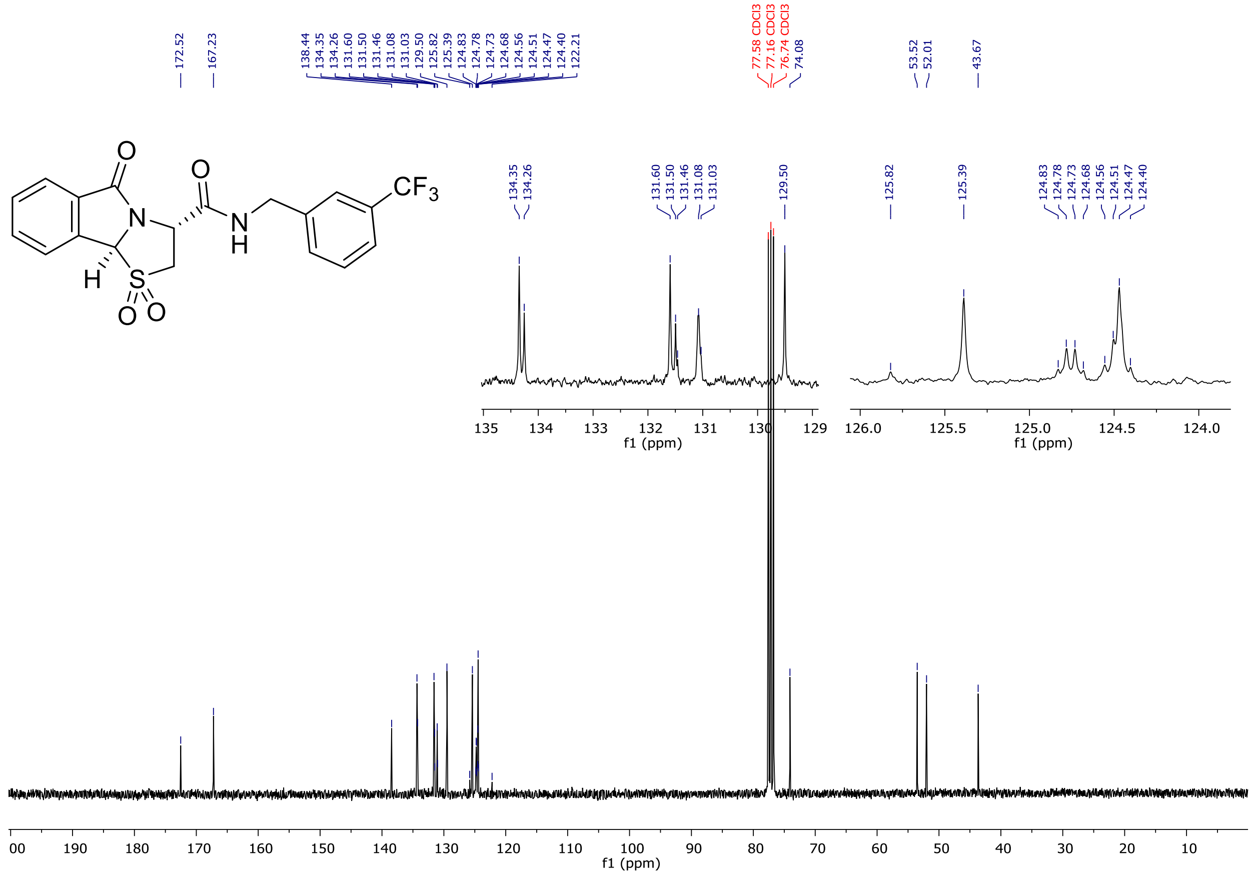

^{13}C NMR spectrum of compound **7aD**

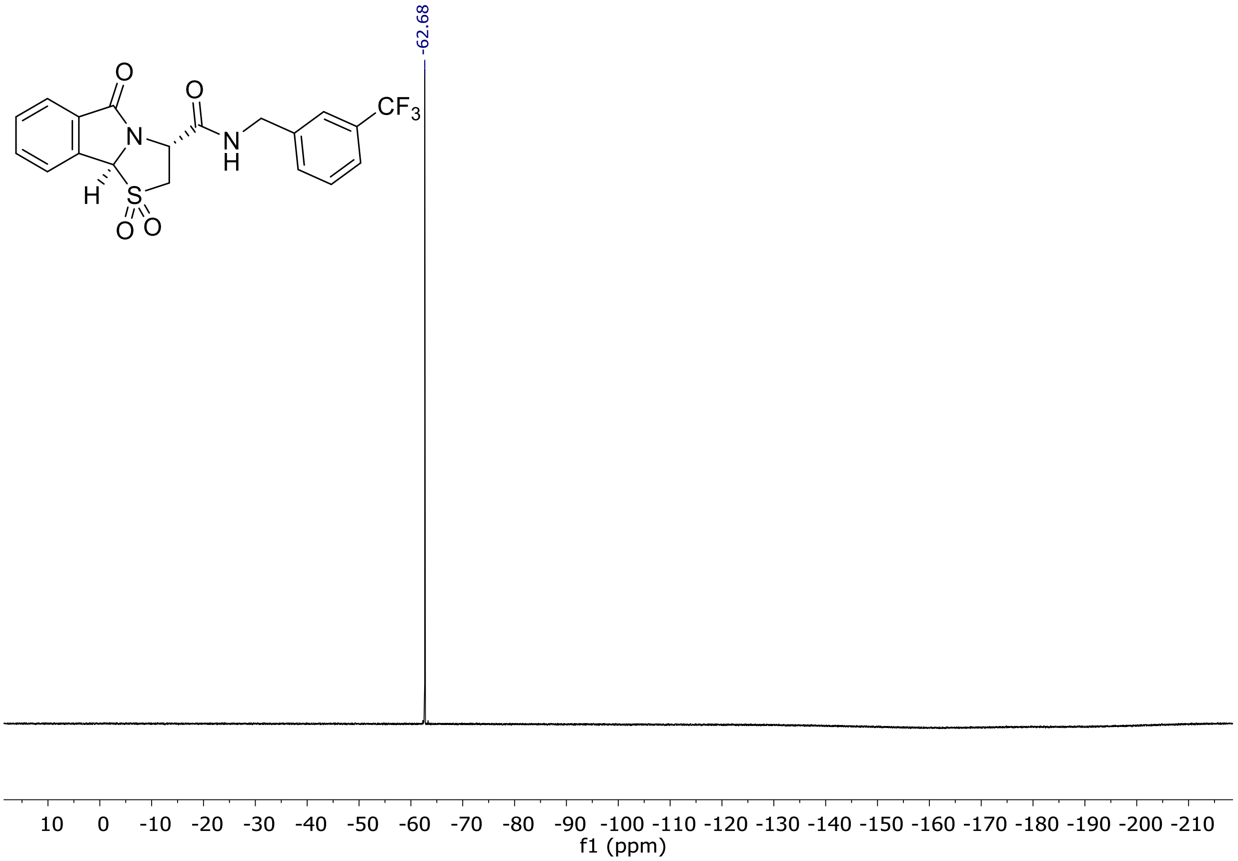

^{19}F NMR spectrum of compound **7aD**

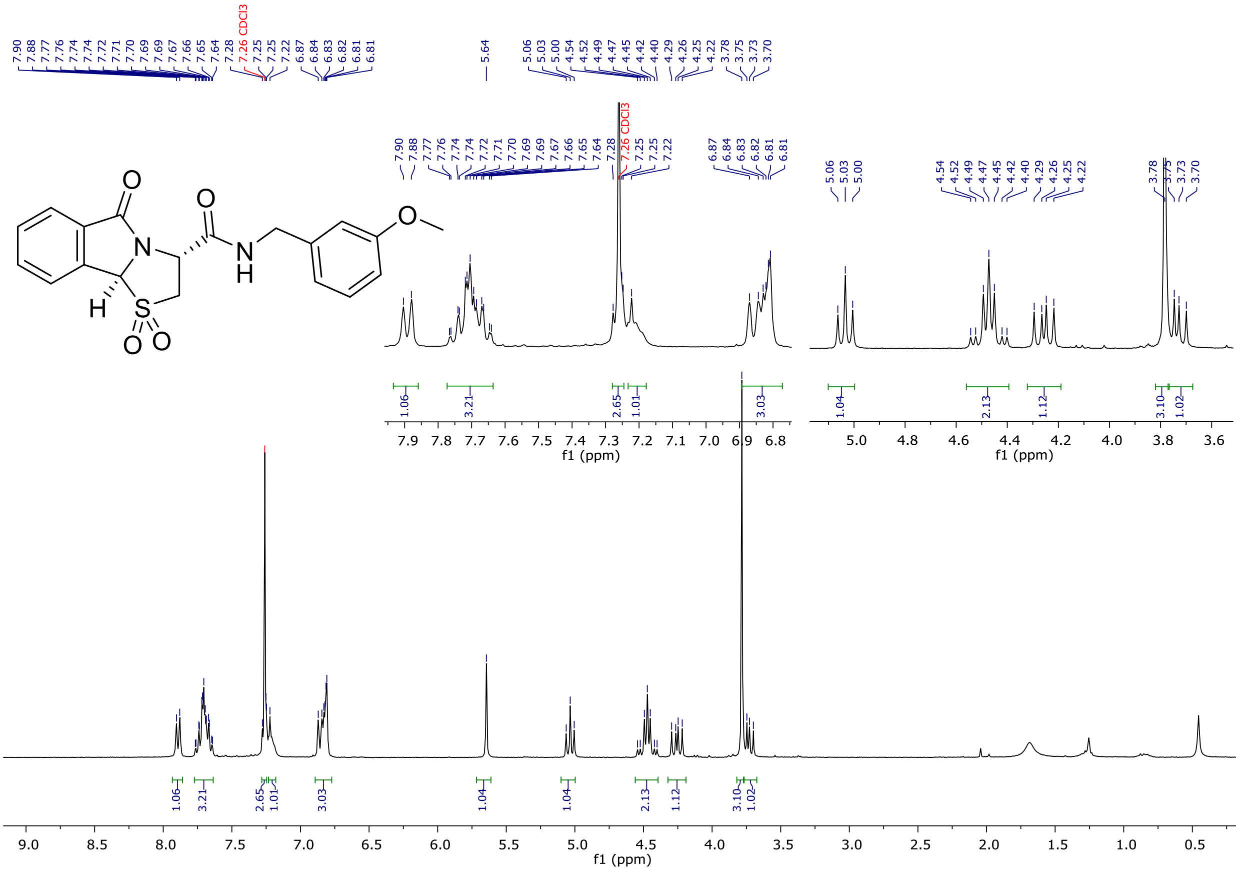

^{1}H NMR spectrum of compound **7aE**

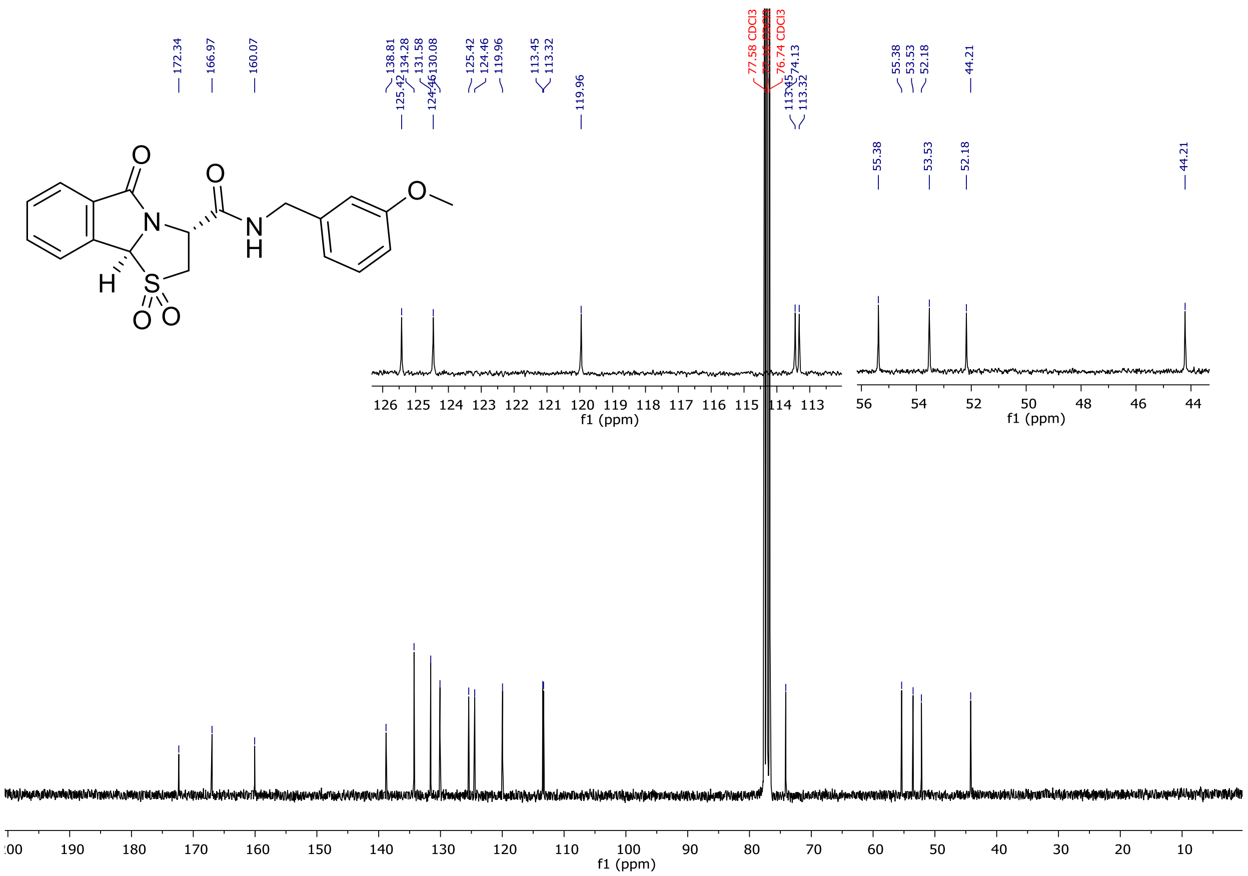

^{13}C NMR spectrum of compound **7aE**

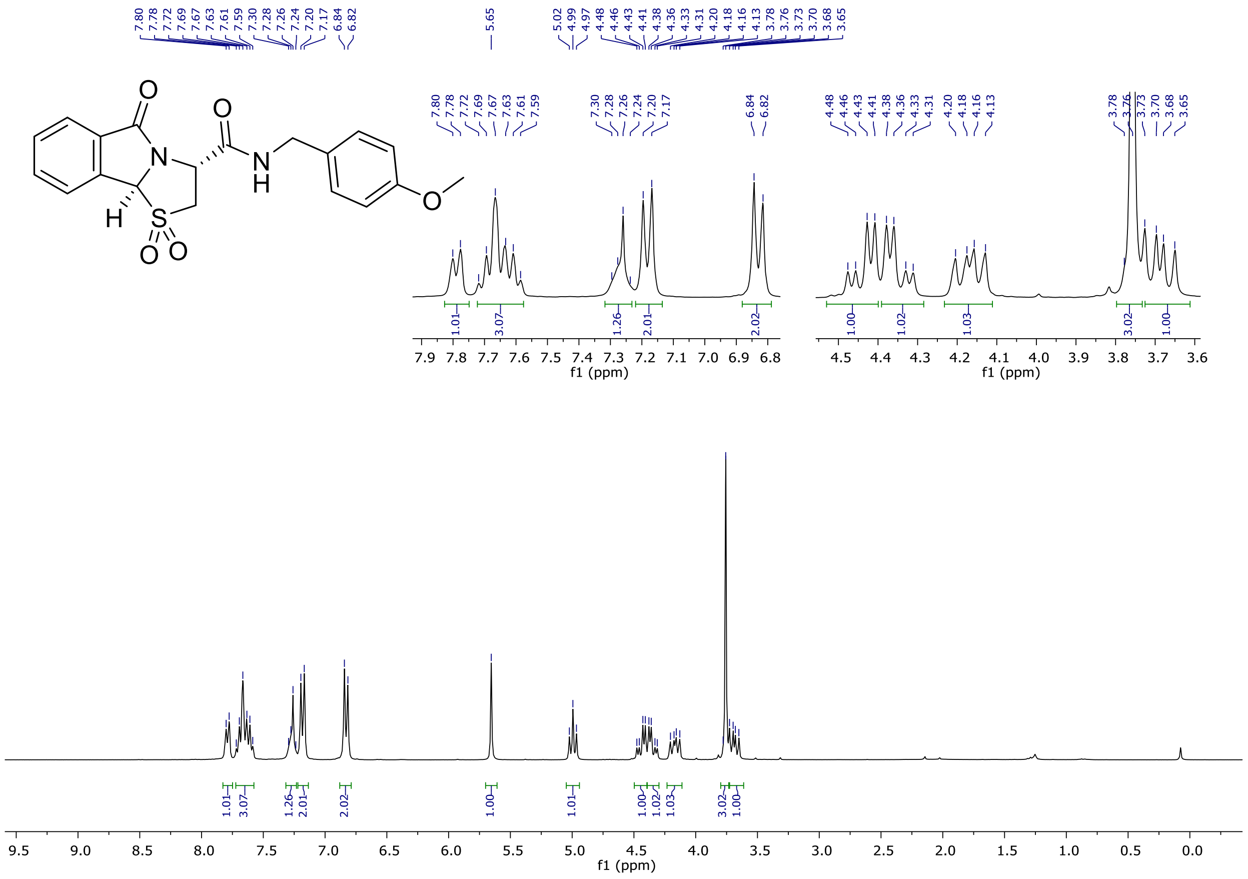

^{13}C NMR spectrum of compound **7aF**

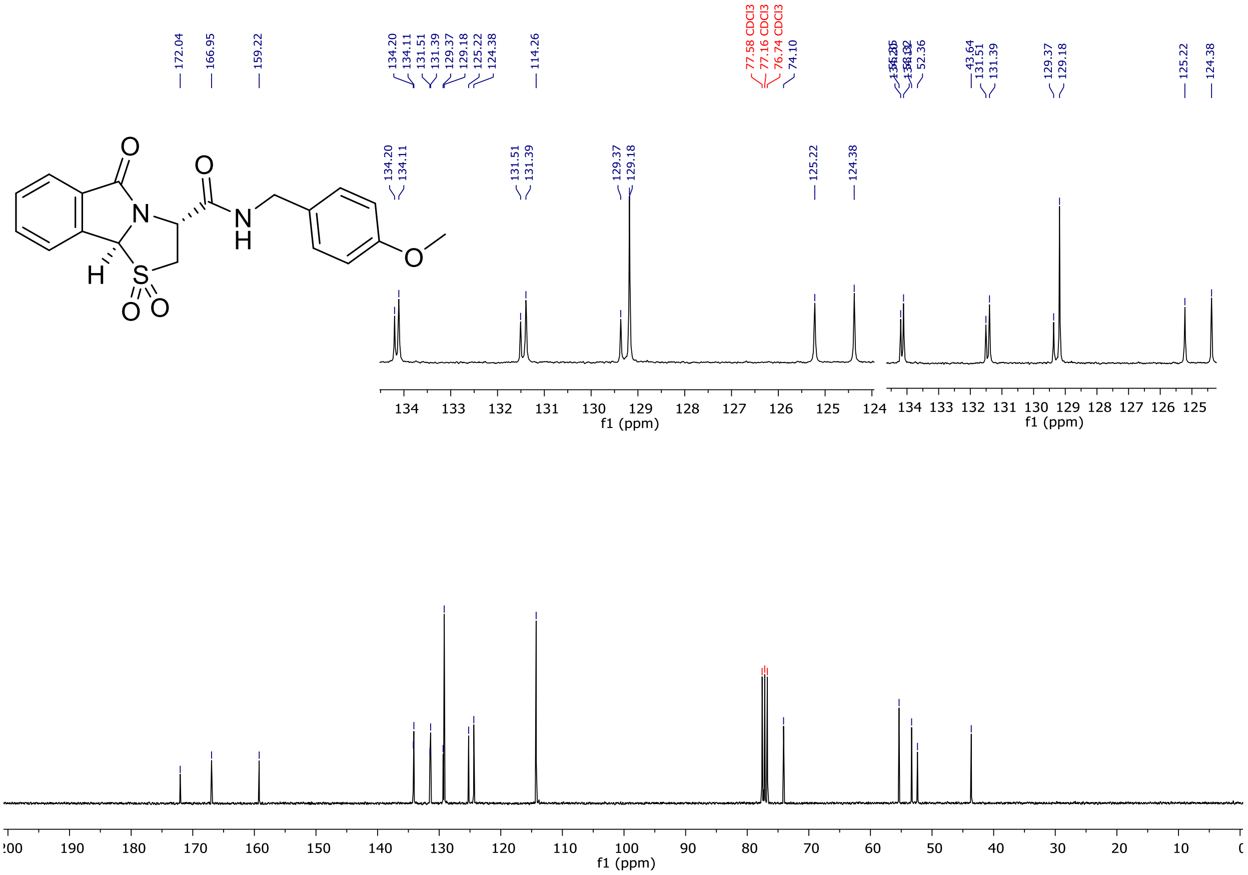

^{13}C NMR spectrum of compound **7aF**

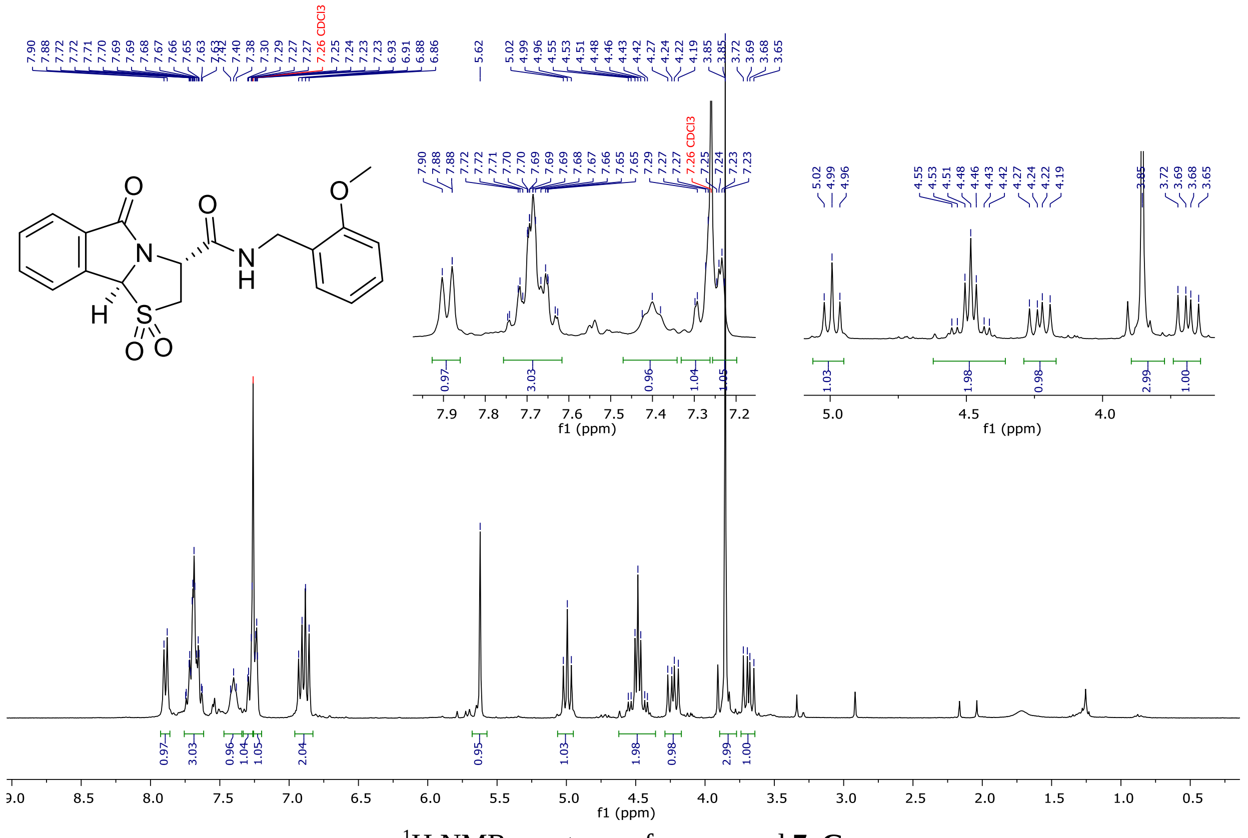

^{1}H NMR spectrum of compound **7aG**

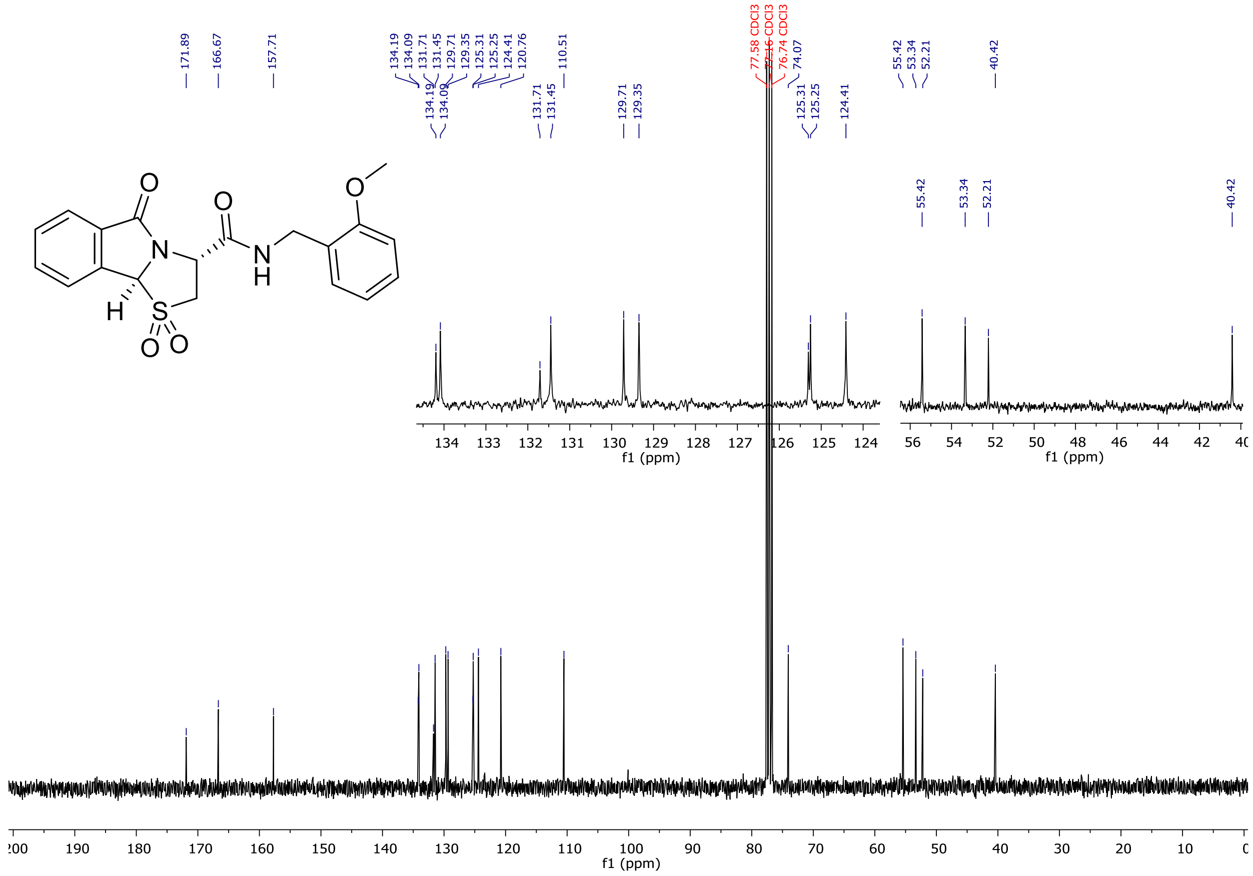

^{13}C NMR spectrum of compound **7aG**

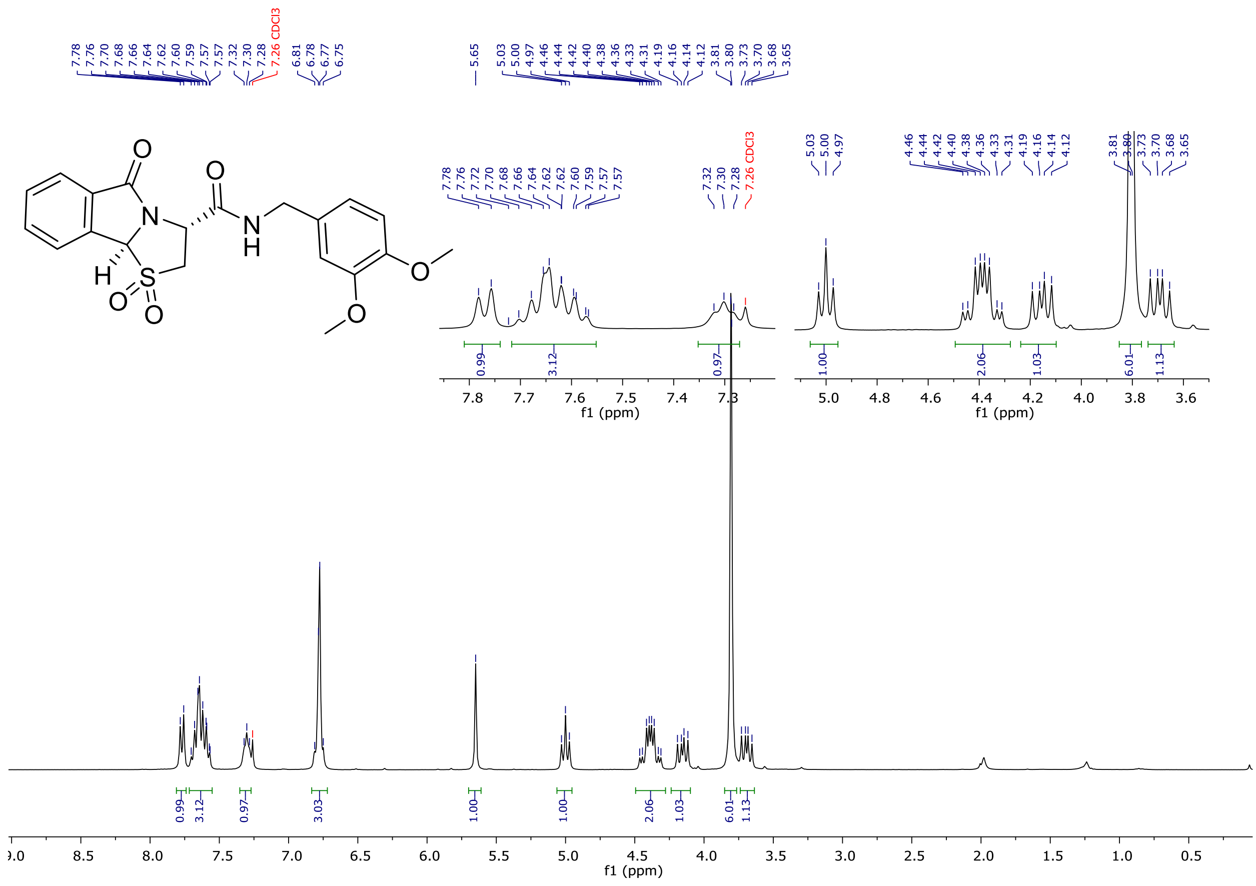

^{1}H NMR spectrum of compound **7aI**

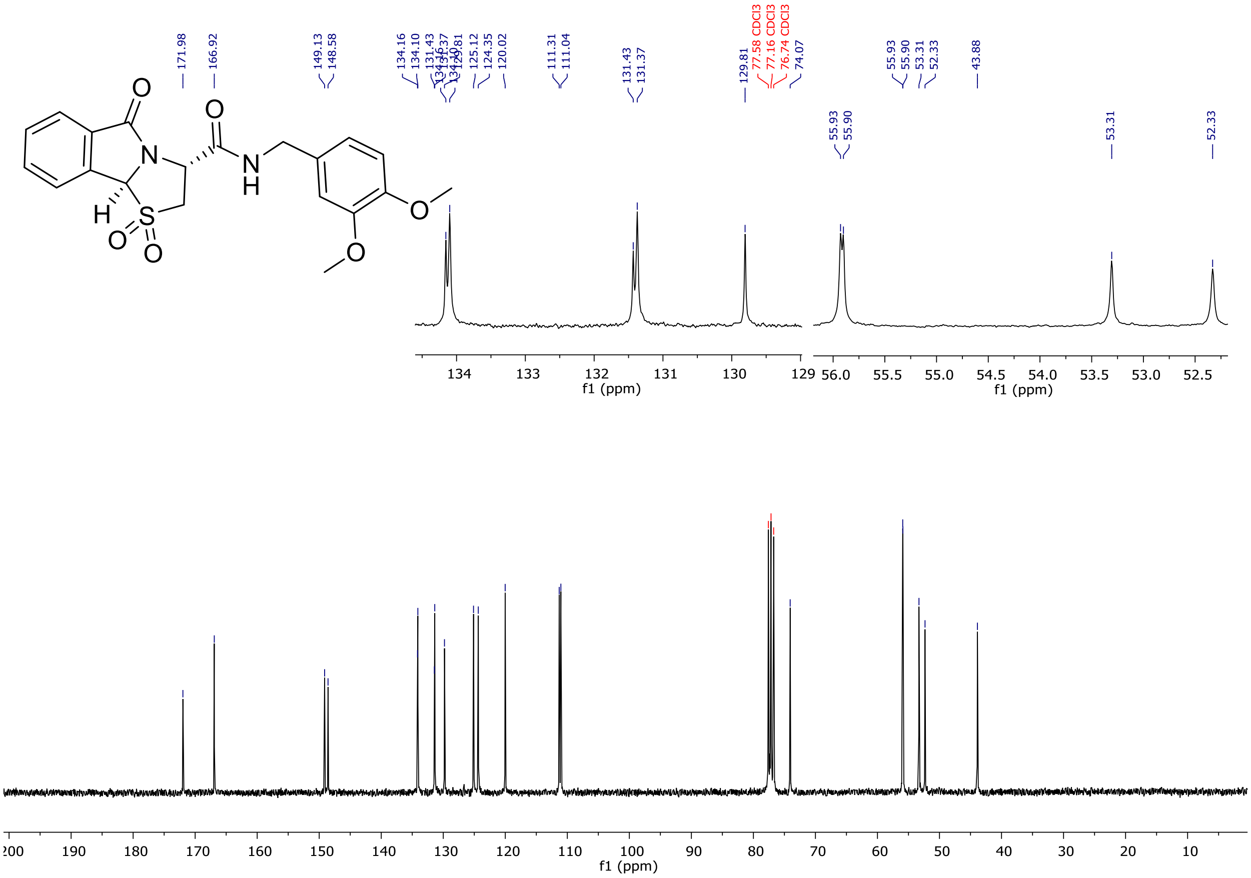

^{13}C NMR spectrum of compound **7aI**

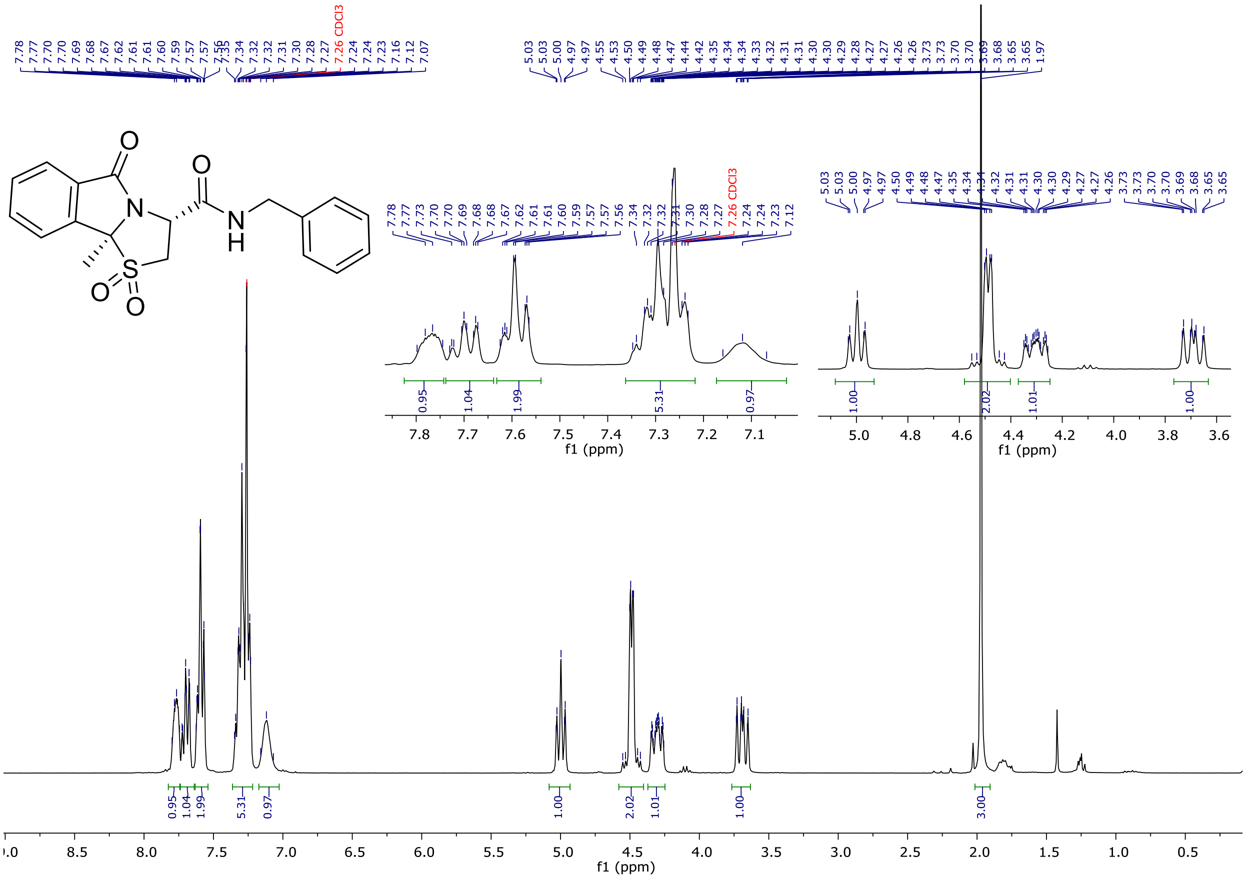

^{1}H NMR spectrum of compound **7bA**

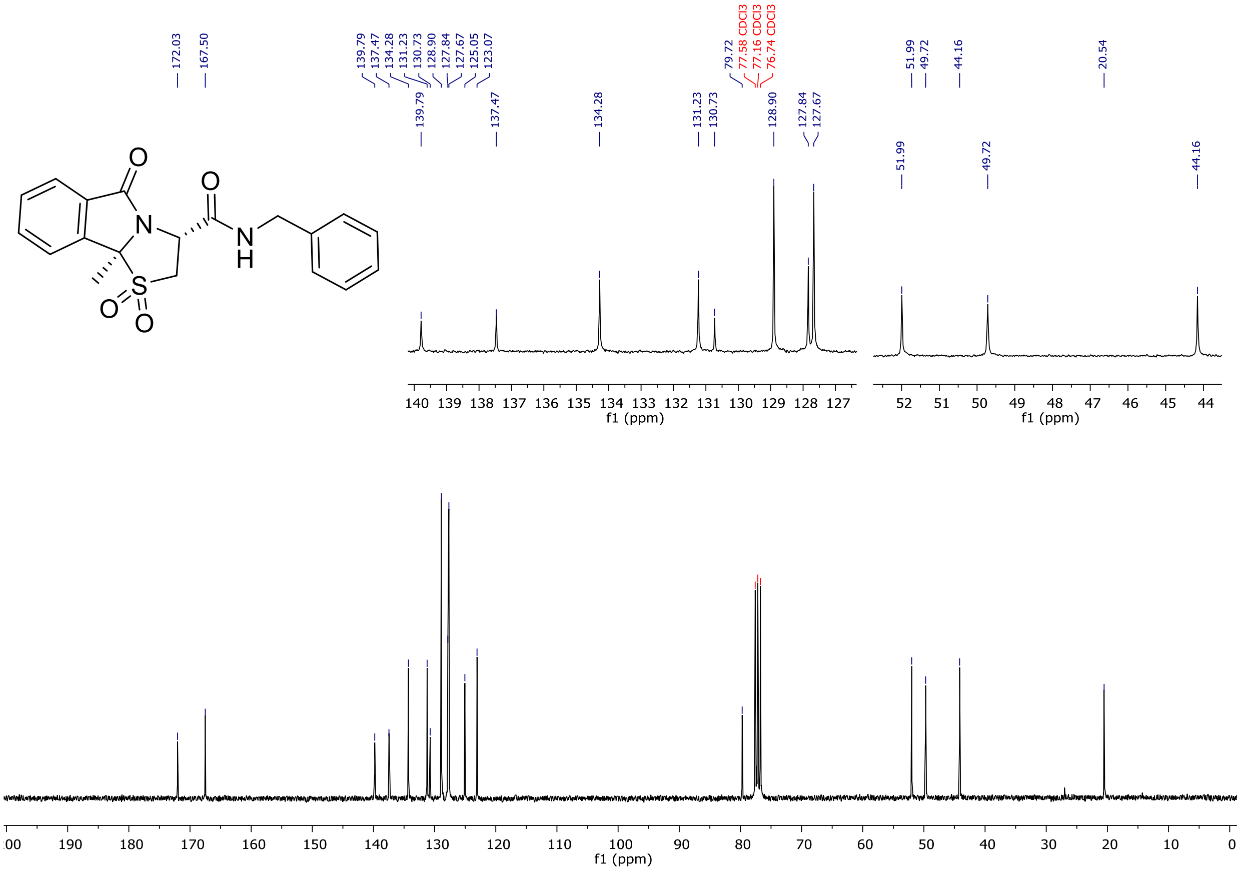

^{13}C NMR spectrum of compound **7bA**

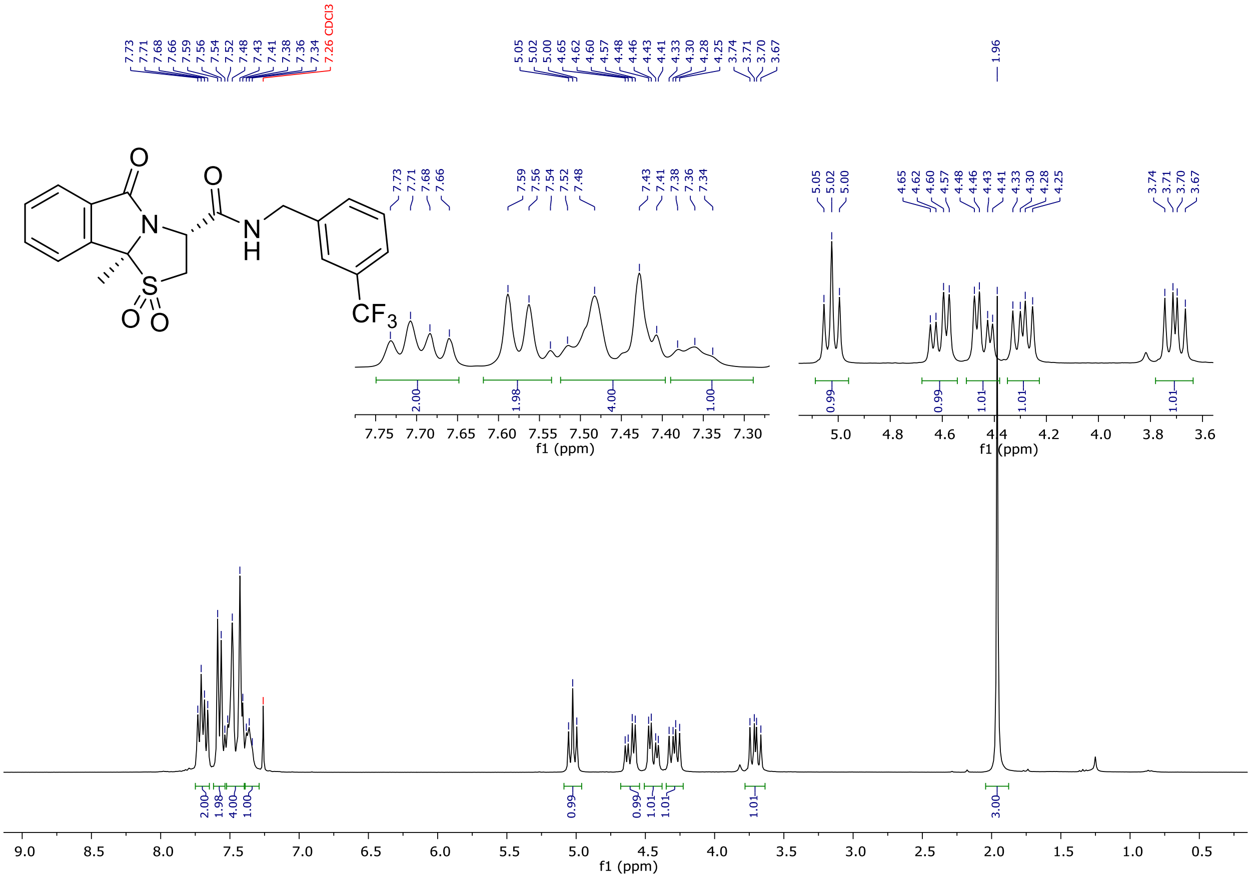

^{1}H NMR spectrum of compound **7bD**

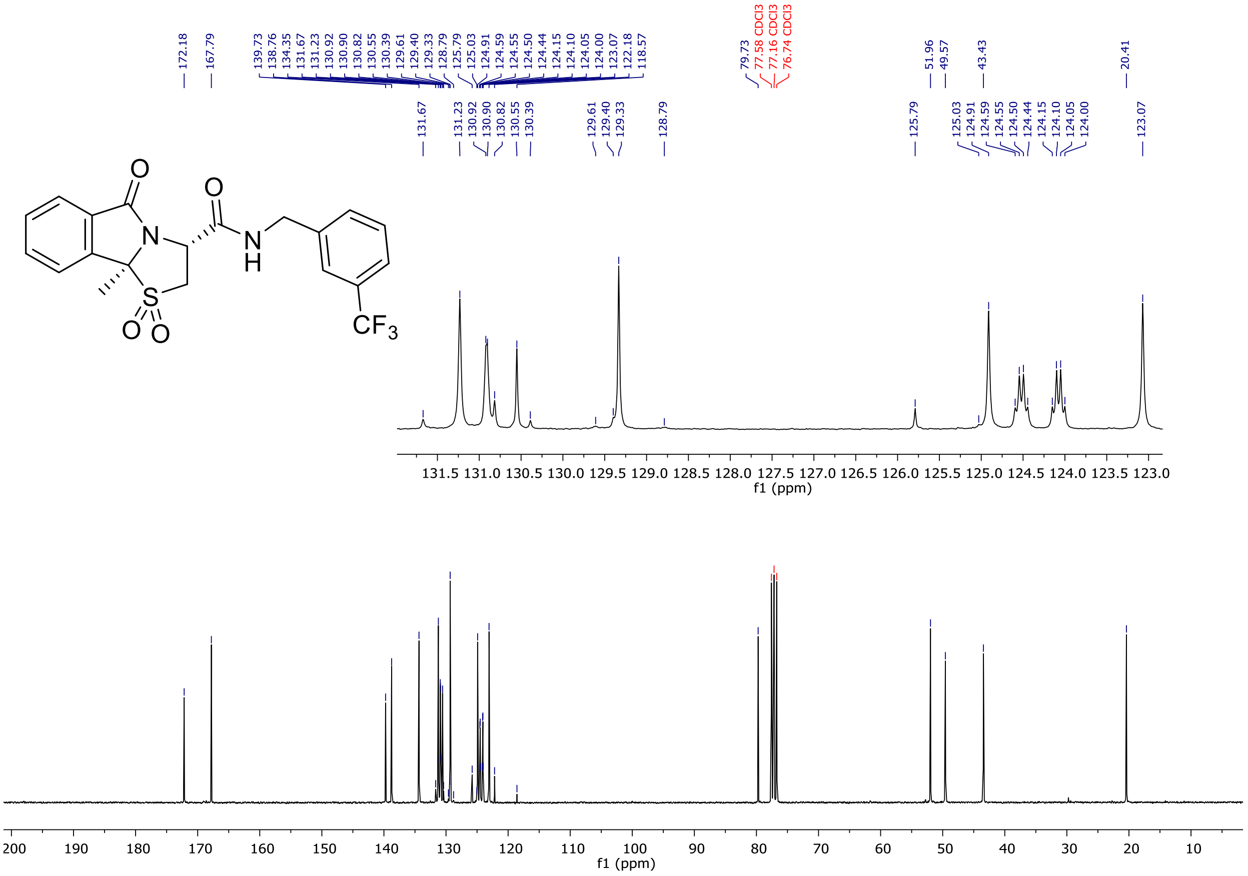

^{13}C NMR spectrum of compound **7bD**

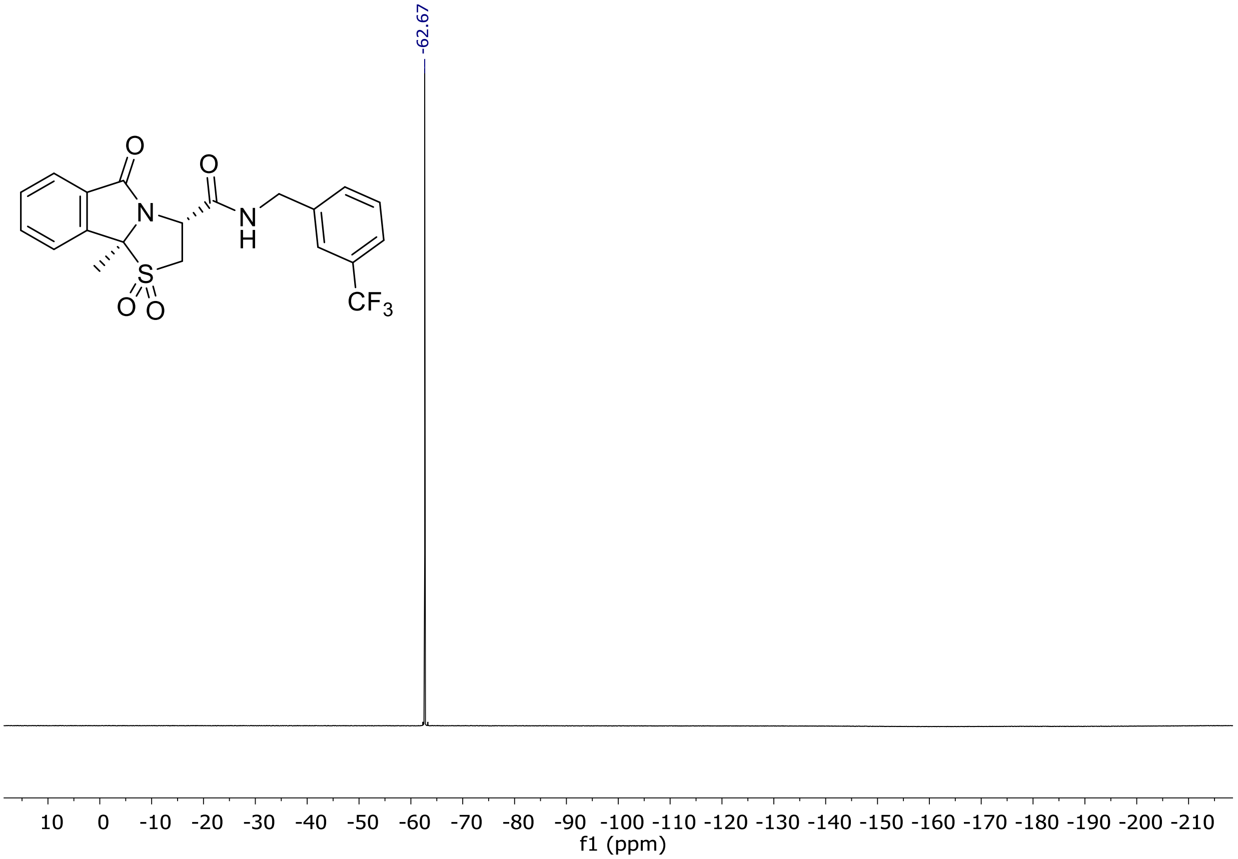

^{19}F NMR spectrum of compound **7bD**

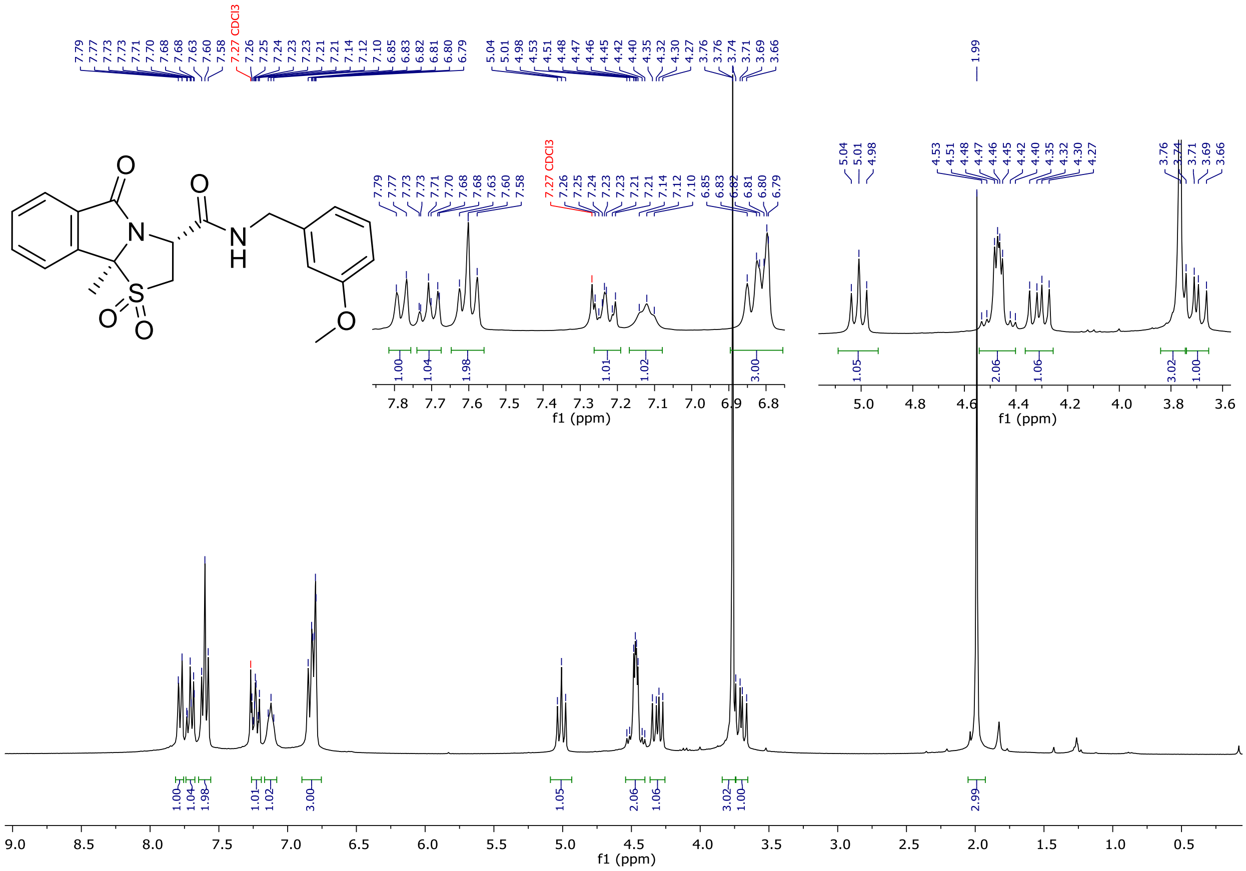

^{1}H NMR spectrum of compound **7bE**

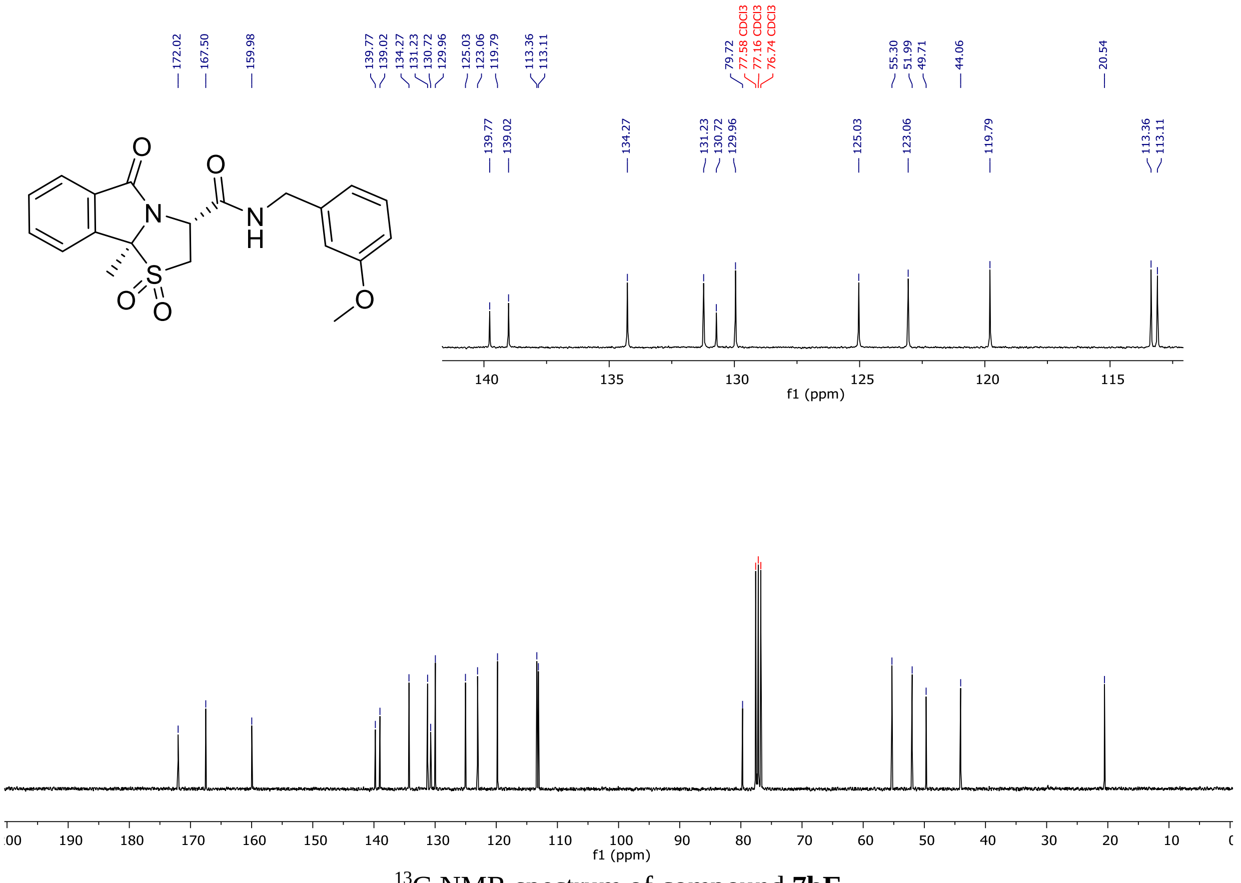

^{13}C NMR spectrum of compound **7bE**

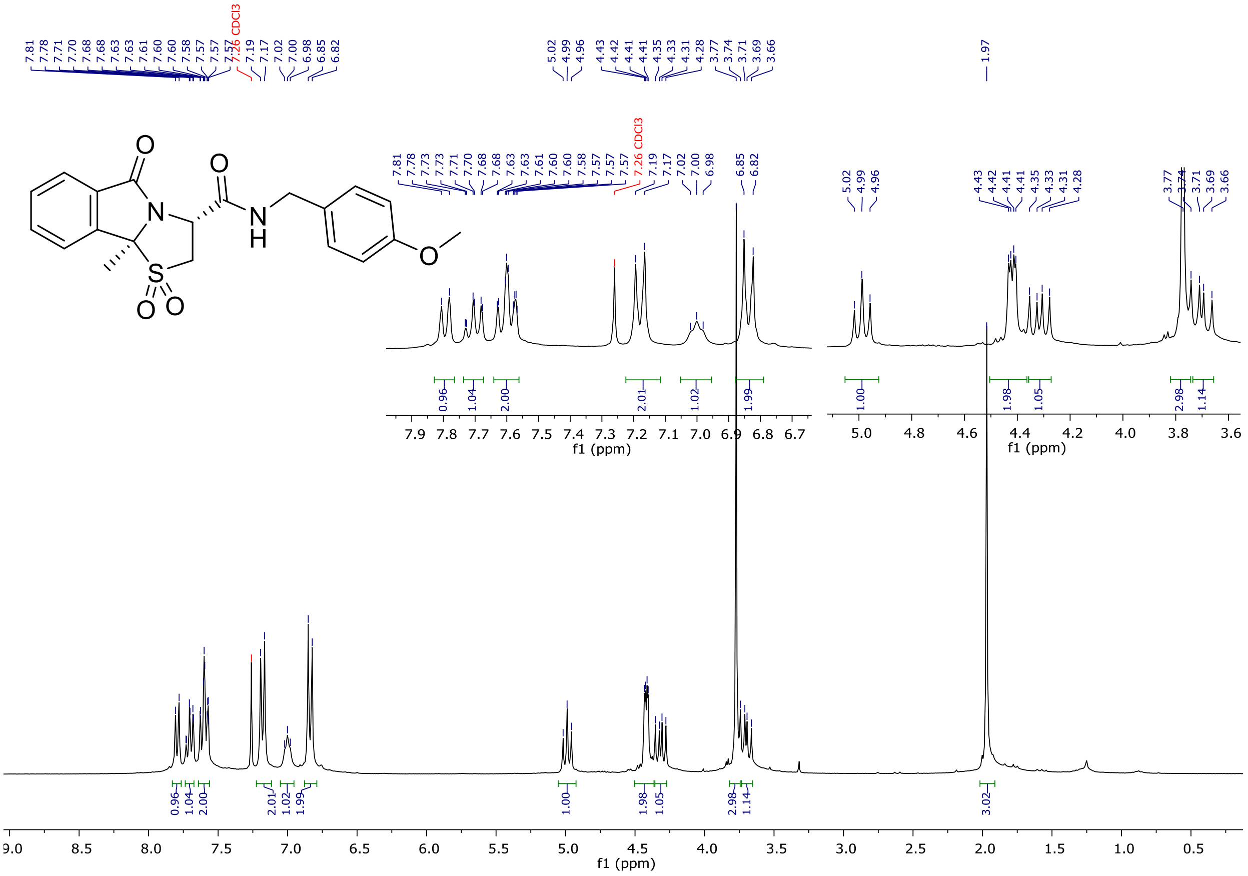

^{1}H NMR spectrum of compound **7bF**

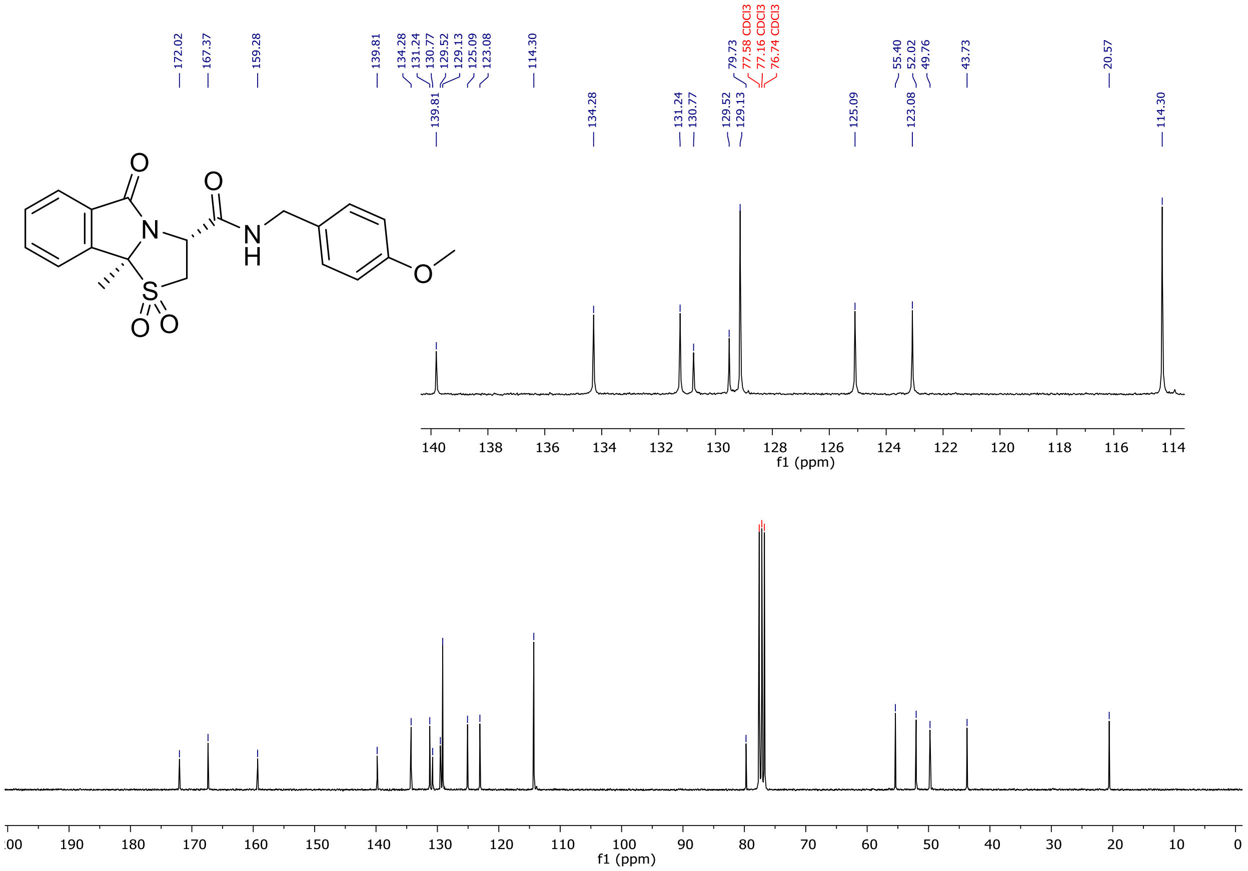

^{13}C NMR spectrum of compound **7bF**

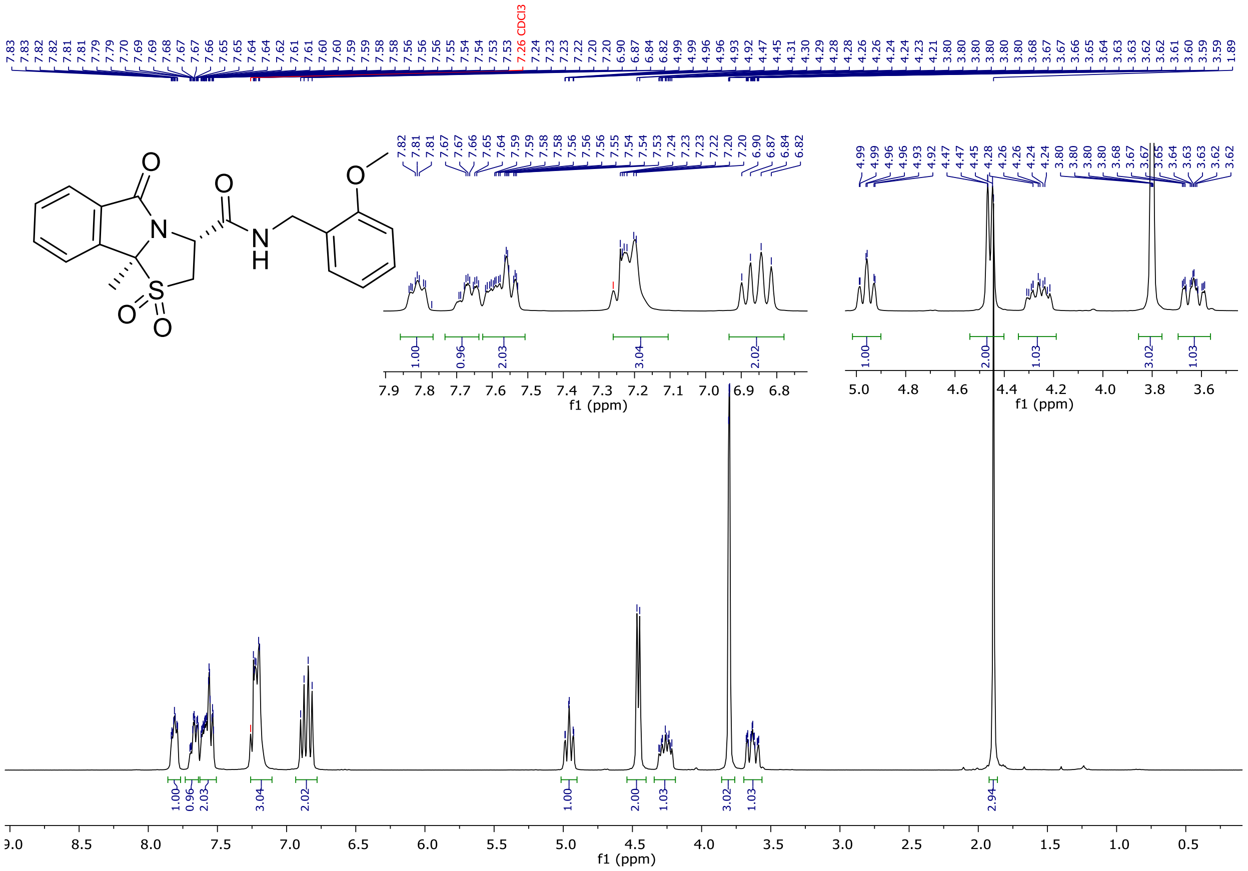

^{1}H NMR spectrum of compound **7bG**

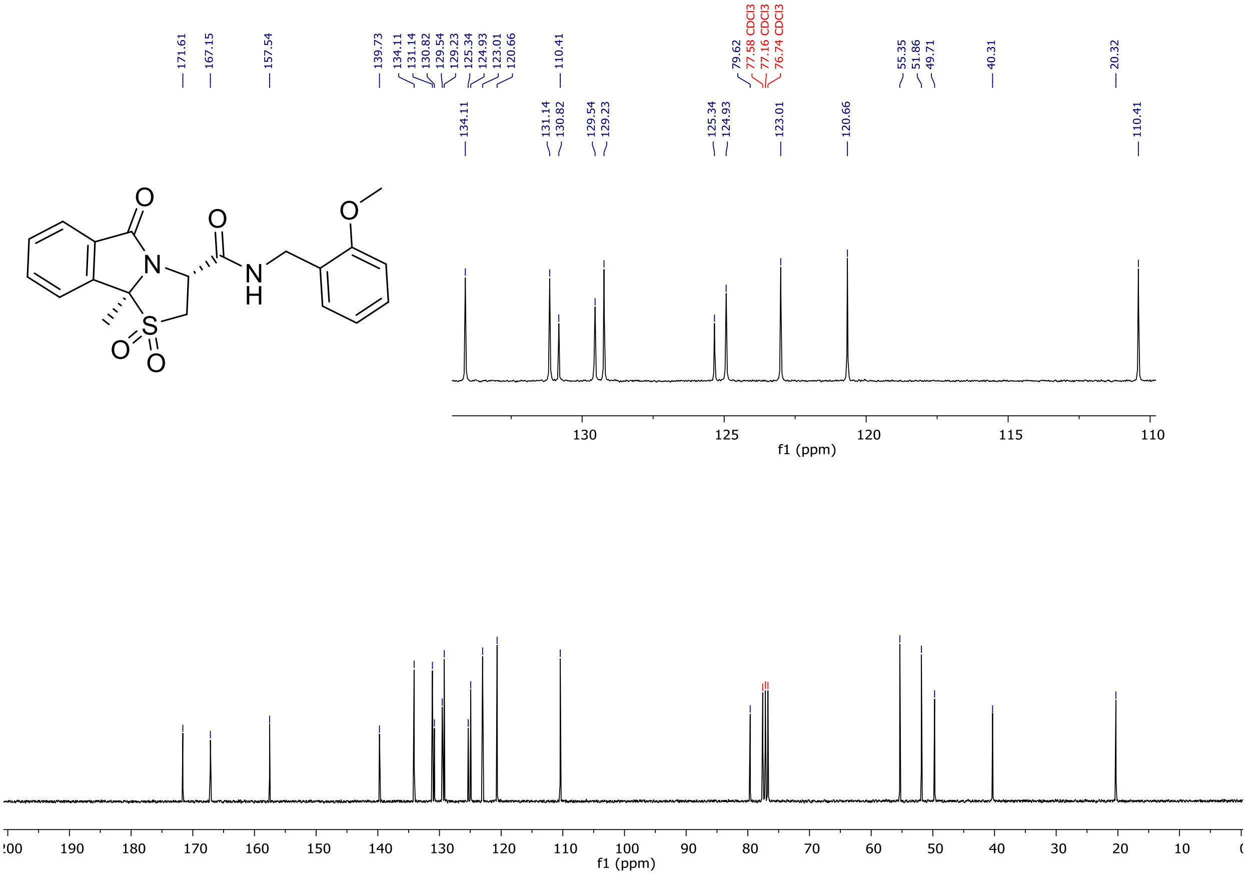

^{13}C NMR spectrum of compound **7bG**

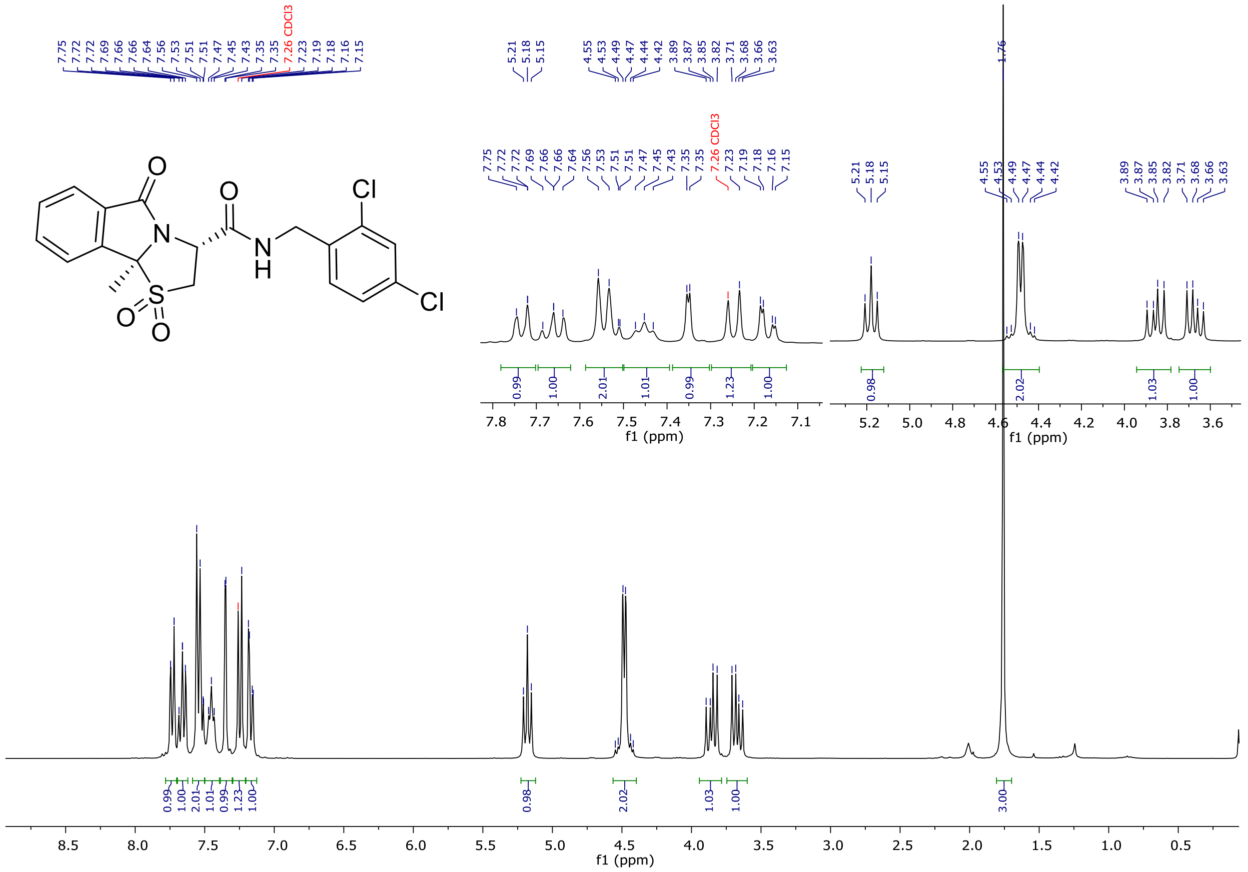

^{1}H NMR spectrum of compound **7bH**

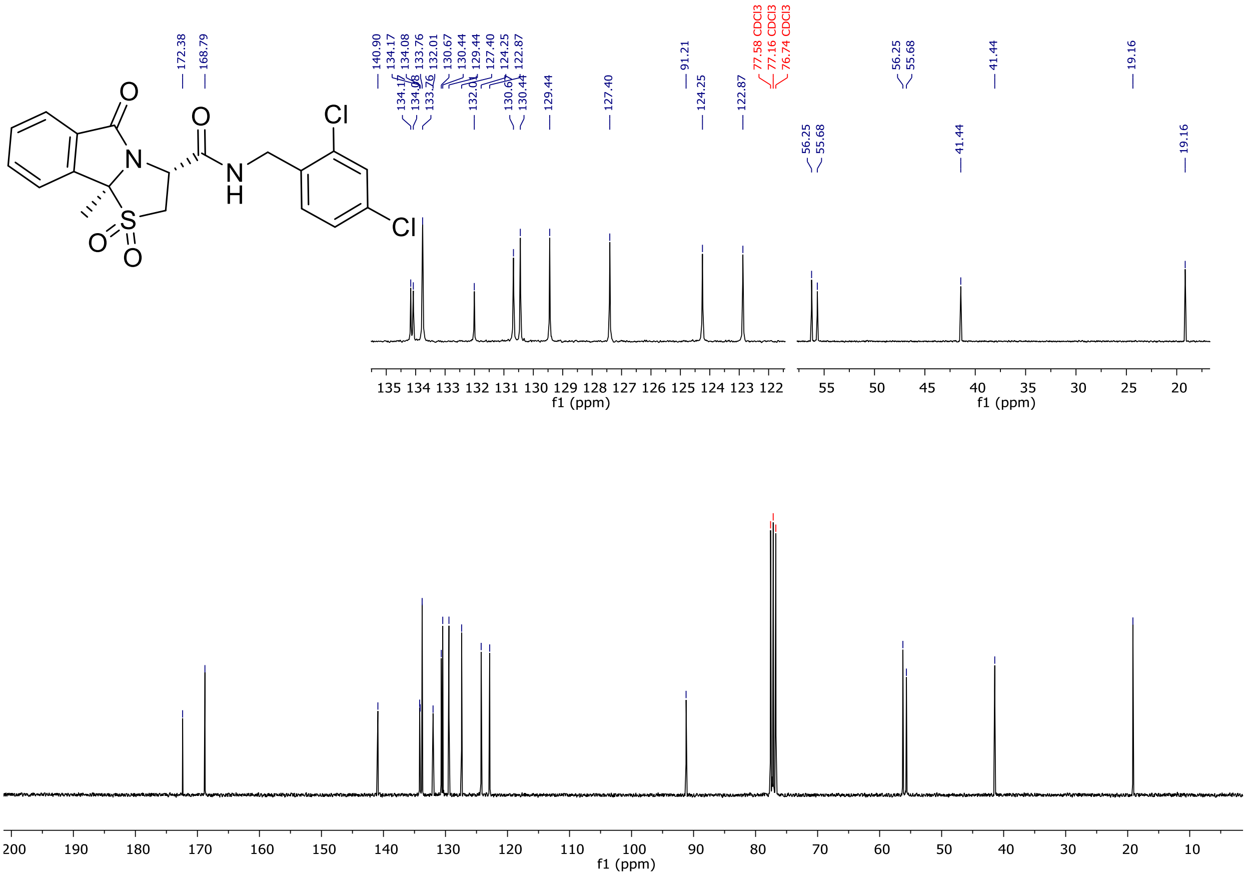

^{13}C NMR spectrum of compound **7bH**

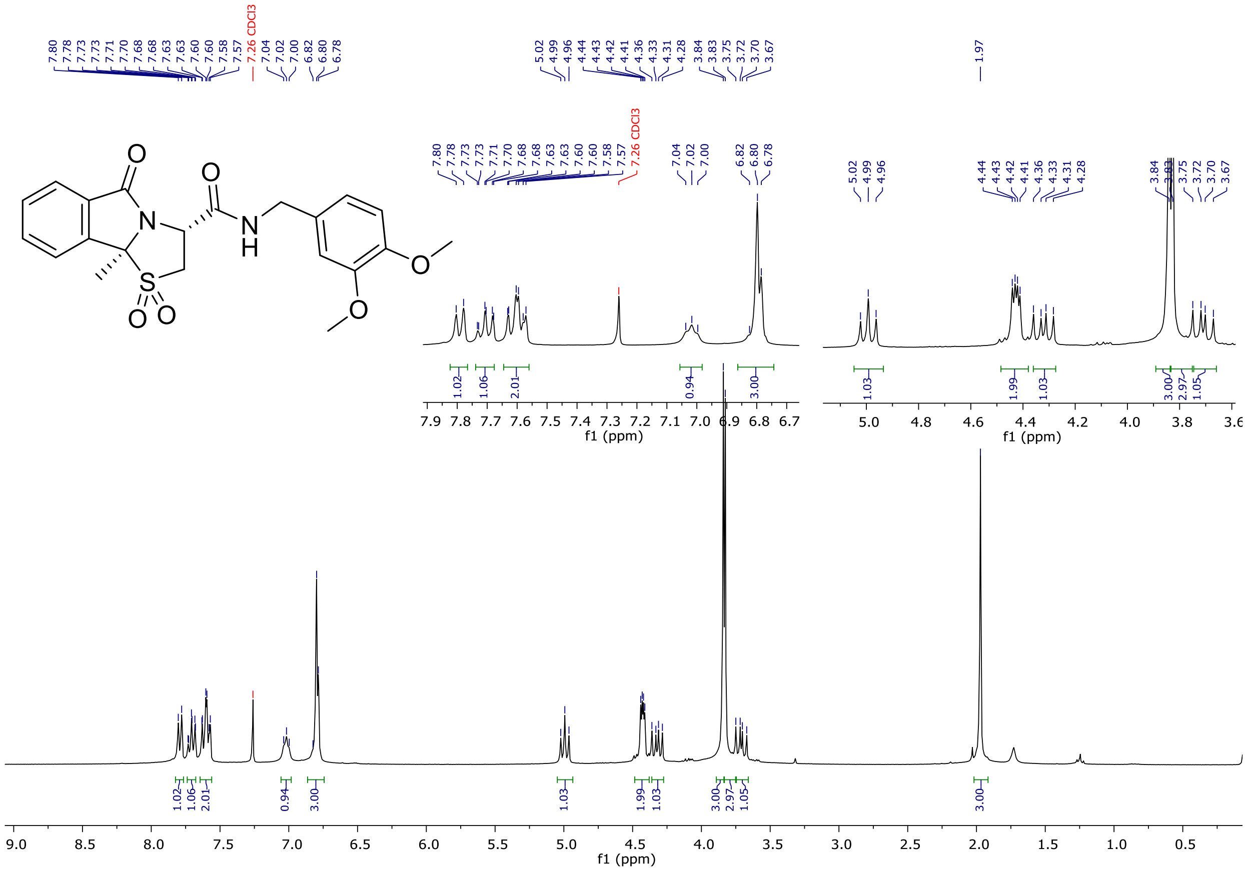

^{1}H NMR spectrum of compound **7bI**

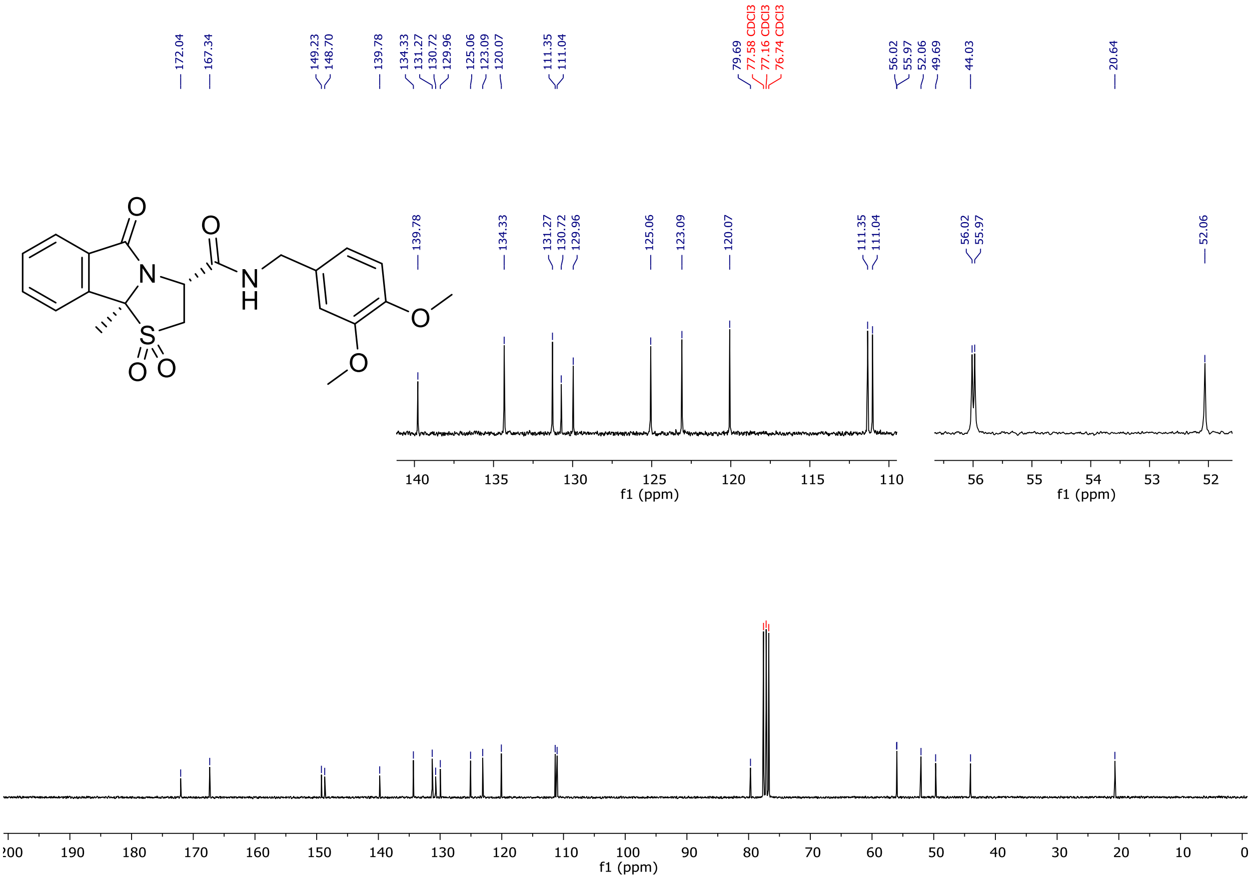

^{13}C NMR spectrum of compound **7bI**

VIII. 2D-NMR Analysis of the sulfoxide product 5Af

2D NMR Analysis of Product 5aF

- Arbitrary product numbering -

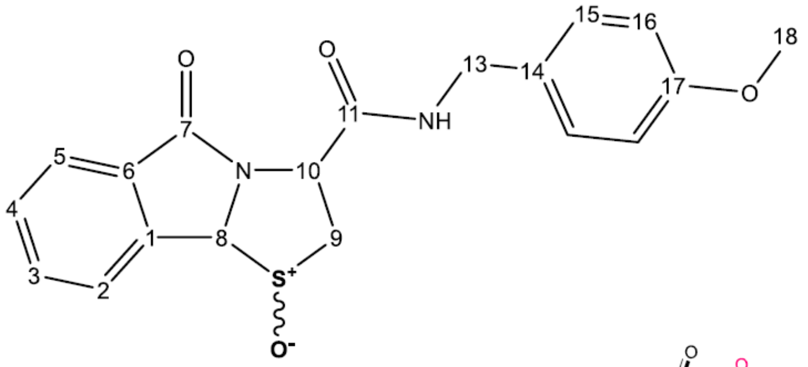

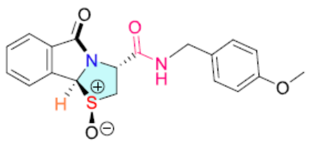

1°)- COSY Correlations: Attribution of H_8, H_9, H_{10}, H_{13} and NH signals

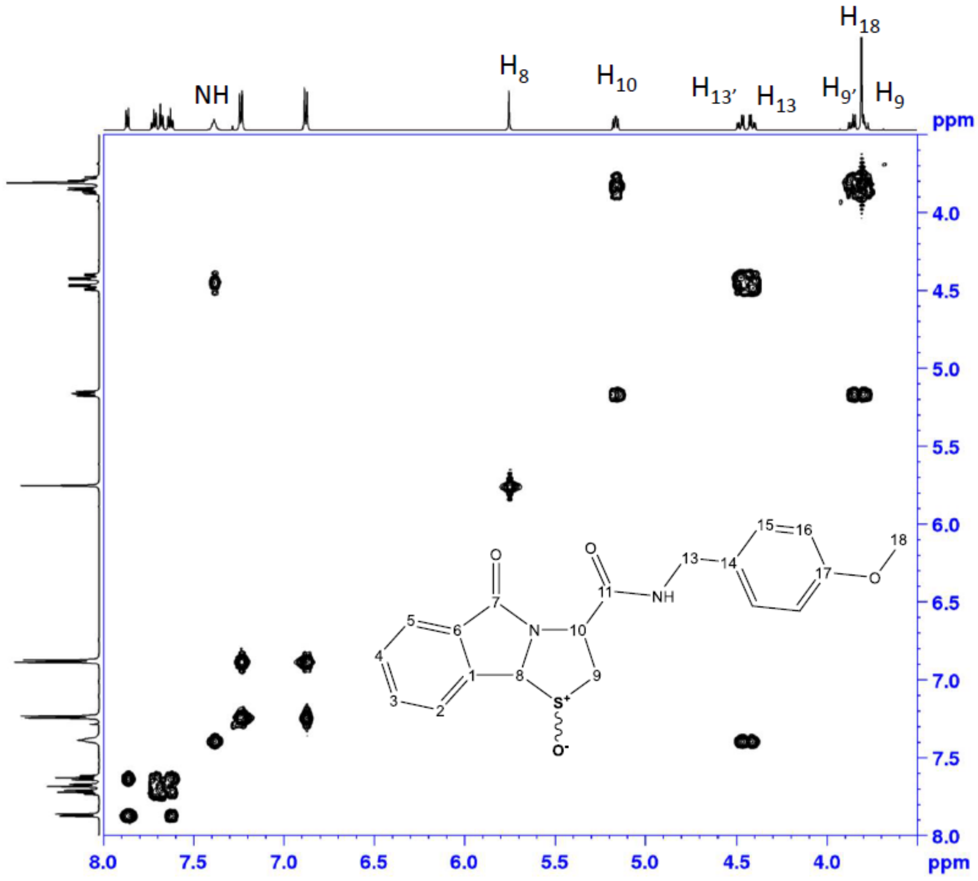

2°)- NOEs Correlations allowing the of H_2 and H_{16} signals

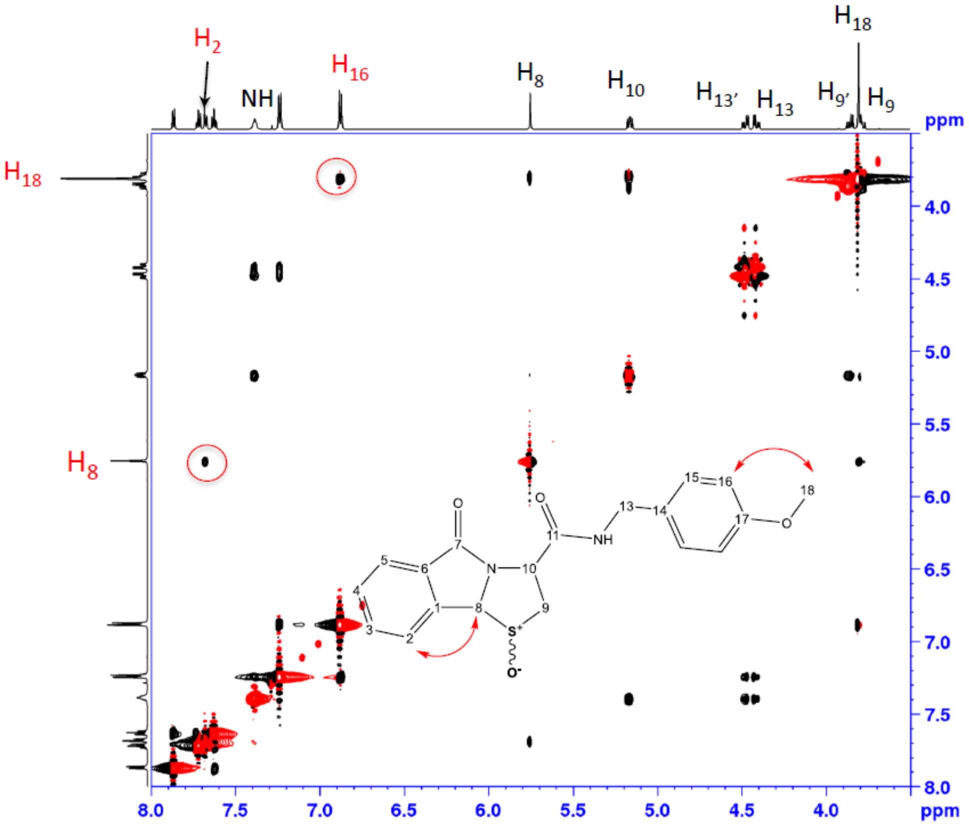

3°)- COSY Correlations: Attribution of all signals

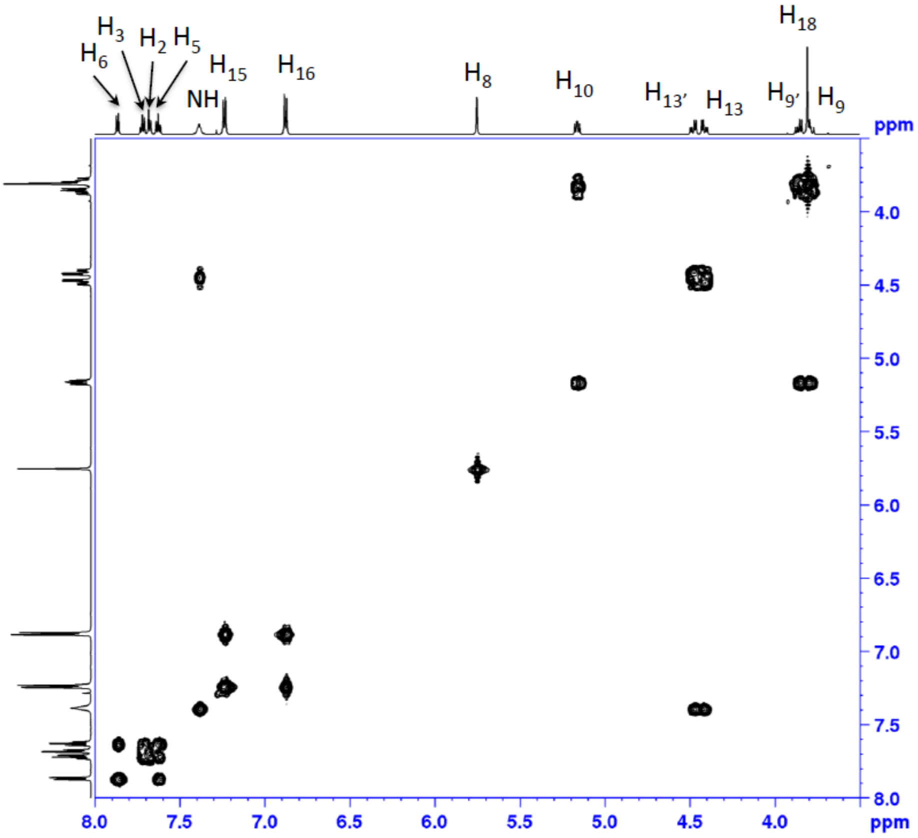

4°)- NOEs Correlations allowing the orientation of the H_8 and H_{10} protons

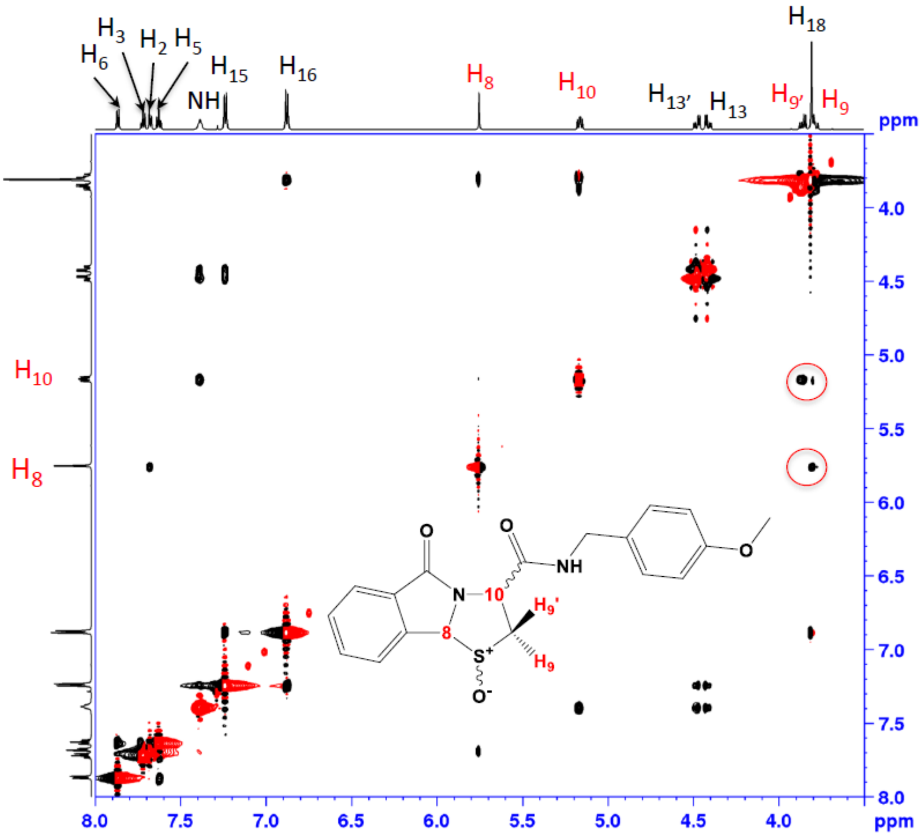

5°)- NOEs Correlations allowing the orientation of the H_8 and H_9 protons

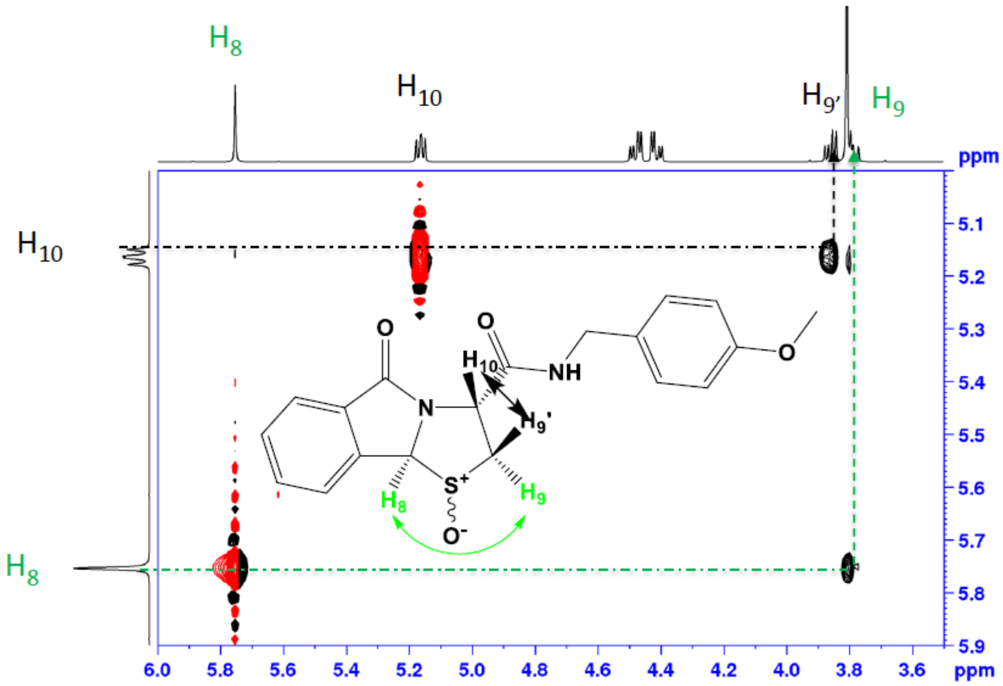

IX. X-Ray data including Stick drawing of compound 5bA (named in the following AD1065)

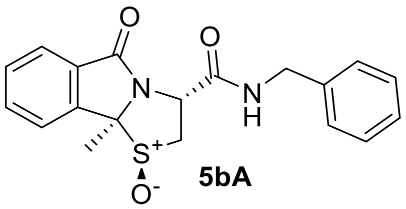

5bA

Table 1: Crystal data and structure refinement for AD1065.	
Identification code	AD1065
Empirical formula	$C_{19}H_{18}N_2O_3S$
Formula weight	354.41
Temperature/K	100
Crystal system	monoclinic
Space group	$P2_1$
a/Å	7.30520(10)
b/Å	10.5288(2)
c/Å	11.2949(2)
α/°	90
β/°	101.2400(10)
γ/°	90
Volume/Å^3	852.08(3)
Z	2
ρ_{calc}g/cm^3	1.381
μ/mm^{-1}	1.866
F(000)	372.0
Crystal size/mm^3	0.21 × 0.16 × 0.12
Radiation	CuKα (λ = 1.54186)
2Θ range for data collection/°	7.98 to 143.608
Index ranges	-7 ≤ h ≤ 9, -10 ≤ k ≤ 12, -11 ≤ l ≤ 13
Reflections collected	37896
Independent reflections	3021 [R_{int} = 0.0219, R_{sigma} = 0.0102]
Data/restraints/parameters	3021/1/227
Goodness-of-fit on F^2	1.033
Final R indexes [I>=2σ (I)]	R_1 = 0.0252, wR_2 = 0.0669
Final R indexes [all data]	R_1 = 0.0256, wR_2 = 0.0675
Largest diff. peak/hole / e Å^{-3}	0.26/-0.21
Flack parameter	0.012(14)

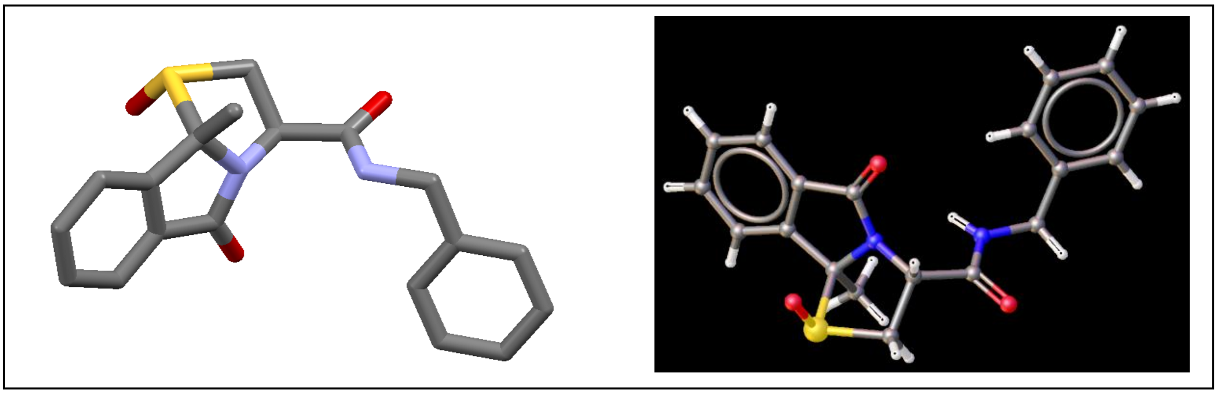

Stick drawings of compound **5bA** [**AD1065**].

Table 2: Fractional Atomic Coordinates (×10^4) and Equivalent Isotropic Displacement Parameters (Å^2×10^3) for compound **5bA** [**AD1065**]. U_{eq} is defined as 1/3 of of the trace of the orthogonalised U_{IJ} tensor.				
**Atom**	***x***	***y***	***z***	**U(eq)**
S1	7814.8(7)	6992.7(5)	5255.7(4)	17.00(14)
O1	1854(2)	6098.1(17)	4275.1(14)	22.0(4)
O2	5395(2)	5847.8(16)	1261.1(14)	22.0(4)
O3	6459(2)	7942.7(16)	5568.3(14)	21.2(4)
N1	4962(3)	5610.1(18)	4393.2(16)	15.8(4)
N2	4456(3)	4234.4(19)	2339.8(17)	17.2(4)
C1	3370(3)	5783(2)	4863.4(19)	17.1(4)
C2	3928(3)	5506(2)	6170(2)	18.0(5)
C3	2836(3)	5453(2)	7047(2)	22.0(5)
C4	3723(4)	5150(3)	8219(2)	25.4(5)
C5	5636(4)	4918(3)	8495(2)	25.6(5)
C6	6727(4)	4983(3)	7612(2)	22.9(5)
C7	5839(3)	5281(2)	6448(2)	18.6(5)
C8	6622(3)	5415(2)	5320.2(19)	16.5(5)
C9	7894(3)	4332(2)	5082(2)	21.0(5)
C10	5255(3)	6268(2)	3311.4(18)	16.4(4)
C11	7222(3)	6882(2)	3618.3(18)	18.1(4)
C12	5046(3)	5415(2)	2197.1(19)	16.5(5)
C13	4012(3)	3347(2)	1344(2)	18.6(5)
C14	1946(3)	3090(2)	942(2)	18.7(5)
C15	634(3)	3504(2)	1594(2)	21.9(5)
C16	-1237(3)	3198(3)	1214(2)	28.6(6)
C17	-1821(4)	2485(3)	183(2)	26.8(6)
C18	-517(3)	2077(3)	-478(2)	27.6(5)
C19	1354(3)	2379(2)	-99(2)	24.0(5)

Table 3: Anisotropic Displacement Parameters ($Å^2×10^3$) for compound **5bA** [**AD1065**]. The Anisotropic displacement factor exponent takes the form: $-2\pi^2[h^2a^{*2}U_{11}+2hka^*b^*U_{12}+…]$.						
**Atom**	**U_{11}**	**U_{22}**	**U_{33}**	**U_{23}**	**U_{13}**	**U_{12}**
S1	19.3(2)	15.7(3)	16.3(2)	-1.8(2)	4.09(18)	-1.5(2)
O1	19.3(8)	24.8(9)	21.8(8)	0.2(7)	3.7(6)	1.3(7)
O2	27.8(8)	23.4(10)	15.7(7)	1.8(7)	6.6(6)	-2.3(7)
O3	27.2(9)	16.7(9)	20.0(8)	-1.6(6)	5.9(6)	1.6(7)
N1	18.8(9)	14.8(10)	13.9(8)	0.9(7)	3.4(7)	-0.4(7)
N2	19.9(9)	19.3(11)	13.0(9)	-0.4(7)	5.0(7)	-0.9(8)
C1	21.7(10)	12.7(11)	18(1)	-2.0(9)	6.4(8)	-1.4(9)
C2	23.4(11)	13.4(12)	17.9(10)	-1.9(8)	5.8(8)	-2.4(9)
C3	27.8(12)	19.3(13)	21.0(11)	-3.4(9)	9.6(9)	-5.1(9)
C4	38.2(14)	21.5(13)	19.7(11)	-1.5(10)	13.8(10)	-5.9(11)
C5	40.1(14)	20.8(13)	15.9(11)	2.1(9)	5.4(10)	-1.5(10)
C6	28.5(13)	19.2(13)	20.4(11)	1.4(9)	3.1(10)	0.7(10)
C7	25.3(11)	13.8(12)	17.7(10)	-0.9(8)	6.4(9)	-2.1(9)
C8	20.2(11)	13.9(12)	15.3(10)	-0.9(8)	2.8(8)	-2.1(8)
C9	23.6(12)	15.2(13)	24.3(11)	-1.1(9)	5.2(9)	2.9(9)
C10	18.8(10)	15.5(12)	15.5(10)	0.9(9)	5.1(8)	0.3(8)
C11	22.5(10)	17.7(12)	15.2(9)	-1.8(9)	6.3(8)	-4.1(10)
C12	13.7(10)	19.8(13)	16(1)	2.1(9)	2.5(8)	3.8(8)
C13	19.8(11)	20.3(13)	16.6(10)	-2.6(9)	5.6(8)	-0.4(9)
C14	20.9(11)	16.2(11)	19.1(10)	2.3(9)	4.6(8)	0.9(9)
C15	22.4(12)	24.0(13)	19.7(11)	-4.4(10)	4.9(9)	-0.3(10)
C16	21.0(12)	36.1(16)	29.9(13)	-4.8(11)	7.9(10)	2.6(11)
C17	19.7(11)	27.1(14)	32.2(13)	-1.4(11)	1.3(10)	-1.1(10)
C18	26.4(12)	25.3(13)	28.7(11)	-9.5(11)	-0.1(9)	-2.3(12)
C19	22.7(12)	26.6(14)	23.2(11)	-6.9(10)	5.2(9)	2.1(9)

Table 4: Bond Lengths for compound **5bA** [**AD1065**].					
**Atom**	**Atom**	**Length/Å**	**Atom**	**Atom**	**Length/Å**
S1	O3	1.4976(17)	C4	C5	1.393(4)
S1	C8	1.883(2)	C5	C6	1.394(3)
S1	C11	1.819(2)	C6	C7	1.384(3)
O1	C1	1.221(3)	C7	C8	1.501(3)
O2	C12	1.223(3)	C8	C9	1.528(3)
N1	C1	1.381(3)	C10	C11	1.552(3)

N1	C8	1.454(3)		C10	C12	1.530(3)
N1	C10	1.456(3)		C13	C14	1.513(3)
N2	C12	1.336(3)		C14	C15	1.387(3)
N2	C13	1.450(3)		C14	C19	1.390(3)
C1	C2	1.482(3)		C15	C16	1.388(4)
C2	C3	1.388(3)		C16	C17	1.381(4)
C2	C7	1.390(3)		C17	C18	1.388(4)
C3	C4	1.392(3)		C18	C19	1.387(3)

Table 5: Bond Angles for compound **5bA** [**AD1065**].

**Atom**	**Atom**	**Atom**	**Angle/˚**		**Atom**	**Atom**	**Atom**	**Angle/˚**
O3	S1	C8	104.32(10)		N1	C8	C7	102.84(18)
O3	S1	C11	104.18(10)		N1	C8	C9	115.42(18)
C11	S1	C8	87.86(10)		C7	C8	S1	111.81(15)
C1	N1	C8	112.84(17)		C7	C8	C9	115.11(19)
C1	N1	C10	122.16(18)		C9	C8	S1	110.45(15)
C8	N1	C10	115.66(17)		N1	C10	C11	106.97(17)
C12	N2	C13	122.35(19)		N1	C10	C12	113.78(18)
O1	C1	N1	124.7(2)		C12	C10	C11	111.60(17)
O1	C1	C2	129.8(2)		C10	C11	S1	106.28(13)
N1	C1	C2	105.57(18)		O2	C12	N2	125.3(2)
C3	C2	C1	129.3(2)		O2	C12	C10	119.2(2)
C3	C2	C7	121.6(2)		N2	C12	C10	115.48(19)
C7	C2	C1	109.08(19)		N2	C13	C14	114.00(18)
C2	C3	C4	117.5(2)		C15	C14	C13	122.5(2)
C3	C4	C5	120.9(2)		C15	C14	C19	119.0(2)
C4	C5	C6	121.3(2)		C19	C14	C13	118.5(2)
C7	C6	C5	117.6(2)		C14	C15	C16	120.2(2)
C2	C7	C8	108.95(19)		C17	C16	C15	120.7(2)
C6	C7	C2	121.1(2)		C16	C17	C18	119.3(2)
C6	C7	C8	129.9(2)		C19	C18	C17	120.1(2)
N1	C8	S1	100.09(14)		C18	C19	C14	120.6(2)

Table 6: Hydrogen Atom Coordinates (Å×10[4]) and Isotropic Displacement Parameters (Å[2]×10[3]) for **5bA** [**AD1065**].				
**Atom**	***x***	***y***	***z***	**U(eq)**
H2	4331.54	3986.7	3064.08	21
H3	1532.88	5616.75	6854.75	26
H4	3013.22	5100.59	8838.17	30
H5	6209.04	4711.64	9300.91	31
H6	8032.29	4827.81	7802.42	27
H9A	8428.8	4535.93	4374.18	31
H9B	7168	3546.04	4933.81	31
H9C	8901.46	4220.68	5787	31
H10	4311.85	6965.65	3132.66	20
H11A	7205.37	7736.56	3250.24	22
H11B	8143.99	6350.17	3310.37	22
H13A	4656.87	2533.12	1585.77	22
H13B	4503.48	3685.06	650.02	22
H15	1016.86	3998.27	2302.1	26
H16	-2125.38	3482.78	1667.11	34
H17	-3102.18	2275.48	-71.32	32
H18	-905.82	1590.3	-1190.73	33
H19	2240.07	2096.18	-554.92	29

Experimental

Single crystals of $C_{19}H_{18}N_2O_3S$ [AD1065] were []. A suitable crystal was selected and [] on a Stoe Stadivari with Xenocs Genix3D Cu HF diffractometer. The crystal was kept at 100 K during data collection. Using Olex2 [1], the structure was solved with the ShelXT [2] structure solution program using Intrinsic Phasing and refined with the ShelXL [3] refinement package using Least Squares minimisation.

1. Dolomanov, O. V., Bourhis, L. J., Gildea, R. J., Howard, J. A. K. and Puschmann, H. (2009), *J. Appl. Cryst.* **42**, 339-341.
2. Sheldrick, G. M. (2015). *Acta Cryst.* **A71**, 3-8.
3. Sheldrick, G. M. (2015). *Acta Cryst.* **C71**, 3-8.

Crystal structure determination of compound 5aB [AD1065]

Crystal Data for $C_{19}H_{18}N_2O_3S$ (M =354.41 g/mol): monoclinic, space group $P2_1$ (no. 4), a = 7.30520(10) Å, b = 10.5288(2) Å, c = 11.2949(2) Å, β = 101.2400(10)°, V = 852.08(3) Å^3, Z = 2, T = 100 K, μ(CuKα) = 1.866 mm^{-1}, $Dcalc$ = 1.381 g/cm^3, 37896 reflections measured (7.98° ≤ 2Θ ≤ 143.608°), 3021 unique (R_{int} = 0.0219, R_{sigma} = 0.0102) which were used in all calculations. The final R_1 was 0.0252 (I > 2σ(I)) and wR_2 was 0.0675 (all data).

Refinement model description

Number of restraints - 1, number of constraints - unknown.

Details:

1. Fixed Uiso
   At 1.2 times of:
      All C(H) groups, All C(H,H) groups, All N(H) groups
   At 1.5 times of:
      All C(H,H,H) groups

2.a Ternary CH refined with riding coordinates:
   C10(H10)

2.b Secondary CH2 refined with riding coordinates:
   C11(H11A,H11B), C13(H13A,H13B)

2.c Aromatic/amide H refined with riding coordinates:
   N2(H2),
   C3(H3), C4(H4), C5(H5), C6(H6), C15(H15), C16(H16), C17(H17), C18(H18), C19(H19)

2.d Idealised Me refined as rotating group:
   C9(H9A,H9B,H9C)

X. NCI One Dose Mean Graph of sulfides 4a(A-J) and 4b(A-J)

The entire series of sulfides regrouping twenty synthesized FTI-candidates (**4aA-J**, **4bA-J**) were selected by the National Cancer Institute (NCI) for evaluation of their antiproliferative activity against 60 cancer cell lines (see the followings twenty pages for full evaluation reports).

Investigated molecules showed specific cytostatic activity against NCI-H522 non-small cell lung cancer cells. Indeed, seventeen molecules engendered 20-45% inhibition of the growth of these cells at a concentration of 10 µM. The most active compound on cancer cells was molecule **4bI** which was also the best inhibitor against farnesyltransferase (IC_{50} = 25.1 nM) among the twenty selected molecules. This methylated compound **4bI** also inhibited the growth of K-562 leukemia (34% inhibition), UACC-257 melanoma (27% inhibition), MDA-MB-468 breast cancer (30% inhibition) and UO-31 renal cancer (20% inhibition) cells. In the non-methylated series, the analogue **4aI** proved to be the most cytostatic compound inhibiting 36% of the NCI-H522 cells. Such difference between IC_{50} on human FTase in the nanomolar range and IC_{50} on cancer cell growth in the micromolar range is characteristic to potent FTIs and was reported previously on different series of compounds (see for example reference [37] in the references part in the text).

Of interest, none of the tested compounds at a high single dose (10 µM) in the full NCI 60 cell panel has shown a cytotoxic profile. This result allied to the fact that many of the tested compounds show high inhibitory potential on FTase raise good hope for further investigation of these series as agents fighting progeria.

Developmental Therapeutics Program	NSC: D-802583 / 1	Conc: 1.00E-5 Molar	Test Date: Dec 11, 2017
One Dose Mean Graph	Experiment ID: 1712OS12	Product 4aA	Report Date: Jan 06, 2018

Panel/Cell Line	Growth Percent
Leukemia	
CCRF-CEM	96.52
HL-60(TB)	102.43
K-562	101.44
MOLT-4	95.69
RPMI-8226	105.32
Non-Small Cell Lung Cancer	
A549/ATCC	97.86
EKVX	105.39
HOP-62	97.31
HOP-92	87.72
NCI-H226	86.94
NCI-H23	100.85
NCI-H322M	102.34
NCI-H522	79.32
Colon Cancer	
HCC-2998	101.83
HCT-116	87.66
HCT-15	103.68
HT29	99.44
KM12	110.51
SW-620	102.46
CNS Cancer	
SF-268	90.56
SF-295	99.96
SF-539	102.43
SNB-19	96.70
SNB-75	84.04
U251	97.80
Melanoma	
LOX IMVI	97.27
MALME-3M	111.55
M14	94.47
MDA-MB-435	106.84
SK-MEL-2	105.03
SK-MEL-28	110.09
SK-MEL-5	114.63
UACC-257	96.60
UACC-62	97.72
Ovarian Cancer	
IGROV1	105.80
OVCAR-3	112.79
OVCAR-4	111.39
OVCAR-5	91.72
OVCAR-8	101.18
NCI/ADR-RES	99.86
SK-OV-3	102.04
Renal Cancer	
786-0	85.46
A498	102.15
ACHN	102.35
CAKI-1	94.90
RXF 393	114.31
SN12C	98.99
TK-10	96.56
UO-31	91.66
Prostate Cancer	
PC-3	96.96
DU-145	111.90
Breast Cancer	
MCF7	87.71
MDA-MB-231/ATCC	100.79
HS 578T	107.68
BT-549	93.57
MDA-MB-468	105.86
Mean	99.75
Delta	20.43
Range	35.31

Mean Growth Percent - Growth Percent

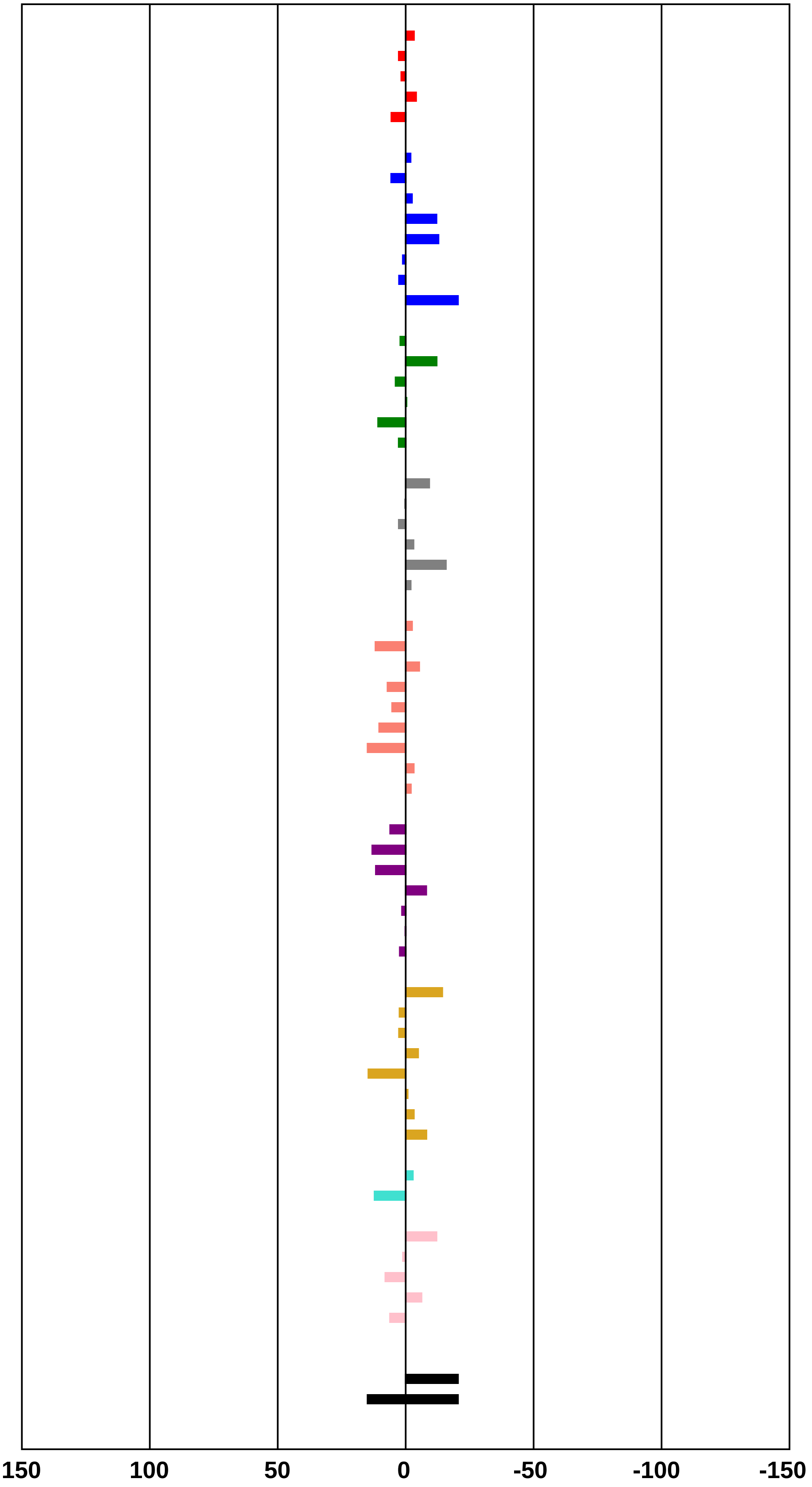

Developmental Therapeutics Program	NSC: D-802588 / 1	Conc: 1.00E-5 Molar	Test Date: Dec 11, 2017
One Dose Mean Graph	Experiment ID: 1712OS12	Product 4aB	Report Date: Jan 06, 2018

Panel/Cell Line	Growth Percent
**Leukemia**	
CCRF-CEM	98.35
HL-60(TB)	99.18
K-562	85.58
MOLT-4	97.90
RPMI-8226	91.88
**Non-Small Cell Lung Cancer**	
A549/ATCC	93.61
EKVX	106.71
HOP-62	93.04
HOP-92	85.84
NCI-H226	96.85
NCI-H23	103.48
NCI-H322M	97.04
NCI-H522	80.19
**Colon Cancer**	
HCC-2998	107.23
HCT-116	91.11
HCT-15	94.68
HT29	94.05
KM12	106.54
SW-620	99.75
**CNS Cancer**	
SF-268	101.56
SF-295	96.38
SF-539	104.22
SNB-19	101.75
SNB-75	86.13
U251	99.57
**Melanoma**	
LOX IMVI	100.95
MALME-3M	112.75
M14	93.75
MDA-MB-435	107.16
SK-MEL-2	101.94
SK-MEL-28	100.81
SK-MEL-5	100.94
UACC-257	96.30
UACC-62	86.77
**Ovarian Cancer**	
IGROV1	99.24
OVCAR-3	112.31
OVCAR-4	111.65
OVCAR-5	96.32
OVCAR-8	100.80
NCI/ADR-RES	98.43
SK-OV-3	104.13
**Renal Cancer**	
786-0	89.50
A498	102.03
ACHN	104.51
CAKI-1	98.96
RXF 393	110.05
SN12C	107.39
TK-10	94.50
UO-31	95.36
**Prostate Cancer**	
PC-3	89.38
DU-145	111.93
**Breast Cancer**	
MCF7	89.74
MDA-MB-231/ATCC	100.50
HS 578T	113.21
BT-549	90.69
MDA-MB-468	94.29
Mean	98.73
Delta	18.54
Range	33.02

Mean Growth Percent - Growth Percent

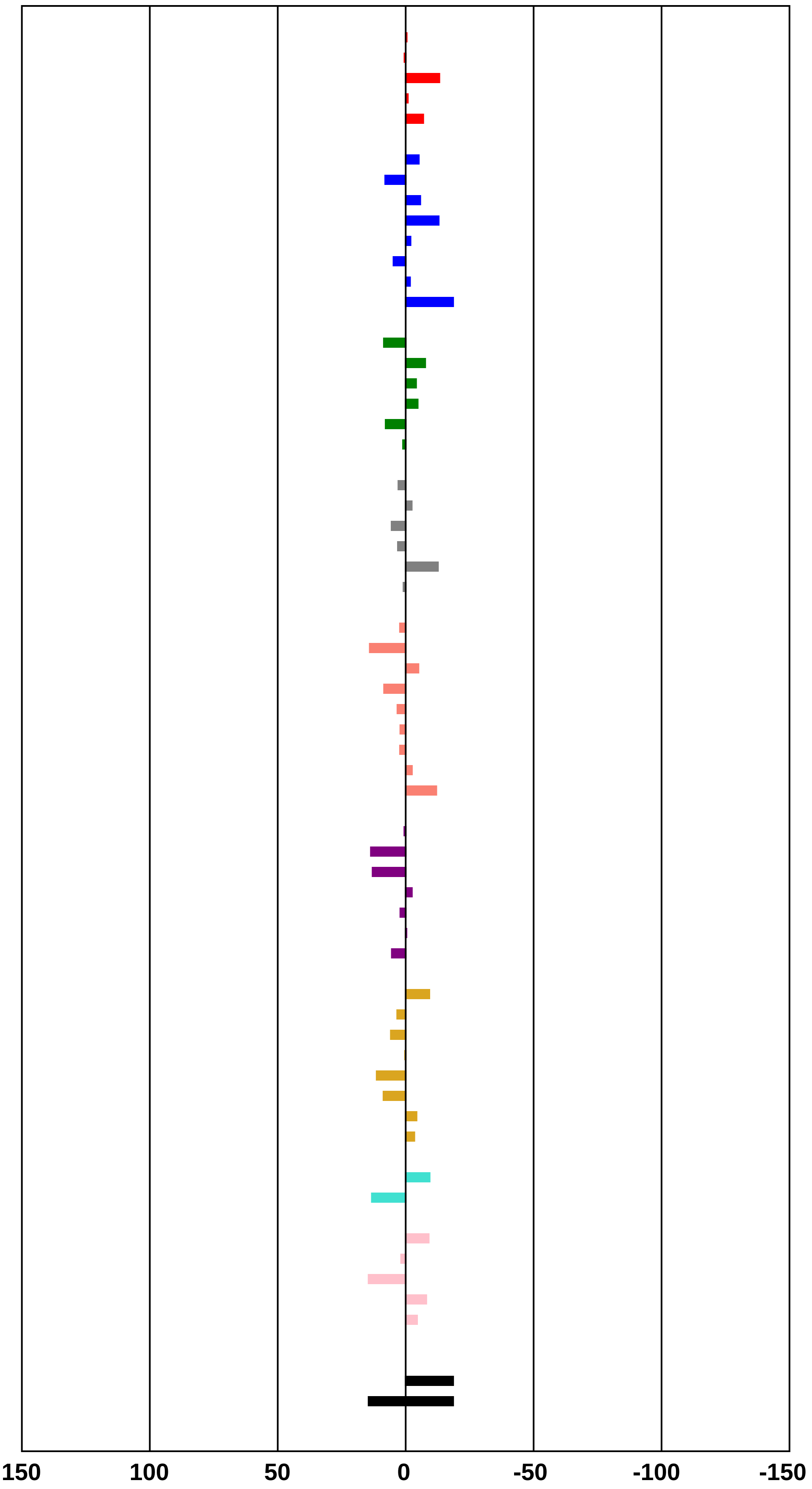

Developmental Therapeutics Program

One Dose Mean Graph

**NSC:** D-802584 / 1	**Conc:** 1.00E-5 Molar	**Test Date:** Dec 11, 2017
**Experiment ID:** 1712OS12	Product 4aC	**Report Date:** Jan 06, 2018

Panel/Cell Line	Growth Percent
Leukemia	
CCRF-CEM	93.25
HL-60(TB)	101.08
K-562	94.12
MOLT-4	85.46
RPMI-8226	87.69
Non-Small Cell Lung Cancer	
A549/ATCC	90.42
EKVX	99.35
HOP-62	87.37
HOP-92	77.56
NCI-H226	95.46
NCI-H23	105.15
NCI-H322M	103.27
NCI-H522	78.18
Colon Cancer	
HCC-2998	111.38
HCT-116	87.63
HCT-15	103.73
HT29	91.65
KM12	102.90
SW-620	104.34
CNS Cancer	
SF-268	93.86
SF-295	94.85
SF-539	95.88
SNB-19	95.80
SNB-75	85.21
U251	91.06
Melanoma	
LOX IMVI	102.58
MALME-3M	108.73
M14	95.62
MDA-MB-435	102.87
SK-MEL-2	99.87
SK-MEL-28	99.12
SK-MEL-5	107.31
UACC-257	86.31
UACC-62	88.82
Ovarian Cancer	
IGROV1	98.93
OVCAR-3	106.48
OVCAR-4	105.74
OVCAR-5	83.18
OVCAR-8	94.34
NCI/ADR-RES	99.79
SK-OV-3	85.91
Renal Cancer	
786-0	86.33
A498	98.14
ACHN	99.65
CAKI-1	97.48
RXF 393	115.46
SN12C	101.45
TK-10	108.86
UO-31	74.84
Prostate Cancer	
PC-3	85.22
DU-145	107.34
Breast Cancer	
MCF7	90.35
MDA-MB-231/ATCC	91.35
HS 578T	104.53
BT-549	96.07
MDA-MB-468	112.27
Mean	96.28
Delta	21.44
Range	40.62

Mean Growth Percent - Growth Percent

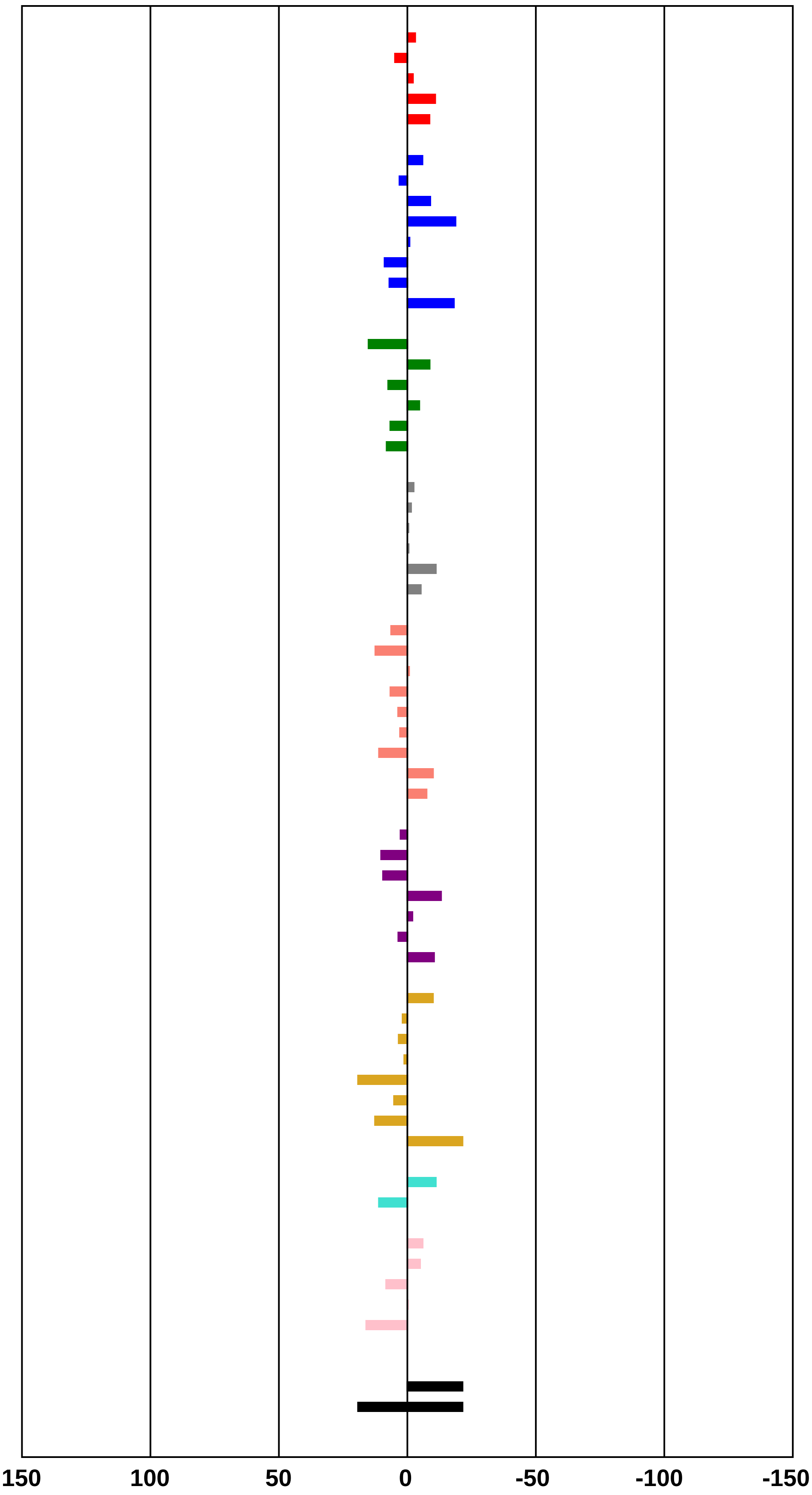

Developmental Therapeutics Program One Dose Mean Graph	NSC: D-802586 / 1	Conc: 1.00E-5 Molar	Test Date: Dec 11, 2017
	Experiment ID: 1712OS12	Product 4aD	Report Date: Jan 06, 2018

Panel/Cell Line	Growth Percent
**Leukemia**	
CCRF-CEM	99.20
HL-60(TB)	96.47
K-562	89.33
MOLT-4	86.93
RPMI-8226	94.54
**Non-Small Cell Lung Cancer**	
A549/ATCC	94.49
EKVX	103.29
HOP-62	85.91
HOP-92	79.43
NCI-H226	100.68
NCI-H23	100.33
NCI-H322M	93.97
NCI-H522	74.73
**Colon Cancer**	
HCC-2998	121.37
HCT-116	91.63
HCT-15	100.16
HT29	91.88
KM12	98.82
SW-620	107.41
**CNS Cancer**	
SF-268	96.16
SF-295	95.03
SF-539	101.49
SNB-19	95.15
SNB-75	75.92
U251	94.74
**Melanoma**	
LOX IMVI	100.26
MALME-3M	106.15
M14	97.50
MDA-MB-435	107.10
SK-MEL-2	94.10
SK-MEL-28	108.89
SK-MEL-5	110.95
UACC-257	89.32
UACC-62	89.84
**Ovarian Cancer**	
IGROV1	95.38
OVCAR-3	104.21
OVCAR-4	105.79
OVCAR-5	97.67
OVCAR-8	94.59
NCI/ADR-RES	92.62
SK-OV-3	88.01
**Renal Cancer**	
786-0	88.74
A498	103.16
ACHN	101.09
CAKI-1	97.61
RXF 393	114.41
SN12C	98.14
TK-10	117.52
UO-31	78.58
**Prostate Cancer**	
PC-3	86.39
DU-145	110.52
**Breast Cancer**	
MCF7	91.11
MDA-MB-231/ATCC	97.44
HS 578T	110.09
BT-549	92.70
MDA-MB-468	98.77
Mean	97.10
Delta	22.37
Range	46.64

Mean Growth Percent - Growth Percent

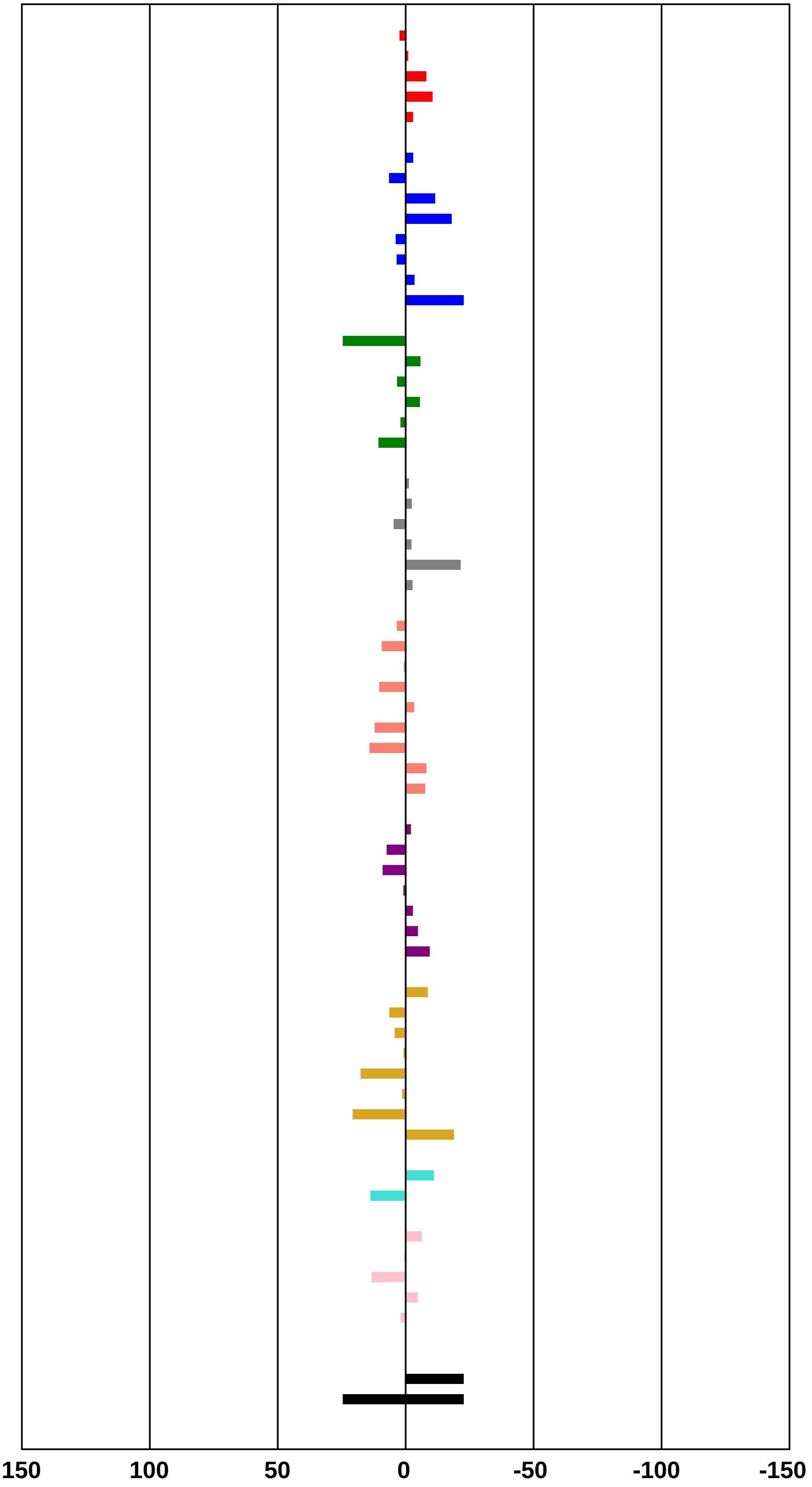

Developmental Therapeutics Program One Dose Mean Graph	**NSC:** D-800143 / 1	**Conc:** 1.00E-5 Molar	**Test Date:** Aug 21, 2017
	**Experiment ID:** 1708OS57	Product 4aE	**Report Date:** Sep 05, 2017

Panel/Cell Line	Growth Percent
Leukemia	
CCRF-CEM	102.31
K-562	85.13
MOLT-4	80.83
RPMI-8226	101.67
SR	84.95
Non-Small Cell Lung Cancer	
A549/ATCC	83.51
EKVX	111.45
HOP-62	96.82
HOP-92	99.32
NCI-H23	99.84
NCI-H322M	105.81
NCI-H460	113.41
NCI-H522	74.09
Colon Cancer	
COLO 205	112.81
HCC-2998	98.50
HCT-116	102.32
HCT-15	105.05
HT29	89.79
KM12	100.40
SW-620	108.22
CNS Cancer	
SF-268	100.53
SF-295	99.74
SF-539	110.14
SNB-19	104.75
SNB-75	100.11
U251	102.42
Melanoma	
LOX IMVI	102.67
MALME-3M	107.90
M14	107.40
MDA-MB-435	107.01
SK-MEL-2	95.73
SK-MEL-28	113.63
SK-MEL-5	106.50
UACC-257	93.82
UACC-62	102.11
Ovarian Cancer	
IGROV1	109.07
OVCAR-3	104.00
OVCAR-4	112.11
OVCAR-5	113.91
OVCAR-8	100.28
NCI/ADR-RES	106.48
SK-OV-3	110.57
Renal Cancer	
786-0	102.72
A498	92.71
ACHN	110.85
CAKI-1	96.42
RXF 393	101.84
SN12C	105.19
TK-10	89.04
UO-31	97.14
Prostate Cancer	
PC-3	98.06
DU-145	104.21
Breast Cancer	
MCF7	98.52
MDA-MB-231/ATCC	107.16
HS 578T	98.68
BT-549	110.89
T-47D	103.32
MDA-MB-468	106.71
Mean	101.56
Delta	27.47
Range	39.82

Mean Growth Percent - Growth Percent

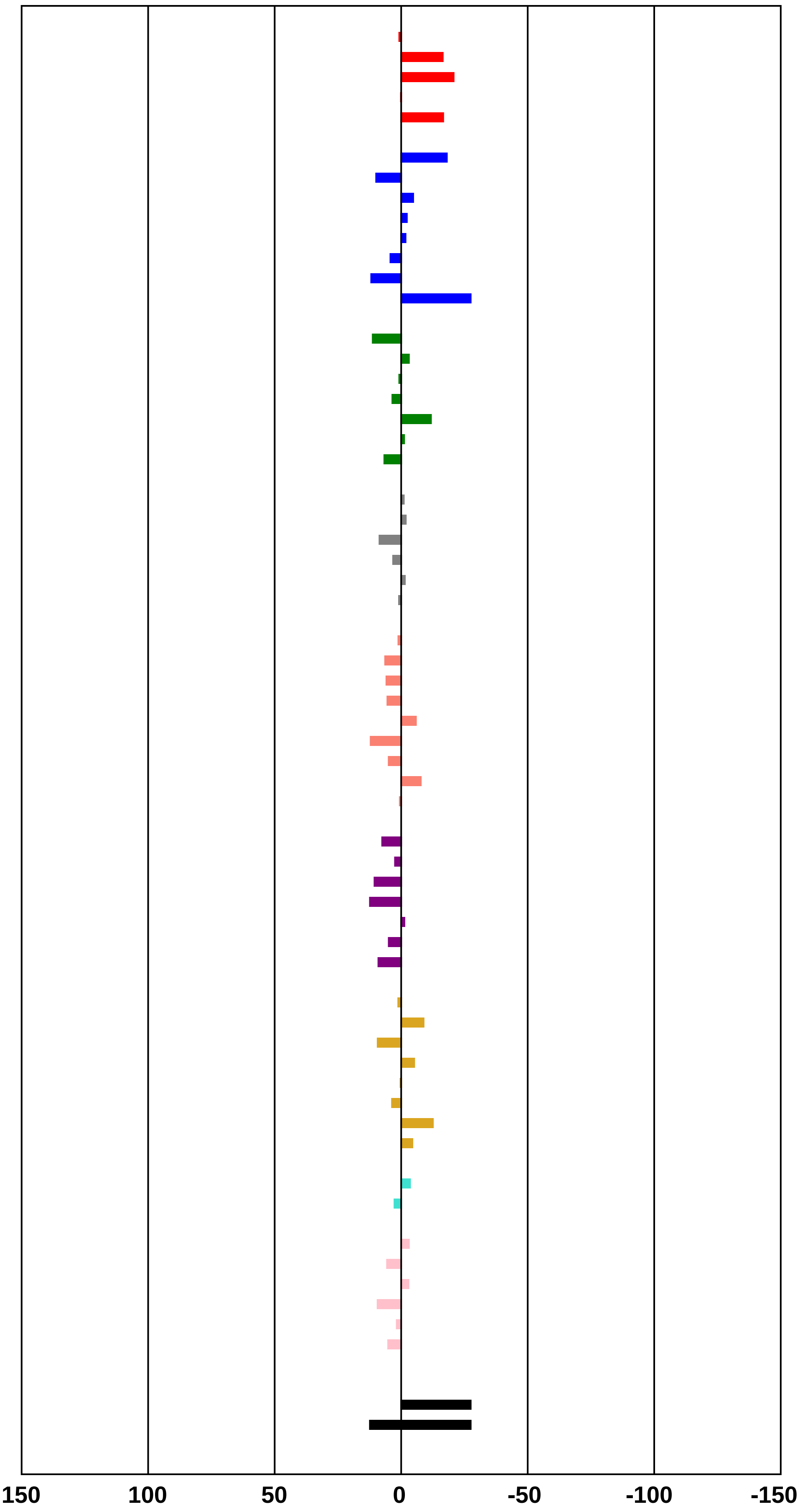

Developmental Therapeutics Program

One Dose Mean Graph

**NSC:** D-802585 / 1	**Conc:** 1.00E-5 Molar	**Test Date:** Dec 11, 2017
**Experiment ID:** 1712OS12	Product 4aF	**Report Date:** Jan 06, 2018

Panel/Cell Line	Growth Percent
Leukemia	
CCRF-CEM	97.27
HL-60(TB)	97.30
K-562	99.90
MOLT-4	97.28
RPMI-8226	101.90
Non-Small Cell Lung Cancer	
A549/ATCC	95.62
EKVX	106.31
HOP-62	94.33
HOP-92	86.03
NCI-H226	101.53
NCI-H23	108.19
NCI-H322M	88.19
NCI-H522	87.40
Colon Cancer	
HCC-2998	114.93
HCT-116	101.21
HCT-15	100.29
HT29	103.88
KM12	100.55
SW-620	105.03
CNS Cancer	
SF-268	101.17
SF-295	95.57
SF-539	107.51
SNB-19	90.14
SNB-75	114.38
U251	98.53
Melanoma	
LOX IMVI	100.31
MALME-3M	100.21
M14	99.91
MDA-MB-435	114.08
SK-MEL-2	118.58
SK-MEL-28	102.14
SK-MEL-5	110.95
UACC-257	97.39
UACC-62	91.16
Ovarian Cancer	
IGROV1	93.78
OVCAR-3	106.29
OVCAR-4	110.64
OVCAR-5	95.48
OVCAR-8	99.52
NCI/ADR-RES	103.64
SK-OV-3	99.01
Renal Cancer	
786-0	97.97
A498	105.77
ACHN	95.49
CAKI-1	97.50
RXF 393	117.51
SN12C	110.21
TK-10	105.25
UO-31	75.18
Prostate Cancer	
PC-3	90.27
DU-145	116.46
Breast Cancer	
MCF7	85.78
MDA-MB-231/ATCC	107.73
HS 578T	104.20
BT-549	103.55
MDA-MB-468	122.00
Mean	101.29
Delta	26.11
Range	46.82

Mean Growth Percent - Growth Percent

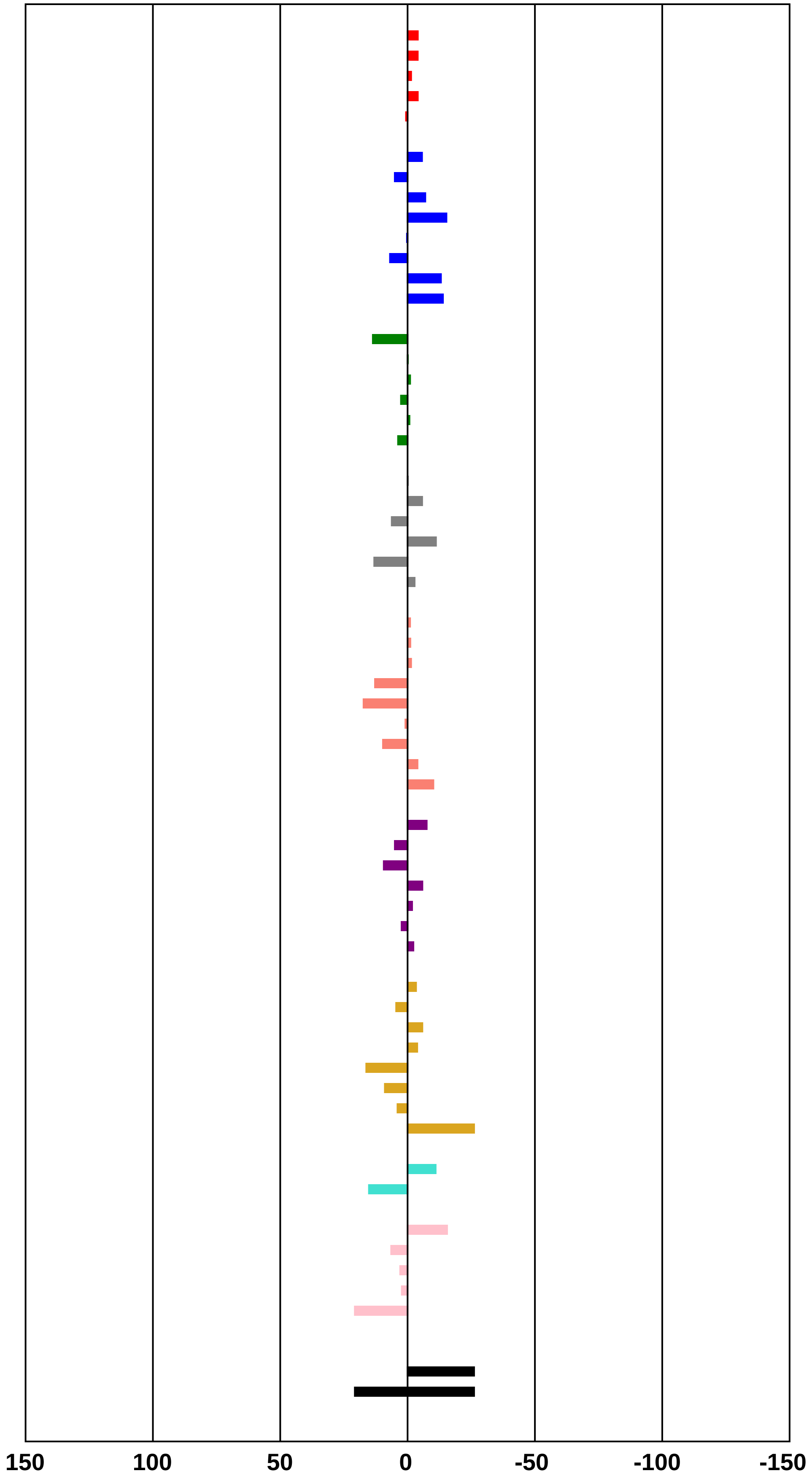

**Developmental Therapeutics Program**	**NSC:** D-800144 / 1	**Conc:** 1.00E-5 Molar	**Test Date:** Aug 21, 2017
**One Dose Mean Graph**	**Experiment ID:** 1708OS57	Product 4aG	**Report Date:** Sep 05, 2017

Panel/Cell Line	Growth Percent
Leukemia	
CCRF-CEM	101.17
K-562	81.36
MOLT-4	97.42
RPMI-8226	102.98
SR	83.51
Non-Small Cell Lung Cancer	
A549/ATCC	78.79
EKVX	106.79
HOP-62	104.24
HOP-92	102.49
NCI-H23	106.16
NCI-H322M	100.55
NCI-H460	110.17
NCI-H522	69.93
Colon Cancer	
COLO 205	111.29
HCC-2998	120.86
HCT-116	105.23
HCT-15	107.09
HT29	77.01
KM12	105.74
SW-620	105.78
CNS Cancer	
SF-268	102.73
SF-295	100.33
SF-539	103.40
SNB-19	107.13
SNB-75	98.49
U251	99.09
Melanoma	
LOX IMVI	99.95
MALME-3M	108.91
M14	104.36
MDA-MB-435	110.28
SK-MEL-2	96.50
SK-MEL-28	116.98
SK-MEL-5	103.84
UACC-257	74.73
UACC-62	103.22
Ovarian Cancer	
IGROV1	109.59
OVCAR-3	109.74
OVCAR-4	106.91
OVCAR-5	112.94
OVCAR-8	96.60
NCI/ADR-RES	105.58
SK-OV-3	107.78
Renal Cancer	
786-0	107.93
A498	84.76
ACHN	109.98
CAKI-1	94.09
RXF 393	114.62
SN12C	110.50
TK-10	90.98
UO-31	94.39
Prostate Cancer	
PC-3	102.91
DU-145	106.12
Breast Cancer	
MCF7	102.38
MDA-MB-231/ATCC	109.17
HS 578T	96.02
BT-549	110.45
T-47D	98.98
MDA-MB-468	101.62
Mean	101.60
Delta	31.67
Range	50.93

Mean Growth Percent - Growth Percent

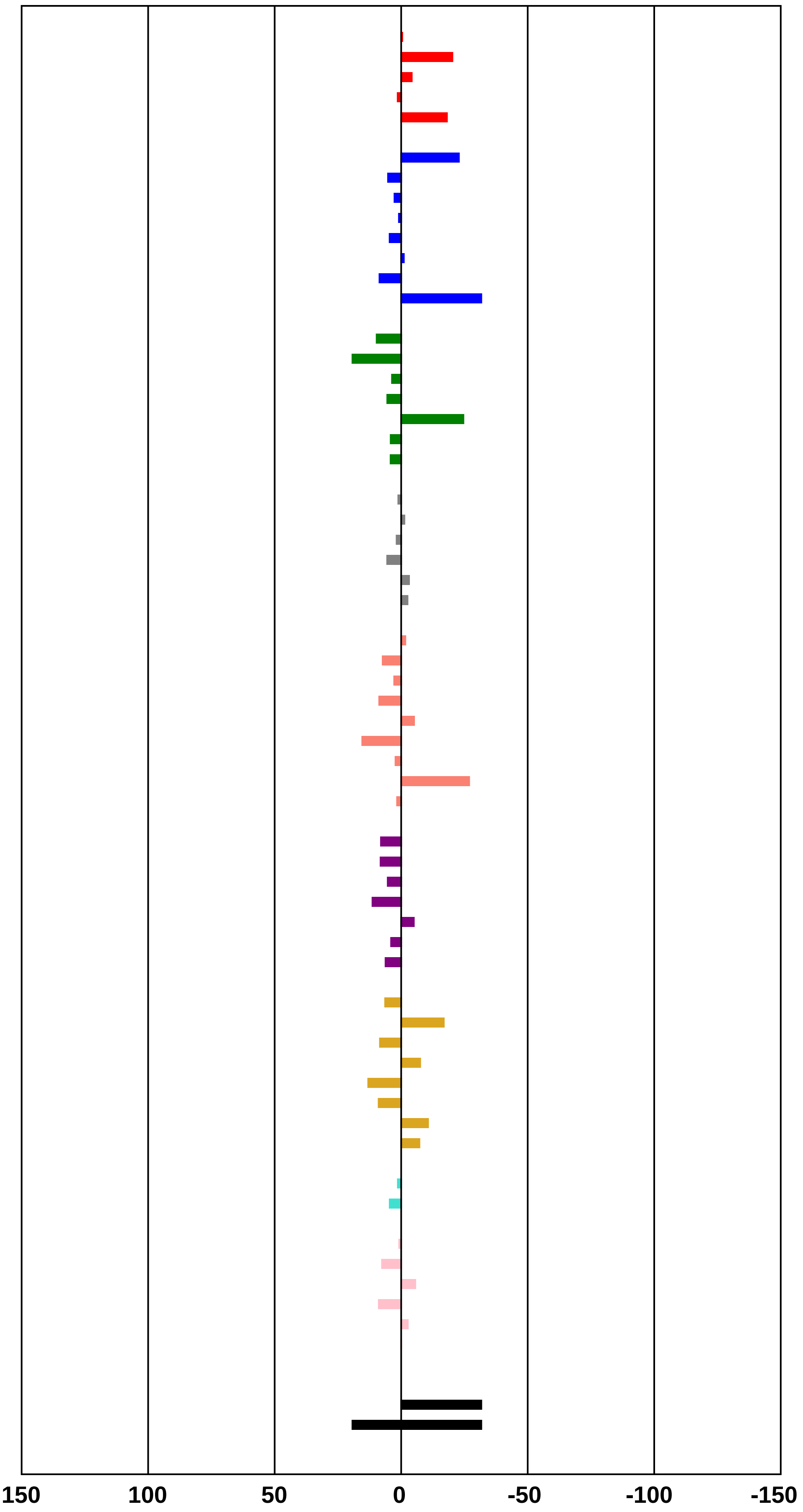

Developmental Therapeutics Program One Dose Mean Graph	NSC: D-802587 / 1	Conc: 1.00E-5 Molar	Test Date: Dec 11, 2017
	Experiment ID: 1712OS12	Product 4aH	Report Date: Jan 06, 2018

Panel/Cell Line	Growth Percent
**Leukemia**	
CCRF-CEM	92.66
HL-60(TB)	93.16
K-562	91.56
MOLT-4	79.54
RPMI-8226	80.68
**Non-Small Cell Lung Cancer**	
A549/ATCC	97.39
EKVX	100.24
HOP-62	97.52
HOP-92	75.56
NCI-H226	95.83
NCI-H23	99.59
NCI-H322M	91.63
NCI-H522	72.97
**Colon Cancer**	
HCC-2998	102.84
HCT-116	83.47
HCT-15	102.53
HT29	96.30
KM12	99.99
SW-620	105.59
**CNS Cancer**	
SF-268	98.11
SF-295	95.60
SF-539	106.56
SNB-19	97.06
SNB-75	89.63
U251	95.21
**Melanoma**	
LOX IMVI	100.51
MALME-3M	107.73
M14	99.48
MDA-MB-435	100.39
SK-MEL-2	99.61
SK-MEL-28	103.04
SK-MEL-5	109.91
UACC-257	96.52
UACC-62	85.33
**Ovarian Cancer**	
IGROV1	101.47
OVCAR-3	107.79
OVCAR-4	106.54
OVCAR-5	104.58
OVCAR-8	97.57
NCI/ADR-RES	92.00
SK-OV-3	103.37
**Renal Cancer**	
786-0	89.83
A498	101.60
ACHN	99.60
CAKI-1	101.46
RXF 393	105.25
SN12C	98.06
TK-10	103.48
UO-31	83.58
**Prostate Cancer**	
PC-3	81.35
DU-145	109.93
**Breast Cancer**	
MCF7	94.95
MDA-MB-231/ATCC	110.76
HS 578T	113.78
BT-549	97.76
MDA-MB-468	91.86
Mean	97.15
Delta	24.18
Range	40.81

Mean Growth Percent - Growth Percent

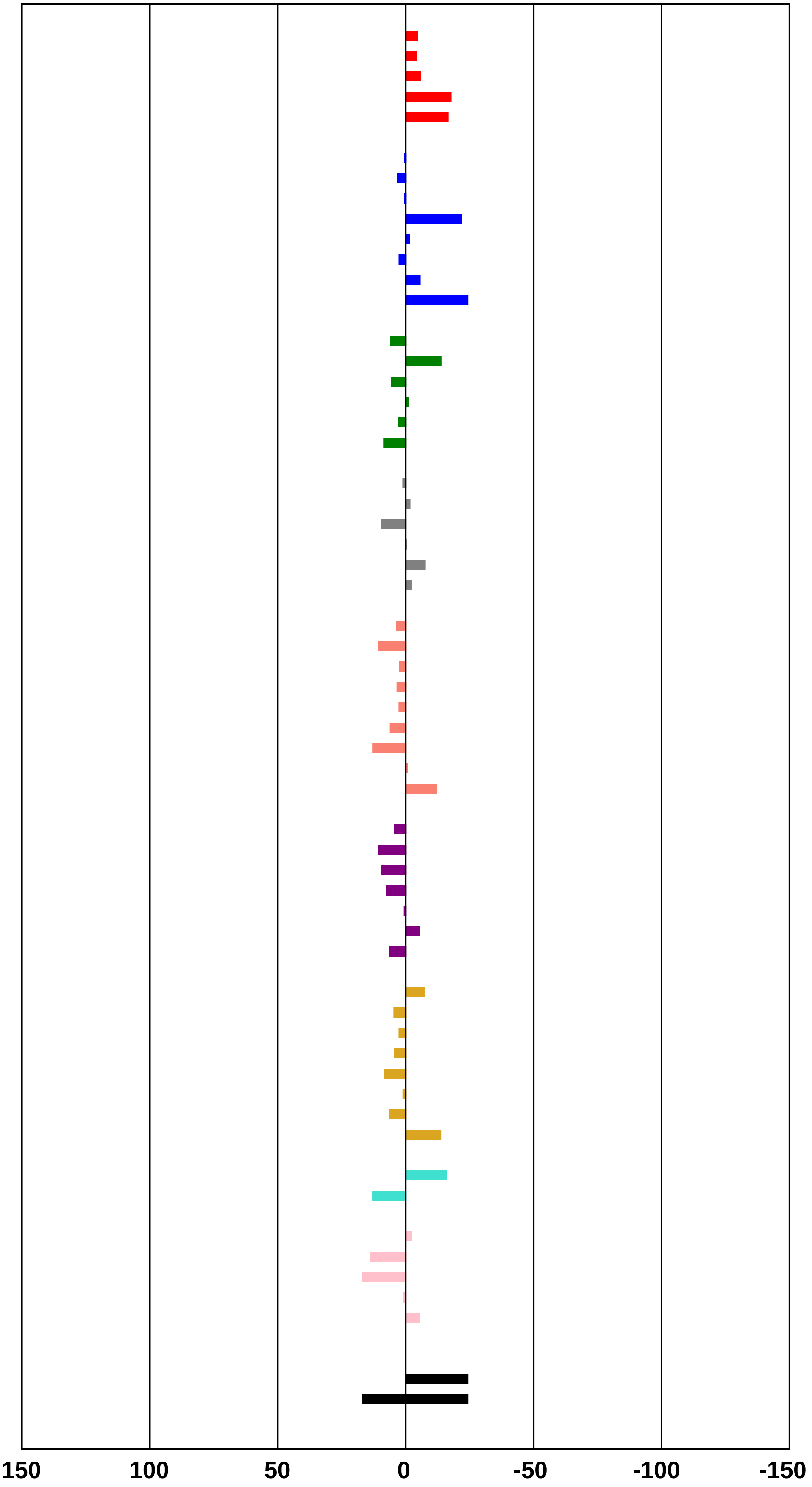

Developmental Therapeutics Program	NSC: D-800142 / 1	Conc: 1.00E-5 Molar	Test Date: Aug 21, 2017
One Dose Mean Graph	Experiment ID: 1708OS57	Product 4aI	Report Date: Sep 05, 2017

Panel/Cell Line	Growth Percent
Leukemia	
CCRF-CEM	94.08
K-562	84.27
MOLT-4	99.18
RPMI-8226	99.43
SR	74.16
Non-Small Cell Lung Cancer	
A549/ATCC	74.91
EKVX	114.59
HOP-62	98.51
HOP-92	93.36
NCI-H23	92.75
NCI-H322M	101.37
NCI-H460	111.19
NCI-H522	63.65
Colon Cancer	
COLO 205	111.84
HCC-2998	105.82
HCT-116	98.84
HCT-15	109.55
HT29	80.71
KM12	107.09
SW-620	102.54
CNS Cancer	
SF-268	104.77
SF-295	101.16
SF-539	102.96
SNB-19	104.62
SNB-75	95.63
U251	93.84
Melanoma	
LOX IMVI	99.42
MALME-3M	102.18
M14	107.33
MDA-MB-435	107.42
SK-MEL-2	80.08
SK-MEL-28	110.57
SK-MEL-5	102.31
UACC-257	75.20
UACC-62	98.01
Ovarian Cancer	
IGROV1	109.87
OVCAR-3	116.00
OVCAR-4	111.31
OVCAR-5	102.57
OVCAR-8	88.68
NCI/ADR-RES	99.35
SK-OV-3	104.05
Renal Cancer	
786-0	106.53
A498	89.23
ACHN	105.56
CAKI-1	95.39
RXF 393	110.33
SN12C	100.89
TK-10	87.34
UO-31	94.41
Prostate Cancer	
PC-3	88.12
DU-145	107.46
Breast Cancer	
MCF7	99.04
MDA-MB-231/ATCC	102.55
HS 578T	98.06
BT-549	108.27
T-47D	85.94
MDA-MB-468	102.27
Mean	98.56
Delta	34.91
Range	52.35

Mean Growth Percent - Growth Percent

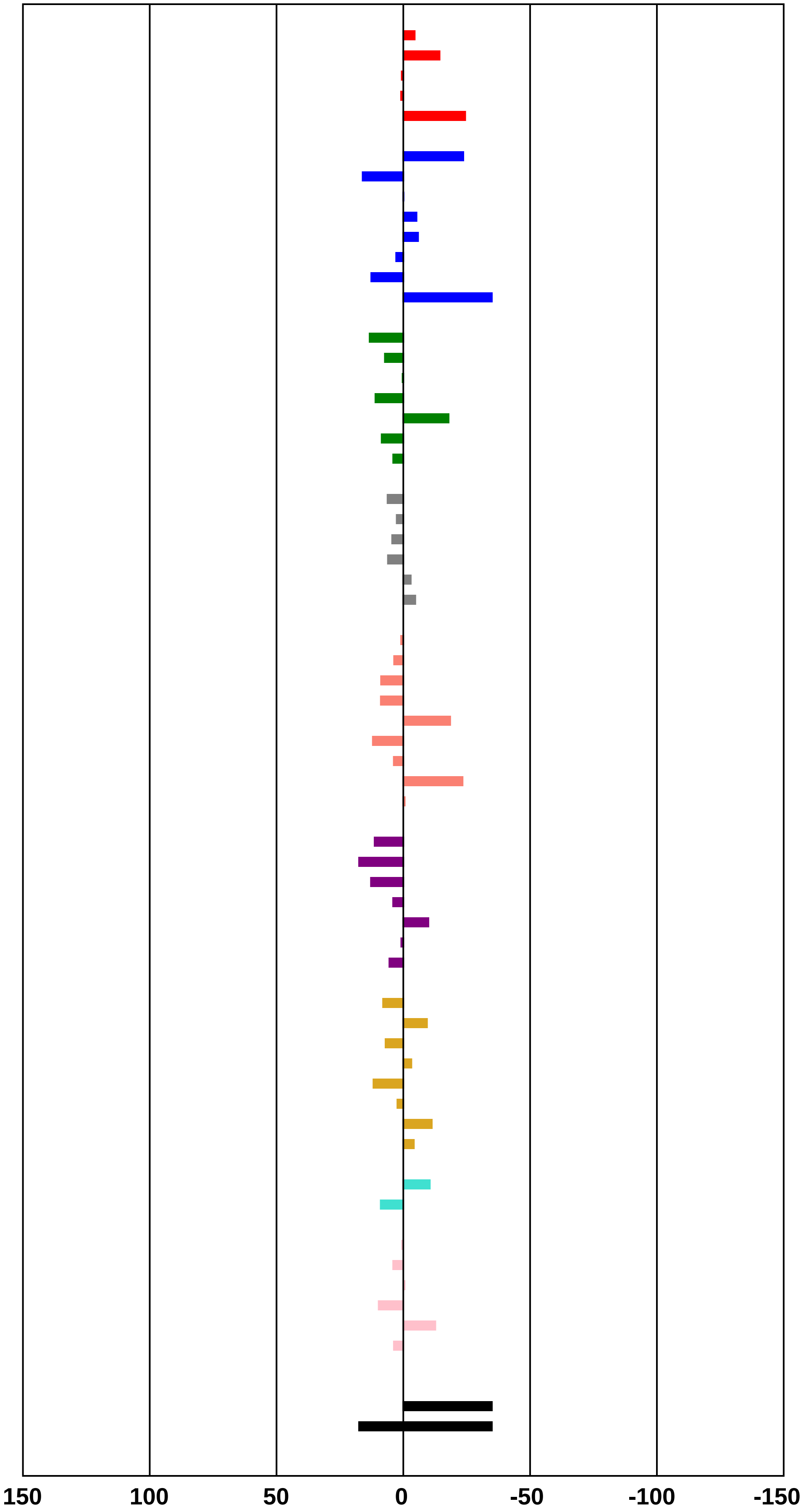

Developmental Therapeutics Program

One Dose Mean Graph

**NSC:** D-802589 / 1	**Conc:** 1.00E-5 Molar
**Test Date:** Dec 11, 2017	**Experiment ID:** 1712OS12
Product 4aJ	**Report Date:** Jan 06, 2018

Panel/Cell Line	Growth Percent
**Leukemia**	
CCRF-CEM	96.01
HL-60(TB)	96.47
K-562	90.55
MOLT-4	90.32
RPMI-8226	95.00
**Non-Small Cell Lung Cancer**	
A549/ATCC	98.15
EKVX	101.46
HOP-62	94.96
HOP-92	93.68
NCI-H226	97.62
NCI-H23	100.31
NCI-H322M	107.00
NCI-H522	83.00
**Colon Cancer**	
HCC-2998	113.62
HCT-116	92.64
HCT-15	107.89
HT29	103.83
KM12	103.41
SW-620	104.14
**CNS Cancer**	
SF-268	104.63
SF-295	107.19
SF-539	89.92
SNB-19	92.64
SNB-75	86.61
U251	104.21
**Melanoma**	
LOX IMVI	99.61
MALME-3M	105.79
M14	94.92
MDA-MB-435	106.49
SK-MEL-2	95.25
SK-MEL-28	109.82
SK-MEL-5	107.99
UACC-257	98.86
UACC-62	92.01
**Ovarian Cancer**	
IGROV1	102.86
OVCAR-3	105.82
OVCAR-4	104.76
OVCAR-5	94.62
OVCAR-8	103.46
NCI/ADR-RES	95.00
SK-OV-3	93.11
**Renal Cancer**	
786-0	90.66
A498	100.74
ACHN	100.59
CAKI-1	95.05
RXF 393	110.79
SN12C	99.01
TK-10	103.10
UO-31	84.08
**Prostate Cancer**	
PC-3	87.87
DU-145	113.37
**Breast Cancer**	
MCF7	104.04
MDA-MB-231/ATCC	84.71
HS 578T	101.31
BT-549	92.77
MDA-MB-468	91.25
Mean	98.66
Delta	15.66
Range	30.62

Mean Growth Percent - Growth Percent

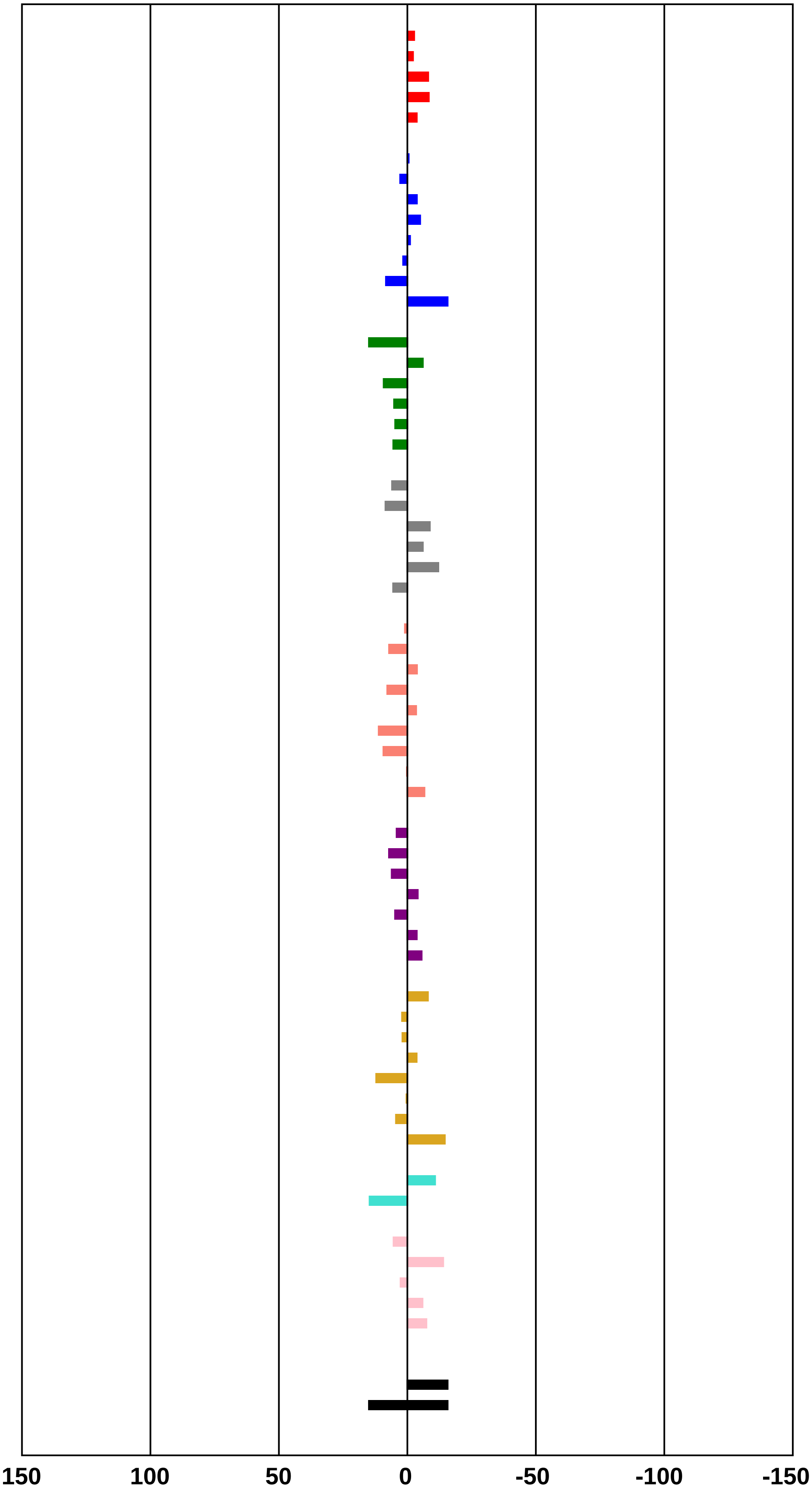

Developmental Therapeutics Program

One Dose Mean Graph

NSC: D-800134 / 1	Conc: 1.00E-5 Molar	Test Date: Aug 21, 2017
Experiment ID: 1708OS57	Product 4bA	Report Date: Sep 05, 2017

Panel/Cell Line	Growth Percent
**Leukemia**	
CCRF-CEM	95.36
K-562	91.39
MOLT-4	93.49
RPMI-8226	104.44
SR	78.43
**Non-Small Cell Lung Cancer**	
A549/ATCC	78.86
EKVX	104.65
HOP-62	100.09
HOP-92	83.94
NCI-H23	98.21
NCI-H322M	96.63
NCI-H460	111.12
NCI-H522	74.90
**Colon Cancer**	
COLO 205	108.00
HCC-2998	113.03
HCT-116	97.31
HCT-15	98.84
HT29	80.95
KM12	102.18
SW-620	108.38
**CNS Cancer**	
SF-268	109.78
SF-295	98.04
SF-539	95.44
SNB-19	100.20
SNB-75	88.09
U251	92.28
**Melanoma**	
LOX IMVI	101.57
MALME-3M	107.28
M14	101.98
MDA-MB-435	101.35
SK-MEL-2	92.83
SK-MEL-28	105.22
SK-MEL-5	102.19
UACC-257	79.51
UACC-62	97.03
**Ovarian Cancer**	
IGROV1	105.63
OVCAR-3	119.90
OVCAR-4	102.82
OVCAR-5	97.87
OVCAR-8	90.77
NCI/ADR-RES	98.79
SK-OV-3	102.08
**Renal Cancer**	
786-0	101.14
A498	91.48
ACHN	102.90
CAKI-1	89.87
RXF 393	108.00
SN12C	95.20
TK-10	85.29
UO-31	99.51
**Prostate Cancer**	
PC-3	90.34
DU-145	110.52
**Breast Cancer**	
MCF7	96.64
MDA-MB-231/ATCC	99.53
HS 578T	100.47
BT-549	106.90
T-47D	90.12
MDA-MB-468	99.04
Mean	97.89
Delta	22.99
Range	45.00

Mean Growth Percent - Growth Percent

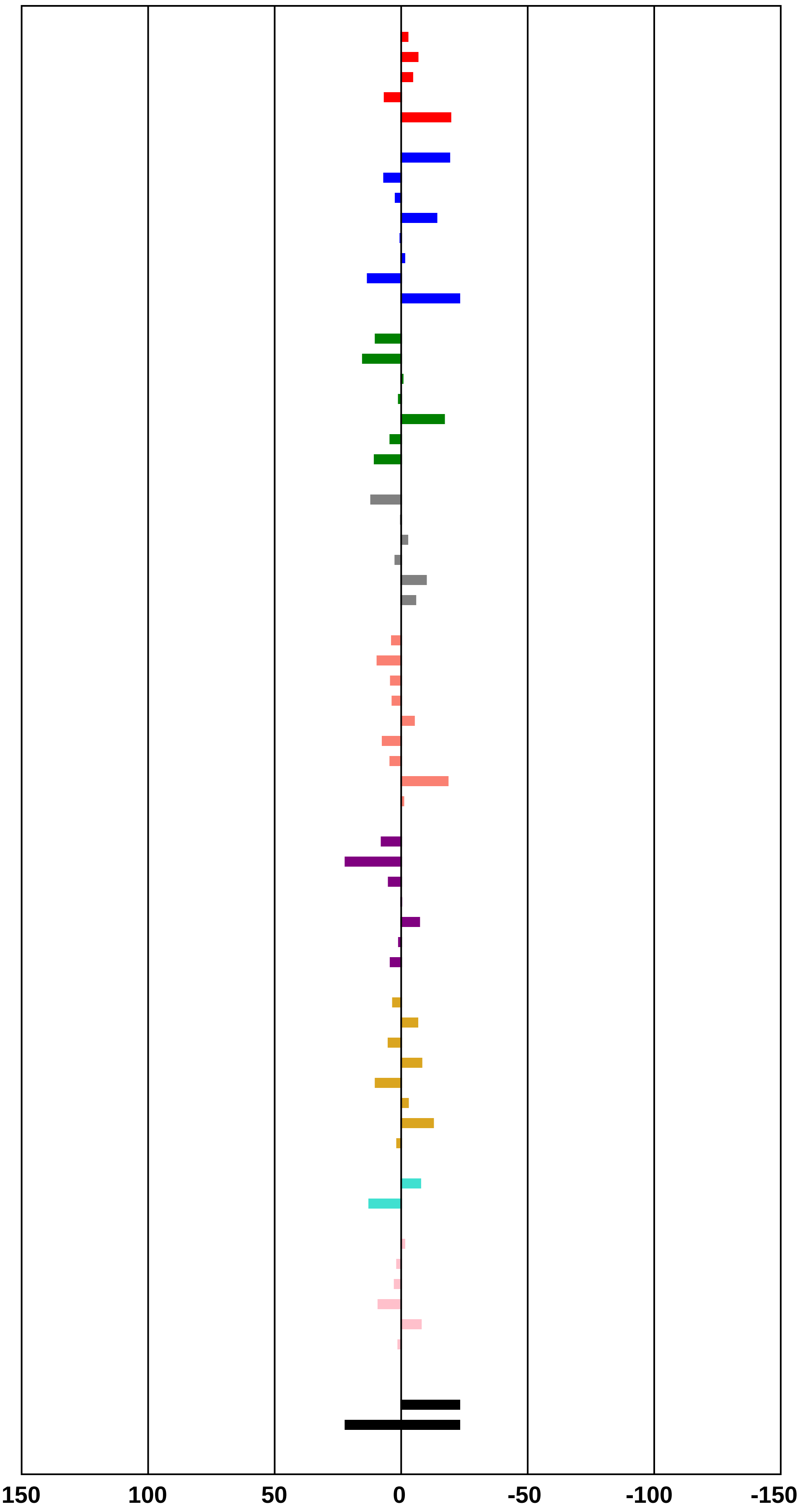

Developmental Therapeutics Program	NSC: D-800140 / 1	Conc: 1.00E-5 Molar	Test Date: Aug 21, 2017
One Dose Mean Graph	Experiment ID: 1708OS57	Product 4bB	Report Date: Sep 05, 2017

Panel/Cell Line	Growth Percent
**Leukemia**	
CCRF-CEM	88.50
K-562	80.28
MOLT-4	82.96
RPMI-8226	91.24
SR	80.60
**Non-Small Cell Lung Cancer**	
A549/ATCC	77.15
EKVX	106.14
HOP-62	99.61
HOP-92	88.23
NCI-H23	91.89
NCI-H322M	99.70
NCI-H460	103.30
NCI-H522	78.46
**Colon Cancer**	
COLO 205	111.70
HCC-2998	97.16
HCT-116	104.70
HCT-15	95.65
HT29	83.02
KM12	100.14
SW-620	103.50
**CNS Cancer**	
SF-268	96.91
SF-295	92.26
SF-539	90.78
SNB-19	100.80
SNB-75	92.47
U251	91.25
**Melanoma**	
LOX IMVI	97.04
MALME-3M	97.54
M14	107.44
MDA-MB-435	96.92
SK-MEL-2	85.75
SK-MEL-28	104.28
SK-MEL-5	90.98
UACC-257	75.54
UACC-62	82.12
**Ovarian Cancer**	
IGROV1	97.21
OVCAR-3	111.49
OVCAR-4	80.04
OVCAR-5	93.99
OVCAR-8	92.33
NCI/ADR-RES	94.45
SK-OV-3	88.35
**Renal Cancer**	
786-0	101.65
A498	75.85
ACHN	106.88
CAKI-1	79.18
RXF 393	96.88
SN12C	89.32
TK-10	99.69
UO-31	86.71
**Prostate Cancer**	
PC-3	78.76
DU-145	101.37
**Breast Cancer**	
MCF7	87.26
MDA-MB-231/ATCC	97.39
HS 578T	91.10
BT-549	107.51
T-47D	83.93
MDA-MB-468	88.64
Mean	93.03
Delta	17.49
Range	36.16

Mean Growth Percent - Growth Percent

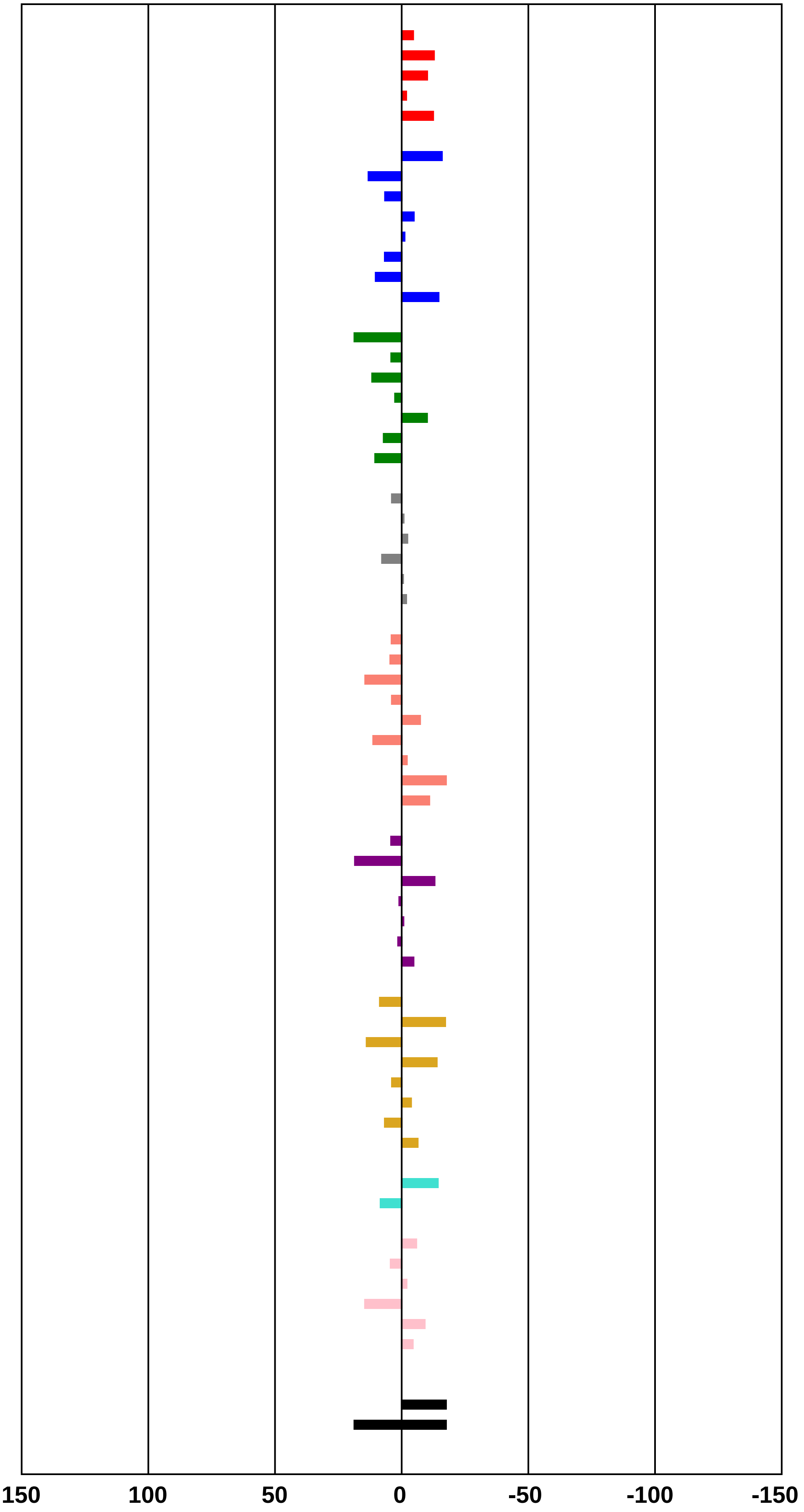

Developmental Therapeutics Program	NSC: D-800137 / 1	Conc: 1.00E-5 Molar	Test Date: Aug 21, 2017
One Dose Mean Graph	Experiment ID: 1708OS57	Product 4bC	Report Date: Sep 05, 2017

Panel/Cell Line	Growth Percent
**Leukemia**	
CCRF-CEM	88.01
K-562	87.85
MOLT-4	74.60
RPMI-8226	87.02
SR	72.94
**Non-Small Cell Lung Cancer**	
A549/ATCC	69.56
EKVX	104.01
HOP-62	97.48
HOP-92	87.86
NCI-H23	99.44
NCI-H322M	101.48
NCI-H460	105.02
NCI-H522	80.71
**Colon Cancer**	
COLO 205	104.03
HCC-2998	105.71
HCT-116	89.85
HCT-15	100.40
HT29	79.15
KM12	100.49
SW-620	104.67
**CNS Cancer**	
SF-268	103.76
SF-295	93.93
SF-539	100.26
SNB-19	103.86
SNB-75	96.49
U251	91.69
**Melanoma**	
LOX IMVI	102.39
MALME-3M	112.02
M14	100.54
MDA-MB-435	98.98
SK-MEL-2	87.91
SK-MEL-28	111.34
SK-MEL-5	100.19
UACC-257	73.01
UACC-62	87.36
**Ovarian Cancer**	
IGROV1	103.13
OVCAR-3	115.46
OVCAR-4	93.35
OVCAR-5	102.51
OVCAR-8	92.54
NCI/ADR-RES	101.64
SK-OV-3	100.95
**Renal Cancer**	
786-0	101.91
A498	79.95
ACHN	106.49
CAKI-1	84.55
RXF 393	101.10
SN12C	94.74
TK-10	92.04
UO-31	91.39
**Prostate Cancer**	
PC-3	80.00
DU-145	102.05
**Breast Cancer**	
MCF7	90.83
MDA-MB-231/ATCC	100.45
HS 578T	94.37
BT-549	104.97
T-47D	86.40
MDA-MB-468	101.77
Mean	95.29
Delta	25.73
Range	45.90

Mean Growth Percent - Growth Percent

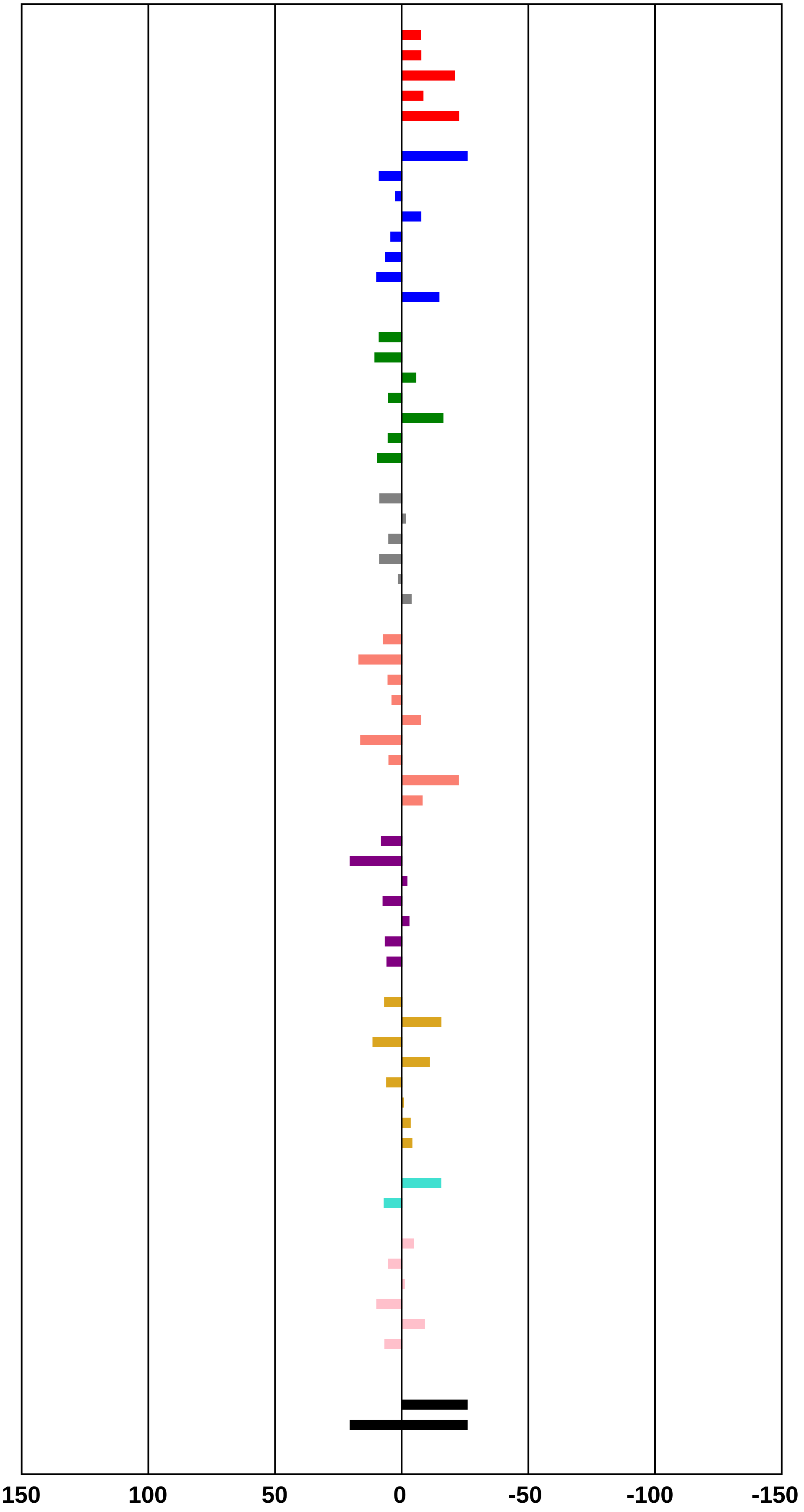

Developmental Therapeutics Program	NSC: D-800139 / 1	Conc: 1.00E-5 Molar	Test Date: Aug 21, 2017
One Dose Mean Graph	Experiment ID: 1708OS57	Product 4bD	Report Date: Sep 05, 2017

Panel/Cell Line	Growth Percent
**Leukemia**	
CCRF-CEM	89.45
K-562	81.47
MOLT-4	75.84
RPMI-8226	85.05
SR	75.10
**Non-Small Cell Lung Cancer**	
A549/ATCC	77.57
EKVX	98.64
HOP-62	90.69
HOP-92	88.94
NCI-H23	99.06
NCI-H322M	100.14
NCI-H460	109.48
NCI-H522	68.93
**Colon Cancer**	
COLO 205	104.27
HCC-2998	110.65
HCT-116	87.94
HCT-15	95.74
HT29	78.21
KM12	99.50
SW-620	108.66
**CNS Cancer**	
SF-268	102.43
SF-295	92.24
SF-539	99.59
SNB-19	99.21
SNB-75	93.18
U251	94.94
**Melanoma**	
LOX IMVI	100.31
MALME-3M	99.33
M14	103.59
MDA-MB-435	102.18
SK-MEL-2	85.25
SK-MEL-28	108.59
SK-MEL-5	101.35
UACC-257	74.68
UACC-62	87.82
**Ovarian Cancer**	
IGROV1	99.72
OVCAR-3	109.46
OVCAR-4	89.60
OVCAR-5	104.21
OVCAR-8	92.35
NCI/ADR-RES	100.57
SK-OV-3	97.14
**Renal Cancer**	
786-0	100.40
A498	85.48
ACHN	95.88
CAKI-1	80.18
RXF 393	105.49
SN12C	93.35
TK-10	101.65
UO-31	81.22
**Prostate Cancer**	
PC-3	92.84
DU-145	108.47
**Breast Cancer**	
MCF7	85.93
MDA-MB-231/ATCC	97.63
HS 578T	96.99
BT-549	100.02
T-47D	89.18
MDA-MB-468	97.93
Mean	94.41
Delta	25.48
Range	41.72

Mean Growth Percent - Growth Percent

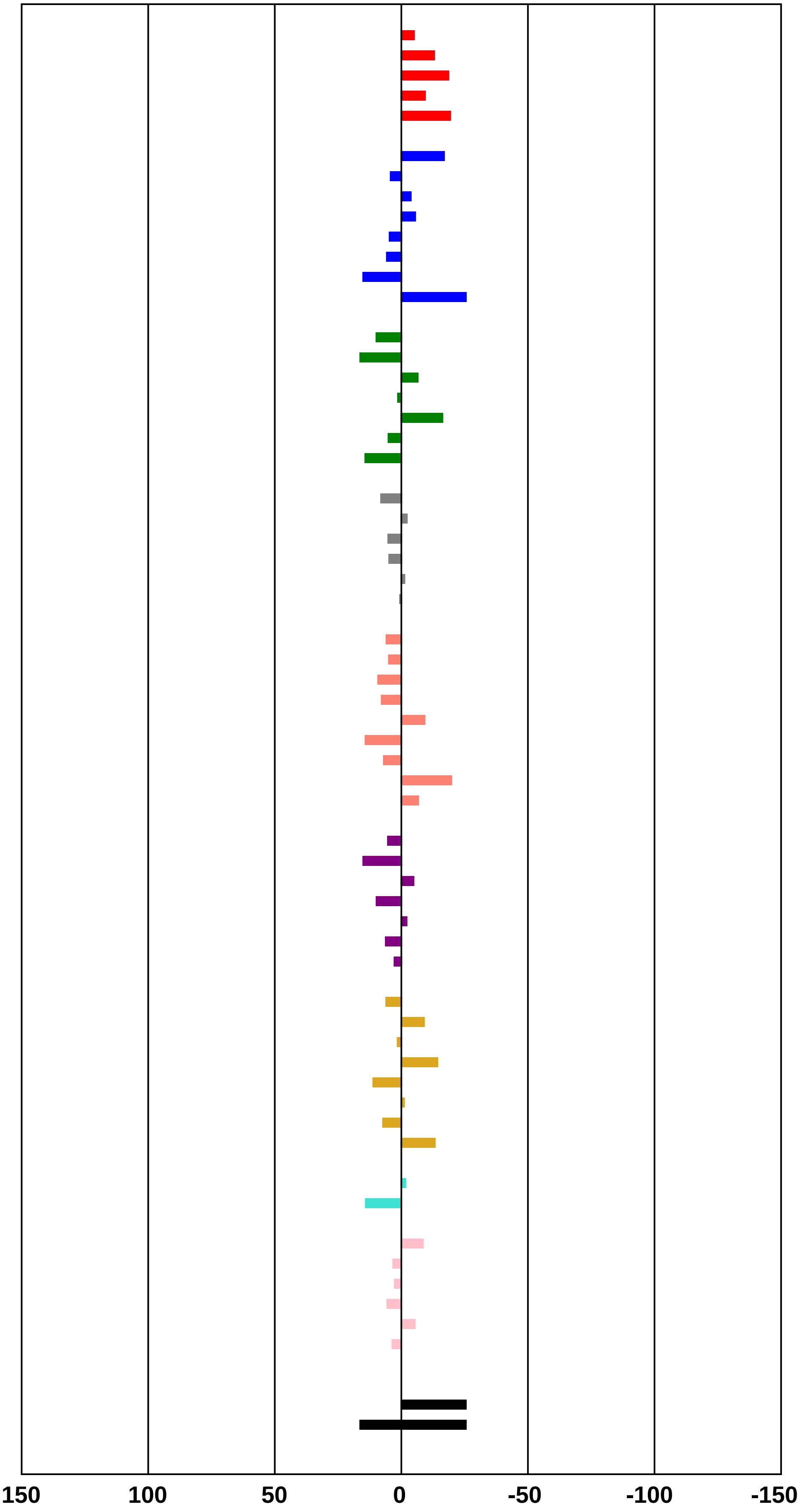

Developmental Therapeutics Program	NSC: D-800138 / 1	Conc: 1.00E-5 Molar	Test Date: Aug 21, 2017
One Dose Mean Graph	Experiment ID: 1708OS57	Product 4bE	Report Date: Sep 05, 2017

Panel/Cell Line	Growth Percent
Leukemia	
CCRF-CEM	94.80
K-562	82.59
MOLT-4	85.50
RPMI-8226	95.74
SR	81.22
Non-Small Cell Lung Cancer	
A549/ATCC	76.25
EKVX	112.43
HOP-62	98.17
HOP-92	101.27
NCI-H23	98.91
NCI-H322M	102.58
NCI-H460	104.83
NCI-H522	67.45
Colon Cancer	
COLO 205	115.50
HCC-2998	107.01
HCT-116	104.89
HCT-15	102.87
HT29	89.19
KM12	107.56
SW-620	104.85
CNS Cancer	
SF-268	106.18
SF-295	97.61
SF-539	104.59
SNB-19	102.99
SNB-75	98.09
U251	100.00
Melanoma	
LOX IMVI	101.37
MALME-3M	110.68
M14	104.58
MDA-MB-435	98.34
SK-MEL-2	97.24
SK-MEL-28	112.92
SK-MEL-5	101.12
UACC-257	81.73
UACC-62	91.91
Ovarian Cancer	
IGROV1	107.98
OVCAR-3	115.54
OVCAR-4	94.52
OVCAR-5	110.33
OVCAR-8	90.08
NCI/ADR-RES	98.88
SK-OV-3	101.46
Renal Cancer	
786-0	105.14
A498	85.49
ACHN	106.16
CAKI-1	85.65
RXF 393	107.08
SN12C	101.62
TK-10	95.45
UO-31	91.68
Prostate Cancer	
PC-3	96.89
DU-145	106.55
Breast Cancer	
MCF7	89.13
MDA-MB-231/ATCC	105.57
HS 578T	95.18
BT-549	110.64
T-47D	96.67
MDA-MB-468	93.38
Mean	98.86
Delta	31.41
Range	48.09

Mean Growth Percent - Growth Percent

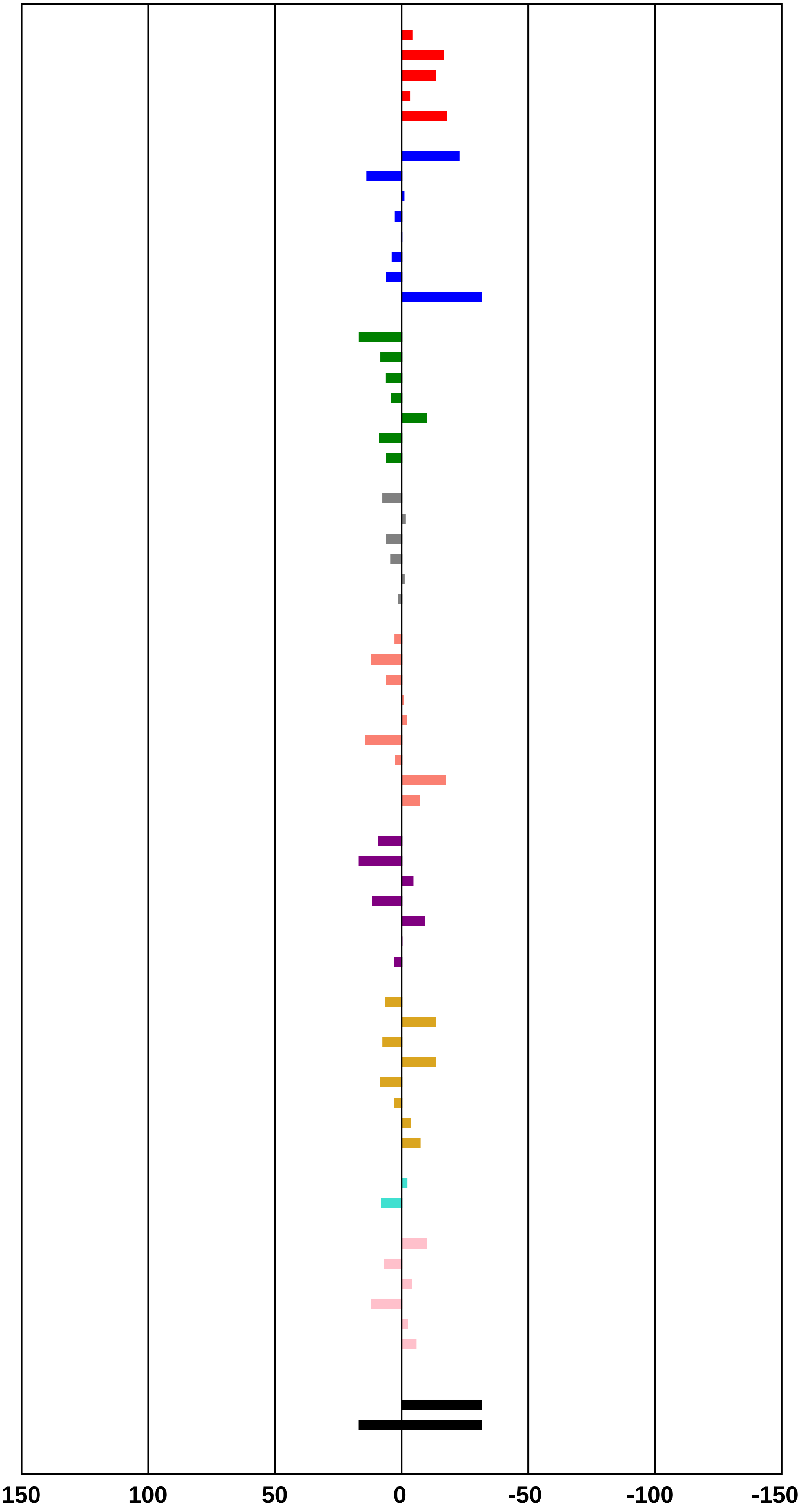

Developmental Therapeutics Program

One Dose Mean Graph

NSC: D-800135 / 1	Conc: 1.00E-5 Molar	Test Date: Aug 21, 2017
Experiment ID: 1708OS57	Product 4bF	Report Date: Sep 05, 2017

Panel/Cell Line	Growth Percent
**Leukemia**	
CCRF-CEM	93.94
K-562	77.39
MOLT-4	76.38
RPMI-8226	88.87
SR	84.33
**Non-Small Cell Lung Cancer**	
A549/ATCC	80.46
EKVX	106.83
HOP-62	95.58
HOP-92	80.27
NCI-H23	96.08
NCI-H322M	107.95
NCI-H460	110.45
NCI-H522	72.32
**Colon Cancer**	
COLO 205	108.39
HCC-2998	101.61
HCT-116	93.74
HCT-15	101.20
HT29	84.10
KM12	96.53
SW-620	106.66
**CNS Cancer**	
SF-268	98.37
SF-295	93.44
SF-539	105.73
SNB-19	99.83
SNB-75	94.53
U251	93.45
**Melanoma**	
LOX IMVI	102.28
MALME-3M	111.34
M14	102.83
MDA-MB-435	102.01
SK-MEL-2	93.38
SK-MEL-28	106.19
SK-MEL-5	103.30
UACC-257	87.83
UACC-62	93.24
**Ovarian Cancer**	
IGROV1	107.92
OVCAR-3	108.61
OVCAR-4	98.29
OVCAR-5	107.98
OVCAR-8	94.06
NCI/ADR-RES	97.36
SK-OV-3	106.02
**Renal Cancer**	
786-0	98.12
A498	96.46
ACHN	101.13
CAKI-1	85.69
RXF 393	95.28
SN12C	101.88
TK-10	99.99
UO-31	89.30
**Prostate Cancer**	
PC-3	93.51
DU-145	105.22
**Breast Cancer**	
MCF7	90.37
MDA-MB-231/ATCC	93.86
HS 578T	96.39
BT-549	106.11
T-47D	97.83
MDA-MB-468	86.72
Mean	96.71
Delta	24.39
Range	39.02

Mean Growth Percent - Growth Percent

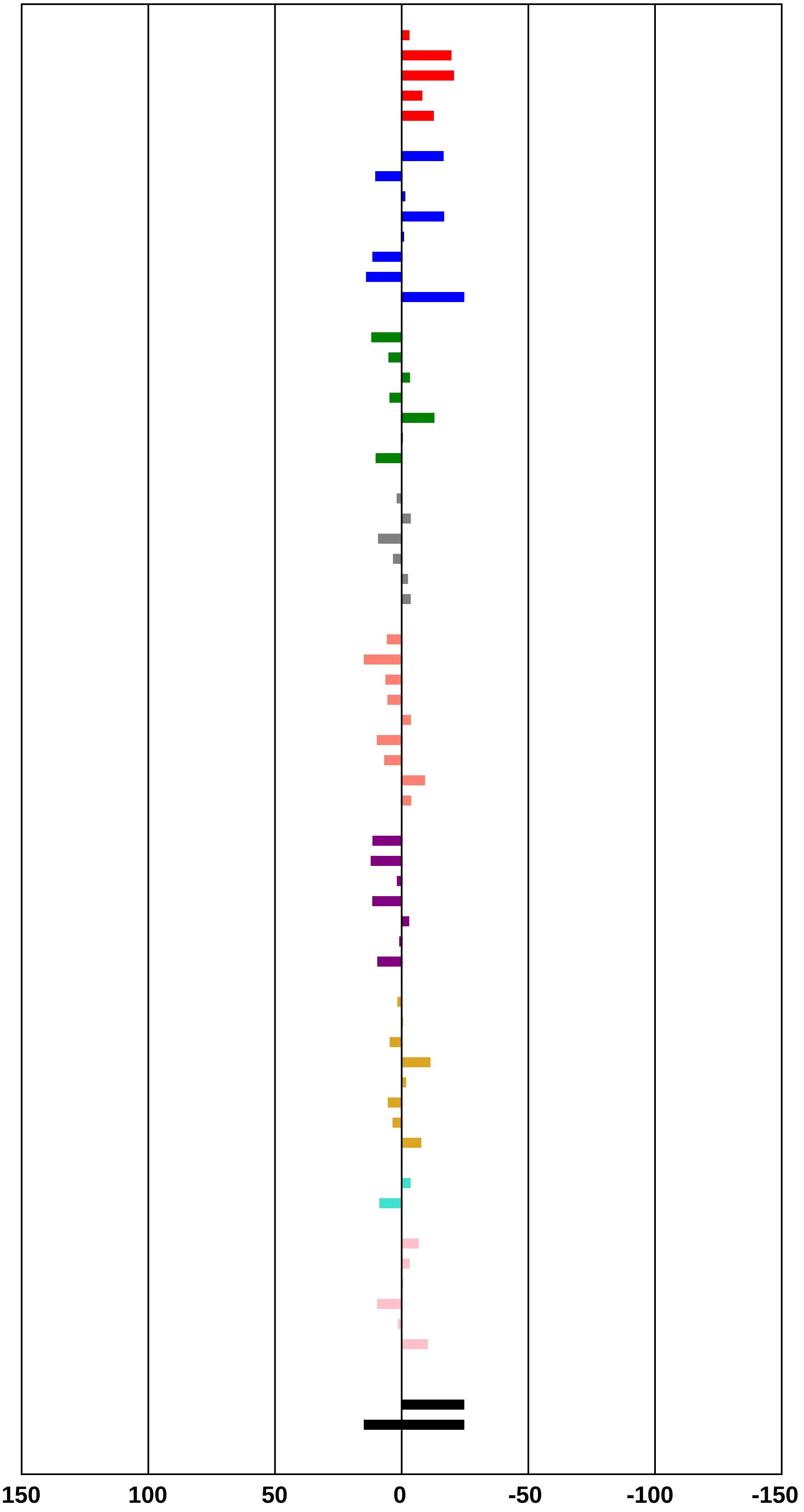

Developmental Therapeutics Program

One Dose Mean Graph

NSC: D-800136 / 1 | **Conc:** 1.00E-5 Molar | **Test Date:** Aug 21, 2017

Experiment ID: 1708OS57 | Product 4bG | **Report Date:** Sep 05, 2017

Panel/Cell Line	Growth Percent
Leukemia	
CCRF-CEM	98.16
K-562	85.09
MOLT-4	96.78
RPMI-8226	96.98
SR	82.72
Non-Small Cell Lung Cancer	
A549/ATCC	78.33
EKVX	102.57
HOP-62	98.30
HOP-92	98.11
NCI-H23	103.28
NCI-H322M	100.27
NCI-H460	107.31
NCI-H522	70.03
Colon Cancer	
COLO 205	105.75
HCC-2998	108.79
HCT-116	99.72
HCT-15	98.16
HT29	79.32
KM12	103.74
SW-620	104.41
CNS Cancer	
SF-268	105.82
SF-295	99.22
SF-539	109.96
SNB-19	102.87
SNB-75	96.63
U251	94.53
Melanoma	
LOX IMVI	98.70
MALME-3M	100.10
M14	100.11
MDA-MB-435	103.94
SK-MEL-2	98.59
SK-MEL-28	109.27
SK-MEL-5	98.16
UACC-257	74.15
UACC-62	95.39
Ovarian Cancer	
IGROV1	107.94
OVCAR-3	110.44
OVCAR-4	95.75
OVCAR-5	107.74
OVCAR-8	95.21
NCI/ADR-RES	102.18
SK-OV-3	99.44
Renal Cancer	
786-0	104.93
A498	93.00
ACHN	109.61
CAKI-1	90.92
RXF 393	105.28
SN12C	107.44
TK-10	91.89
UO-31	94.50
Prostate Cancer	
PC-3	98.65
DU-145	107.06
Breast Cancer	
MCF7	92.90
MDA-MB-231/ATCC	98.66
HS 578T	95.65
BT-549	109.63
T-47D	98.35
MDA-MB-468	93.22
Mean	98.55
Delta	28.52
Range	40.41

Mean Growth Percent - Growth Percent

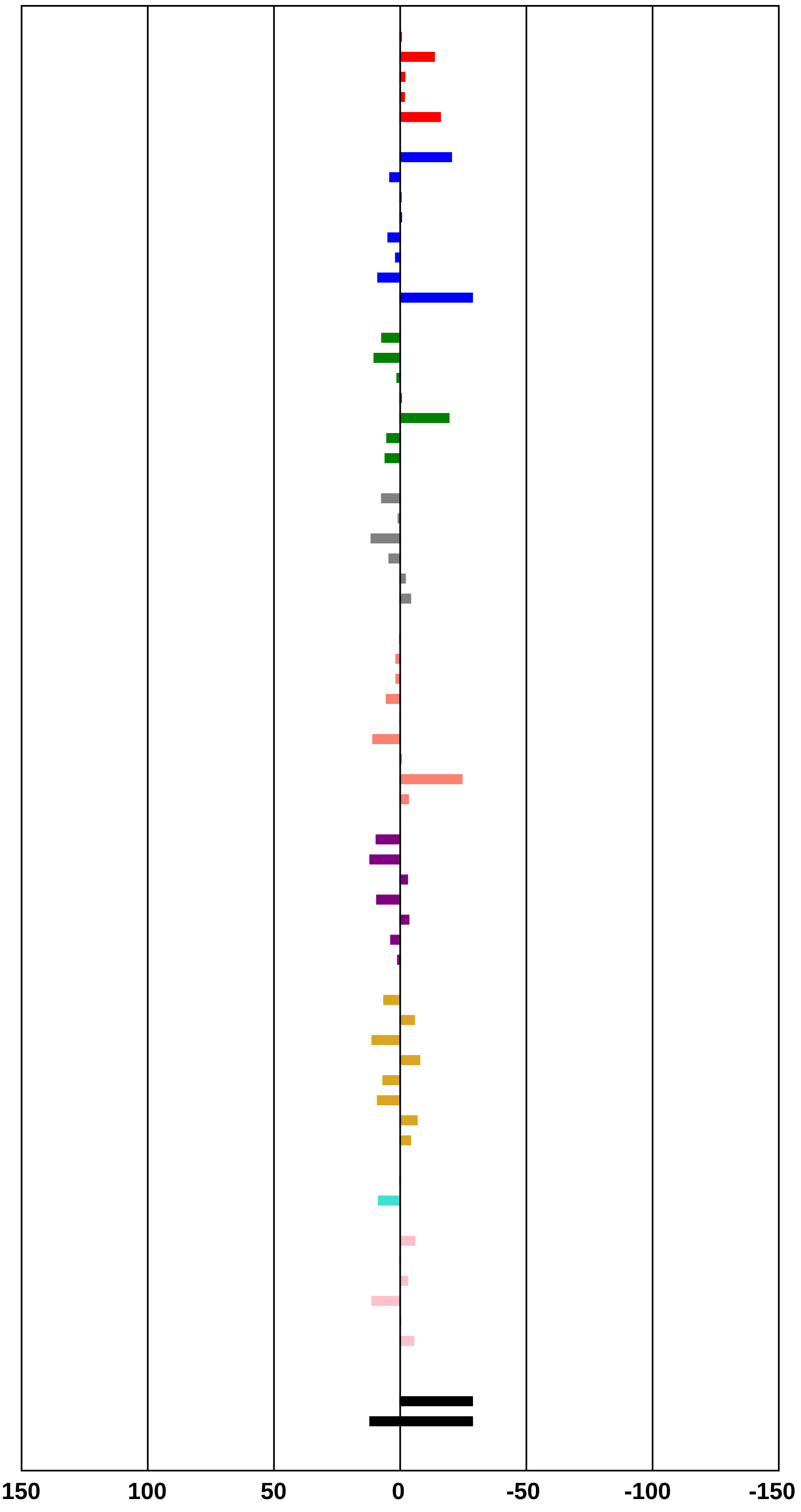

Developmental Therapeutics Program	NSC: D-800132 / 1	Conc: 1.00E-5 Molar	Test Date: Aug 21, 2017
One Dose Mean Graph	Experiment ID: 1708OS57	Product 4bH	Report Date: Sep 05, 2017

Panel/Cell Line	Growth Percent
Leukemia	
CCRF-CEM	84.65
K-562	79.34
MOLT-4	83.18
RPMI-8226	86.31
SR	88.00
Non-Small Cell Lung Cancer	
A549/ATCC	77.42
EKVX	112.50
HOP-62	103.25
HOP-92	106.23
NCI-H23	94.51
NCI-H322M	92.74
NCI-H460	104.39
NCI-H522	71.73
Colon Cancer	
COLO 205	109.32
HCC-2998	99.28
HCT-116	96.09
HCT-15	105.31
HT29	83.74
KM12	101.11
SW-620	104.06
CNS Cancer	
SF-268	99.59
SF-295	98.60
SF-539	100.42
SNB-19	100.91
SNB-75	99.75
U251	90.46
Melanoma	
LOX IMVI	101.94
MALME-3M	108.23
M14	108.31
MDA-MB-435	102.36
SK-MEL-2	92.79
SK-MEL-28	108.92
SK-MEL-5	92.84
UACC-257	79.49
UACC-62	81.85
Ovarian Cancer	
IGROV1	100.21
OVCAR-3	101.07
OVCAR-4	93.22
OVCAR-5	99.53
OVCAR-8	93.06
NCI/ADR-RES	96.56
SK-OV-3	99.25
Renal Cancer	
786-0	103.58
A498	77.10
ACHN	106.47
CAKI-1	86.12
RXF 393	102.82
SN12C	97.63
TK-10	105.53
UO-31	86.15
Prostate Cancer	
PC-3	78.16
DU-145	109.42
Breast Cancer	
MCF7	89.35
MDA-MB-231/ATCC	99.81
HS 578T	91.90
BT-549	107.71
T-47D	79.65
MDA-MB-468	88.80
Mean	95.56
Delta	23.83
Range	40.77

Mean Growth Percent - Growth Percent

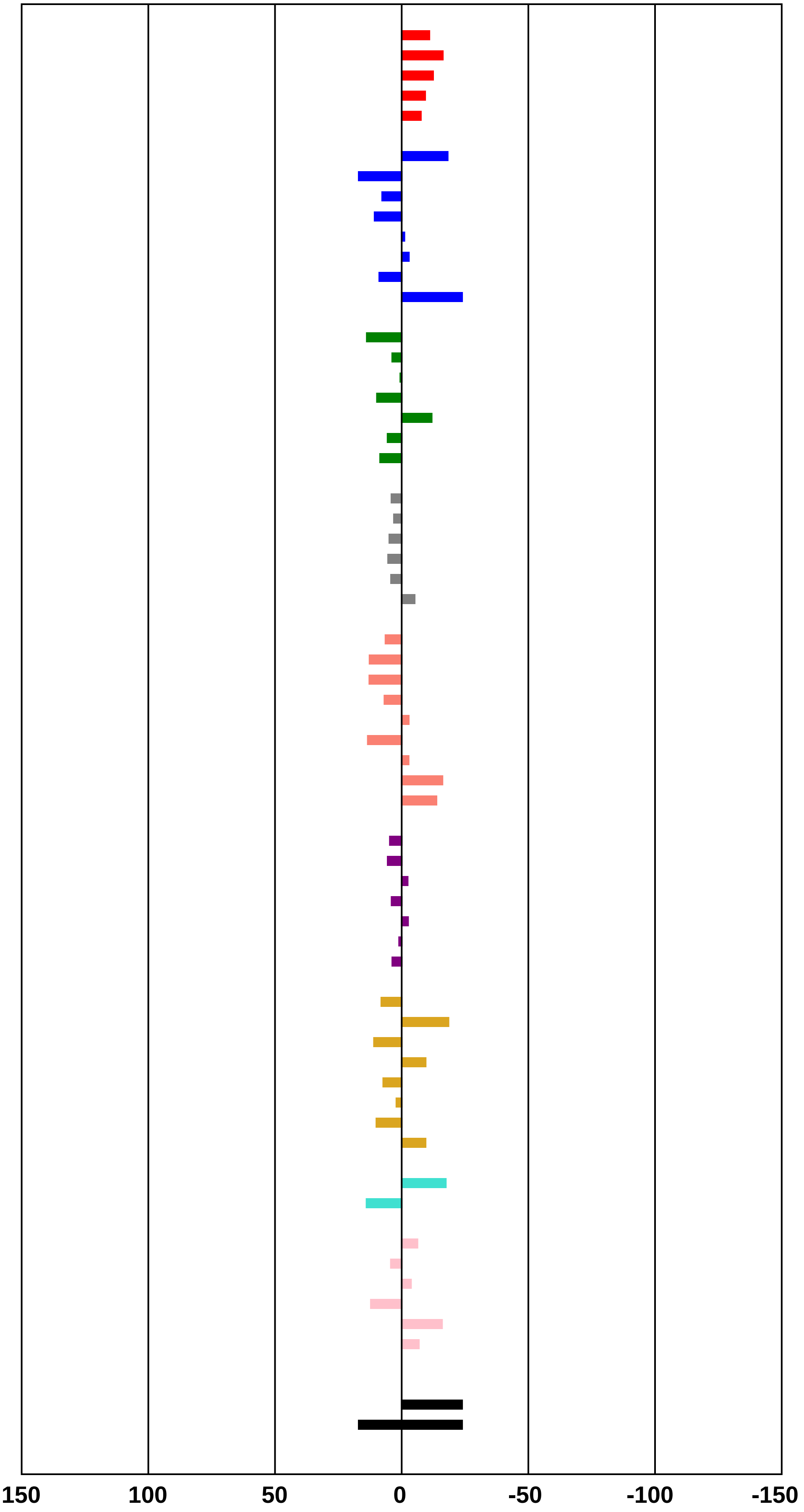

Developmental Therapeutics Program

One Dose Mean Graph

NSC: D-800141 / 1	Conc: 1.00E-5 Molar	Test Date: Aug 21, 2017
Experiment ID: 1708OS57	Product 4bI	Report Date: Sep 05, 2017

Panel/Cell Line	Growth Percent
**Leukemia**	
CCRF-CEM	81.76
K-562	66.10
MOLT-4	76.47
RPMI-8226	81.37
SR	75.23
**Non-Small Cell Lung Cancer**	
A549/ATCC	79.51
EKVX	107.62
HOP-62	97.01
HOP-92	93.76
NCI-H23	82.91
NCI-H322M	96.18
NCI-H460	103.23
NCI-H522	54.70
**Colon Cancer**	
COLO 205	107.68
HCC-2998	96.29
HCT-116	95.39
HCT-15	112.46
HT29	77.04
KM12	102.81
SW-620	101.58
**CNS Cancer**	
SF-268	99.31
SF-295	102.29
SF-539	99.30
SNB-19	96.85
SNB-75	110.74
U251	93.58
**Melanoma**	
LOX IMVI	100.38
MALME-3M	106.59
M14	107.68
MDA-MB-435	105.24
SK-MEL-2	79.40
SK-MEL-28	104.89
SK-MEL-5	88.12
UACC-257	73.35
UACC-62	79.36
**Ovarian Cancer**	
IGROV1	104.59
OVCAR-3	110.84
OVCAR-4	91.78
OVCAR-5	111.28
OVCAR-8	91.42
NCI/ADR-RES	100.18
SK-OV-3	103.22
**Renal Cancer**	
786-0	107.06
A498	83.43
ACHN	96.90
CAKI-1	89.36
RXF 393	104.59
SN12C	96.04
TK-10	95.68
UO-31	79.77
**Prostate Cancer**	
PC-3	88.41
DU-145	103.50
**Breast Cancer**	
MCF7	87.17
MDA-MB-231/ATCC	98.00
HS 578T	94.35
BT-549	110.79
T-47D	71.47
MDA-MB-468	70.36
Mean	93.56
Delta	38.86
Range	57.76

Mean Growth Percent - Growth Percent

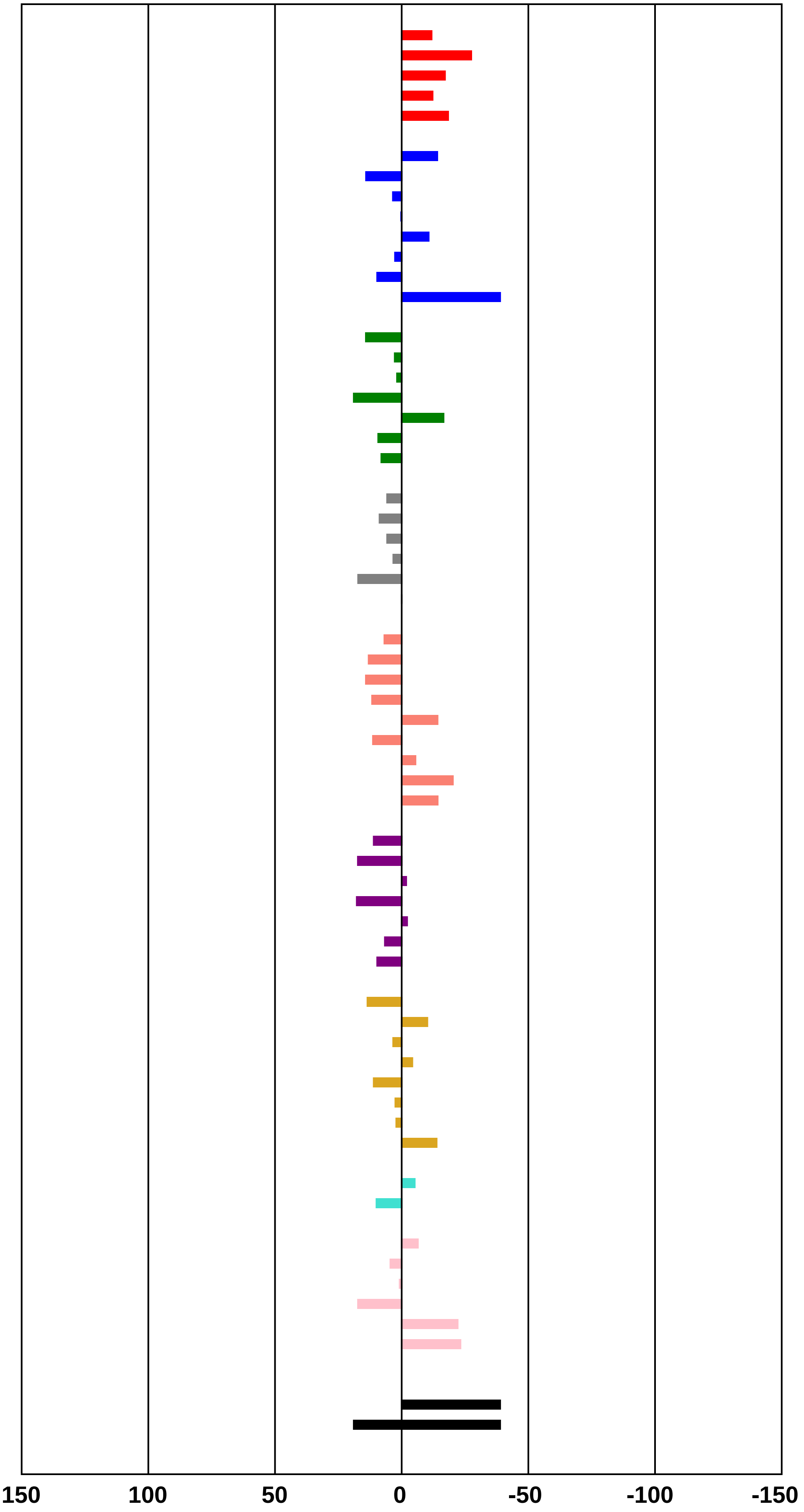

Developmental Therapeutics Program

One Dose Mean Graph

NSC: D-800133 / 1	Conc: 1.00E-5 Molar	Test Date: Aug 21, 2017
Experiment ID: 1708OS57	Product 4bJ	Report Date: Sep 05, 2017

Panel/Cell Line	Growth Percent
Leukemia	
CCRF-CEM	90.73
K-562	71.92
MOLT-4	81.24
RPMI-8226	90.22
SR	77.35
Non-Small Cell Lung Cancer	
A549/ATCC	83.12
EKVX	108.30
HOP-62	90.09
HOP-92	88.36
NCI-H23	96.92
NCI-H322M	99.71
NCI-H460	105.78
NCI-H522	68.76
Colon Cancer	
COLO 205	105.48
HCC-2998	95.49
HCT-116	98.10
HCT-15	105.84
HT29	82.56
KM12	106.18
SW-620	100.88
CNS Cancer	
SF-268	101.09
SF-295	103.92
SF-539	98.24
SNB-19	93.01
SNB-75	97.50
U251	82.69
Melanoma	
LOX IMVI	105.04
MALME-3M	108.52
M14	108.34
MDA-MB-435	104.68
SK-MEL-2	89.27
SK-MEL-28	103.28
SK-MEL-5	102.14
UACC-257	73.76
UACC-62	92.46
Ovarian Cancer	
IGROV1	106.56
OVCAR-3	112.40
OVCAR-4	97.02
OVCAR-5	102.06
OVCAR-8	92.35
NCI/ADR-RES	101.60
SK-OV-3	103.62
Renal Cancer	
786-0	102.08
A498	90.55
ACHN	97.41
CAKI-1	89.81
RXF 393	98.72
SN12C	91.58
TK-10	102.44
UO-31	90.29
Prostate Cancer	
PC-3	89.97
DU-145	108.72
Breast Cancer	
MCF7	93.75
MDA-MB-231/ATCC	98.30
HS 578T	94.07
BT-549	107.75
T-47D	87.15
MDA-MB-468	89.65
Mean	95.84
Delta	27.08
Range	43.64

Mean Growth Percent - Growth Percent

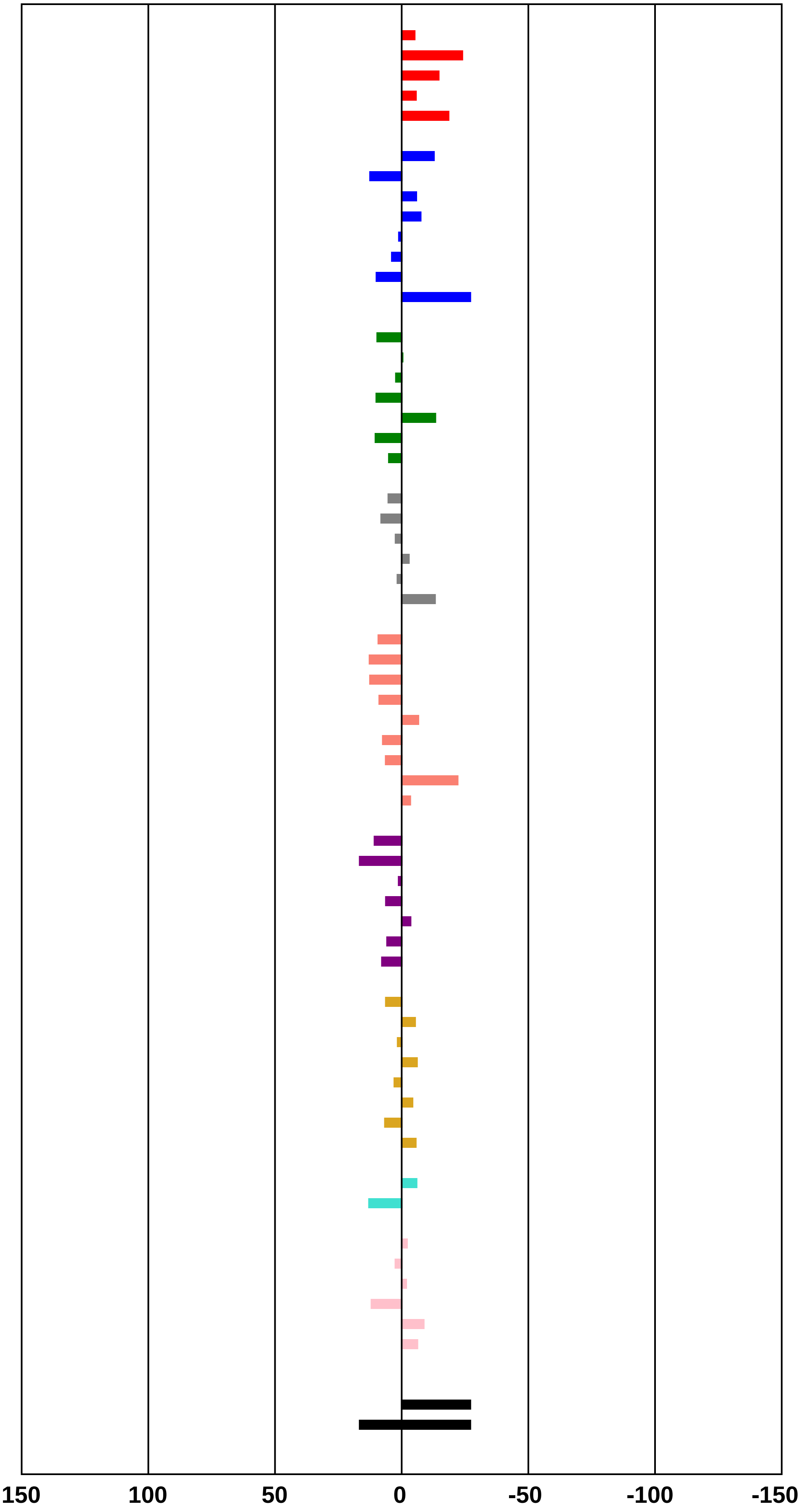

Supplement: Supplementary file 1 [file ijms-26-01717-s001.zip › ijms-3377882-supplementary.pdf]
